# DNA Barcoding Reveals Cryptic Diversity within Commercially Exploited Indo-Malay Carangidae (Teleosteii: Perciformes)

**DOI:** 10.1371/journal.pone.0049623

**Published:** 2012-11-29

**Authors:** Tun Nurul Aimi Mat Jaafar, Martin I. Taylor, Siti Azizah Mohd Nor, Mark de Bruyn, Gary R. Carvalho

**Affiliations:** 1 Molecular Ecology and Fisheries Genetics Laboratory, Environment Centre Wales, Bangor University, Bangor, Gwynedd, United Kingdom; 2 School of Biological Sciences, Universiti Sains Malaysia, Pulau Pinang, Malaysia; 3 Faculty of Fisheries and Aqua Industry, Universiti Malaysia Terengganu, Terengganu, Malaysia; Consiglio Nazionale delle Ricerche (CNR), Italy

## Abstract

**Background:**

DNA barcodes, typically focusing on the *cytochrome oxidase I gene (COI)* in many animals, have been used widely as a species-identification tool. The ability of DNA barcoding to distinguish species from a range of taxa and to reveal cryptic species has been well documented. Despite the wealth of DNA barcode data for fish from many temperate regions, there are relatively few available from the Southeast Asian region. Here, we target the marine fish Family Carangidae, one of the most commercially-important families from the Indo-Malay Archipelago (IMA), to produce an initial reference DNA barcode library.

**Methodology/Principal Findings:**

Here, a 652 bp region of *COI* was sequenced for 723 individuals from 36 putative species of Family Carangidae distributed within IMA waters. Within the newly-generated dataset, three described species exhibited conspecific divergences up to ten times greater (4.32–4.82%) than mean estimates (0.24–0.39%), indicating a discrepancy with assigned morphological taxonomic identification, and the existence of cryptic species. Variability of the mitochondrial DNA *COI* region was compared within and among species to evaluate the *COI* region's suitability for species identification. The trend in range of mean K2P distances observed was generally in accordance with expectations based on taxonomic hierarchy: 0% to 4.82% between individuals within species, 0% to 16.4% between species within genera, and 8.64% to 25.39% between genera within families. The average Kimura 2-parameter (K2P) distance between individuals, between species within genera, and between genera within family were 0.37%, 10.53% and 16.56%, respectively. All described species formed monophyletic clusters in the Neighbour-joining phylogenetic tree, although three species representing complexes of six potential cryptic species were detected in Indo-Malay Carangidae; *Atule mate, Selar crumenophthalmus* and *Seriolina nigrofasciata*.

**Conclusion/Significance:**

This study confirms that *COI* is an effective tool for species identification of Carangidae from the IMA. There were moderate levels of cryptic diversity among putative species within the central IMA. However, to explain the hypothesis of species richness in the IMA, it is necessary to sample the whole family across their broad geographic range. Such insights are helpful not only to document mechanisms driving diversification and recruitment in Carangidae, but also to provide a scientific framework for management strategies and conservation of commercially-important fisheries resources.

## Introduction

Spectacular biodiversity exists in tropical marine ecosystems. One mega-diverse tropical region, where the ranges of many tropical marine species overlap, is the centre of maximum marine biodiversity of the Indo-Malay Archipelago (IMA) [1]. Various hypotheses giving rise to this extraordinary species richness have been proposed [2], though two in particular have been widely addressed [3]–[5]: the Centre-of-Overlap and the Centre-of-Origin hypotheses, both of which postulate contrasting patterns of species ranges and distribution of species richness. The former proposes geographic isolation and allopatric speciation with midpoint ranges of species distributions falling on each side of the IMA, with overlap across the IMA. Large scale genetic structure is expected to result from geographic isolation, and cryptic species may be expected to exhibit allopatric distribution ranges, potentially overlapping in the IMA. The Centre-of-Origin hypothesis proposes speciation centred in the IMA, with midpoint ranges of species distributions occurring within the IMA. Large scale genetic structure is expected to be shallow as a consequence of high connectivity and larval dispersal across the IMA. Since the IMA encompassess the centre of the distributional range of the target taxa studied here, the Carangidae, we test whether there is any evidence of highly divergent cryptic lineages in sympatry, as predicted by the Centre-of-Origin hypothesis.

Given that only a small fraction of all global species have been formally described, between 1.5–1.8 million out of an estimated 10 million [6], efforts to catalogue and understand drivers of biodiversity need to be prioritised. Research on cryptic species has increased recently with studies [5], [7]–[8] indicating the frequent occurrence of cryptic species occurring within and outside the IMA. One of the problems associated with identifying cryptic species is that many taxonomic protocols rely on phenotypic characters, and require lengthy and detailed inspection of the specimens [9]. Such traditional methods of identifying, naming and classifying organisms are largely based on visible morphology. Misidentification of economically important species in cryptic species-complexes can result in inaccurate data collection potentially leading to the overexploitation of stocks [10]. Therefore, in addition to disclosing potential drivers of diversification, accurate identification at the species-level is vital to ensure the successful management of commercially important fish stocks in IMA waters, and here, a DNA barcoding database can play an important role.

The introduction of the DNA barcoding approach, which utilises a short, standardised gene region [11] to identify species [12]–[17] has been shown to be useful in solving taxonomic ambiguities. Hebert *et al.* [11] proposed that within species, DNA sequences would be more similar than that among different species, and that this ‘barcoding gap’ could be used to delimit species. To date, the *Cytochrome Oxidase subunit I (COI)* mitochondrial protein-coding gene has been accepted widely as a practical, standardized species-level barcode for the majority of the animal kingdom [18]. The main goal of DNA barcoding is to facilitate rapid identification of potentially unidentified taxa in global biodiversity assessment and conservation, including cryptic and microscopic taxa, and organisms with morphologically ambiguous characters [11]. DNA barcoding has also focused on the development of a global barcoding database [19] as a species identification tool for large taxonomic assemblages of animals, representing a quick and easy method for non-specialists to identify disparate specimens. The identification process through DNA barcoding is relatively straight-forward, and depends upon the quantifiable matching of *COI* sequences from unknown specimens with previously documented and archived voucher specimens. Where marked discordance is found in the *COI* sequences of test and reference specimens, additional taxonomic and related studies are undertaken to assess likelihood of discovering novel taxa [20].

To date, many barcoding projects involving various organisms from different geographic regions can be accessed from the public barcode library, the Barcode of Life Data Systems (www.barcodinglife.com) [19]. Despite the wealth of DNA barcode information for fish from many temperate regions [21]–[24], there are relatively few data available from Southeast Asian waters, an area exceptionally rich in biodiversity. DNA barcoding should prove useful for rapid biodiversity assessment [25] in this region, where significant levels of biodiversity loss are escalating [1]. Our study provides the first barcode records for 723 specimens representing 36 putative species from Carangidae sampled from waters of the IMA. Variability of *COI* was compared both within and among species to evaluate its suitability for species identification. Samples for assaying the *COI* barcodes were analysed and compared with field-based morphological species identifications and additional molecular data from other geographical regions were obtained from GenBank and the BOLD System. Such analyses may identify hidden diversity in Carangidae, where such diversity exists.

The family Carangidae encompasses fishes whose body size ranges from small (TL = 16 cm) to large (TL = 250 cm) and body shapes vary from elongate and fusiform to deep and strongly compressed [26]. This diverse family of marine fishes are known variously by common names such as jacks, trevallies, amberjacks, pompanos, scads, kingfish, pilotfish, queenfishes and rainbow runner [27]. Carangids represent an important food source and play a significant role in the commercial fisheries industry in Southeast Asia [27]. All members, small or large are considered as edible protein and can be caught in large numbers every year (ca. 1,556,578 tonnes in 2010) [28]. Despite their high economic value and ecological importance, the taxonomy of Carangids remains poorly understood [29]. FishBase citations include many synonyms, which indicate taxonomic ambiguities in Carangids [30] due to morphological and meristic similarities across species, as well as plasticity in body shape, size and colour patterns [7], [31]. In addition, Carangids typically display significant changes in morphology and pigmentation during growth [32], and such changes have likely lead to misidentification of specimens, and contributed to general taxonomic confusion. An interesting example of change with growth occurs in juveniles of African pompano (*Alectis ciliaris*), which are easily recognized by the presence of long filaments trailing from five to six dorsal and anal fins. As fish grow larger, these filaments shorten and eventually disappear [33]. The exact biological mechanism behind such developmental changeis unclear, as is the function of the filaments. Carangid eggs and newly hatched larvae are also difficult to distinguish from the eggs and larvae of many other families of marine fishes [34], making it difficult to map spawning grounds and identify ichthyoplankton [35]. Pigmentation changes during development in Carangid larvae and its diagnostic value is thereby of limited value for species identification [36]. Unambiguous delineation of such apparent phenotypic plasticity is required not only for taxonomy and systematics, but also is of critical importance for fisheries management, trade and conservation purposes. *Cytochrome oxidase subunit 1 (COI)* has been shown to accurately discriminate between closely related species of various animal groups [13], [15]–[17], [37], and is applied here to examine the integrity of species delineation in Carangids.

## Materials and Methods

### Sampling

**Figure 1:**
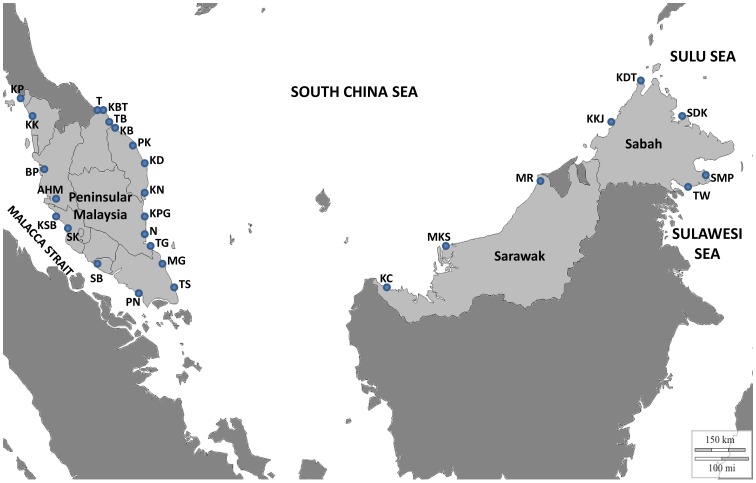
Distribution of locations for the 845 specimens sampled along the coast of Malaysia. See Table S1 for detailed sampling information.

We collected 845 Carangidae specimens from four geographic regions within the IMA: South China Sea, Straits of Malacca, Sulu Sea and Celebes Sea. The samples were collected from several fish landing sites during two field trips; from October to November 2009, and from June to July 2010 (Figure 1). Specimens encompassed 39 putative species and 18 genera from the Family Carangidae. Sample collections included tissue sampling for genetic analysis, as well as collection of whole specimens (adult fish and larvae) for storage as barcode voucher specimens. All samples were preserved in 99% ethanol. Digital photographs of all fishes were taken immediately and voucher specimens were tagged according to museum ID number and archived in the South China Sea Museum, Universiti Malaysia Terengganu (www.umt.edu.my). All details regarding collection dates, collection sites with geographical coordinates, taxonomy and vouchers can be found in the Barcode of Life Data System website (BOLD, www.barcodinglife.com) [19] under project ‘DNA Barcoding of Malaysian Fish’ (DBMF). At least five individuals of each species were collected from each sampling site depending on their abundance. Few specimens were collected in some low abundance species (<5), while those that were abundant enabled the collection of more individuals (up to 75), with 29/36 species having sample sizes of >5 individuals. All fishes were identified based on morphology, with the help of expert local taxonomists in most cases, FAO-Fisheries Identification Sheets [38] and identification books published by the Department of Fisheries Malaysia [39]–[40].

Fin clips were removed from the right pectoral fin of each fish and preserved in 99% ethanol. Fish specimens were then placed in ice, frozen on site and transported to South China Sea Museum, University Malaysia Terengganu. Fin clips were sent to the Canadian Centre for DNA Barcoding (CCDB), University of Guelph Ontario, Canada for further processing. Total genomic DNA was extracted from fin clips of 39 putative species and PCR amplifications performed using the procedure of [41]. Following the CBOL standard practice, *COI* genes were sequenced in both directions. All *COI* sequences and trace files have been deposited in the Barcode of Life Data System (www.barcodinglife.com) under a project named ‘DNA Barcoding of Malaysian Marine Fish’ (DBMF). Sequences have also been deposited in GenBank (Table S1, Supporting information).

### Data validation

For this study, we collected 845 individuals of Carangidae. However, a total of 110 individuals generated sequences of insufficient quality to be uploaded into the BOLD system, and were therefore not considered further. After exclusion of these 110 individuals, our *COI* data base encompasses a total of 735 sequences. Incorrect taxonomic classification may affect divergence assessment of our data set. Therefore, all 735 sequences were aligned and a Neighbour Joining tree produced using the BOLD platform. A small percentage (1.63%) of samples which did not cluster with their own taxa had their photographs reviewed and this revealed potential misidentification. The remaining three species (*Carangoides oblongus*, *Carangoides orthogrammus*, *Trachinotus blochii*) with one specimen each, failed to PCR amplify, leaving a total of 36 species in the data set. Subsequently, we analysed 723 sequences from 36 species and 18 genera from Family Carangidae.

### COI divergence assessment

The diversity assessment for Carangidae were analysed from the data set with 723 sequences, 18 genera and 36 putative species. The Kimura 2-parameter (K2P) distance measure has become the most widely used in barcoding studies [42] and was employed here. Genetic distances between specimens were calculated for each intraspecies, intragenus and intrafamily with the ‘Distance Summary’ command implemented by BOLD. K2P was also used for Neighbour-joining (NJ) analysis (Figure S1, Supporting Information), using the BOLD Management and Analysis System. All sequences were aligned using the MUSCLE algorithm in the software programme MEGA5 [43], and the amino acid translation was examined to ensure that no gaps or stop codons were present in the alignment. NJ analyses were conducted using 1000 bootstrap replicates. Nucleotide divergences of *COI* variation across 36 species of Carangidae were analysed. Genetic distances among specimens were calculated for each intraspecies and intragenus pairwise comparison with the ‘Distance Summary’ analysis in BOLD. Other analytical tools in BOLD such as Nearest Neighbour, Identify Unknown and BOLD Identification System were also applied to the data. The Maximum Likelihood (ML) approach was also conducted by determining the highest likelihood tree bootstrapped 1000 times using RAxML 7.2.8 [44] (Figure S2, Supporting Information). Bayesian phylogenetic analyses was conducted in Mr Bayes v3.2.1 [45], though outputs showed no convergence after 10 million generations. We thus discarded these analyses and present here only NJ and ML analyses. We also employed the recently described bioinformatics tool, Automatic Barcode Gap Discovery (ABGD) [46] for species delimitation analysis. ABGD automatically detects the breaks in the distribution of genetic pairwise distances, referred to as the ‘barcode gap’ and uses it to partition the data. The method proposes a standard definition of the barcode gap and can be used even when the two distributions overlap to partition the data set into candidate species. The same species therefore should be grouped in the same partition.

Additional *COI* sequences from GenBank and BOLD Systems were added to compare *COI* sequences of 23 selected species from this study with conspecifics from West (South Africa, Mozambique, Iran, India and Turkey) and East (Australia, Philippines, China, Japan, Hawaii, French Polynesia and Mexico) of the IMA. All species and GenBank accession numbers are listed in Table S1.

## Results

### General findings

*COI* barcodes were recovered for a total of 36 species and 18 genera from the Family Carangidae, for the first time from the IMA. The number of sequences per species varied between 1 (*Carangoides gymnosthetus*) for species that were rare, to 75 (*Selar crumenophtalmus*) for species that were abundant in Malaysian waters. Thus a total of 723 *COI* barcodes with an average length of 652 bp were obtained for this commercially important fish family. No insertions/deletions, heterozygous sites or stop codons were observed, supporting the view that all of the amplified sequences constitute functional mitochondrial *COI* sequences.

### COI divergence assessment

**Figure 2:**
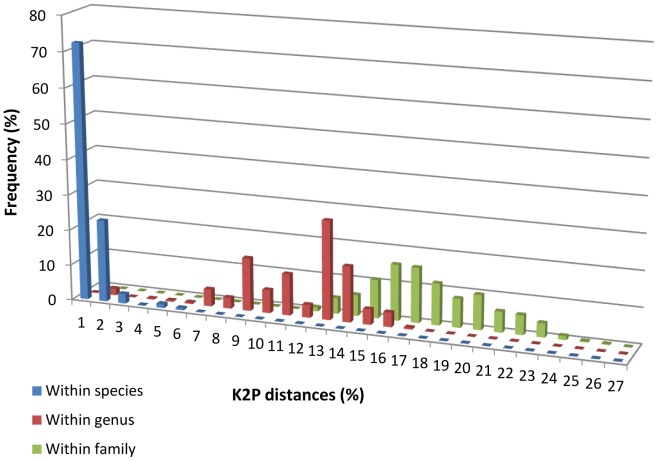
Frequency distributions of *COI* K2P distances (%) intraspecies, intragenus and intrafamily. 36 species, 18 genera and 1 family.

*COI* nucleotide divergences were calculated for the dataset of 723 sequences of 36 species and 18 genera. Sample sizes and mean divergences at various taxonomic levels are given in Table 1. As expected, genetic divergence increased progressively with higher taxonomic level: 0% to 4.82% between individuals within species, 0% to 16.4% between species within genera, and 8.64% to 25.39% between genera within family, which support a marked change in genetic divergence at the species boundary (Figure 2).

**Table 1:** Kimura 2-parameter (K2P) distances between Indo-Malay Carangidae.

Comparison within	*n*	Number of comparisons	Min (%)	Mean (%)	Max (%)	SE (%)
Species	36	13445	0	0.37	4.82	0.006
Genus	18	10680	0	10.53	16.4	0.028
Family	1	240503	8.64	16.56	25.39	0.006

The average within species K2P distance is 0.37% with far less, 0.00% for *Carangoides ferdau*, *Gnathanodon speciosus* and *Trachinotus baillonii*. The latter estimates were largely due to the low number of specimens collected, and all specimens were from the same landing site (n = 1–4). *Atropus atropos* (1.13%) and *Seriolina nigrofasciata* (1.79%) displayed slightly higher divergence rates than average (Table 2). The average congeneric distance was 10.53%, which was higher than the conspecific distance. The congeneric distances were lowest among queen fishes, *Scomberoides* (7.52% - 3 species), followed by *Caranx* (7.53% - 3 species); *Alepes* (8.84% - 4 species); *Decapterus* (8.89% - 3 species); *Alectis* (11.37% - 2 species); *Carangoides* (11.66% - 7 species) and the highest variation observed in the genus *Selar* (12.25% - 2 species) (Table 3).

**Table 2:** Intraspecific nucleotide K2P distances for 36 species of Indo-Malay Carangidae.

Species	No. of sequences (n)	Mean K2P distance (%)
*Alectis ciliaris (Bloch, 1787)*	8	0.16
*Alectis indicus (Rüppell, 1830)*	10	0.17
*Alepes djedaba (Forsskål, 1775)*	31	0.25
*Alepes kleinii (Bloch, 1793)*	11	0.16
*Alepes melanoptera (Swainson, 1839)*	15	0.40
*Alepes vari (Cuvier, 1833)*	13	0.16
*Atropus atropos (Bloch & Schneider, 1801)*	13	1.13
*Atule mate (Cuvier, 1833)*	67	0.34
*Carangoides bajad (Forsskål, 1775)*	26	0.39
*Carangoides chrysophrys (Cuvier, 1833)*	19	0.33
*Carangoides dinema (Bleeker, 1851)*	6	0.03
*Carangoides ferdau (Forsskål, 1775)*	2	0.00
*Carangoides fulvoguttatus (Forsskål, 1775)*	3	0.21
*Carangoides gymnostethus* * *(Cuvier, 1833)*	1	N/A
*Carangoides hedlandensis (Whitley, 1934)*	3	0.31
*Carangoides malabaricus (Bloch & Schneider, 1801)*	33	0.54
*Caranx ignobilis (Forsskål, 1775)*	6	0.51
*Caranx sexfasciatus Quoy & Gaimard, 1825*	8	0.16
*Caranx tille Cuvier, 1833*	9	0.07
*Decapterus kurroides Bleeker, 1855*	10	0.09
*Decapterus macrosoma Bleeker, 1851*	26	0.08
*Decapterus maruadsi (Temminck & Schlegel, 1843)*	24	0.15
*Elagatis bipinnulata (Quoy & Gaimard, 1825)*	8	0.22
*Gnathanodon speciosus (Forsskål, 1775)*	4	0.00
*Megalaspis cordyla (Linnaeus, 1758)*	63	0.53
*Parastromateus niger (Bloch, 1795)*	51	0.30
*Scomberoides commersonnianus Lacepède, 1801*	17	0.56
*Scomberoides tala (Cuvier, 1832)*	11	0.08
*Scomberoides tol (Cuvier, 1832)*	32	0.09
*Selar boops (Cuvier, 1833)*	40	0.37
*Selar crumenophthalmus (Bloch, 1793)*	75	0.39
*Selaroides leptolepis (Cuvier, 1833)*	39	0.18
*Seriola dumerili (Risso, 1810)*	4	0.31
*Seriolina nigrofasciata (Rüppell, 1829)*	9	1.79
*Trachinotus baillonii (Lacepède, 1801)*	4	0.00
*Uraspis uraspis (Günther, 1860)*	22	0.67

* only 1 sequence available.

**Table 3:** Congeneric nucleotide K2P distances for seven genera in Indo-Malay Carangidae

Genus	No. of sequences (n)	Mean K2P distance (%)
*Alectis*	18	11.37
*Alepes*	70	8.84
*Carangoides*	93	11.66
*Caranx*	23	7.53
*Decapterus*	60	8.89
*Scomberoides*	60	7.52
*Selar*	115	12.25

**Figure 3:**
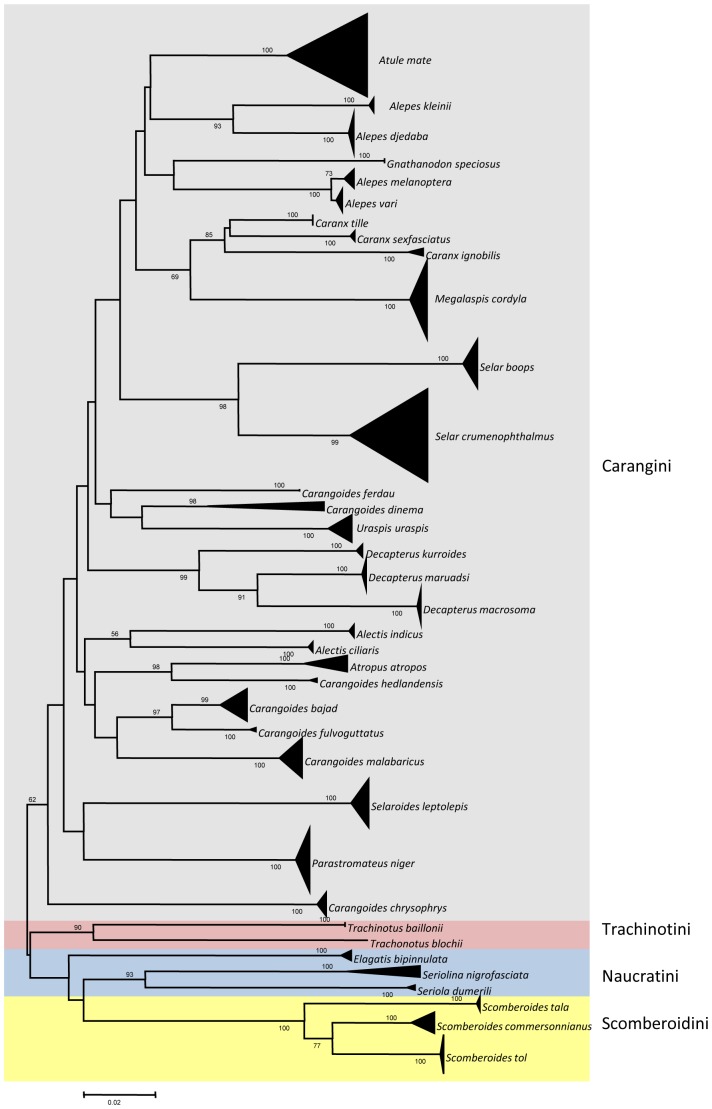
Neighbour-joining tree (K2P distance) of 36 Carangidae species. All species formed monophyletic clusters. Only bootstrap values greater than 50 are shown.

Mean intraspecific K2P divergence of Indo-Malay Carangidae was 0.37% (range 0–4.82%), while mean congeneric species K2P divergence was 10.53% (range 0–16.4%) (Table 1). In the NJ analyses, the majority of recognised species formed monophyletic clusters (Figure 3). Such patterns illustrate the utility of *COI* sequences to provide species-level resolution. All assemblages of conspecific individuals had bootstrap support of 98–100%. However, in ML analyses (Figure S2, Supporting Information), four species which have been identified as different species formed two monophyletic clusters; *Alepes vari* grouped together with *Alepes melanoptera*, while *Carangoides bajad* grouped in the same cluster as *Carangoides gymnosthetus*. These results were also supported by the ABGD analysis (Figure S3, Supporting Information).

### Cryptic diversity in the Indo-Malay Archipelago

**Figure 4:**
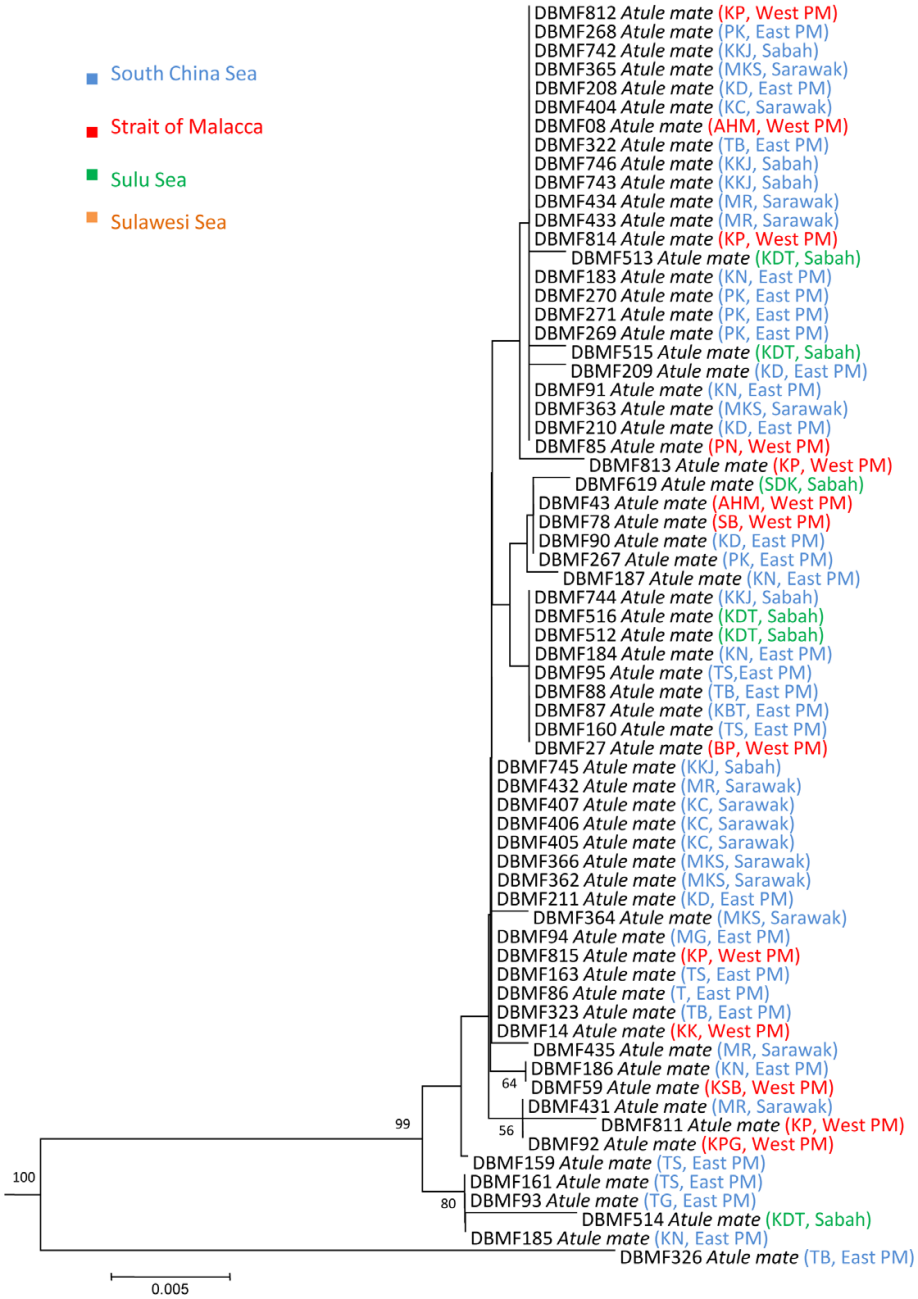
Neighbour-joining tree (K2P distance) of 67 *COI* sequences of *Atule mate*. Only bootstrap values greater than 50 are shown. Sample ID for the Barcode of Life Database (BOLD, www.barcodinglife.org) provided.

**Figure 5:**
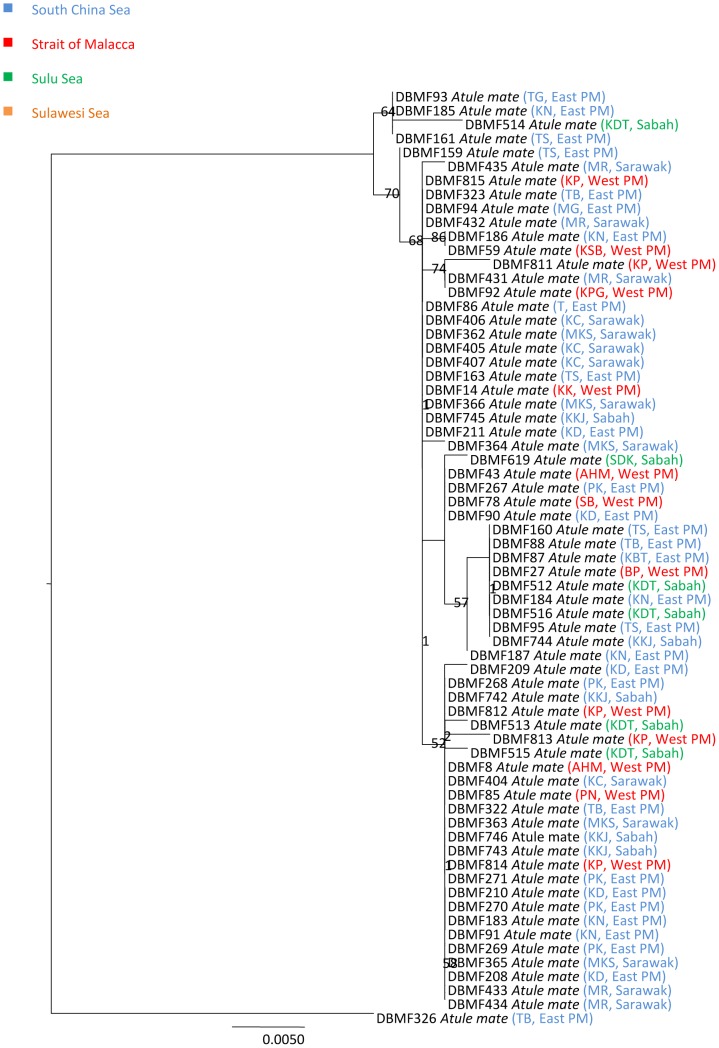
Maximum-likelihood tree of 67 *COI* sequences of *Atule mate*. Only bootstrap values greater than 50 are shown. Sample ID for the Barcode of Life Database (BOLD, www.barcodinglife.org) provided.

**Figure 6:**
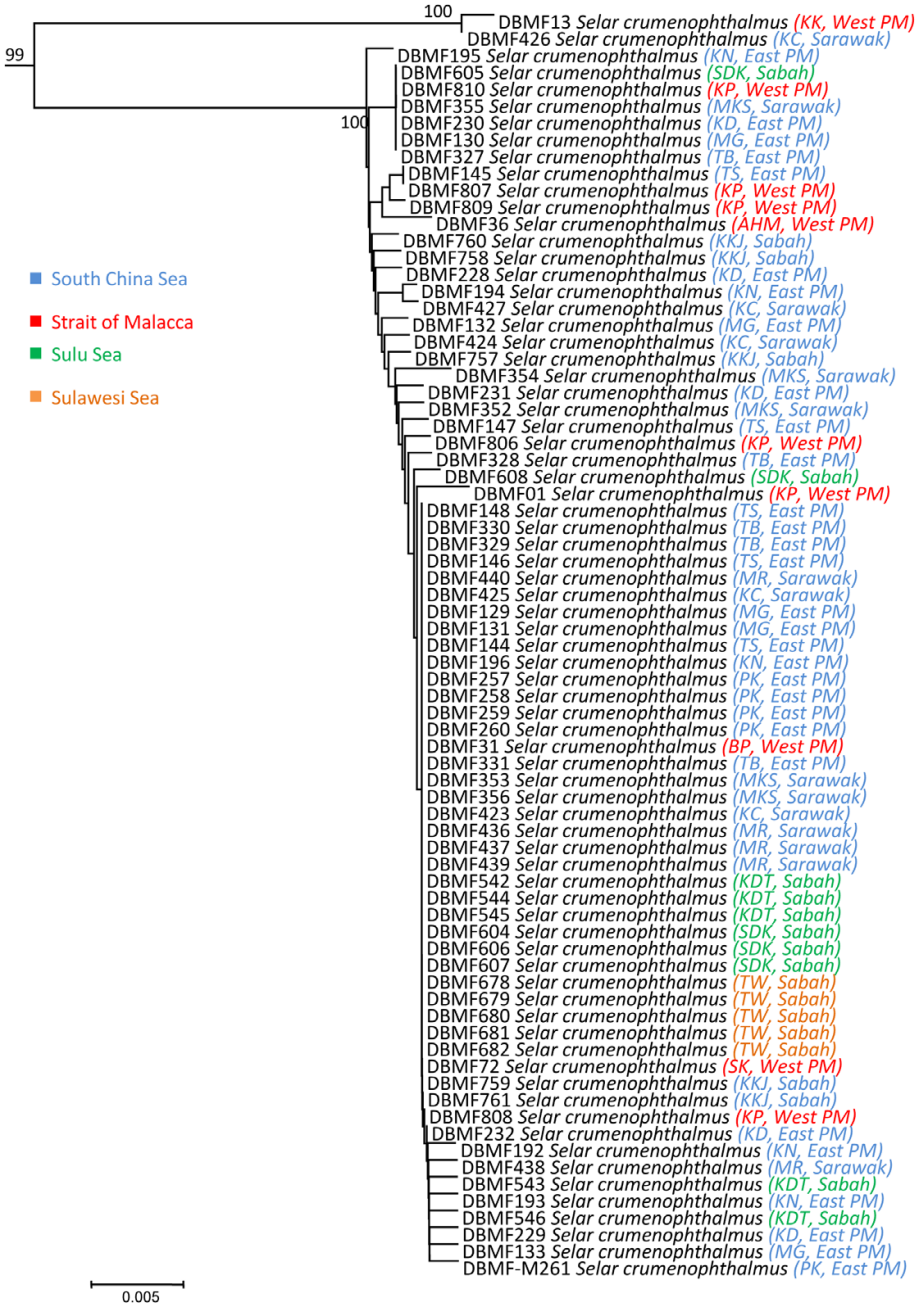
Neighbour-joining tree (K2P distance) of 75 *COI* sequences of *Selar crumenophthalmus*. Only bootstrap values greater than 50 are shown. Sample ID for the Barcode of Life Database (BOLD, www.barcodinglife.org) provided.

**Figure 7:**
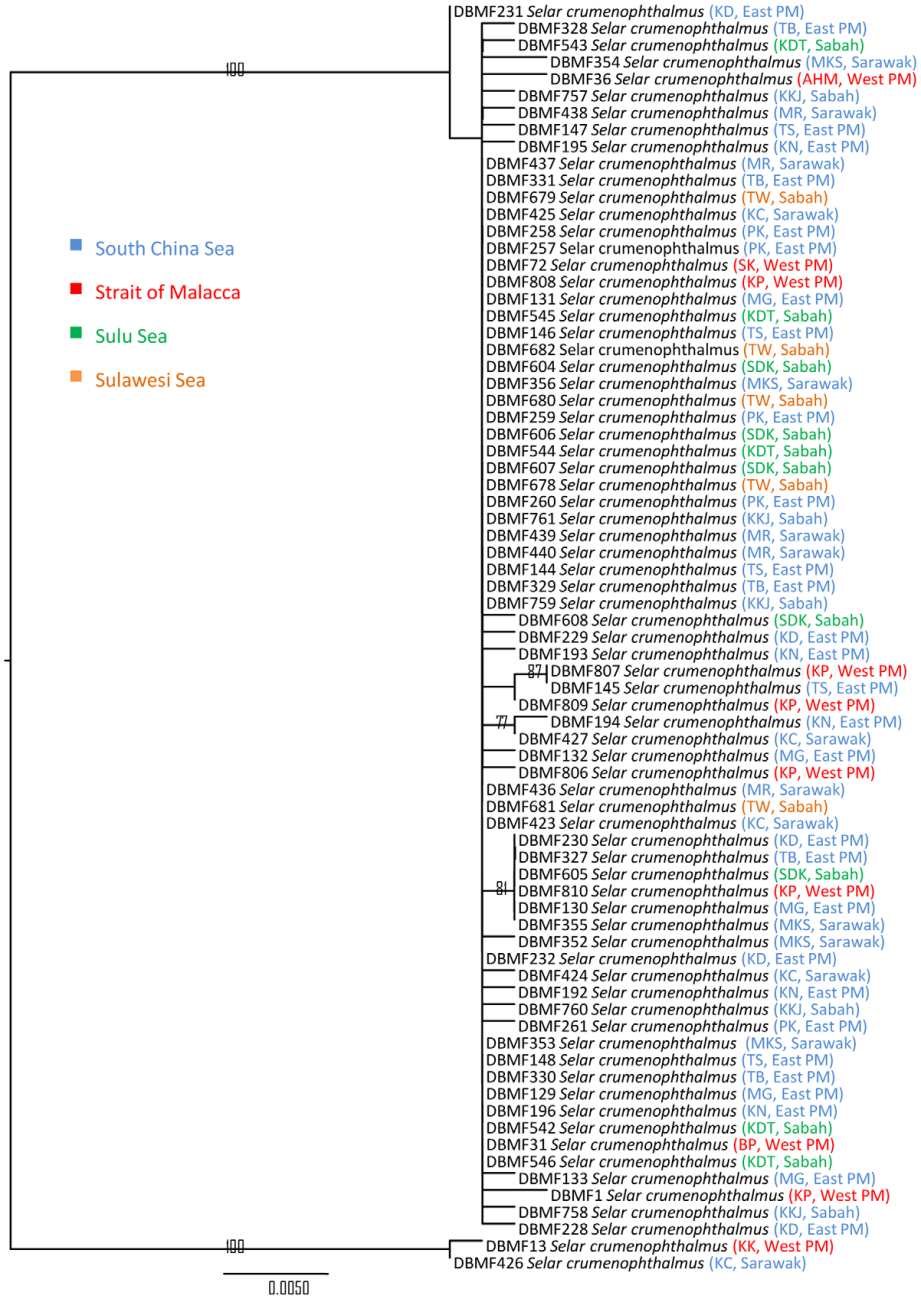
Maximum-likelihood tree of 75 *COI* sequences of *Selar crumenophthalmus*. Only bootstrap values greater than 50 are shown. Sample ID for the Barcode of Life Database (BOLD, www.barcodinglife.org) provided.

**Figure 8:**
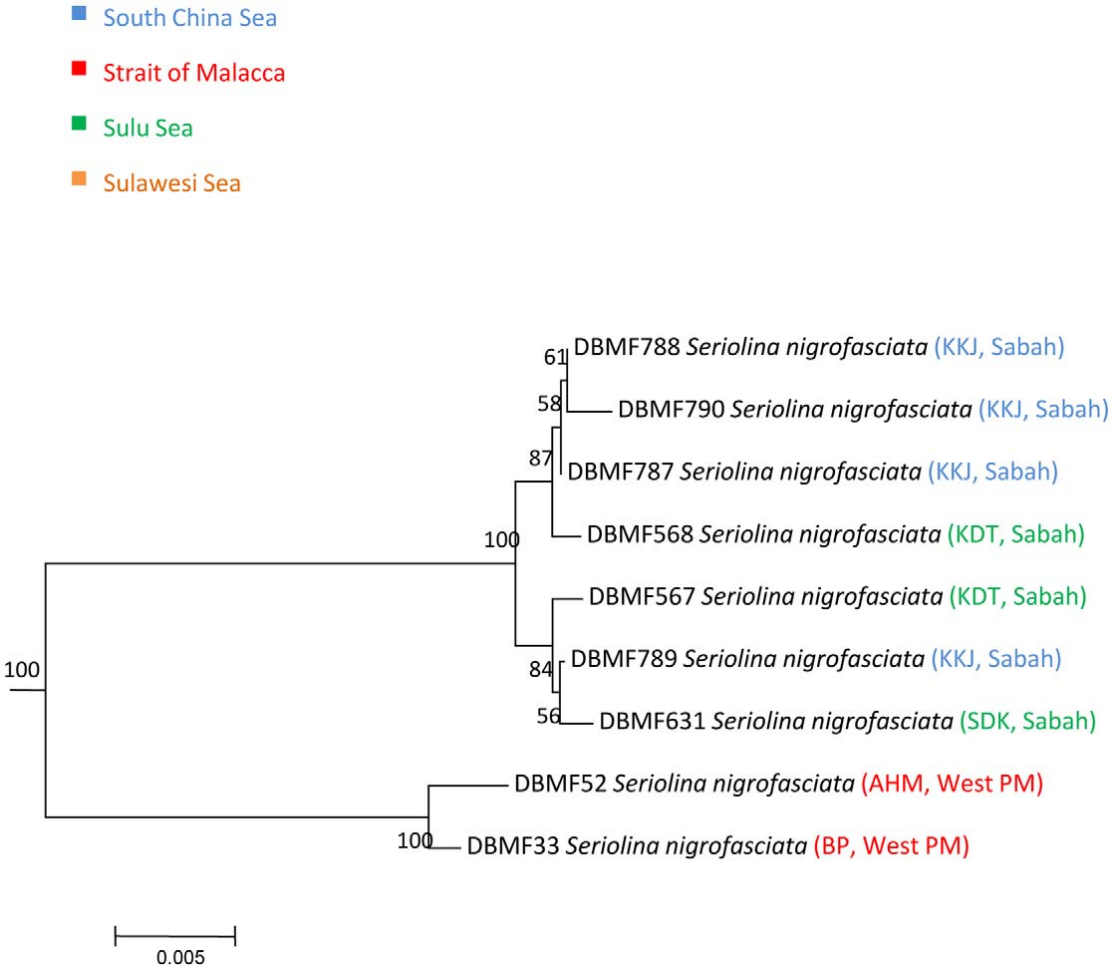
Neighbour-joining tree (K2P distance) of 9 *COI* sequences of *Seriolina nigrofasciata*. Only bootstrap values greater than 50 are shown. Sample ID for the Barcode of Life Database (BOLD, www.barcodinglife.org) provided.

**Figure 9:**
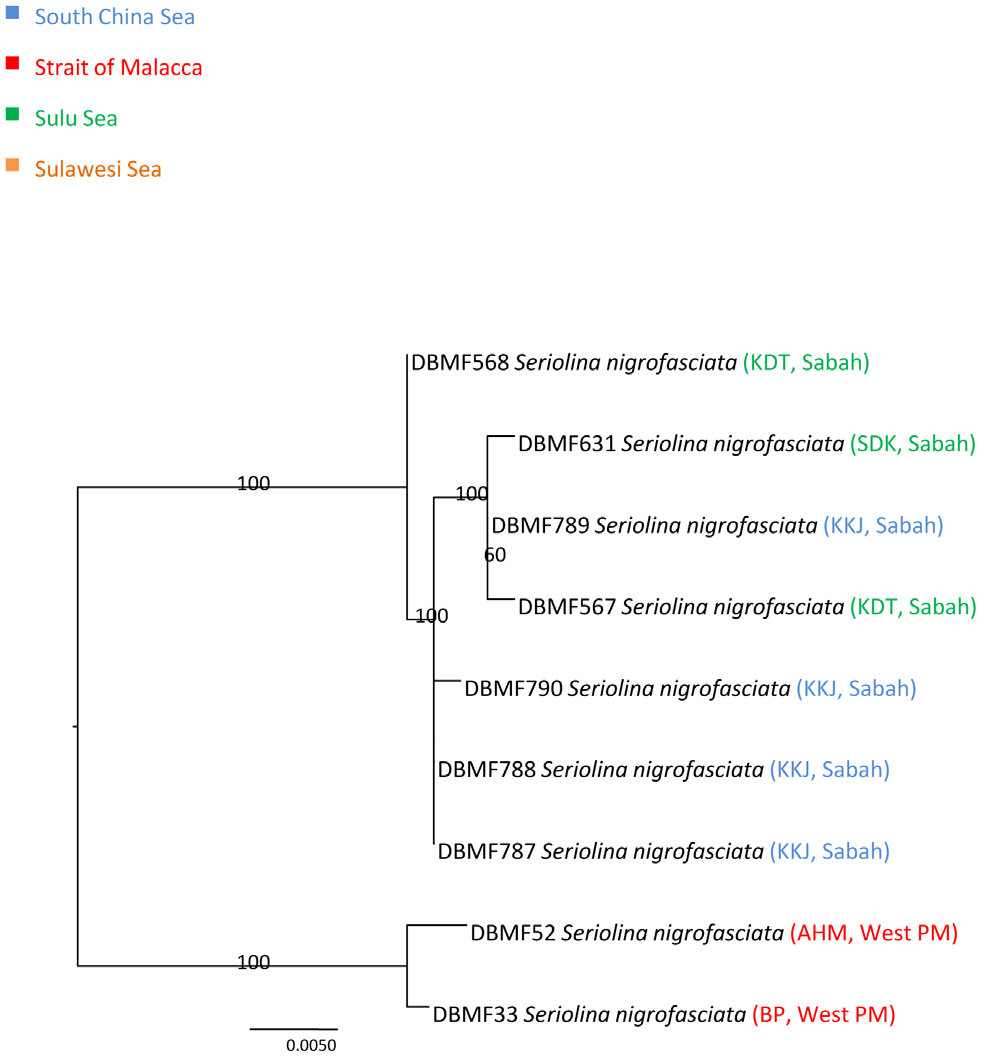
Maximum-likelihood tree of 9 *COI* sequences of *Seriolina nigrofasciata*. Only bootstrap values greater than 50 are shown. Sample ID for the Barcode of Life Database (BOLD, www.barcodinglife.org) provided.

In three species, we detected deep divergences among individuals that had been assigned to a single taxon. Closer observation of the data associated with *Atule mate, Selar crumenopthalmus* and *Seriolina nigrofasciata* showed maximum intraspecific divergences of 4.82%, 4.66% and 4.32% (Table S2, Supporting information) respectively, revealing that the specimens of each in fact formed two clusters in both NJ and ML analyses with 99–100% bootstrap support (Figures 4–9). Divergent as they were, members of the two clusters nonetheless were more similar to each other than to members of any other species in our data set.

### Atule mate

Phylogenetic analyses also revealed two clusters generated from 67 *Atule mate* samples (Figures 4 and 5). Mean K2P distance within species was 0.34% with a maximum of 4.82% nucleotide divergence. These clusters were separated by a mean *COI* nucleotide divergence of 4%. Cluster I, the major lineage containing most specimens from all sampling regions exhibited no obvious geographic structuring, and was strongly supported with a bootstrap value of 100% in the NJ tree. In contrast, Cluster II is a minor lineage, containing only a single specimen from Tok Bali, Kelantan, eastern Peninsular Malaysia (TB). Phylogenetic trees constructed from control region and Rag 1 (nuclear DNA) data were consistent with the pattern observed at *COI* (unpublished data).

### Selar crumenophthalmus

Seventy five specimens of *Selar crumenophthalmus* also formed two clusters in the *COI* NJ and ML trees (Figures 6 and 7). Mean K2P distance within species was 0.39% with a maximum of 4.66% nucleotide divergence. Cluster I comprised the majority of the specimens with a high bootstrap value of 100%, while Cluster II comprised only two individuals from Kuala Kedah, western Peninsular Malaysia (KK) and Kuching, Sarawak (KC), also supported by a high bootstrap value of 100%. A mean pairwise distance of 4.5% separated these two clusters. No geographic pattern was apparent.

### Seriolina nigrofasciata

Mean K2P distance within species of *Seriolina nigrofasciata* was 1.79% with a maximum nucleotide divergence of 4.32%. Nine specimens of this species formed two clusters with Cluster I comprising the specimens from Kota Kinabalu (KKJ) and Kudat (KDT), Sabah. Cluster II comprised only two individuals from Hutan Melintang (AHM) and Bagan Panchor (BP) from western Peninsular Malaysia, supported by a bootstrap value of 100% (Figures 8 and 9). A mean pairwise distance of 4.32% separated these two clusters.

*COI* sequences of 23 species examined here were compared with data available from conspecifics from other geographical regions (downloaded from BOLD and GenBank), and NJ trees were produced for each species (Figure S4, Supporting Information). From these 23 widespread species, 13 species exhibited shallow genetic structure with mixed *COI* lineages found on either side of the IMA. The other 10 species each formed two clusters with maximum nucleotide divergences ranging from 2.68–8.81%.

## Discussion

According to the Fish Barcode of Life project database (www.fishbol.org), in 2009, 69% of species from Family Carangidae had been barcoded in Southeast Asia, but with some species represented by only a single sample. DNA barcodes had increased to 83% with 43 species having more than four barcodes in November 2011, including our data. We sequenced a total of 723 specimens from 18 genera and 36 species of Carangidae at the *COI* barcoding region. Thirty-three species could be accurately discriminated, illustrating the effectiveness of the *COI* gene for identifying commercial marine fish from Malaysian waters, and providing resolution at the species-level. However, the remaining three species showed deep divergences (4.32–4.82%) among individuals that had been assigned to a single taxon. Divergent as they were, members of the two clusters nonetheless were more similar to each other than members of any other species. These high sympatric divergences suggested that each may comprise cryptic species.

The average K2P distance of individuals within species was 0.37% compared with 10.53% for species within genera. Hence, congeneric species were approximately 28 times more divergent than conspecific individuals. The mean intraspecific K2P distance observed was similar to the intraspecific K2P distance reported for marine (0.24–0.39%) [23] and freshwater species (0.3–0.45%) [21]. The branch length among species tends to be much deeper than among conspecific individuals leading to a gap in the distribution of the pairwise distance among conspecific individuals and among species that has been referred to as the barcoding gap [47]. Mean divergence among species within families increased to 16.56%. These data show that increasing genetic divergence was observed with increasing taxonomic level, supporting a marked difference in genetic divergence at the species boundary. Such patterns in taxonomic distribution of nucleotide divergence supports observations obtained by Ward *et al.* [22] with genetic distances of 0.39% for conspecifics, 9.93% for congenerics and 15.46% for confamilial species of 754 *COI* sequences representing 207 species of Australian fish. Data obtained in our study were also consistent with those obtained by Asgharian *et al.* [48] for 187 individuals of Persian Gulf fish with values of 0.18%, 12% and 17.43% among conspecifics, congenerics and confamilial species respectively.

The NJ tree revealed that species identification and phylogenetic relationships based on morphological evidence and molecular methods are broadly consistent. However, the ML analyses suggested that four species might comprise only two taxonomic units, as these four species formed two reciprocally monophyletic clusters in the ML tree (*Alepes vari* and *Alepes melanoptera*; *Carangoides bajad* and *Carangoides gymnosthetus*). ABGD analysis supports such findings as the same pattern was evident. Further analyses should be undertaken by the inclusion of more genes and larger sample sizes to confirm the relationships across these four species. Phylogenetic relationships among species with NJ analysis were clearly established, and individuals from the same species were grouped in the same taxonomic cluster with 98–100% bootstrap support. According to Smith-Vaniz [49], Carangidae can be categorized into four tribes based on morphological evidence; the Carangini, Trachinotini, Naucratini and Scomberoidini. All species of Carangidae in our study grouped according to Smith-Vaniz [49] (Figure 3), with the larger clade consisting of specimens known as jacks, trevallies, scads and black pomfret (tribe Carangini). The second clade comprised the other three tribes; Trachinotini, Naucratini and Scomberoidini, representing pompano, amberjacks and queen fishes. The emergence of these four tribes in NJ analyses clearly demonstrates that there is deep phylogenetic signal in the relatively short *COI* sequence fragments, even though barcode analysis seeks only to delineate species boundaries. However, the phylogenetic relationships of these four tribes remain questionable [50]–[52], and our approach in isolation is not sufficient to explore such questions in depth. Additional gene regions, together with more comprehensive analytical methods including parsimony, ML and Bayesian approaches should be included to resolve such apparently deep phylogenetic relationships.

**Figure 10:**
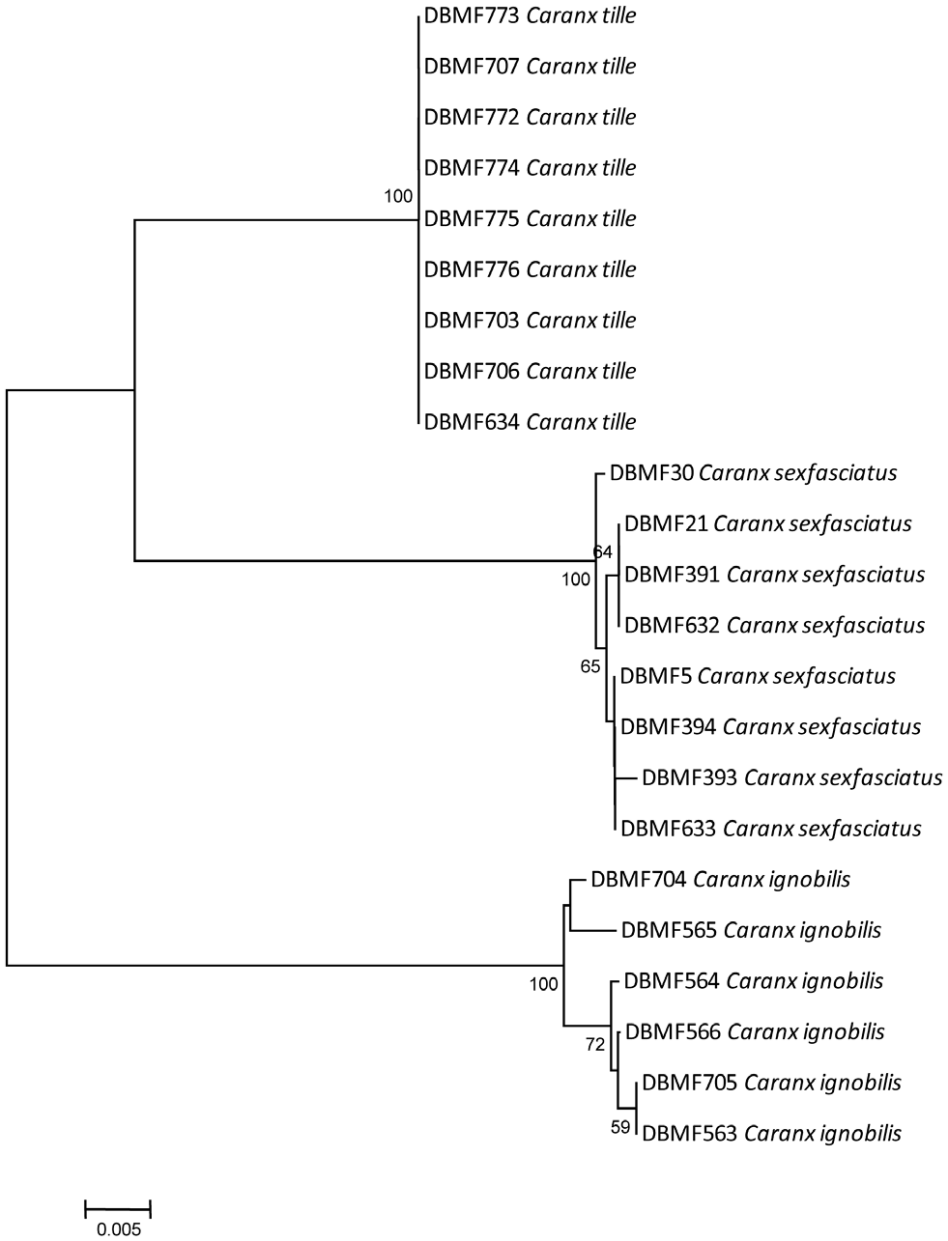
Neighbour-joining tree (K2P distance) of genus *Caranx*. Only bootstrap values greater than 50 are shown. Sample ID for the Barcode of Life Database (BOLD, www.barcodinglife.org) provided.

The main goals of DNA barcoding are to assign unknown specimens to a species category, and enhance the disclosure of new and cryptic species. DNA barcoding also facilitates identification, particularly in microscopic, diverse life history stages, and other organisms with complex or inaccessible morphology [11]. Furthermore, the approach is also able to discriminate species of highly similar morphology. The Carangids, which are morphologically very similar, such as the three species (*Caranx ignobilis, Caranx sexfasciatus* and *Caranx tille*), form a sister grouping (Figure 10). Because of such high similarity, they are sometimes misidentified. However, DNA barcoding discriminated these *Caranx* samples effectively on all occasions. Three distinct clusters were formed, separating the three species by an average interspecific distance of 7.53%, and average intraspecific distances of 0.51%, 0.16% and 0.07% for *Caranx ignobilis, Caranx sexfasciatus* and *Caranx tille*, respectively.

The Indo-Malay Archipelago has long been considered as the centre of maximum marine biodiversity [53]–[54]. A few studies based on the *COI* marker have discovered high cryptic diversity in coral reef fish around this region [5], [22]. Several hypotheses have been proposed to explain the remarkably high diversity found in this region: 1) centre of origin [55], 2) centre of accumulation [56], and 3) centre of overlap [57]. Hypotheses 1 and 2 have recently been raised [5] to explain speciation and dispersal of marine species in the Indo-Malay Archipelago. It might either be the result of diversification within the region and subsequent species dispersed into peripheral areas (Centre of Origin), or the result of an overlap of the faunas from the Indian and Pacific Oceans (Centre of Overlap).

A few studies have identified high levels of cryptic species occurring within and outside the IMA [5], [22], , though here, we detected only a moderate frequency of potentially cryptic species within commercially exploited Indo-Malay Carangidae. Small sample size, bias in range of species collected, and restricted geographic ranges may have lead to fewer cryptic species being identified compared to previous studies. By increasing the geographic sampling range, more cyrptic diversity will likely be detected [5], [22], [58]. The majority of the species in Carangidae have a pelagic lifestyle. Interestingly, within marine ecosystems, most diversity is benthic, with such organisms including 98% of species diversity, while the remaining 2% are pelagic [59]. Three species representing complexes of six potential cryptic species were detected in Indo-Malay Carangidae; *Atule mate, Selar crumenophthalmus* and *Seriolina nigrofasciata*. All NJ and ML trees identified two separate lineages but only *Seriolina nigrofasciata* showed allopatric divergence, with the Sabah lineage separated from the West Peninsular Malaysia lineage by 4.32%. The other two showed sympatric divergences with both clusters consisting of geographically mixed *COI* lineages.

Comparison of COI sequences of 23 species from this study with conspecific sequences available from other geographical regions [48], [60] revealed the existence of several more complexes of potentially cryptic species from outside the IMA. Using the ABGD analysis [46], 10 lineages were flagged as candidate cryptic species. Four recognised species (*Caranx sexfasciatus, Decapterus maruadsi, Gnathanodon speciosus* and *Seriolina nigrofasciata*) each comprised two lineages exhibiting allopatric divergences with a maximum nucleotide divergence of 7.1%, 2.7%, 3.8% and 4.35%, respectively (Figure S4, Supporting Information). As for *Seriolina nigrofasciata*, additional sequences from India and Iran clustered together, and samples from West Peninsular Malaysia were clearly separated from the western part of the IMA together with Sabah (Borneo), representing an additional complex of two potential cryptic species. Such findings are consistent with large faunal discontinuities between Indian and Pacific Ocean ichthyofaunas as a consequence of geographic isolation on each side of IMA, as discussed by Springer and Williams [61]. However, our data is not sufficient to explain the hypothesis of species richness in the IMA. To explore hypotheses of species diversification it is necessary to sample the whole family across their broad geographic range.

Our study has examined only one family with different lifestyles, body shape and body size. We did not identify any significant association between genetic distances and these biological characteristics (pers. obs.). However, Zemlak *et al.* [58] used *COI* to examine patterns of divergences between fish species representing different lifestyles from opposite sides of the Indian Ocean. They detected deep divergences between certain inshore taxa, with the inshore taxa (mean *COI* divergence = 0.51%) exhibiting significantly higher levels of putative cryptic species than the offshore (mean *COI* divergence = 0.26%) fish. Such deep divergences were more representative of patterns in congeneric species than among populations of a single species, highlighting the possible genetic isolation of presumed cosmopolitan species. Out of the 35 species studied by Zemlak *et al.* [58], the one member of Carangidae sampled, the needlescaled queenfish (*Scomberoides tol*), appears to represent a broadly distributed sibling species pair whose distribution spans the Indian Ocean. Such findings reinforce the need in such *COI* barcoding studies to sample throughout the extremes of the geographic range to investigate the extent of hidden diversity in marine fauna.

### Conclusion

In conclusion, the establishment of an Indo-Malay Carangidae *COI* barcoding library presented here contributes to the global DNA barcoding effort to document and catalogue the diversity of life, particularly with regard to conservation and management applications. We anticipate that the accumulation of biodiversity data will help drive and inform effective planning and monitoring of conservation and fisheries programmes in the Indo-Malay region. Intensification of industrial and commercial activities in Malaysian waters renders the biodiversity of the region highly vulnerable to threats and degradation. Therefore, such data are helpful not only to document mechanisms driving population structuring and recruitment in Carangidae, but also provide a scientific framework in support of effective management strategies and the conservation of commercially-important fisheries resources.

## Supporting Information

Figure S1 **Taxon ID Tree of Carangidae generated by BOLD.** Neighbour-joining tree (Kimura 2-parameter, pairwise deletion). A total of 723 sequences from 36 species and 18 genera were analysed.
(PDF)

Figure S2 **Phylogenetic tree from Maximum-likelihood analysis.** Numbers above the branches represent bootstrap support based on 1000 replicates.
(PDF)

Figure S3 **Tree corresponding to partition detected by ABGD method.**
(PDF)

Figure S4 **Taxon ID Tree of 23 widespread Carangidae species generated by MEGA5 including conspecifics from other geographical regions.** Neighbour-joining tree (Kimura 2-parameter, pairwise deletion).
(PDF)

Table S1 **Specimen data and GenBank accession numbers used in this study.**
(DOC)

Table S2 **K2P distances of Indo-Malay Carangidae.**
(DOC)
