# Supplementary material for: DNA Barcoding Reveals Cryptic Diversity within Commercially Exploited Indo-Malay Carangidae (Teleosteii: Perciformes)
Source: PLoS One. 2012 Nov 29;7(11):e49623. doi: 10.1371/journal.pone.0049623 (PMC3510217; doi:10.1371/journal.pone.0049623)
Supplement: Figure S1 — Taxon ID Tree of Carangidae generated by BOLD. Neighbour-joining tree (Kimura 2-parameter, pairwise deletion). A total of 723 sequences from 36 species and 18 genera were analysed. (PDF) [file pone.0049623.s001.pdf]

BOLD TaxonID Tree

Title : GenBank submission DBMF [DATASET-DBMFGB1]

Date : 25-July-2012

Data Type : Nucleotide

Distance Model : Kimura 2 Parameter

Marker : COI-5P

Codon Positions : 1st, 2nd, 3rd

Labels : SampleID

Filters : Length > 200

Colorization : [blue]=Stop Codons [red]=Contamination or misidentification

Sequence Count : 723

Species count : 36

Genus count : 18

Family count : 1

Unidentified : 0

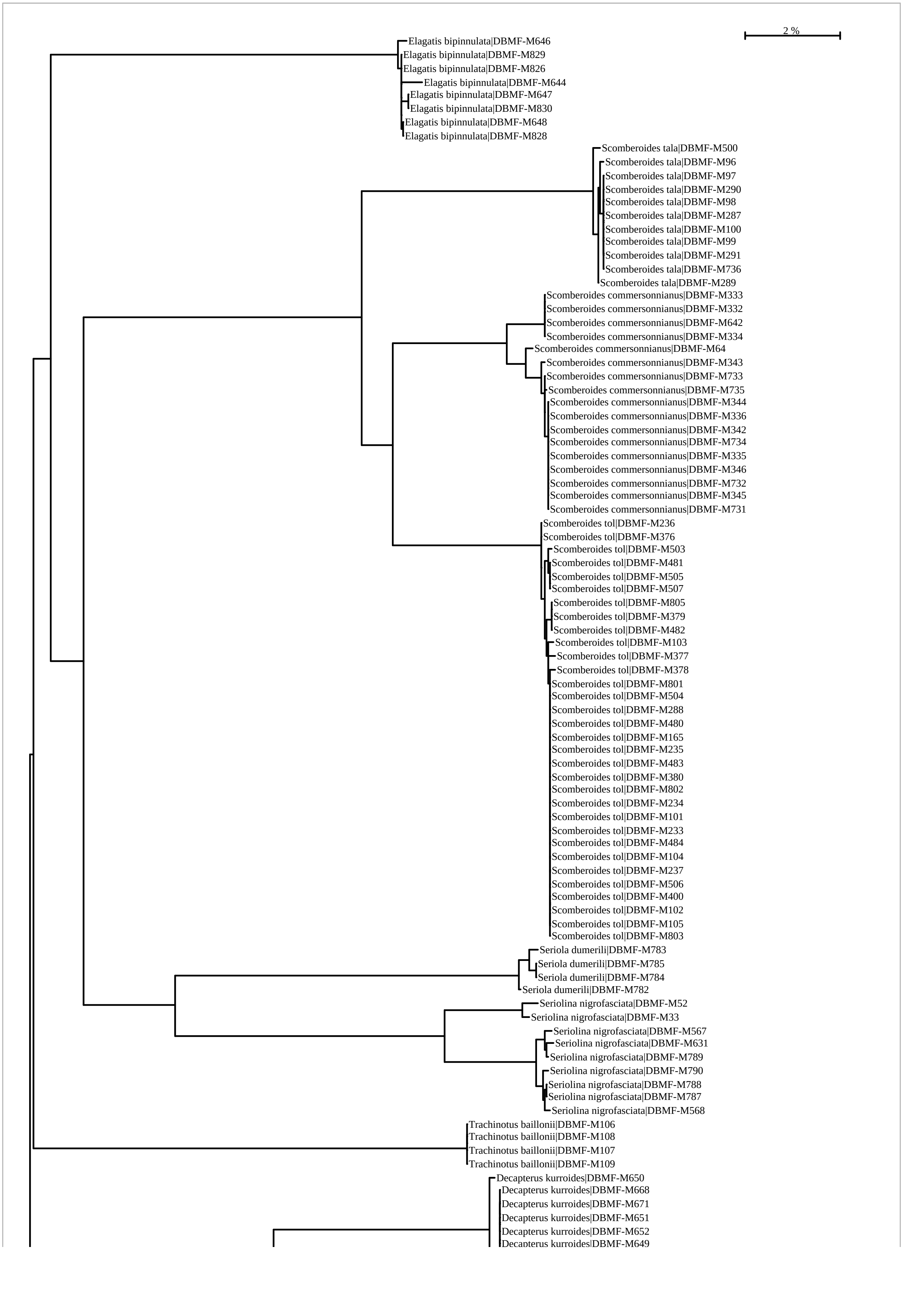
2 %
Elagatis bipinnulata|DBMF-M646
Elagatis bipinnulata|DBMF-M829
Elagatis bipinnulata|DBMF-M826
Elagatis bipinnulata|DBMF-M644
Elagatis bipinnulata|DBMF-M647
Elagatis bipinnulata|DBMF-M830
Elagatis bipinnulata|DBMF-M648
Elagatis bipinnulata|DBMF-M828
Scomberoides tala|DBMF-M500
Scomberoides tala|DBMF-M96
Scomberoides tala|DBMF-M97
Scomberoides tala|DBMF-M290
Scomberoides tala|DBMF-M98
Scomberoides tala|DBMF-M287
Scomberoides tala|DBMF-M100
Scomberoides tala|DBMF-M99
Scomberoides tala|DBMF-M291
Scomberoides tala|DBMF-M736
Scomberoides tala|DBMF-M289
Scomberoides commersonnianus|DBMF-M333
Scomberoides commersonnianus|DBMF-M332
Scomberoides commersonnianus|DBMF-M642
Scomberoides commersonnianus|DBMF-M334
Scomberoides commersonnianus|DBMF-M64
Scomberoides commersonnianus|DBMF-M343
Scomberoides commersonnianus|DBMF-M733
Scomberoides commersonnianus|DBMF-M735
Scomberoides commersonnianus|DBMF-M344
Scomberoides commersonnianus|DBMF-M336
Scomberoides commersonnianus|DBMF-M342
Scomberoides commersonnianus|DBMF-M734
Scomberoides commersonnianus|DBMF-M335
Scomberoides commersonnianus|DBMF-M346
Scomberoides commersonnianus|DBMF-M732
Scomberoides commersonnianus|DBMF-M345
Scomberoides commersonnianus|DBMF-M731
Scomberoides tol|DBMF-M236
Scomberoides tol|DBMF-M376
Scomberoides tol|DBMF-M503
Scomberoides tol|DBMF-M481
Scomberoides tol|DBMF-M505
Scomberoides tol|DBMF-M507
Scomberoides tol|DBMF-M805
Scomberoides tol|DBMF-M379
Scomberoides tol|DBMF-M482
Scomberoides tol|DBMF-M103
Scomberoides tol|DBMF-M377
Scomberoides tol|DBMF-M378
Scomberoides tol|DBMF-M801
Scomberoides tol|DBMF-M504
Scomberoides tol|DBMF-M288
Scomberoides tol|DBMF-M480
Scomberoides tol|DBMF-M165
Scomberoides tol|DBMF-M235
Scomberoides tol|DBMF-M483
Scomberoides tol|DBMF-M380
Scomberoides tol|DBMF-M802
Scomberoides tol|DBMF-M234
Scomberoides tol|DBMF-M101
Scomberoides tol|DBMF-M233
Scomberoides tol|DBMF-M484
Scomberoides tol|DBMF-M104
Scomberoides tol|DBMF-M237
Scomberoides tol|DBMF-M506
Scomberoides tol|DBMF-M400
Scomberoides tol|DBMF-M102
Scomberoides tol|DBMF-M105
Scomberoides tol|DBMF-M803
Seriola dumerili|DBMF-M783
Seriola dumerili|DBMF-M785
Seriola dumerili|DBMF-M784
Seriola dumerili|DBMF-M782
Seriolina nigrofasciata|DBMF-M52
Seriolina nigrofasciata|DBMF-M33
Seriolina nigrofasciata|DBMF-M567
Seriolina nigrofasciata|DBMF-M631
Seriolina nigrofasciata|DBMF-M789
Seriolina nigrofasciata|DBMF-M790
Seriolina nigrofasciata|DBMF-M788
Seriolina nigrofasciata|DBMF-M787
Seriolina nigrofasciata|DBMF-M568
Trachinotus baillonii|DBMF-M106
Trachinotus baillonii|DBMF-M108
Trachinotus baillonii|DBMF-M107
Trachinotus baillonii|DBMF-M109
Decapterus kurroides|DBMF-M650
Decapterus kurroides|DBMF-M668
Decapterus kurroides|DBMF-M671
Decapterus kurroides|DBMF-M651
Decapterus kurroides|DBMF-M652
Decapterus kurroides|DBMF-M649

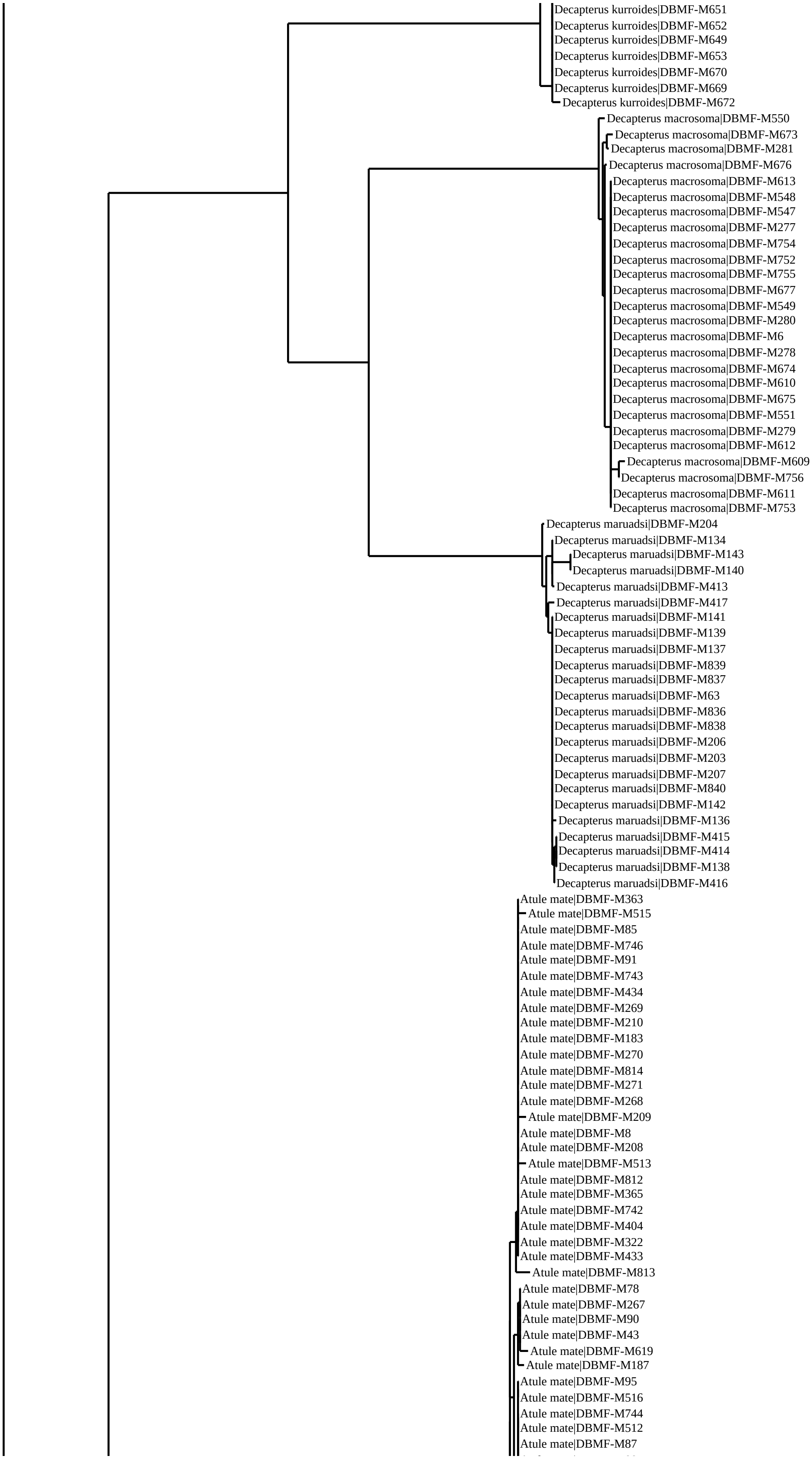
Decapterus kurroides|DBMF-M651
Decapterus kurroides|DBMF-M652
Decapterus kurroides|DBMF-M649
Decapterus kurroides|DBMF-M653
Decapterus kurroides|DBMF-M670
Decapterus kurroides|DBMF-M669
Decapterus kurroides|DBMF-M672
Decapterus macrosoma|DBMF-M550
Decapterus macrosoma|DBMF-M673
Decapterus macrosoma|DBMF-M281
Decapterus macrosoma|DBMF-M676
Decapterus macrosoma|DBMF-M613
Decapterus macrosoma|DBMF-M548
Decapterus macrosoma|DBMF-M547
Decapterus macrosoma|DBMF-M277
Decapterus macrosoma|DBMF-M754
Decapterus macrosoma|DBMF-M752
Decapterus macrosoma|DBMF-M755
Decapterus macrosoma|DBMF-M677
Decapterus macrosoma|DBMF-M549
Decapterus macrosoma|DBMF-M280
Decapterus macrosoma|DBMF-M6
Decapterus macrosoma|DBMF-M278
Decapterus macrosoma|DBMF-M674
Decapterus macrosoma|DBMF-M610
Decapterus macrosoma|DBMF-M675
Decapterus macrosoma|DBMF-M551
Decapterus macrosoma|DBMF-M279
Decapterus macrosoma|DBMF-M612
Decapterus macrosoma|DBMF-M609
Decapterus macrosoma|DBMF-M756
Decapterus macrosoma|DBMF-M611
Decapterus macrosoma|DBMF-M753
Decapterus maruadsi|DBMF-M204
Decapterus maruadsi|DBMF-M134
Decapterus maruadsi|DBMF-M143
Decapterus maruadsi|DBMF-M140
Decapterus maruadsi|DBMF-M413
Decapterus maruadsi|DBMF-M417
Decapterus maruadsi|DBMF-M141
Decapterus maruadsi|DBMF-M139
Decapterus maruadsi|DBMF-M137
Decapterus maruadsi|DBMF-M839
Decapterus maruadsi|DBMF-M837
Decapterus maruadsi|DBMF-M63
Decapterus maruadsi|DBMF-M836
Decapterus maruadsi|DBMF-M838
Decapterus maruadsi|DBMF-M206
Decapterus maruadsi|DBMF-M203
Decapterus maruadsi|DBMF-M207
Decapterus maruadsi|DBMF-M840
Decapterus maruadsi|DBMF-M142
Decapterus maruadsi|DBMF-M136
Decapterus maruadsi|DBMF-M415
Decapterus maruadsi|DBMF-M414
Decapterus maruadsi|DBMF-M138
Decapterus maruadsi|DBMF-M416
Atule mate|DBMF-M363
Atule mate|DBMF-M515
Atule mate|DBMF-M85
Atule mate|DBMF-M746
Atule mate|DBMF-M91
Atule mate|DBMF-M743
Atule mate|DBMF-M434
Atule mate|DBMF-M269
Atule mate|DBMF-M210
Atule mate|DBMF-M183
Atule mate|DBMF-M270
Atule mate|DBMF-M814
Atule mate|DBMF-M271
Atule mate|DBMF-M268
Atule mate|DBMF-M209
Atule mate|DBMF-M8
Atule mate|DBMF-M208
Atule mate|DBMF-M513
Atule mate|DBMF-M812
Atule mate|DBMF-M365
Atule mate|DBMF-M742
Atule mate|DBMF-M404
Atule mate|DBMF-M322
Atule mate|DBMF-M433
Atule mate|DBMF-M813
Atule mate|DBMF-M78
Atule mate|DBMF-M267
Atule mate|DBMF-M90
Atule mate|DBMF-M43
Atule mate|DBMF-M619
Atule mate|DBMF-M187
Atule mate|DBMF-M95
Atule mate|DBMF-M516
Atule mate|DBMF-M744
Atule mate|DBMF-M512
Atule mate|DBMF-M87

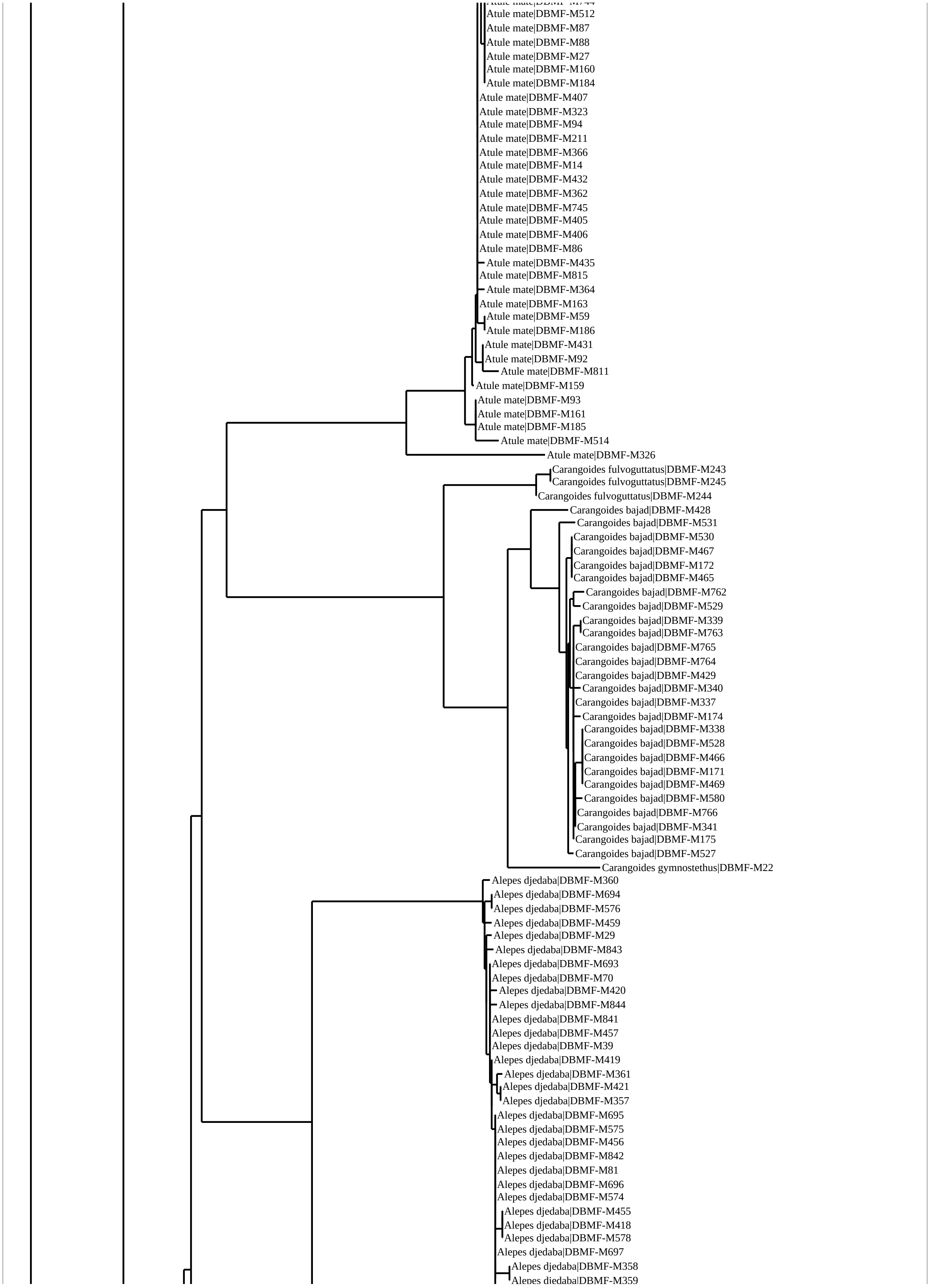
Atule mate|DBMF-M512
Atule mate|DBMF-M87
Atule mate|DBMF-M88
Atule mate|DBMF-M27
Atule mate|DBMF-M160
Atule mate|DBMF-M184
Atule mate|DBMF-M407
Atule mate|DBMF-M323
Atule mate|DBMF-M94
Atule mate|DBMF-M211
Atule mate|DBMF-M366
Atule mate|DBMF-M14
Atule mate|DBMF-M432
Atule mate|DBMF-M362
Atule mate|DBMF-M745
Atule mate|DBMF-M405
Atule mate|DBMF-M406
Atule mate|DBMF-M86
Atule mate|DBMF-M435
Atule mate|DBMF-M815
Atule mate|DBMF-M364
Atule mate|DBMF-M163
Atule mate|DBMF-M59
Atule mate|DBMF-M186
Atule mate|DBMF-M431
Atule mate|DBMF-M92
Atule mate|DBMF-M811
Atule mate|DBMF-M159
Atule mate|DBMF-M93
Atule mate|DBMF-M161
Atule mate|DBMF-M185
Atule mate|DBMF-M514
Atule mate|DBMF-M326
Carangoides fulvoguttatus|DBMF-M243
Carangoides fulvoguttatus|DBMF-M245
Carangoides fulvoguttatus|DBMF-M244
Carangoides bajad|DBMF-M428
Carangoides bajad|DBMF-M531
Carangoides bajad|DBMF-M530
Carangoides bajad|DBMF-M467
Carangoides bajad|DBMF-M172
Carangoides bajad|DBMF-M465
Carangoides bajad|DBMF-M762
Carangoides bajad|DBMF-M529
Carangoides bajad|DBMF-M339
Carangoides bajad|DBMF-M763
Carangoides bajad|DBMF-M765
Carangoides bajad|DBMF-M764
Carangoides bajad|DBMF-M429
Carangoides bajad|DBMF-M340
Carangoides bajad|DBMF-M337
Carangoides bajad|DBMF-M174
Carangoides bajad|DBMF-M338
Carangoides bajad|DBMF-M528
Carangoides bajad|DBMF-M466
Carangoides bajad|DBMF-M171
Carangoides bajad|DBMF-M469
Carangoides bajad|DBMF-M580
Carangoides bajad|DBMF-M766
Carangoides bajad|DBMF-M341
Carangoides bajad|DBMF-M175
Carangoides bajad|DBMF-M527
Carangoides gymnostethus|DBMF-M22
Alepes djedaba|DBMF-M360
Alepes djedaba|DBMF-M694
Alepes djedaba|DBMF-M576
Alepes djedaba|DBMF-M459
Alepes djedaba|DBMF-M29
Alepes djedaba|DBMF-M843
Alepes djedaba|DBMF-M693
Alepes djedaba|DBMF-M70
Alepes djedaba|DBMF-M420
Alepes djedaba|DBMF-M844
Alepes djedaba|DBMF-M841
Alepes djedaba|DBMF-M457
Alepes djedaba|DBMF-M39
Alepes djedaba|DBMF-M419
Alepes djedaba|DBMF-M361
Alepes djedaba|DBMF-M421
Alepes djedaba|DBMF-M357
Alepes djedaba|DBMF-M695
Alepes djedaba|DBMF-M575
Alepes djedaba|DBMF-M456
Alepes djedaba|DBMF-M842
Alepes djedaba|DBMF-M81
Alepes djedaba|DBMF-M696
Alepes djedaba|DBMF-M574
Alepes djedaba|DBMF-M455
Alepes djedaba|DBMF-M418
Alepes djedaba|DBMF-M578
Alepes djedaba|DBMF-M697
Alepes djedaba|DBMF-M358
Alepes djedaba|DBMF-M359

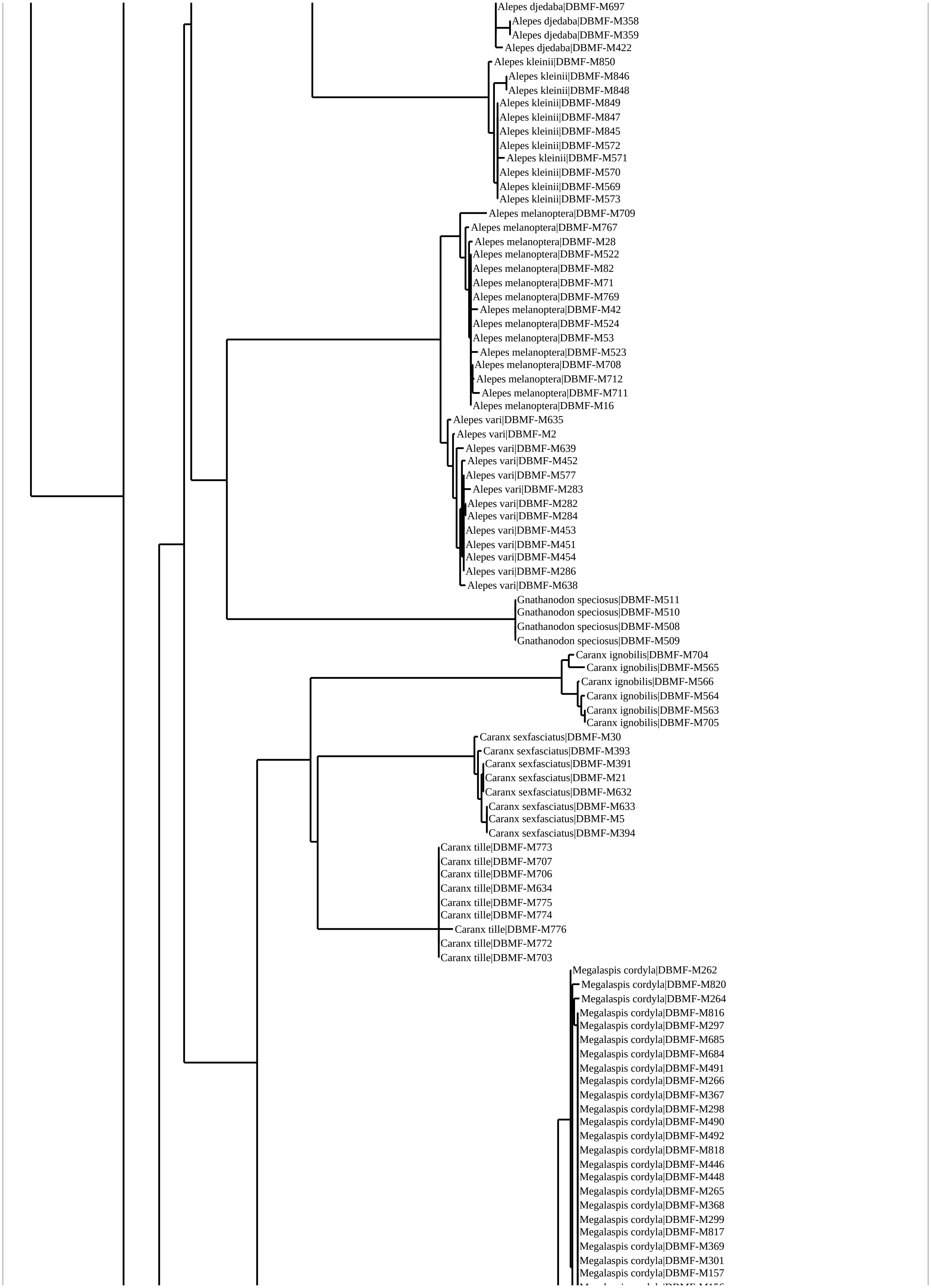
Alepes djedaba|DBMF-M697
Alepes djedaba|DBMF-M358
Alepes djedaba|DBMF-M359
Alepes djedaba|DBMF-M422
Alepes kleinii|DBMF-M850
Alepes kleinii|DBMF-M846
Alepes kleinii|DBMF-M848
Alepes kleinii|DBMF-M849
Alepes kleinii|DBMF-M847
Alepes kleinii|DBMF-M845
Alepes kleinii|DBMF-M572
Alepes kleinii|DBMF-M571
Alepes kleinii|DBMF-M570
Alepes kleinii|DBMF-M569
Alepes kleinii|DBMF-M573
Alepes melanoptera|DBMF-M709
Alepes melanoptera|DBMF-M767
Alepes melanoptera|DBMF-M28
Alepes melanoptera|DBMF-M522
Alepes melanoptera|DBMF-M82
Alepes melanoptera|DBMF-M71
Alepes melanoptera|DBMF-M769
Alepes melanoptera|DBMF-M42
Alepes melanoptera|DBMF-M524
Alepes melanoptera|DBMF-M53
Alepes melanoptera|DBMF-M523
Alepes melanoptera|DBMF-M708
Alepes melanoptera|DBMF-M712
Alepes melanoptera|DBMF-M711
Alepes melanoptera|DBMF-M16
Alepes vari|DBMF-M635
Alepes vari|DBMF-M2
Alepes vari|DBMF-M639
Alepes vari|DBMF-M452
Alepes vari|DBMF-M577
Alepes vari|DBMF-M283
Alepes vari|DBMF-M282
Alepes vari|DBMF-M284
Alepes vari|DBMF-M453
Alepes vari|DBMF-M451
Alepes vari|DBMF-M454
Alepes vari|DBMF-M286
Alepes vari|DBMF-M638
Gnathanodon speciosus|DBMF-M511
Gnathanodon speciosus|DBMF-M510
Gnathanodon speciosus|DBMF-M508
Gnathanodon speciosus|DBMF-M509
Caranx ignobilis|DBMF-M704
Caranx ignobilis|DBMF-M565
Caranx ignobilis|DBMF-M566
Caranx ignobilis|DBMF-M564
Caranx ignobilis|DBMF-M563
Caranx ignobilis|DBMF-M705
Caranx sexfasciatus|DBMF-M30
Caranx sexfasciatus|DBMF-M393
Caranx sexfasciatus|DBMF-M391
Caranx sexfasciatus|DBMF-M21
Caranx sexfasciatus|DBMF-M632
Caranx sexfasciatus|DBMF-M633
Caranx sexfasciatus|DBMF-M5
Caranx sexfasciatus|DBMF-M394
Caranx tille|DBMF-M773
Caranx tille|DBMF-M707
Caranx tille|DBMF-M706
Caranx tille|DBMF-M634
Caranx tille|DBMF-M775
Caranx tille|DBMF-M774
Caranx tille|DBMF-M776
Caranx tille|DBMF-M772
Caranx tille|DBMF-M703
Megalaspis cordyla|DBMF-M262
Megalaspis cordyla|DBMF-M820
Megalaspis cordyla|DBMF-M264
Megalaspis cordyla|DBMF-M816
Megalaspis cordyla|DBMF-M297
Megalaspis cordyla|DBMF-M685
Megalaspis cordyla|DBMF-M684
Megalaspis cordyla|DBMF-M491
Megalaspis cordyla|DBMF-M266
Megalaspis cordyla|DBMF-M367
Megalaspis cordyla|DBMF-M298
Megalaspis cordyla|DBMF-M490
Megalaspis cordyla|DBMF-M492
Megalaspis cordyla|DBMF-M818
Megalaspis cordyla|DBMF-M446
Megalaspis cordyla|DBMF-M448
Megalaspis cordyla|DBMF-M265
Megalaspis cordyla|DBMF-M368
Megalaspis cordyla|DBMF-M299
Megalaspis cordyla|DBMF-M817
Megalaspis cordyla|DBMF-M369
Megalaspis cordyla|DBMF-M301
Megalaspis cordyla|DBMF-M157

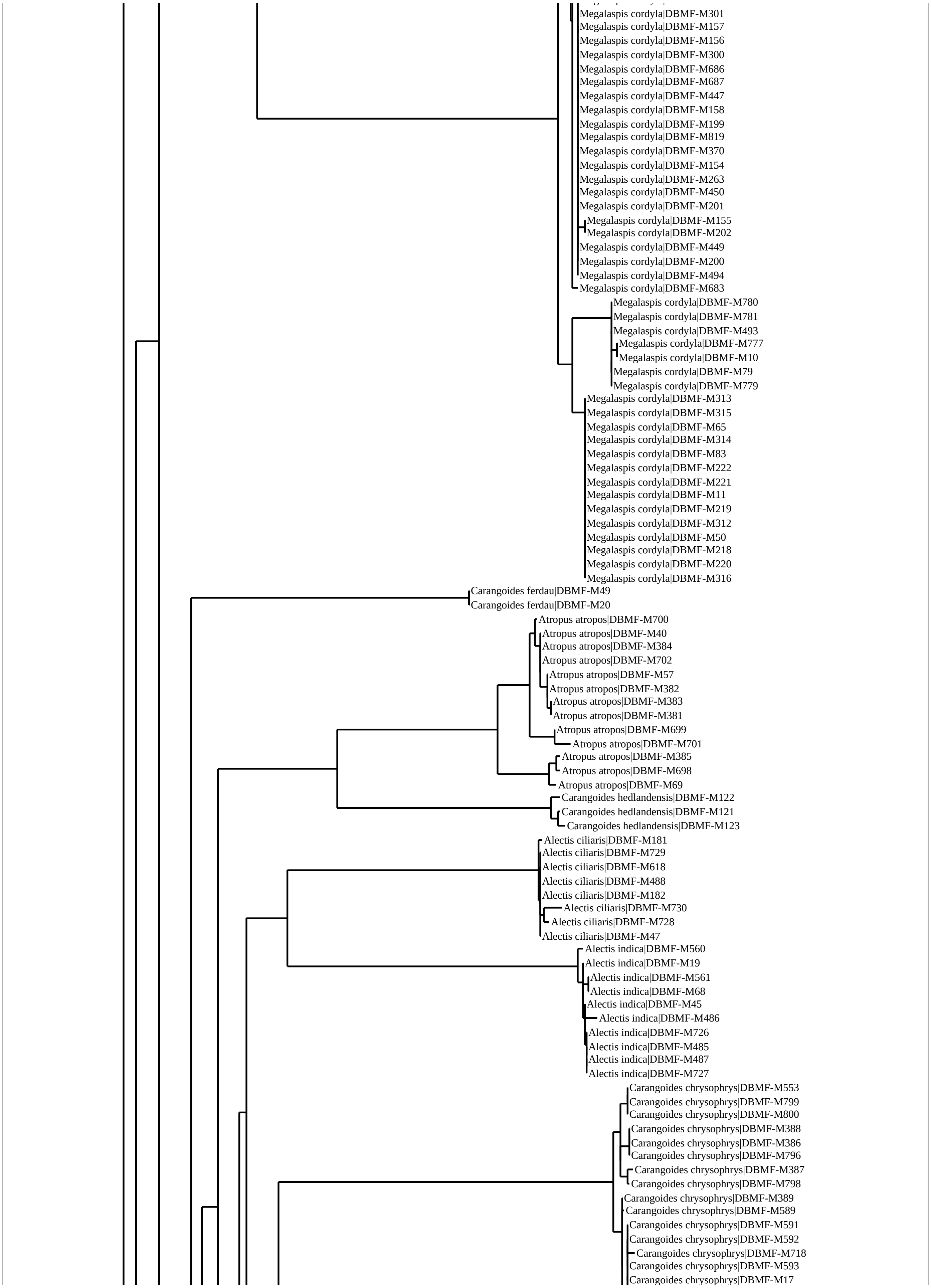
Megalaspis cordyla|DBMF-M301
Megalaspis cordyla|DBMF-M157
Megalaspis cordyla|DBMF-M156
Megalaspis cordyla|DBMF-M300
Megalaspis cordyla|DBMF-M686
Megalaspis cordyla|DBMF-M687
Megalaspis cordyla|DBMF-M447
Megalaspis cordyla|DBMF-M158
Megalaspis cordyla|DBMF-M199
Megalaspis cordyla|DBMF-M819
Megalaspis cordyla|DBMF-M370
Megalaspis cordyla|DBMF-M154
Megalaspis cordyla|DBMF-M263
Megalaspis cordyla|DBMF-M450
Megalaspis cordyla|DBMF-M201
Megalaspis cordyla|DBMF-M155
Megalaspis cordyla|DBMF-M202
Megalaspis cordyla|DBMF-M449
Megalaspis cordyla|DBMF-M200
Megalaspis cordyla|DBMF-M494
Megalaspis cordyla|DBMF-M683
Megalaspis cordyla|DBMF-M780
Megalaspis cordyla|DBMF-M781
Megalaspis cordyla|DBMF-M493
Megalaspis cordyla|DBMF-M777
Megalaspis cordyla|DBMF-M10
Megalaspis cordyla|DBMF-M79
Megalaspis cordyla|DBMF-M779
Megalaspis cordyla|DBMF-M313
Megalaspis cordyla|DBMF-M315
Megalaspis cordyla|DBMF-M65
Megalaspis cordyla|DBMF-M314
Megalaspis cordyla|DBMF-M83
Megalaspis cordyla|DBMF-M222
Megalaspis cordyla|DBMF-M221
Megalaspis cordyla|DBMF-M11
Megalaspis cordyla|DBMF-M219
Megalaspis cordyla|DBMF-M312
Megalaspis cordyla|DBMF-M50
Megalaspis cordyla|DBMF-M218
Megalaspis cordyla|DBMF-M220
Megalaspis cordyla|DBMF-M316
Carangoides ferdau|DBMF-M49
Carangoides ferdau|DBMF-M20
Atropus atropos|DBMF-M700
Atropus atropos|DBMF-M40
Atropus atropos|DBMF-M384
Atropus atropos|DBMF-M702
Atropus atropos|DBMF-M57
Atropus atropos|DBMF-M382
Atropus atropos|DBMF-M383
Atropus atropos|DBMF-M381
Atropus atropos|DBMF-M699
Atropus atropos|DBMF-M701
Atropus atropos|DBMF-M385
Atropus atropos|DBMF-M698
Atropus atropos|DBMF-M69
Carangoides hedlandensis|DBMF-M122
Carangoides hedlandensis|DBMF-M121
Carangoides hedlandensis|DBMF-M123
Alectis ciliaris|DBMF-M181
Alectis ciliaris|DBMF-M729
Alectis ciliaris|DBMF-M618
Alectis ciliaris|DBMF-M488
Alectis ciliaris|DBMF-M182
Alectis ciliaris|DBMF-M730
Alectis ciliaris|DBMF-M728
Alectis ciliaris|DBMF-M47
Alectis indica|DBMF-M560
Alectis indica|DBMF-M19
Alectis indica|DBMF-M561
Alectis indica|DBMF-M68
Alectis indica|DBMF-M45
Alectis indica|DBMF-M486
Alectis indica|DBMF-M726
Alectis indica|DBMF-M485
Alectis indica|DBMF-M487
Alectis indica|DBMF-M727
Carangoides chrysophrys|DBMF-M553
Carangoides chrysophrys|DBMF-M799
Carangoides chrysophrys|DBMF-M800
Carangoides chrysophrys|DBMF-M388
Carangoides chrysophrys|DBMF-M386
Carangoides chrysophrys|DBMF-M796
Carangoides chrysophrys|DBMF-M387
Carangoides chrysophrys|DBMF-M798
Carangoides chrysophrys|DBMF-M389
Carangoides chrysophrys|DBMF-M589
Carangoides chrysophrys|DBMF-M591
Carangoides chrysophrys|DBMF-M592
Carangoides chrysophrys|DBMF-M718
Carangoides chrysophrys|DBMF-M593
Carangoides chrysophrys|DBMF-M17

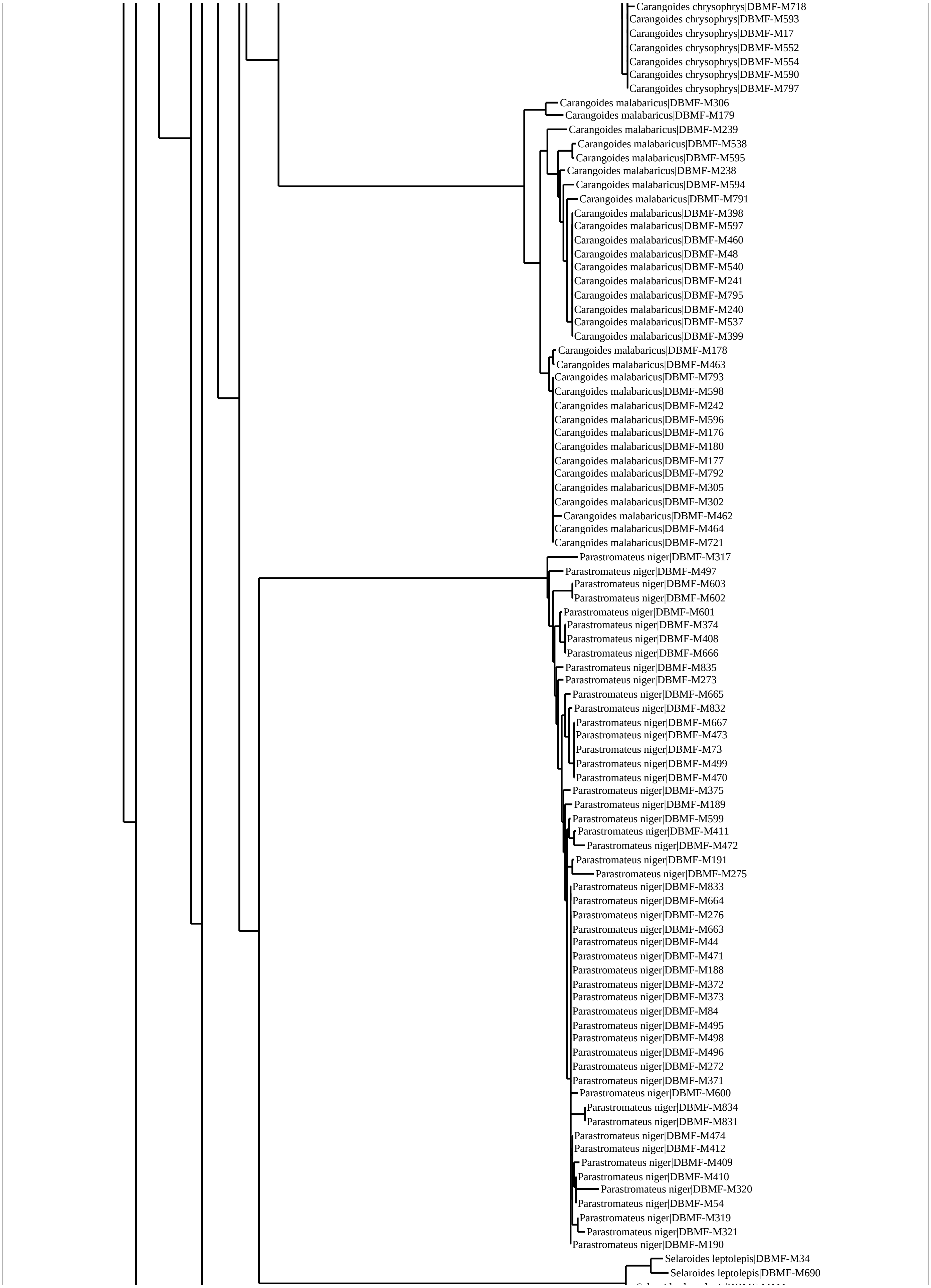
Carangoides chrysophrys|DBMF-M718
Carangoides chrysophrys|DBMF-M593
Carangoides chrysophrys|DBMF-M17
Carangoides chrysophrys|DBMF-M552
Carangoides chrysophrys|DBMF-M554
Carangoides chrysophrys|DBMF-M590
Carangoides chrysophrys|DBMF-M797
Carangoides malabaricus|DBMF-M306
Carangoides malabaricus|DBMF-M179
Carangoides malabaricus|DBMF-M239
Carangoides malabaricus|DBMF-M538
Carangoides malabaricus|DBMF-M595
Carangoides malabaricus|DBMF-M238
Carangoides malabaricus|DBMF-M594
Carangoides malabaricus|DBMF-M791
Carangoides malabaricus|DBMF-M398
Carangoides malabaricus|DBMF-M597
Carangoides malabaricus|DBMF-M460
Carangoides malabaricus|DBMF-M48
Carangoides malabaricus|DBMF-M540
Carangoides malabaricus|DBMF-M241
Carangoides malabaricus|DBMF-M795
Carangoides malabaricus|DBMF-M240
Carangoides malabaricus|DBMF-M537
Carangoides malabaricus|DBMF-M399
Carangoides malabaricus|DBMF-M178
Carangoides malabaricus|DBMF-M463
Carangoides malabaricus|DBMF-M793
Carangoides malabaricus|DBMF-M598
Carangoides malabaricus|DBMF-M242
Carangoides malabaricus|DBMF-M596
Carangoides malabaricus|DBMF-M176
Carangoides malabaricus|DBMF-M180
Carangoides malabaricus|DBMF-M177
Carangoides malabaricus|DBMF-M792
Carangoides malabaricus|DBMF-M305
Carangoides malabaricus|DBMF-M302
Carangoides malabaricus|DBMF-M462
Carangoides malabaricus|DBMF-M464
Carangoides malabaricus|DBMF-M721
Parastromateus niger|DBMF-M317
Parastromateus niger|DBMF-M497
Parastromateus niger|DBMF-M603
Parastromateus niger|DBMF-M602
Parastromateus niger|DBMF-M601
Parastromateus niger|DBMF-M374
Parastromateus niger|DBMF-M408
Parastromateus niger|DBMF-M666
Parastromateus niger|DBMF-M835
Parastromateus niger|DBMF-M273
Parastromateus niger|DBMF-M665
Parastromateus niger|DBMF-M832
Parastromateus niger|DBMF-M667
Parastromateus niger|DBMF-M473
Parastromateus niger|DBMF-M73
Parastromateus niger|DBMF-M499
Parastromateus niger|DBMF-M470
Parastromateus niger|DBMF-M375
Parastromateus niger|DBMF-M189
Parastromateus niger|DBMF-M599
Parastromateus niger|DBMF-M411
Parastromateus niger|DBMF-M472
Parastromateus niger|DBMF-M191
Parastromateus niger|DBMF-M275
Parastromateus niger|DBMF-M833
Parastromateus niger|DBMF-M664
Parastromateus niger|DBMF-M276
Parastromateus niger|DBMF-M663
Parastromateus niger|DBMF-M44
Parastromateus niger|DBMF-M471
Parastromateus niger|DBMF-M188
Parastromateus niger|DBMF-M372
Parastromateus niger|DBMF-M373
Parastromateus niger|DBMF-M84
Parastromateus niger|DBMF-M495
Parastromateus niger|DBMF-M498
Parastromateus niger|DBMF-M496
Parastromateus niger|DBMF-M272
Parastromateus niger|DBMF-M371
Parastromateus niger|DBMF-M600
Parastromateus niger|DBMF-M834
Parastromateus niger|DBMF-M831
Parastromateus niger|DBMF-M474
Parastromateus niger|DBMF-M412
Parastromateus niger|DBMF-M409
Parastromateus niger|DBMF-M410
Parastromateus niger|DBMF-M320
Parastromateus niger|DBMF-M54
Parastromateus niger|DBMF-M319
Parastromateus niger|DBMF-M321
Parastromateus niger|DBMF-M190
Selaroides leptolepis|DBMF-M34
Selaroides leptolepis|DBMF-M690

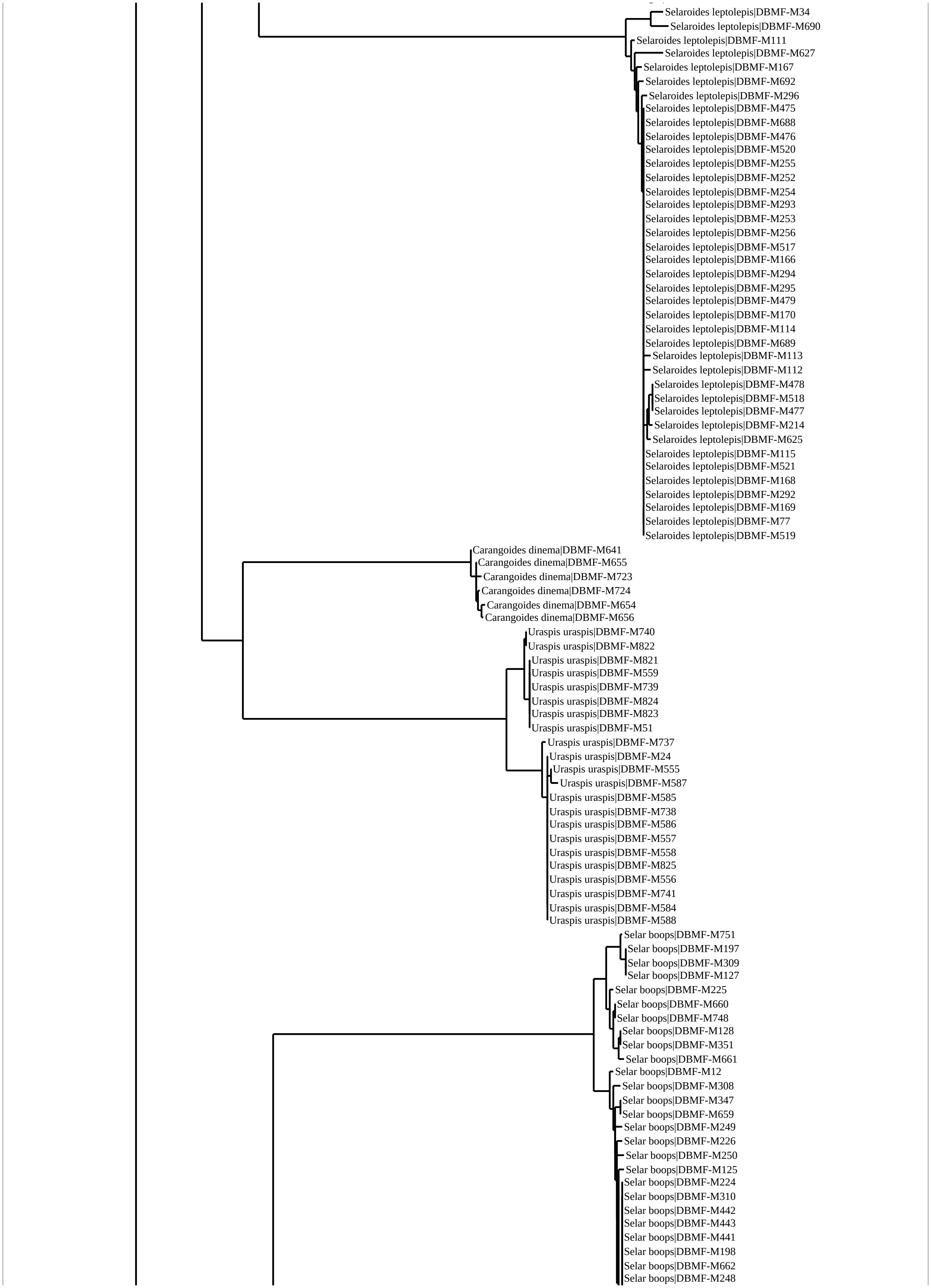
Selaroides leptolepis|DBMF-M34
Selaroides leptolepis|DBMF-M690
Selaroides leptolepis|DBMF-M111
Selaroides leptolepis|DBMF-M627
Selaroides leptolepis|DBMF-M167
Selaroides leptolepis|DBMF-M692
Selaroides leptolepis|DBMF-M296
Selaroides leptolepis|DBMF-M475
Selaroides leptolepis|DBMF-M688
Selaroides leptolepis|DBMF-M476
Selaroides leptolepis|DBMF-M520
Selaroides leptolepis|DBMF-M255
Selaroides leptolepis|DBMF-M252
Selaroides leptolepis|DBMF-M254
Selaroides leptolepis|DBMF-M293
Selaroides leptolepis|DBMF-M253
Selaroides leptolepis|DBMF-M256
Selaroides leptolepis|DBMF-M517
Selaroides leptolepis|DBMF-M166
Selaroides leptolepis|DBMF-M294
Selaroides leptolepis|DBMF-M295
Selaroides leptolepis|DBMF-M479
Selaroides leptolepis|DBMF-M170
Selaroides leptolepis|DBMF-M114
Selaroides leptolepis|DBMF-M689
Selaroides leptolepis|DBMF-M113
Selaroides leptolepis|DBMF-M112
Selaroides leptolepis|DBMF-M478
Selaroides leptolepis|DBMF-M518
Selaroides leptolepis|DBMF-M477
Selaroides leptolepis|DBMF-M214
Selaroides leptolepis|DBMF-M625
Selaroides leptolepis|DBMF-M115
Selaroides leptolepis|DBMF-M521
Selaroides leptolepis|DBMF-M168
Selaroides leptolepis|DBMF-M292
Selaroides leptolepis|DBMF-M169
Selaroides leptolepis|DBMF-M77
Selaroides leptolepis|DBMF-M519
Carangoides dinema|DBMF-M641
Carangoides dinema|DBMF-M655
Carangoides dinema|DBMF-M723
Carangoides dinema|DBMF-M724
Carangoides dinema|DBMF-M654
Carangoides dinema|DBMF-M656
Uraspis uraspis|DBMF-M740
Uraspis uraspis|DBMF-M822
Uraspis uraspis|DBMF-M821
Uraspis uraspis|DBMF-M559
Uraspis uraspis|DBMF-M739
Uraspis uraspis|DBMF-M824
Uraspis uraspis|DBMF-M823
Uraspis uraspis|DBMF-M51
Uraspis uraspis|DBMF-M737
Uraspis uraspis|DBMF-M24
Uraspis uraspis|DBMF-M555
Uraspis uraspis|DBMF-M587
Uraspis uraspis|DBMF-M585
Uraspis uraspis|DBMF-M738
Uraspis uraspis|DBMF-M586
Uraspis uraspis|DBMF-M557
Uraspis uraspis|DBMF-M558
Uraspis uraspis|DBMF-M825
Uraspis uraspis|DBMF-M556
Uraspis uraspis|DBMF-M741
Uraspis uraspis|DBMF-M584
Uraspis uraspis|DBMF-M588
Selar boops|DBMF-M751
Selar boops|DBMF-M197
Selar boops|DBMF-M309
Selar boops|DBMF-M127
Selar boops|DBMF-M225
Selar boops|DBMF-M660
Selar boops|DBMF-M748
Selar boops|DBMF-M128
Selar boops|DBMF-M351
Selar boops|DBMF-M661
Selar boops|DBMF-M12
Selar boops|DBMF-M308
Selar boops|DBMF-M347
Selar boops|DBMF-M659
Selar boops|DBMF-M249
Selar boops|DBMF-M226
Selar boops|DBMF-M250
Selar boops|DBMF-M125
Selar boops|DBMF-M224
Selar boops|DBMF-M310
Selar boops|DBMF-M442
Selar boops|DBMF-M443
Selar boops|DBMF-M441
Selar boops|DBMF-M198
Selar boops|DBMF-M662
Selar boops|DBMF-M248

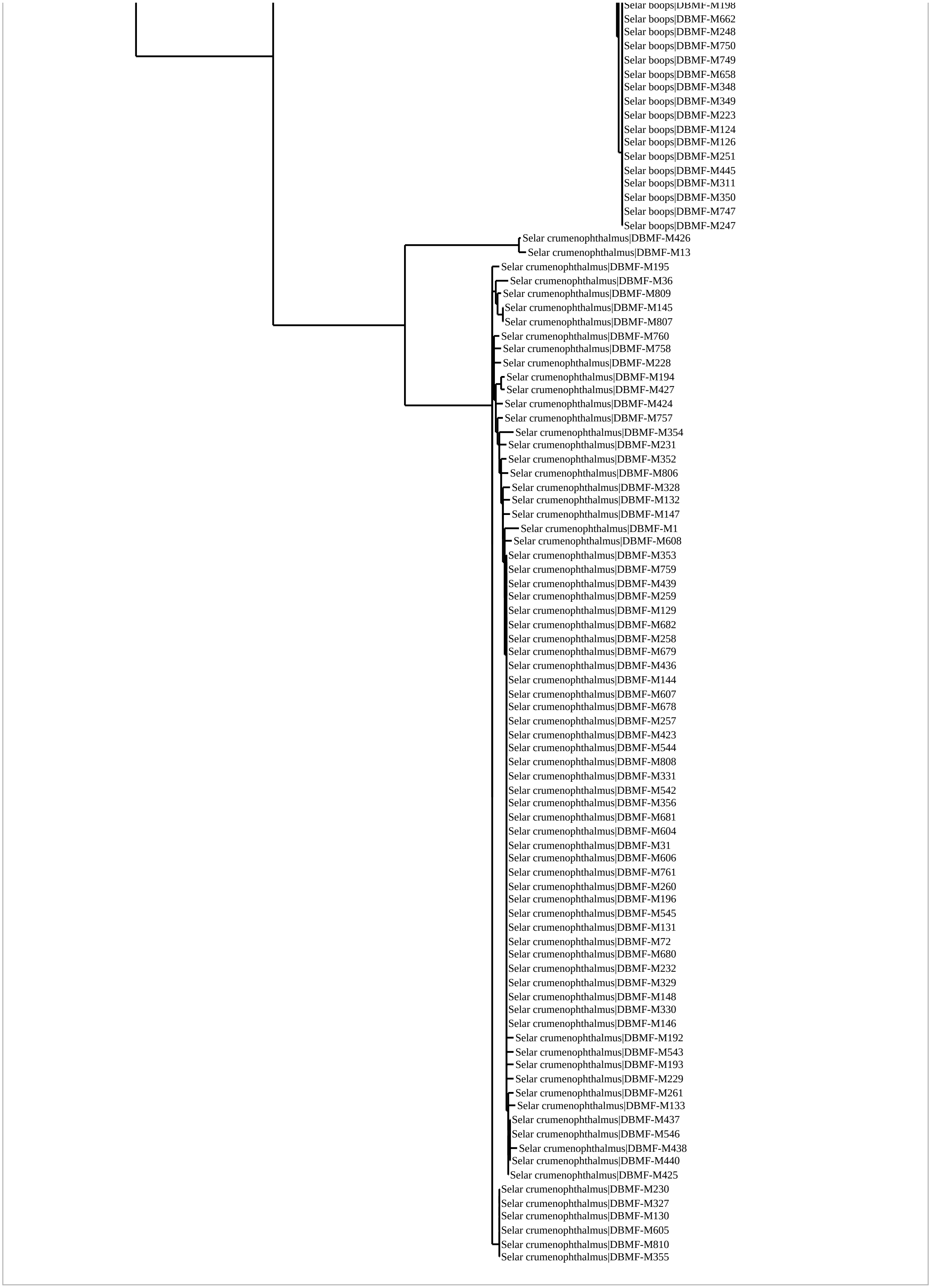
Selar boops|DBMF-M198
Selar boops|DBMF-M662
Selar boops|DBMF-M248
Selar boops|DBMF-M750
Selar boops|DBMF-M749
Selar boops|DBMF-M658
Selar boops|DBMF-M348
Selar boops|DBMF-M349
Selar boops|DBMF-M223
Selar boops|DBMF-M124
Selar boops|DBMF-M126
Selar boops|DBMF-M251
Selar boops|DBMF-M445
Selar boops|DBMF-M311
Selar boops|DBMF-M350
Selar boops|DBMF-M747
Selar boops|DBMF-M247
Selar crumenophthalmus|DBMF-M426
Selar crumenophthalmus|DBMF-M13
Selar crumenophthalmus|DBMF-M195
Selar crumenophthalmus|DBMF-M36
Selar crumenophthalmus|DBMF-M809
Selar crumenophthalmus|DBMF-M145
Selar crumenophthalmus|DBMF-M807
Selar crumenophthalmus|DBMF-M760
Selar crumenophthalmus|DBMF-M758
Selar crumenophthalmus|DBMF-M228
Selar crumenophthalmus|DBMF-M194
Selar crumenophthalmus|DBMF-M427
Selar crumenophthalmus|DBMF-M424
Selar crumenophthalmus|DBMF-M757
Selar crumenophthalmus|DBMF-M354
Selar crumenophthalmus|DBMF-M231
Selar crumenophthalmus|DBMF-M352
Selar crumenophthalmus|DBMF-M806
Selar crumenophthalmus|DBMF-M328
Selar crumenophthalmus|DBMF-M132
Selar crumenophthalmus|DBMF-M147
Selar crumenophthalmus|DBMF-M1
Selar crumenophthalmus|DBMF-M608
Selar crumenophthalmus|DBMF-M353
Selar crumenophthalmus|DBMF-M759
Selar crumenophthalmus|DBMF-M439
Selar crumenophthalmus|DBMF-M259
Selar crumenophthalmus|DBMF-M129
Selar crumenophthalmus|DBMF-M682
Selar crumenophthalmus|DBMF-M258
Selar crumenophthalmus|DBMF-M679
Selar crumenophthalmus|DBMF-M436
Selar crumenophthalmus|DBMF-M144
Selar crumenophthalmus|DBMF-M607
Selar crumenophthalmus|DBMF-M678
Selar crumenophthalmus|DBMF-M257
Selar crumenophthalmus|DBMF-M423
Selar crumenophthalmus|DBMF-M544
Selar crumenophthalmus|DBMF-M808
Selar crumenophthalmus|DBMF-M331
Selar crumenophthalmus|DBMF-M542
Selar crumenophthalmus|DBMF-M356
Selar crumenophthalmus|DBMF-M681
Selar crumenophthalmus|DBMF-M604
Selar crumenophthalmus|DBMF-M31
Selar crumenophthalmus|DBMF-M606
Selar crumenophthalmus|DBMF-M761
Selar crumenophthalmus|DBMF-M260
Selar crumenophthalmus|DBMF-M196
Selar crumenophthalmus|DBMF-M545
Selar crumenophthalmus|DBMF-M131
Selar crumenophthalmus|DBMF-M72
Selar crumenophthalmus|DBMF-M680
Selar crumenophthalmus|DBMF-M232
Selar crumenophthalmus|DBMF-M329
Selar crumenophthalmus|DBMF-M148
Selar crumenophthalmus|DBMF-M330
Selar crumenophthalmus|DBMF-M146
Selar crumenophthalmus|DBMF-M192
Selar crumenophthalmus|DBMF-M543
Selar crumenophthalmus|DBMF-M193
Selar crumenophthalmus|DBMF-M229
Selar crumenophthalmus|DBMF-M261
Selar crumenophthalmus|DBMF-M133
Selar crumenophthalmus|DBMF-M437
Selar crumenophthalmus|DBMF-M546
Selar crumenophthalmus|DBMF-M438
Selar crumenophthalmus|DBMF-M440
Selar crumenophthalmus|DBMF-M425
Selar crumenophthalmus|DBMF-M230
Selar crumenophthalmus|DBMF-M327
Selar crumenophthalmus|DBMF-M130
Selar crumenophthalmus|DBMF-M605
Selar crumenophthalmus|DBMF-M810
Selar crumenophthalmus|DBMF-M355
