# Supplementary material for: DNA Barcoding Reveals Cryptic Diversity within Commercially Exploited Indo-Malay Carangidae (Teleosteii: Perciformes)
Source: PLoS One. 2012 Nov 29;7(11):e49623. doi: 10.1371/journal.pone.0049623 (PMC3510217; doi:10.1371/journal.pone.0049623)
Supplement: Figure S2 — Phylogenetic tree from Maximum-likelihood analysis. Numbers above the branches represent bootstrap support based on 1000 replicates. (PDF) [file pone.0049623.s002.pdf]

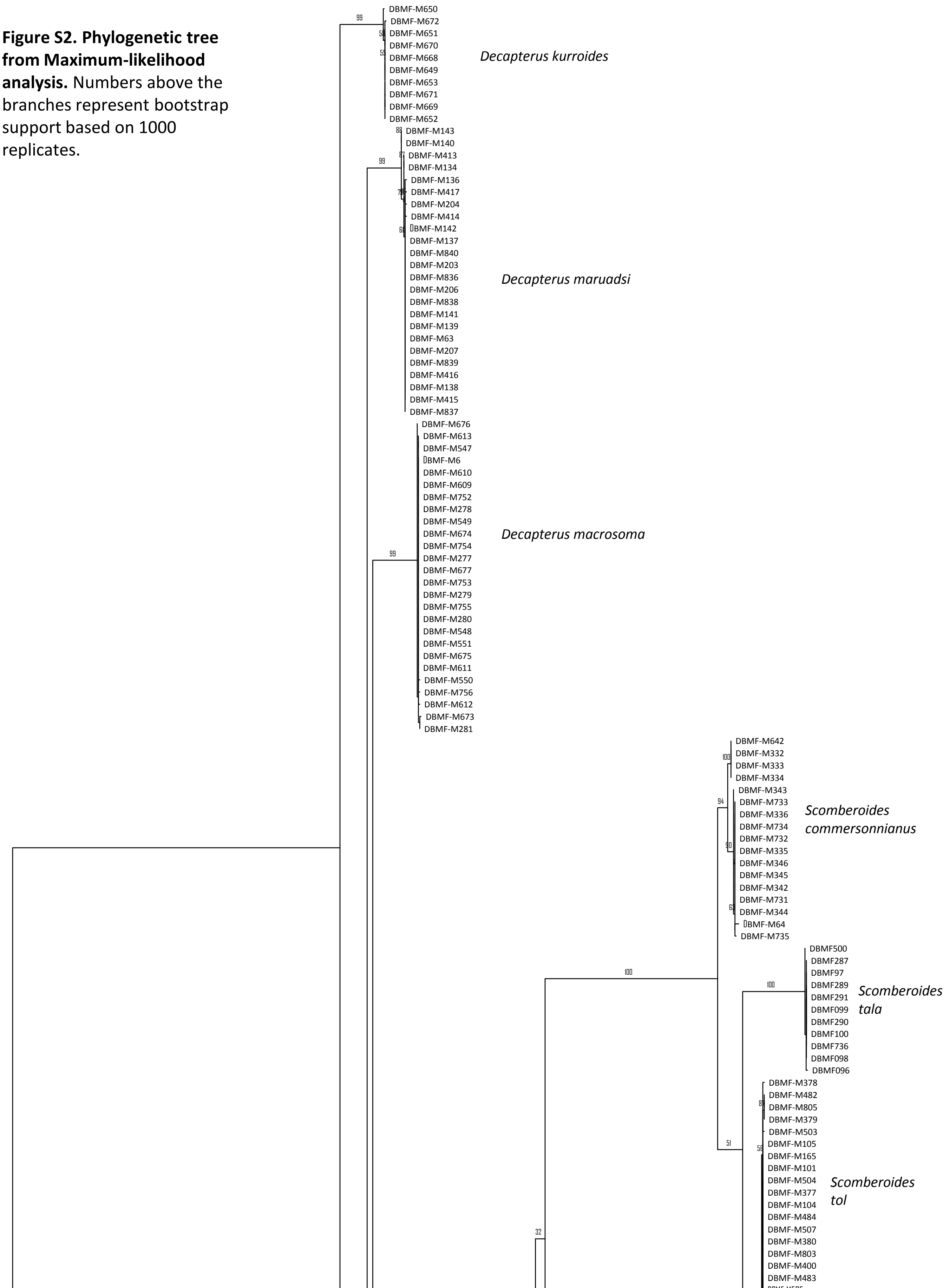


**Figure S2. Phylogenetic tree from Maximum-likelihood analysis.** Numbers above the branches represent bootstrap support based on 1000 replicates.

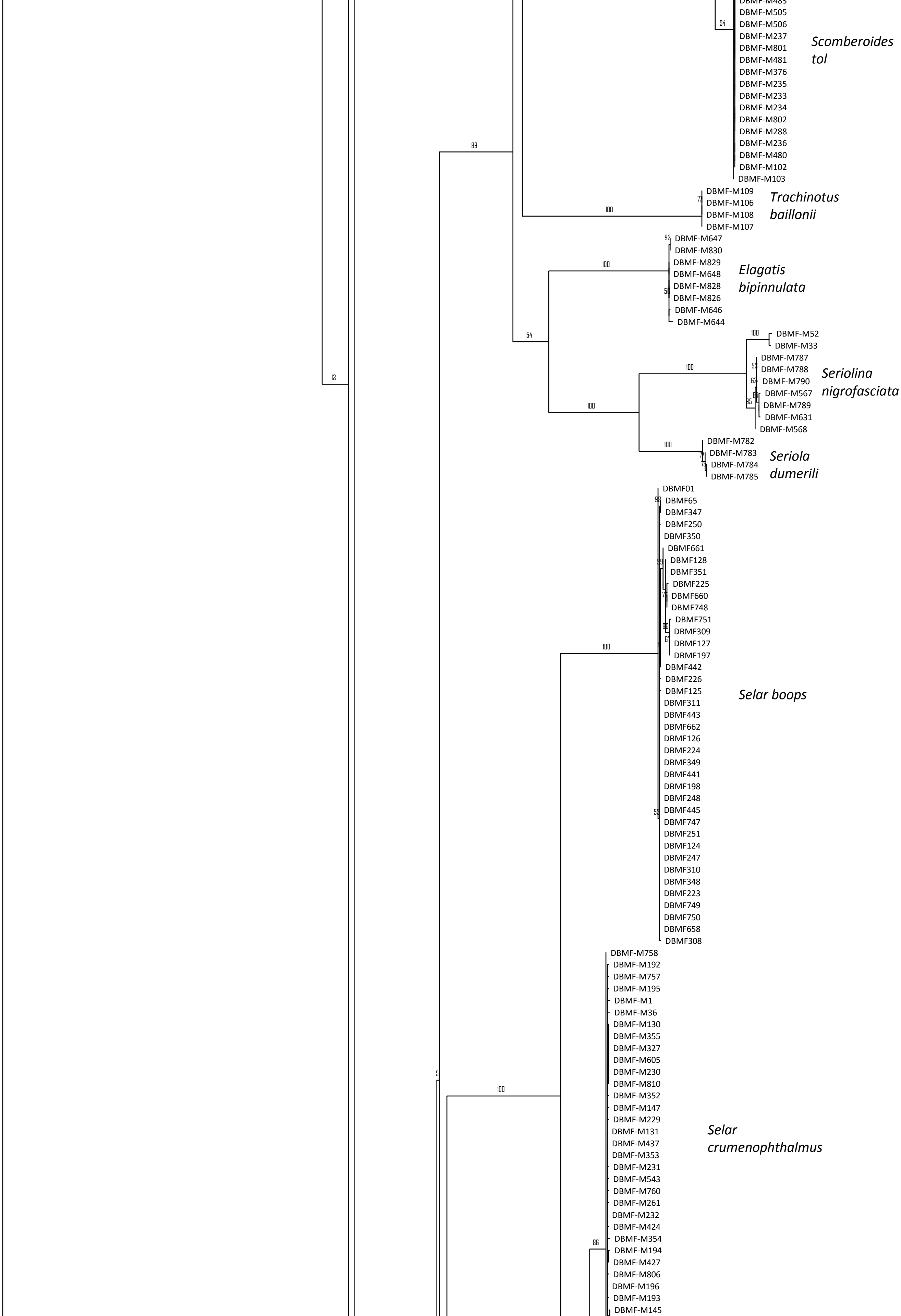

DBMF-M505
DBMF-M506
DBMF-M237
DBMF-M801
DBMF-M481
DBMF-M376
DBMF-M235
DBMF-M233
DBMF-M234
DBMF-M802
DBMF-M288
DBMF-M236
DBMF-M480
DBMF-M102
DBMF-M103
Scomberoides tol
DBMF-M109
DBMF-M106
DBMF-M108
DBMF-M107
Trachinotus baillonii
DBMF-M647
DBMF-M830
DBMF-M829
DBMF-M648
DBMF-M828
DBMF-M826
DBMF-M646
DBMF-M644
Elagatis bipinnulata
DBMF-M52
DBMF-M33
DBMF-M787
DBMF-M788
DBMF-M790
DBMF-M567
DBMF-M789
DBMF-M631
DBMF-M568
Seriolina nigrofasciata
DBMF-M782
DBMF-M783
DBMF-M784
DBMF-M785
Seriola dumerili
DBMF01
DBMF65
DBMF347
DBMF250
DBMF350
DBMF661
DBMF128
DBMF351
DBMF225
DBMF660
DBMF748
DBMF751
DBMF309
DBMF127
DBMF197
DBMF442
DBMF226
DBMF125
DBMF311
DBMF443
DBMF662
DBMF126
DBMF224
DBMF349
DBMF441
DBMF198
DBMF248
DBMF445
DBMF747
DBMF251
DBMF124
DBMF247
DBMF310
DBMF348
DBMF223
DBMF749
DBMF750
DBMF658
DBMF308
Selar boops
DBMF-M758
DBMF-M192
DBMF-M757
DBMF-M195
DBMF-M1
DBMF-M36
DBMF-M130
DBMF-M355
DBMF-M327
DBMF-M605
DBMF-M230
DBMF-M810
DBMF-M352
DBMF-M147
DBMF-M229
DBMF-M131
DBMF-M437
DBMF-M353
DBMF-M231
DBMF-M543
DBMF-M760
DBMF-M261
DBMF-M232
DBMF-M424
DBMF-M354
DBMF-M194
DBMF-M427
DBMF-M806
DBMF-M196
DBMF-M193
DBMF-M145
Selar crumenophthalmus

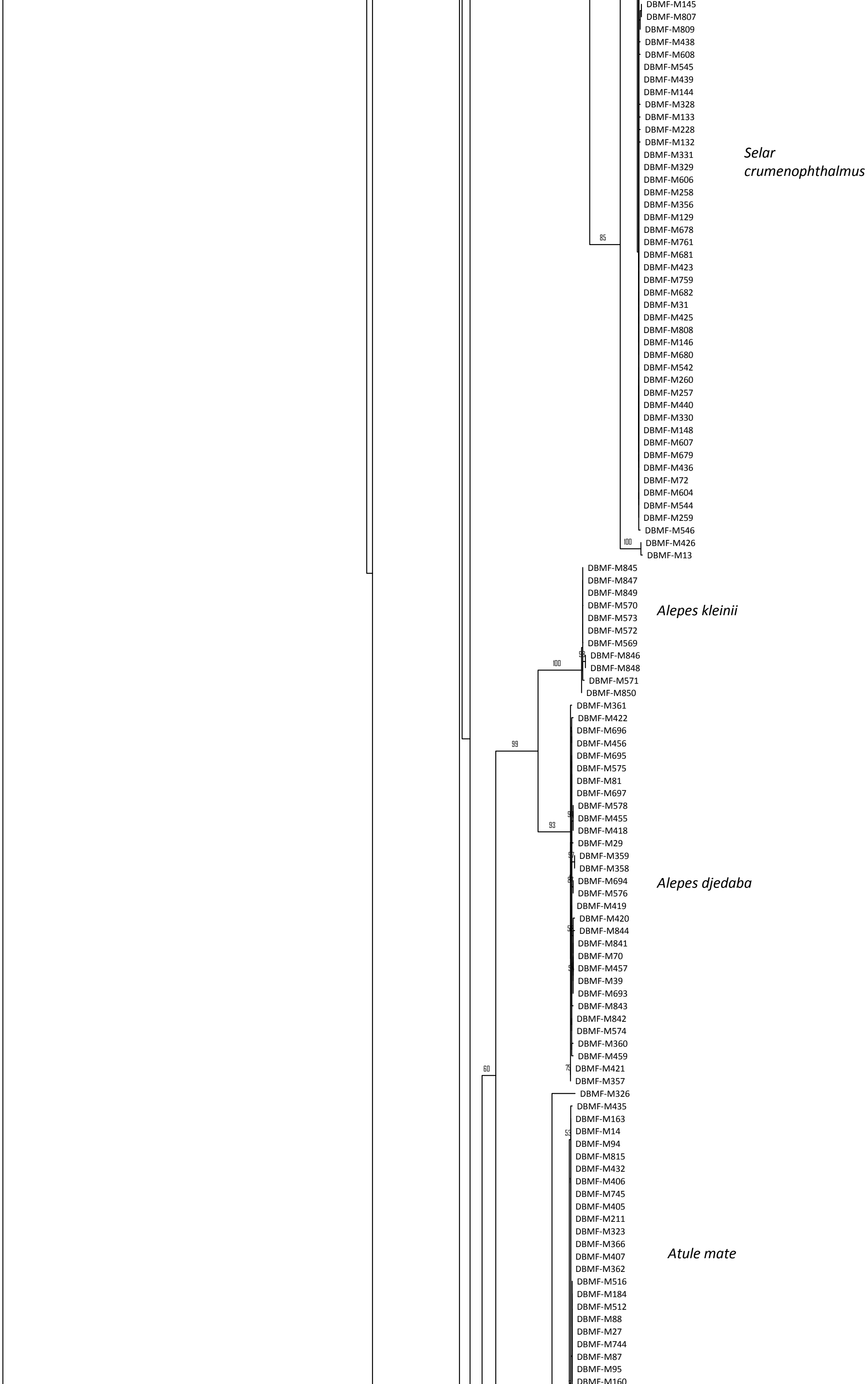
DBMF-M145
DBMF-M807
DBMF-M809
DBMF-M438
DBMF-M608
DBMF-M545
DBMF-M439
DBMF-M144
DBMF-M328
DBMF-M133
DBMF-M228
DBMF-M132
DBMF-M331
DBMF-M329
DBMF-M606
DBMF-M258
DBMF-M356
DBMF-M129
DBMF-M678
DBMF-M761
DBMF-M681
DBMF-M423
DBMF-M759
DBMF-M682
DBMF-M31
DBMF-M425
DBMF-M808
DBMF-M146
DBMF-M680
DBMF-M542
DBMF-M260
DBMF-M257
DBMF-M440
DBMF-M330
DBMF-M148
DBMF-M607
DBMF-M679
DBMF-M436
DBMF-M72
DBMF-M604
DBMF-M544
DBMF-M259
DBMF-M546
DBMF-M426
DBMF-M13
Selar crumenophthalmus
85
100
DBMF-M845
DBMF-M847
DBMF-M849
DBMF-M570
DBMF-M573
DBMF-M572
DBMF-M569
DBMF-M846
DBMF-M848
DBMF-M571
DBMF-M850
Alepes kleinii
100
99
DBMF-M361
DBMF-M422
DBMF-M696
DBMF-M456
DBMF-M695
DBMF-M575
DBMF-M81
DBMF-M697
DBMF-M578
DBMF-M455
DBMF-M418
DBMF-M29
DBMF-M359
DBMF-M358
DBMF-M694
DBMF-M576
DBMF-M419
DBMF-M420
DBMF-M844
DBMF-M841
DBMF-M70
DBMF-M457
DBMF-M39
DBMF-M693
DBMF-M843
DBMF-M842
DBMF-M574
DBMF-M360
DBMF-M459
DBMF-M421
DBMF-M357
Alepes djedaba
93
60
DBMF-M326
DBMF-M435
DBMF-M163
DBMF-M14
DBMF-M94
DBMF-M815
DBMF-M432
DBMF-M406
DBMF-M745
DBMF-M405
DBMF-M211
DBMF-M323
DBMF-M366
DBMF-M407
DBMF-M362
DBMF-M516
DBMF-M184
DBMF-M512
DBMF-M88
DBMF-M27
DBMF-M744
DBMF-M87
DBMF-M95
DBMF-M160
Atule mate

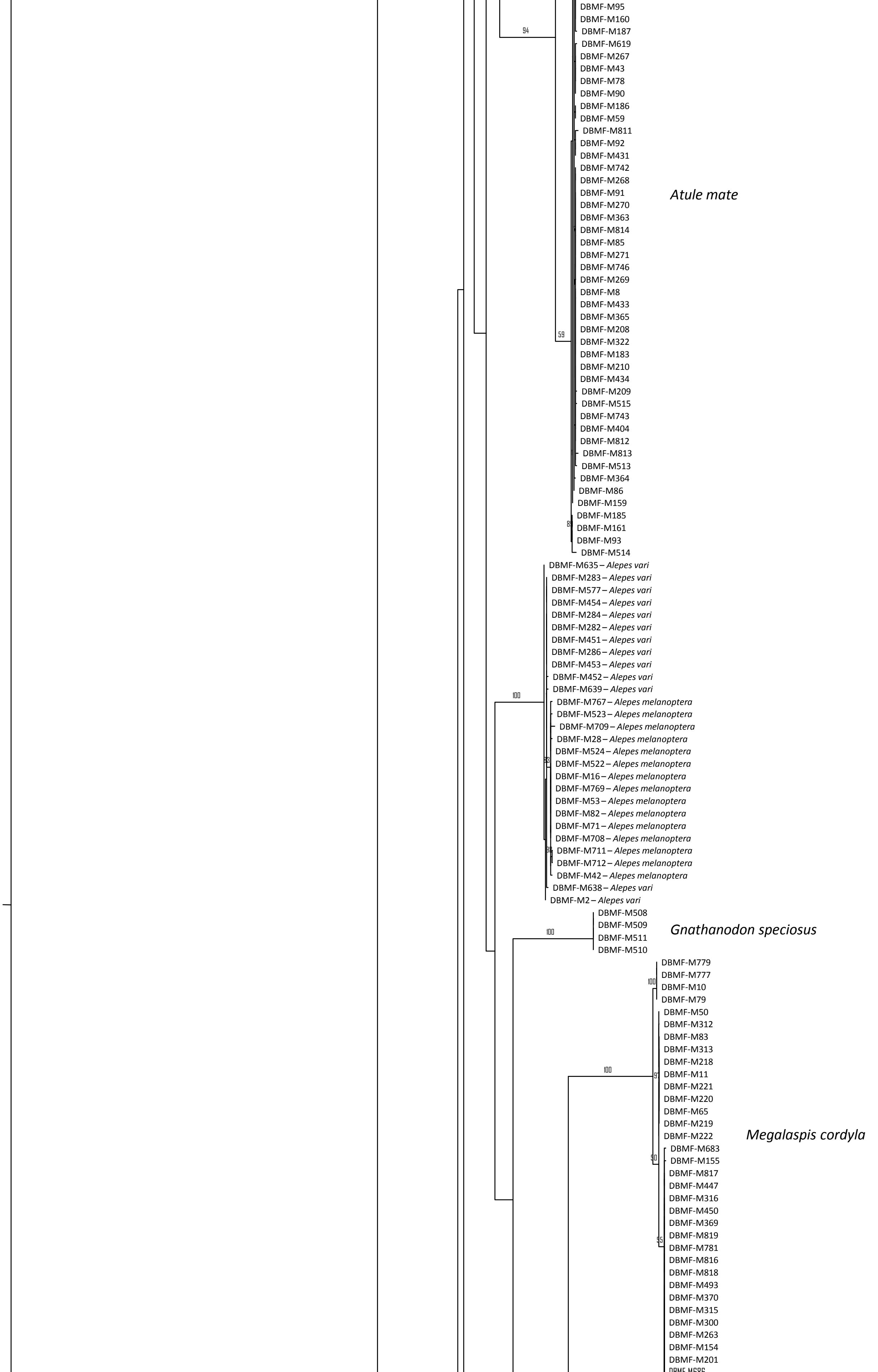
DBMF-M95
DBMF-M160
DBMF-M187
94
DBMF-M619
DBMF-M267
DBMF-M43
DBMF-M78
DBMF-M90
DBMF-M186
DBMF-M59
DBMF-M811
DBMF-M92
DBMF-M431
DBMF-M742
DBMF-M268
DBMF-M91
DBMF-M270
DBMF-M363
DBMF-M814
DBMF-M85
DBMF-M271
DBMF-M746
DBMF-M269
DBMF-M8
DBMF-M433
DBMF-M365
DBMF-M208
59
DBMF-M322
DBMF-M183
DBMF-M210
DBMF-M434
DBMF-M209
DBMF-M515
DBMF-M743
DBMF-M404
DBMF-M812
DBMF-M813
DBMF-M513
DBMF-M364
DBMF-M86
DBMF-M159
DBMF-M185
DBMF-M161
DBMF-M93
DBMF-M514
Atule mate
DBMF-M635 – Alepes vari
DBMF-M283 – Alepes vari
DBMF-M577 – Alepes vari
DBMF-M454 – Alepes vari
DBMF-M284 – Alepes vari
DBMF-M282 – Alepes vari
DBMF-M451 – Alepes vari
DBMF-M286 – Alepes vari
DBMF-M453 – Alepes vari
DBMF-M452 – Alepes vari
DBMF-M639 – Alepes vari
100
DBMF-M767 – Alepes melanoptera
DBMF-M523 – Alepes melanoptera
DBMF-M709 – Alepes melanoptera
DBMF-M28 – Alepes melanoptera
DBMF-M524 – Alepes melanoptera
DBMF-M522 – Alepes melanoptera
DBMF-M16 – Alepes melanoptera
DBMF-M769 – Alepes melanoptera
DBMF-M53 – Alepes melanoptera
DBMF-M82 – Alepes melanoptera
DBMF-M71 – Alepes melanoptera
DBMF-M708 – Alepes melanoptera
DBMF-M711 – Alepes melanoptera
DBMF-M712 – Alepes melanoptera
DBMF-M42 – Alepes melanoptera
DBMF-M638 – Alepes vari
DBMF-M2 – Alepes vari
DBMF-M508
DBMF-M509
100
DBMF-M511
DBMF-M510
Gnathanodon speciosus
DBMF-M779
DBMF-M777
100
DBMF-M10
DBMF-M79
DBMF-M50
DBMF-M312
DBMF-M83
DBMF-M313
DBMF-M218
100
DBMF-M11
DBMF-M221
DBMF-M220
DBMF-M65
DBMF-M219
DBMF-M222
Megalaspis cordyla
DBMF-M683
DBMF-M155
90
DBMF-M817
DBMF-M447
DBMF-M316
DBMF-M450
DBMF-M369
DBMF-M819
DBMF-M781
DBMF-M816
DBMF-M818
DBMF-M493
DBMF-M370
DBMF-M315
DBMF-M300
DBMF-M263
DBMF-M154
DBMF-M201

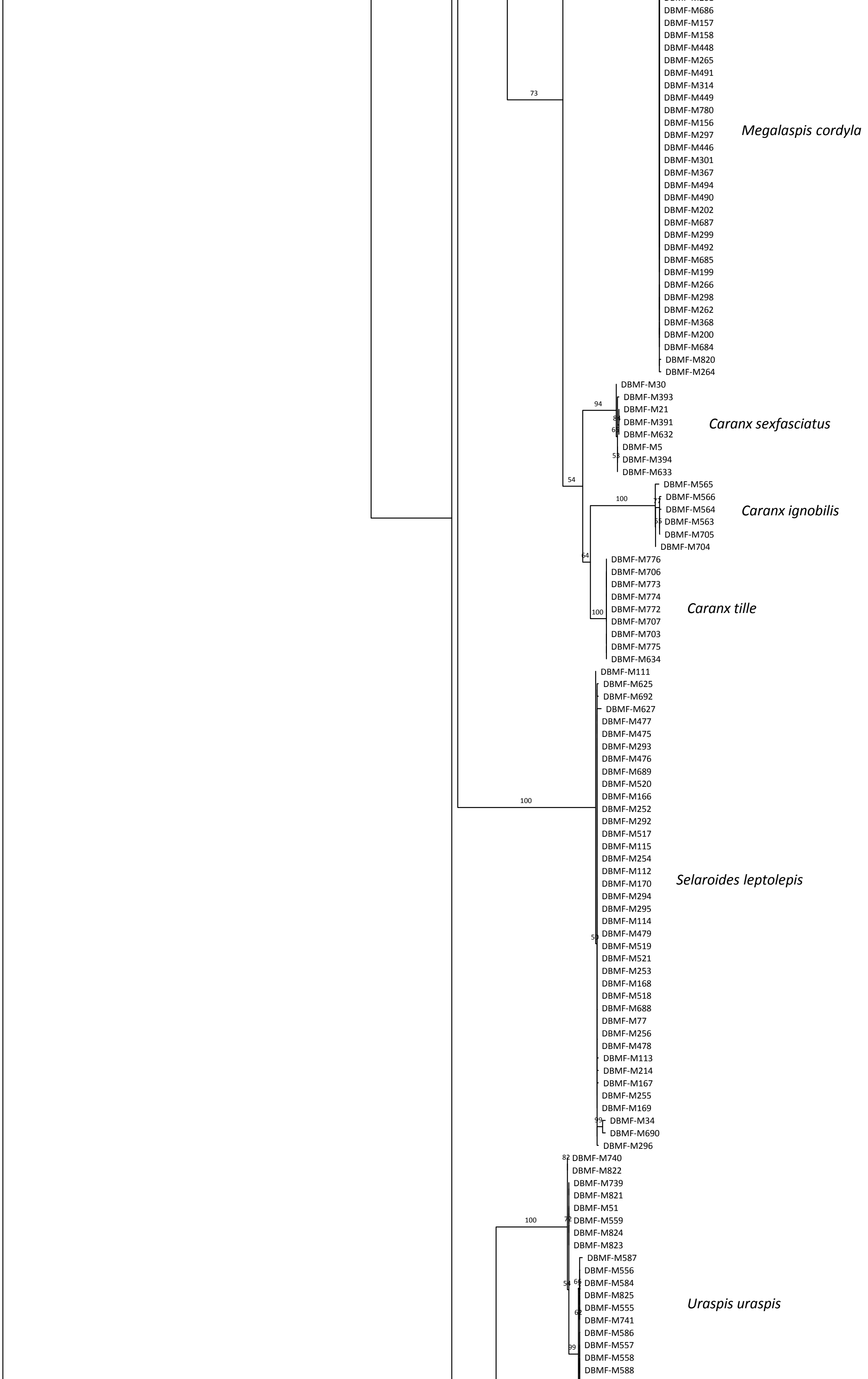
DBMF-M686
DBMF-M157
DBMF-M158
DBMF-M448
DBMF-M265
DBMF-M491
DBMF-M314
DBMF-M449
DBMF-M780
DBMF-M156
DBMF-M297
DBMF-M446
DBMF-M301
DBMF-M367
DBMF-M494
DBMF-M490
DBMF-M202
DBMF-M687
DBMF-M299
DBMF-M492
DBMF-M685
DBMF-M199
DBMF-M266
DBMF-M298
DBMF-M262
DBMF-M368
DBMF-M200
DBMF-M684
DBMF-M820
DBMF-M264
73
Megalaspis cordyla
DBMF-M30
DBMF-M393
DBMF-M21
DBMF-M391
DBMF-M632
DBMF-M5
DBMF-M394
DBMF-M633
94
54
Caranx sexfasciatus
DBMF-M565
DBMF-M566
DBMF-M564
DBMF-M563
DBMF-M705
DBMF-M704
100
Caranx ignobilis
DBMF-M776
DBMF-M706
DBMF-M773
DBMF-M774
DBMF-M772
DBMF-M707
DBMF-M703
DBMF-M775
DBMF-M634
64
100
Caranx tille
DBMF-M111
DBMF-M625
DBMF-M692
DBMF-M627
DBMF-M477
DBMF-M475
DBMF-M293
DBMF-M476
DBMF-M689
DBMF-M520
DBMF-M166
DBMF-M252
DBMF-M292
DBMF-M517
DBMF-M115
DBMF-M254
DBMF-M112
DBMF-M170
DBMF-M294
DBMF-M295
DBMF-M114
DBMF-M479
DBMF-M519
DBMF-M521
DBMF-M253
DBMF-M168
DBMF-M518
DBMF-M688
DBMF-M77
DBMF-M256
DBMF-M478
DBMF-M113
DBMF-M214
DBMF-M167
DBMF-M255
DBMF-M169
DBMF-M34
DBMF-M690
DBMF-M296
100
99
Selaroides leptolepis
DBMF-M740
DBMF-M822
DBMF-M739
DBMF-M821
DBMF-M51
DBMF-M559
DBMF-M824
DBMF-M823
DBMF-M587
DBMF-M556
DBMF-M584
DBMF-M825
DBMF-M555
DBMF-M741
DBMF-M586
DBMF-M557
DBMF-M558
DBMF-M588
100
99
Uraspis uraspis

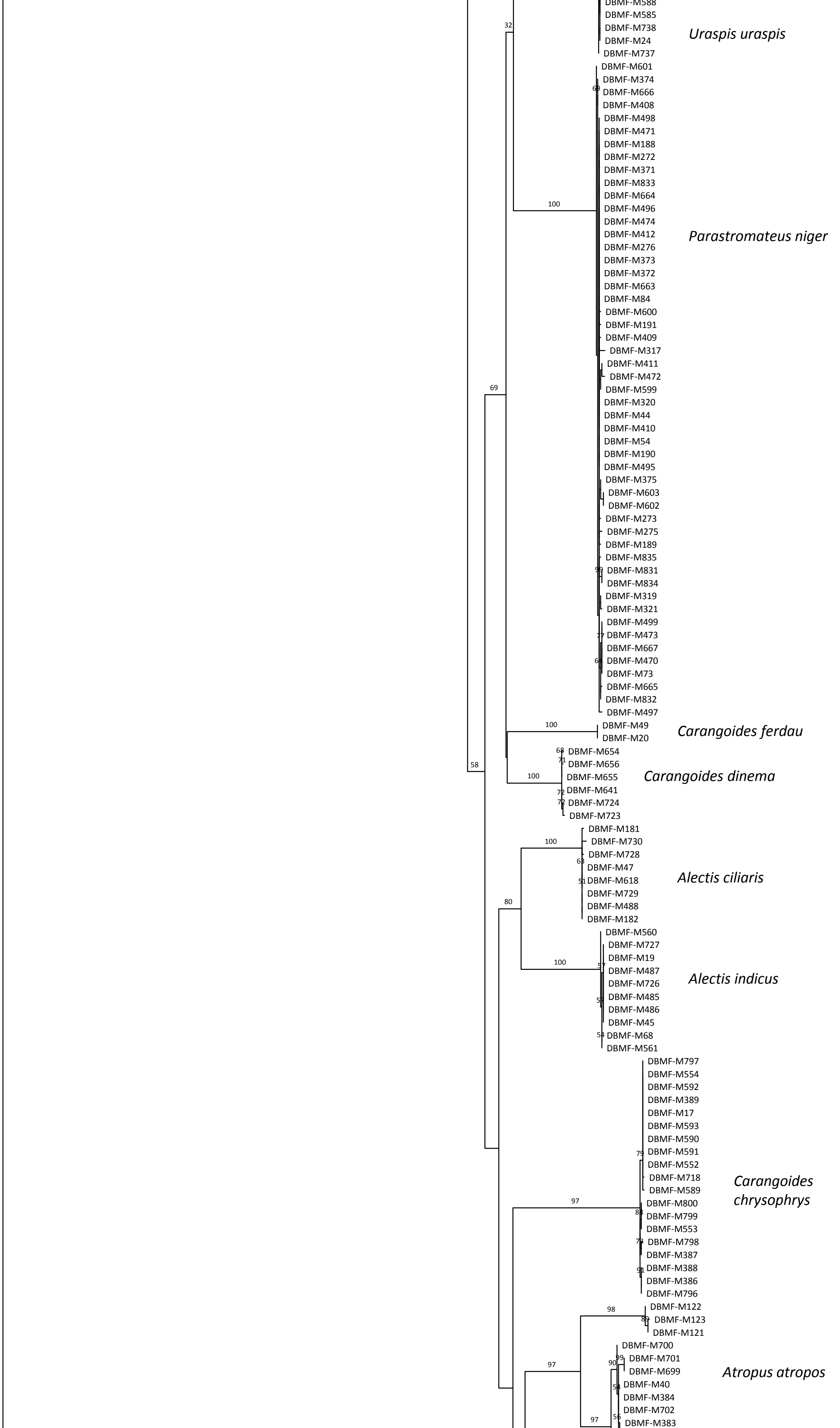
DBMF-M588
DBMF-M585
DBMF-M738
DBMF-M24
DBMF-M737
Uraspis uraspis
DBMF-M601
DBMF-M374
DBMF-M666
DBMF-M408
DBMF-M498
DBMF-M471
DBMF-M188
DBMF-M272
DBMF-M371
DBMF-M833
DBMF-M664
DBMF-M496
DBMF-M474
DBMF-M412
DBMF-M276
DBMF-M373
DBMF-M372
DBMF-M663
DBMF-M84
DBMF-M600
DBMF-M191
DBMF-M409
DBMF-M317
DBMF-M411
DBMF-M472
DBMF-M599
DBMF-M320
DBMF-M44
DBMF-M410
DBMF-M54
DBMF-M190
DBMF-M495
DBMF-M375
DBMF-M603
DBMF-M602
DBMF-M273
DBMF-M275
DBMF-M189
DBMF-M835
DBMF-M831
DBMF-M834
DBMF-M319
DBMF-M321
DBMF-M499
DBMF-M473
DBMF-M667
DBMF-M470
DBMF-M73
DBMF-M665
DBMF-M832
DBMF-M497
Parastromateus niger
DBMF-M49
DBMF-M20
Carangoides ferdau
DBMF-M654
DBMF-M656
DBMF-M655
DBMF-M641
DBMF-M724
DBMF-M723
Carangoides dinema
DBMF-M181
DBMF-M730
DBMF-M728
DBMF-M47
DBMF-M618
DBMF-M729
DBMF-M488
DBMF-M182
Alectis ciliaris
DBMF-M560
DBMF-M727
DBMF-M19
DBMF-M487
DBMF-M726
DBMF-M485
DBMF-M486
DBMF-M45
DBMF-M68
DBMF-M561
Alectis indicus
DBMF-M797
DBMF-M554
DBMF-M592
DBMF-M389
DBMF-M17
DBMF-M593
DBMF-M590
DBMF-M591
DBMF-M552
DBMF-M718
DBMF-M589
DBMF-M800
DBMF-M799
DBMF-M553
DBMF-M798
DBMF-M387
DBMF-M388
DBMF-M386
DBMF-M796
Carangoides chrysophrys
DBMF-M122
DBMF-M123
DBMF-M121
DBMF-M700
DBMF-M701
DBMF-M699
DBMF-M40
DBMF-M384
DBMF-M702
DBMF-M383
Atropus atropos

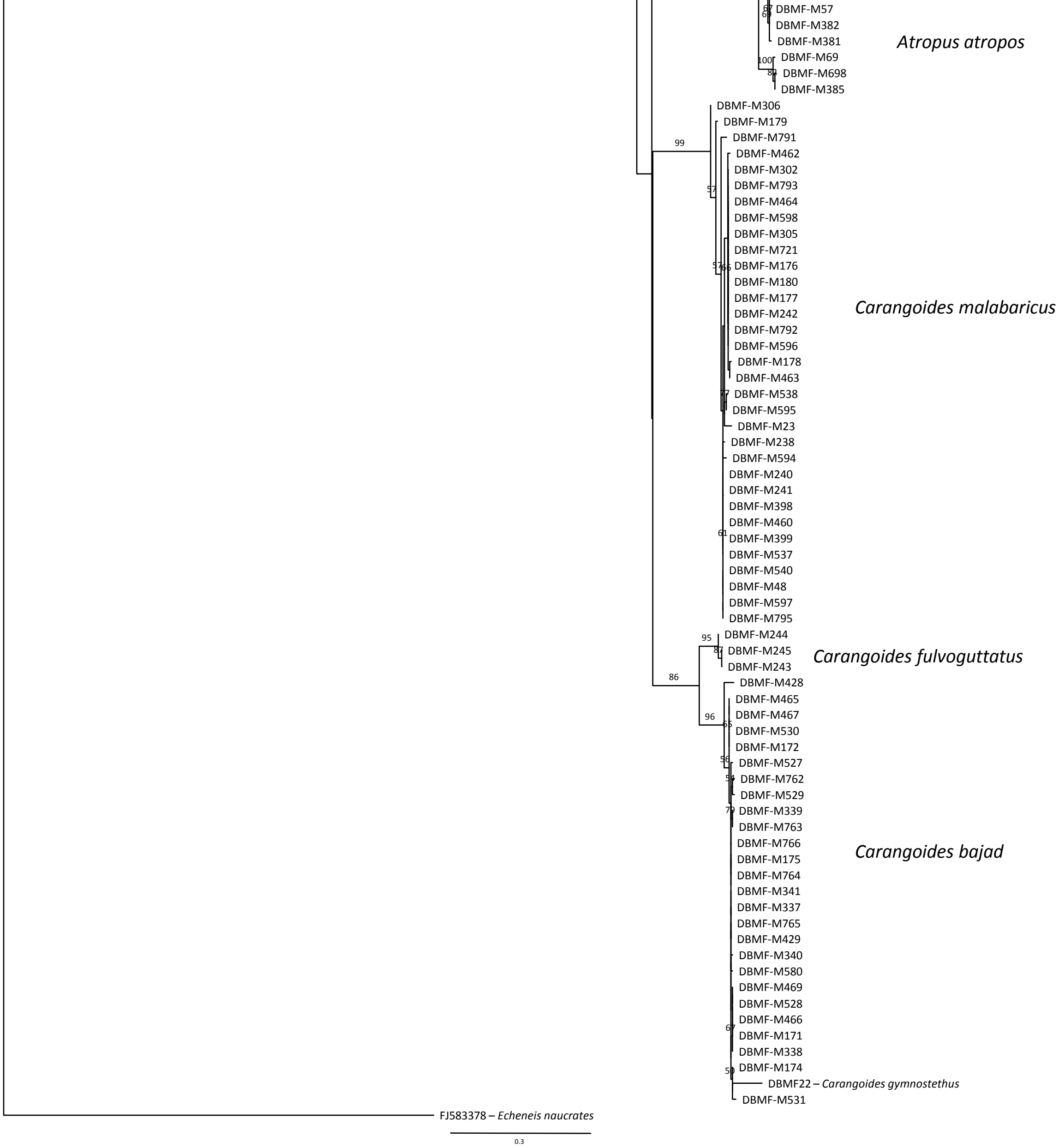
DBMF-M57
DBMF-M382
DBMF-M381
Atropus atropos
DBMF-M69
DBMF-M698
DBMF-M385
DBMF-M306
DBMF-M179
DBMF-M791
DBMF-M462
DBMF-M302
DBMF-M793
DBMF-M464
DBMF-M598
DBMF-M305
DBMF-M721
DBMF-M176
DBMF-M180
DBMF-M177
DBMF-M242
DBMF-M792
DBMF-M596
DBMF-M178
DBMF-M463
DBMF-M538
DBMF-M595
DBMF-M23
DBMF-M238
DBMF-M594
DBMF-M240
DBMF-M241
DBMF-M398
DBMF-M460
DBMF-M399
DBMF-M537
DBMF-M540
DBMF-M48
DBMF-M597
DBMF-M795
Carangoides malabaricus
DBMF-M244
DBMF-M245
DBMF-M243
Carangoides fulvoguttatus
DBMF-M428
DBMF-M465
DBMF-M467
DBMF-M530
DBMF-M172
DBMF-M527
DBMF-M762
DBMF-M529
DBMF-M339
DBMF-M763
DBMF-M766
DBMF-M175
DBMF-M764
DBMF-M341
DBMF-M337
DBMF-M765
DBMF-M429
DBMF-M340
DBMF-M580
DBMF-M469
DBMF-M528
DBMF-M466
DBMF-M171
DBMF-M338
DBMF-M174
DBMF22 – Carangoides gymnostethus
DBMF-M531
Carangoides bajad
FJ583378 – Echeneis naucrates
0.3
