# Supplementary material for: DNA Barcoding Reveals Cryptic Diversity within Commercially Exploited Indo-Malay Carangidae (Teleosteii: Perciformes)
Source: PLoS One. 2012 Nov 29;7(11):e49623. doi: 10.1371/journal.pone.0049623 (PMC3510217; doi:10.1371/journal.pone.0049623)
Supplement: Figure S3 — Tree corresponding to partition detected by ABGD method. (PDF) [file pone.0049623.s003.pdf]

Figure S3. Tree corresponding to partition detected by ABGD method.

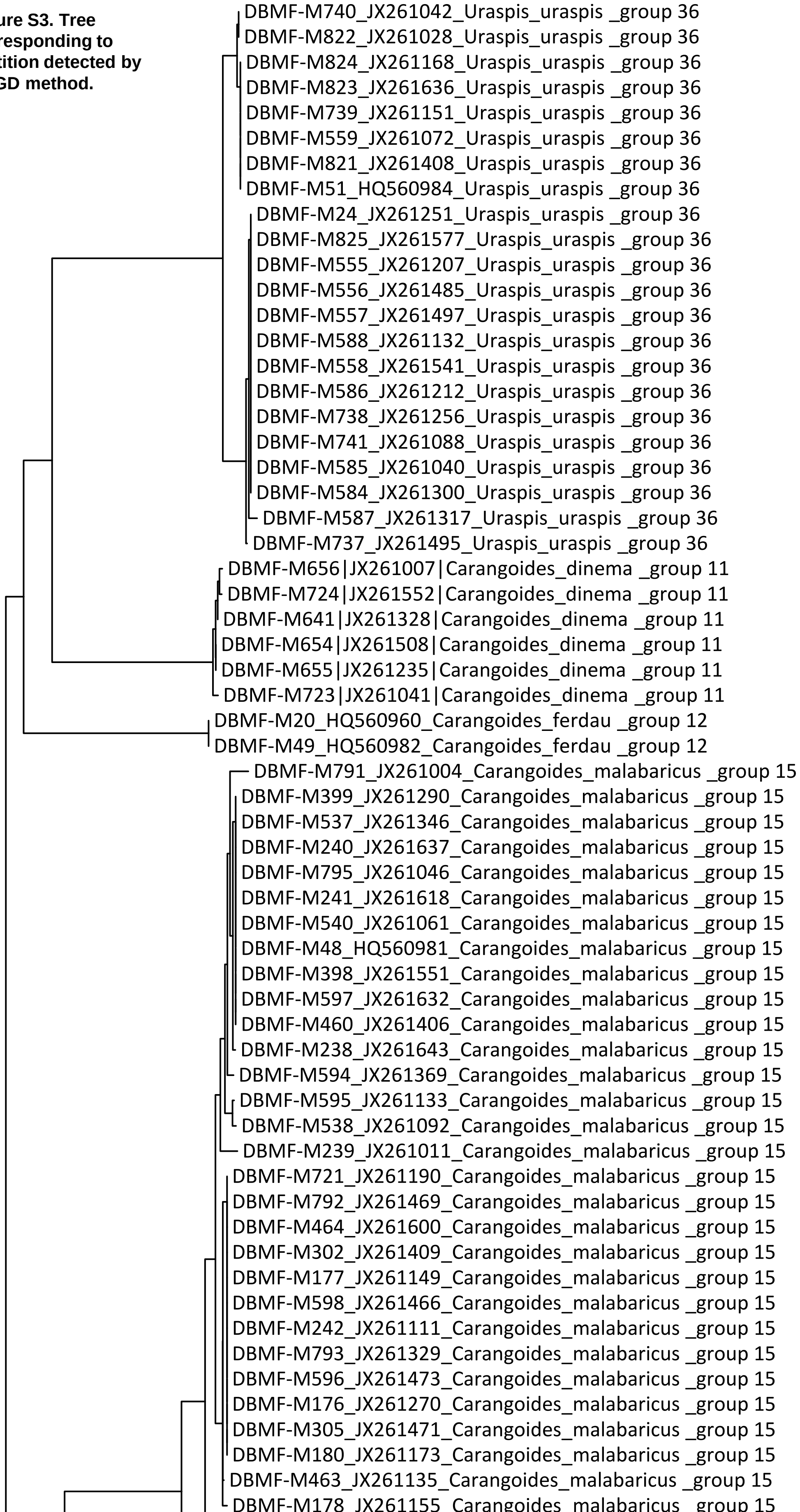

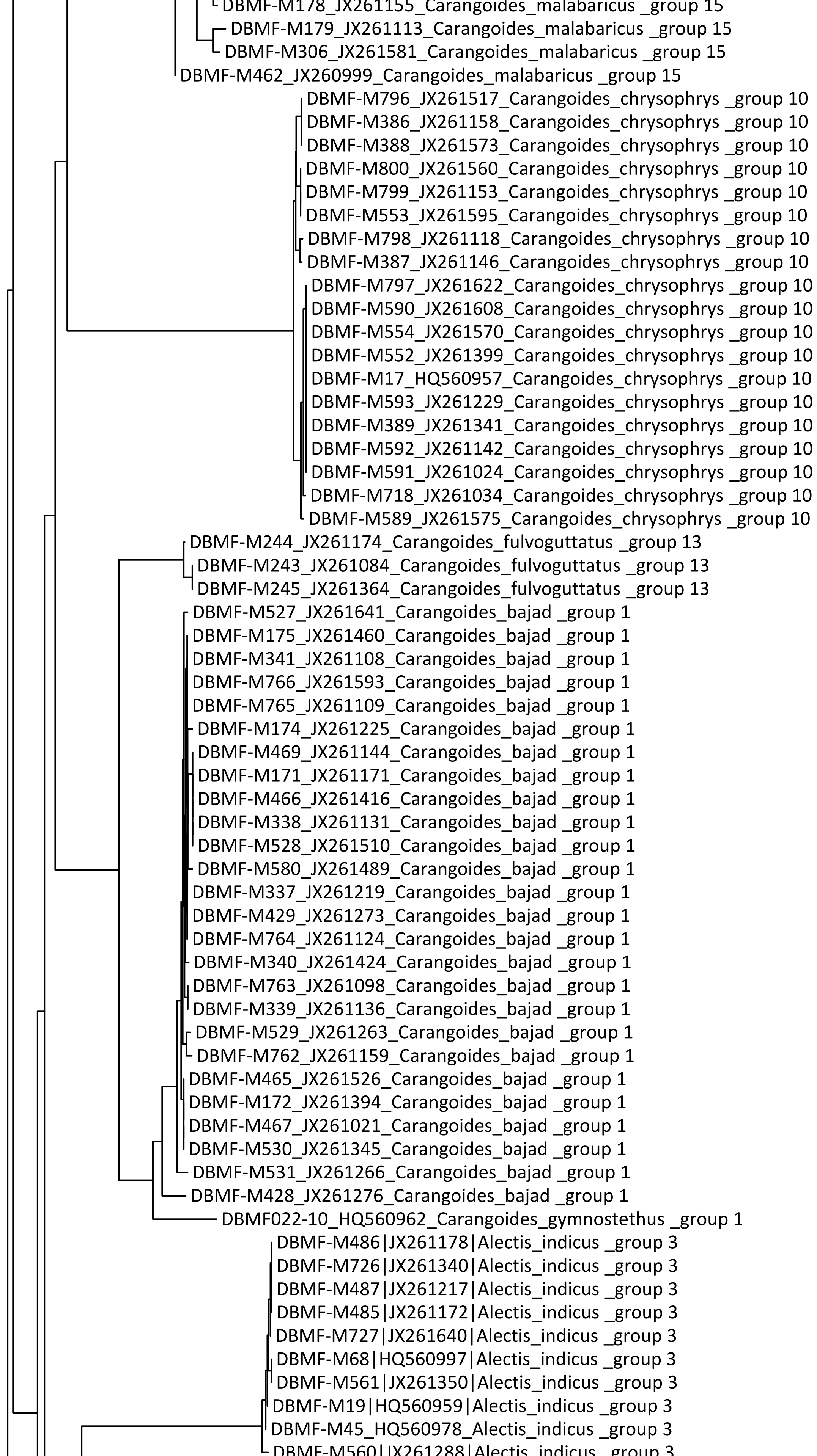

DBMF-M178_JX261155_Carangoides_malabaricus _group 15
DBMF-M179_JX261113_Carangoides_malabaricus _group 15
DBMF-M306_JX261581_Carangoides_malabaricus _group 15
DBMF-M462_JX260999_Carangoides_malabaricus _group 15
DBMF-M796_JX261517_Carangoides_chrysophrys _group 10
DBMF-M386_JX261158_Carangoides_chrysophrys _group 10
DBMF-M388_JX261573_Carangoides_chrysophrys _group 10
DBMF-M800_JX261560_Carangoides_chrysophrys _group 10
DBMF-M799_JX261153_Carangoides_chrysophrys _group 10
DBMF-M553_JX261595_Carangoides_chrysophrys _group 10
DBMF-M798_JX261118_Carangoides_chrysophrys _group 10
DBMF-M387_JX261146_Carangoides_chrysophrys _group 10
DBMF-M797_JX261622_Carangoides_chrysophrys _group 10
DBMF-M590_JX261608_Carangoides_chrysophrys _group 10
DBMF-M554_JX261570_Carangoides_chrysophrys _group 10
DBMF-M552_JX261399_Carangoides_chrysophrys _group 10
DBMF-M17_HQ560957_Carangoides_chrysophrys _group 10
DBMF-M593_JX261229_Carangoides_chrysophrys _group 10
DBMF-M389_JX261341_Carangoides_chrysophrys _group 10
DBMF-M592_JX261142_Carangoides_chrysophrys _group 10
DBMF-M591_JX261024_Carangoides_chrysophrys _group 10
DBMF-M718_JX261034_Carangoides_chrysophrys _group 10
DBMF-M589_JX261575_Carangoides_chrysophrys _group 10
DBMF-M244_JX261174_Carangoides_fulvoguttatus _group 13
DBMF-M243_JX261084_Carangoides_fulvoguttatus _group 13
DBMF-M245_JX261364_Carangoides_fulvoguttatus _group 13
DBMF-M527_JX261641_Carangoides_bajad _group 1
DBMF-M175_JX261460_Carangoides_bajad _group 1
DBMF-M341_JX261108_Carangoides_bajad _group 1
DBMF-M766_JX261593_Carangoides_bajad _group 1
DBMF-M765_JX261109_Carangoides_bajad _group 1
DBMF-M174_JX261225_Carangoides_bajad _group 1
DBMF-M469_JX261144_Carangoides_bajad _group 1
DBMF-M171_JX261171_Carangoides_bajad _group 1
DBMF-M466_JX261416_Carangoides_bajad _group 1
DBMF-M338_JX261131_Carangoides_bajad _group 1
DBMF-M528_JX261510_Carangoides_bajad _group 1
DBMF-M580_JX261489_Carangoides_bajad _group 1
DBMF-M337_JX261219_Carangoides_bajad _group 1
DBMF-M429_JX261273_Carangoides_bajad _group 1
DBMF-M764_JX261124_Carangoides_bajad _group 1
DBMF-M340_JX261424_Carangoides_bajad _group 1
DBMF-M763_JX261098_Carangoides_bajad _group 1
DBMF-M339_JX261136_Carangoides_bajad _group 1
DBMF-M529_JX261263_Carangoides_bajad _group 1
DBMF-M762_JX261159_Carangoides_bajad _group 1
DBMF-M465_JX261526_Carangoides_bajad _group 1
DBMF-M172_JX261394_Carangoides_bajad _group 1
DBMF-M467_JX261021_Carangoides_bajad _group 1
DBMF-M530_JX261345_Carangoides_bajad _group 1
DBMF-M531_JX261266_Carangoides_bajad _group 1
DBMF-M428_JX261276_Carangoides_bajad _group 1
DBMF022-10_HQ560962_Carangoides_gymnostethus _group 1
DBMF-M486|JX261178|Alectis_indicus _group 3
DBMF-M726|JX261340|Alectis_indicus _group 3
DBMF-M487|JX261217|Alectis_indicus _group 3
DBMF-M485|JX261172|Alectis_indicus _group 3
DBMF-M727|JX261640|Alectis_indicus _group 3
DBMF-M68|HQ560997|Alectis_indicus _group 3
DBMF-M561|JX261350|Alectis_indicus _group 3
DBMF-M19|HQ560959|Alectis_indicus _group 3
DBMF-M45_HQ560978_Alectis_indicus _group 3
DBMF-M560|JX261288|Alectis_indicus _group 3

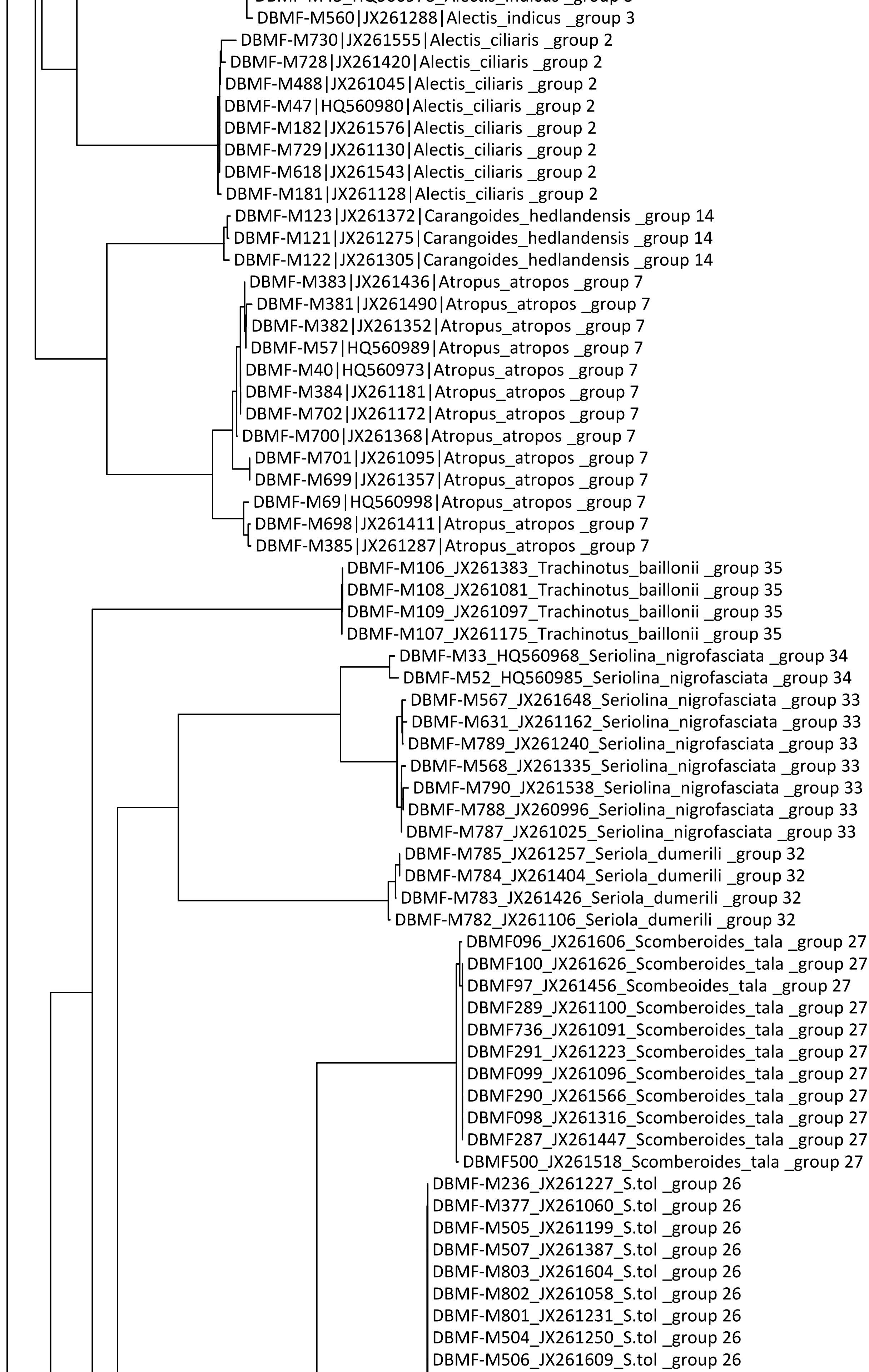
DBMF-M560|JX261288|Alectis_indicus _group 3
DBMF-M730|JX261555|Alectis_ciliaris _group 2
DBMF-M728|JX261420|Alectis_ciliaris _group 2
DBMF-M488|JX261045|Alectis_ciliaris _group 2
DBMF-M47|HQ560980|Alectis_ciliaris _group 2
DBMF-M182|JX261576|Alectis_ciliaris _group 2
DBMF-M729|JX261130|Alectis_ciliaris _group 2
DBMF-M618|JX261543|Alectis_ciliaris _group 2
DBMF-M181|JX261128|Alectis_ciliaris _group 2
DBMF-M123|JX261372|Carangoides_hedlandensis _group 14
DBMF-M121|JX261275|Carangoides_hedlandensis _group 14
DBMF-M122|JX261305|Carangoides_hedlandensis _group 14
DBMF-M383|JX261436|Atropus_atropos _group 7
DBMF-M381|JX261490|Atropus_atropos _group 7
DBMF-M382|JX261352|Atropus_atropos _group 7
DBMF-M57|HQ560989|Atropus_atropos _group 7
DBMF-M40|HQ560973|Atropus_atropos _group 7
DBMF-M384|JX261181|Atropus_atropos _group 7
DBMF-M702|JX261172|Atropus_atropos _group 7
DBMF-M700|JX261368|Atropus_atropos _group 7
DBMF-M701|JX261095|Atropus_atropos _group 7
DBMF-M699|JX261357|Atropus_atropos _group 7
DBMF-M69|HQ560998|Atropus_atropos _group 7
DBMF-M698|JX261411|Atropus_atropos _group 7
DBMF-M385|JX261287|Atropus_atropos _group 7
DBMF-M106_JX261383_Trachinotus_baillonii _group 35
DBMF-M108_JX261081_Trachinotus_baillonii _group 35
DBMF-M109_JX261097_Trachinotus_baillonii _group 35
DBMF-M107_JX261175_Trachinotus_baillonii _group 35
DBMF-M33_HQ560968_Seriolina_nigrofasciata _group 34
DBMF-M52_HQ560985_Seriolina_nigrofasciata _group 34
DBMF-M567_JX261648_Seriolina_nigrofasciata _group 33
DBMF-M631_JX261162_Seriolina_nigrofasciata _group 33
DBMF-M789_JX261240_Seriolina_nigrofasciata _group 33
DBMF-M568_JX261335_Seriolina_nigrofasciata _group 33
DBMF-M790_JX261538_Seriolina_nigrofasciata _group 33
DBMF-M788_JX260996_Seriolina_nigrofasciata _group 33
DBMF-M787_JX261025_Seriolina_nigrofasciata _group 33
DBMF-M785_JX261257_Seriola_dumerili _group 32
DBMF-M784_JX261404_Seriola_dumerili _group 32
DBMF-M783_JX261426_Seriola_dumerili _group 32
DBMF-M782_JX261106_Seriola_dumerili _group 32
DBMF096_JX261606_Scomberoides_tala _group 27
DBMF100_JX261626_Scomberoides_tala _group 27
DBMF97_JX261456_Scombeoides_tala _group 27
DBMF289_JX261100_Scomberoides_tala _group 27
DBMF736_JX261091_Scomberoides_tala _group 27
DBMF291_JX261223_Scomberoides_tala _group 27
DBMF099_JX261096_Scomberoides_tala _group 27
DBMF290_JX261566_Scomberoides_tala _group 27
DBMF098_JX261316_Scomberoides_tala _group 27
DBMF287_JX261447_Scomberoides_tala _group 27
DBMF500_JX261518_Scomberoides_tala _group 27
DBMF-M236_JX261227_S.tol _group 26
DBMF-M377_JX261060_S.tol _group 26
DBMF-M505_JX261199_S.tol _group 26
DBMF-M507_JX261387_S.tol _group 26
DBMF-M803_JX261604_S.tol _group 26
DBMF-M802_JX261058_S.tol _group 26
DBMF-M801_JX261231_S.tol _group 26
DBMF-M504_JX261250_S.tol _group 26
DBMF-M506_JX261609_S.tol _group 26

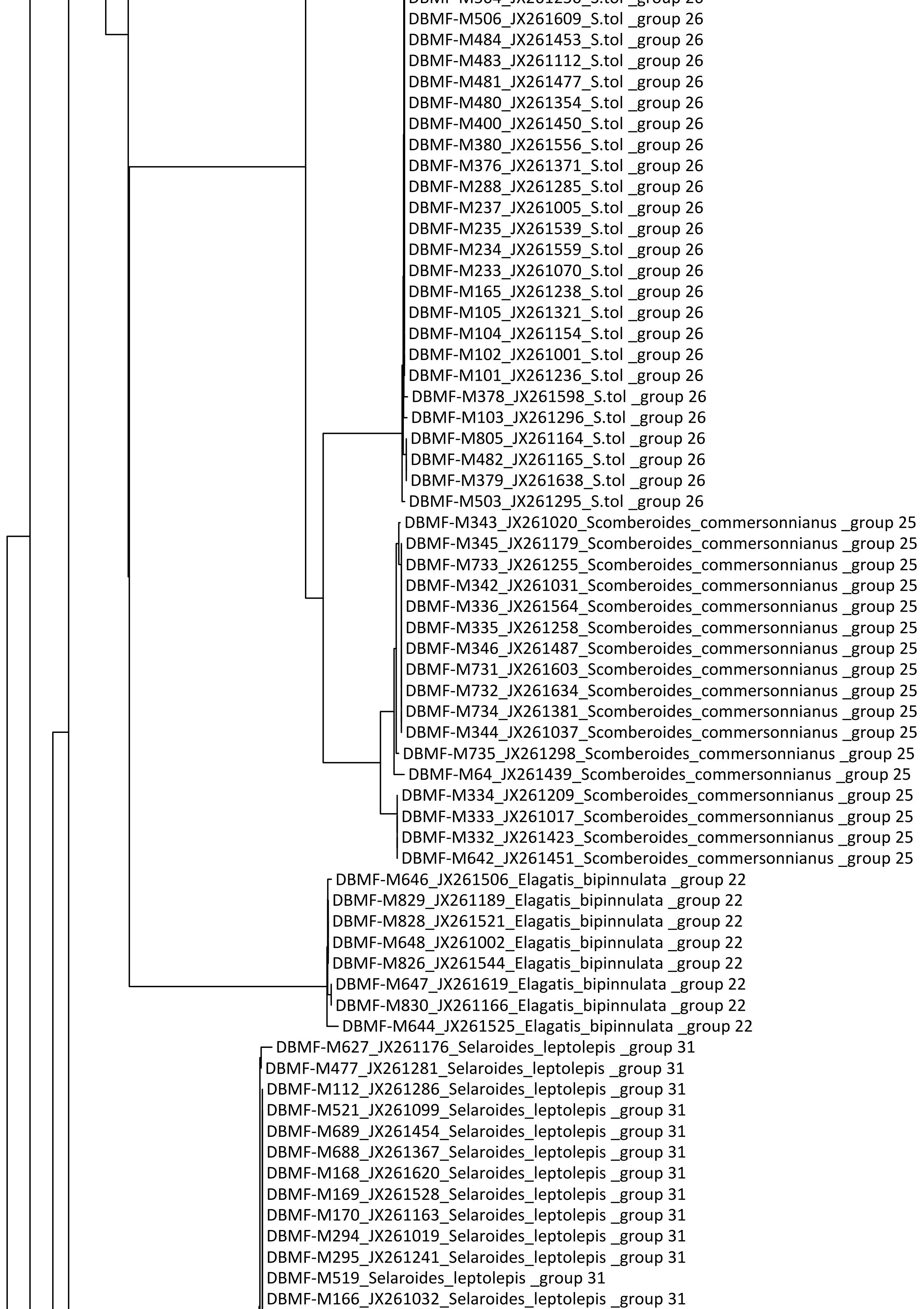

DBMF-M506_JX261609_S.tol _group 26
DBMF-M484_JX261453_S.tol _group 26
DBMF-M483_JX261112_S.tol _group 26
DBMF-M481_JX261477_S.tol _group 26
DBMF-M480_JX261354_S.tol _group 26
DBMF-M400_JX261450_S.tol _group 26
DBMF-M380_JX261556_S.tol _group 26
DBMF-M376_JX261371_S.tol _group 26
DBMF-M288_JX261285_S.tol _group 26
DBMF-M237_JX261005_S.tol _group 26
DBMF-M235_JX261539_S.tol _group 26
DBMF-M234_JX261559_S.tol _group 26
DBMF-M233_JX261070_S.tol _group 26
DBMF-M165_JX261238_S.tol _group 26
DBMF-M105_JX261321_S.tol _group 26
DBMF-M104_JX261154_S.tol _group 26
DBMF-M102_JX261001_S.tol _group 26
DBMF-M101_JX261236_S.tol _group 26
DBMF-M378_JX261598_S.tol _group 26
DBMF-M103_JX261296_S.tol _group 26
DBMF-M805_JX261164_S.tol _group 26
DBMF-M482_JX261165_S.tol _group 26
DBMF-M379_JX261638_S.tol _group 26
DBMF-M503_JX261295_S.tol _group 26
DBMF-M343_JX261020_Scomberoides_commersonnianus _group 25
DBMF-M345_JX261179_Scomberoides_commersonnianus _group 25
DBMF-M733_JX261255_Scomberoides_commersonnianus _group 25
DBMF-M342_JX261031_Scomberoides_commersonnianus _group 25
DBMF-M336_JX261564_Scomberoides_commersonnianus _group 25
DBMF-M335_JX261258_Scomberoides_commersonnianus _group 25
DBMF-M346_JX261487_Scomberoides_commersonnianus _group 25
DBMF-M731_JX261603_Scomberoides_commersonnianus _group 25
DBMF-M732_JX261634_Scomberoides_commersonnianus _group 25
DBMF-M734_JX261381_Scomberoides_commersonnianus _group 25
DBMF-M344_JX261037_Scomberoides_commersonnianus _group 25
DBMF-M735_JX261298_Scomberoides_commersonnianus _group 25
DBMF-M64_JX261439_Scomberoides_commersonnianus _group 25
DBMF-M334_JX261209_Scomberoides_commersonnianus _group 25
DBMF-M333_JX261017_Scomberoides_commersonnianus _group 25
DBMF-M332_JX261423_Scomberoides_commersonnianus _group 25
DBMF-M642_JX261451_Scomberoides_commersonnianus _group 25
DBMF-M646_JX261506_Elagatis_bipinnulata _group 22
DBMF-M829_JX261189_Elagatis_bipinnulata _group 22
DBMF-M828_JX261521_Elagatis_bipinnulata _group 22
DBMF-M648_JX261002_Elagatis_bipinnulata _group 22
DBMF-M826_JX261544_Elagatis_bipinnulata _group 22
DBMF-M647_JX261619_Elagatis_bipinnulata _group 22
DBMF-M830_JX261166_Elagatis_bipinnulata _group 22
DBMF-M644_JX261525_Elagatis_bipinnulata _group 22
DBMF-M627_JX261176_Selaroides_leptolepis _group 31
DBMF-M477_JX261281_Selaroides_leptolepis _group 31
DBMF-M112_JX261286_Selaroides_leptolepis _group 31
DBMF-M521_JX261099_Selaroides_leptolepis _group 31
DBMF-M689_JX261454_Selaroides_leptolepis _group 31
DBMF-M688_JX261367_Selaroides_leptolepis _group 31
DBMF-M168_JX261620_Selaroides_leptolepis _group 31
DBMF-M169_JX261528_Selaroides_leptolepis _group 31
DBMF-M170_JX261163_Selaroides_leptolepis _group 31
DBMF-M294_JX261019_Selaroides_leptolepis _group 31
DBMF-M295_JX261241_Selaroides_leptolepis _group 31
DBMF-M519_Selaroides_leptolepis _group 31
DBMF-M166_JX261032_Selaroides_leptolepis _group 31

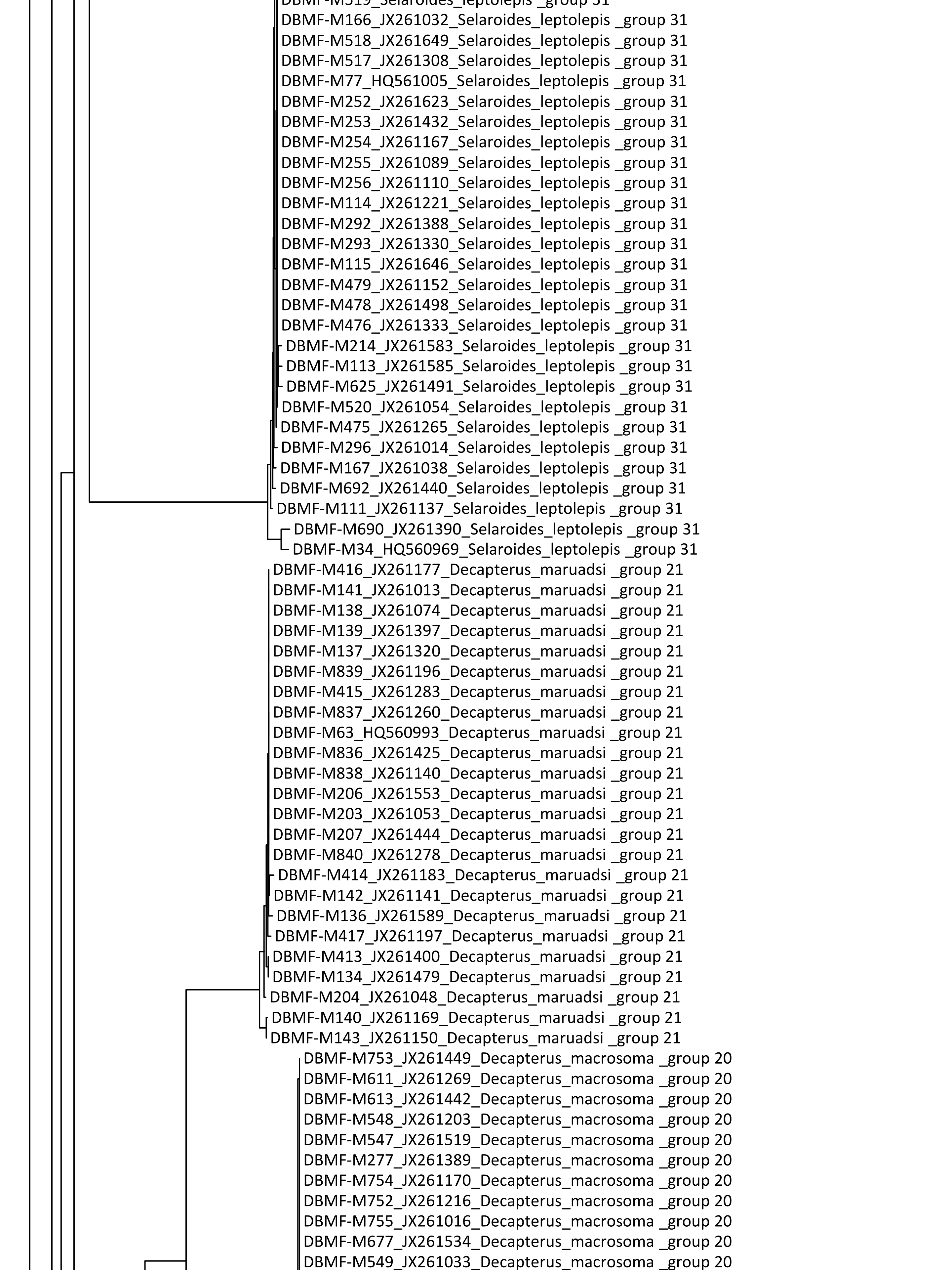

DBMF-M519_Selaroides_leptolepis _group 31
DBMF-M166_JX261032_Selaroides_leptolepis _group 31
DBMF-M518_JX261649_Selaroides_leptolepis _group 31
DBMF-M517_JX261308_Selaroides_leptolepis _group 31
DBMF-M77_HQ561005_Selaroides_leptolepis _group 31
DBMF-M252_JX261623_Selaroides_leptolepis _group 31
DBMF-M253_JX261432_Selaroides_leptolepis _group 31
DBMF-M254_JX261167_Selaroides_leptolepis _group 31
DBMF-M255_JX261089_Selaroides_leptolepis _group 31
DBMF-M256_JX261110_Selaroides_leptolepis _group 31
DBMF-M114_JX261221_Selaroides_leptolepis _group 31
DBMF-M292_JX261388_Selaroides_leptolepis _group 31
DBMF-M293_JX261330_Selaroides_leptolepis _group 31
DBMF-M115_JX261646_Selaroides_leptolepis _group 31
DBMF-M479_JX261152_Selaroides_leptolepis _group 31
DBMF-M478_JX261498_Selaroides_leptolepis _group 31
DBMF-M476_JX261333_Selaroides_leptolepis _group 31
DBMF-M214_JX261583_Selaroides_leptolepis _group 31
DBMF-M113_JX261585_Selaroides_leptolepis _group 31
DBMF-M625_JX261491_Selaroides_leptolepis _group 31
DBMF-M520_JX261054_Selaroides_leptolepis _group 31
DBMF-M475_JX261265_Selaroides_leptolepis _group 31
DBMF-M296_JX261014_Selaroides_leptolepis _group 31
DBMF-M167_JX261038_Selaroides_leptolepis _group 31
DBMF-M692_JX261440_Selaroides_leptolepis _group 31
DBMF-M111_JX261137_Selaroides_leptolepis _group 31
DBMF-M690_JX261390_Selaroides_leptolepis _group 31
DBMF-M34_HQ560969_Selaroides_leptolepis _group 31
DBMF-M416_JX261177_Decapterus_maruadsi _group 21
DBMF-M141_JX261013_Decapterus_maruadsi _group 21
DBMF-M138_JX261074_Decapterus_maruadsi _group 21
DBMF-M139_JX261397_Decapterus_maruadsi _group 21
DBMF-M137_JX261320_Decapterus_maruadsi _group 21
DBMF-M839_JX261196_Decapterus_maruadsi _group 21
DBMF-M415_JX261283_Decapterus_maruadsi _group 21
DBMF-M837_JX261260_Decapterus_maruadsi _group 21
DBMF-M63_HQ560993_Decapterus_maruadsi _group 21
DBMF-M836_JX261425_Decapterus_maruadsi _group 21
DBMF-M838_JX261140_Decapterus_maruadsi _group 21
DBMF-M206_JX261553_Decapterus_maruadsi _group 21
DBMF-M203_JX261053_Decapterus_maruadsi _group 21
DBMF-M207_JX261444_Decapterus_maruadsi _group 21
DBMF-M840_JX261278_Decapterus_maruadsi _group 21
DBMF-M414_JX261183_Decapterus_maruadsi _group 21
DBMF-M142_JX261141_Decapterus_maruadsi _group 21
DBMF-M136_JX261589_Decapterus_maruadsi _group 21
DBMF-M417_JX261197_Decapterus_maruadsi _group 21
DBMF-M413_JX261400_Decapterus_maruadsi _group 21
DBMF-M134_JX261479_Decapterus_maruadsi _group 21
DBMF-M204_JX261048_Decapterus_maruadsi _group 21
DBMF-M140_JX261169_Decapterus_maruadsi _group 21
DBMF-M143_JX261150_Decapterus_maruadsi _group 21
DBMF-M753_JX261449_Decapterus_macrosoma _group 20
DBMF-M611_JX261269_Decapterus_macrosoma _group 20
DBMF-M613_JX261442_Decapterus_macrosoma _group 20
DBMF-M548_JX261203_Decapterus_macrosoma _group 20
DBMF-M547_JX261519_Decapterus_macrosoma _group 20
DBMF-M277_JX261389_Decapterus_macrosoma _group 20
DBMF-M754_JX261170_Decapterus_macrosoma _group 20
DBMF-M752_JX261216_Decapterus_macrosoma _group 20
DBMF-M755_JX261016_Decapterus_macrosoma _group 20
DBMF-M677_JX261534_Decapterus_macrosoma _group 20
DBMF-M549_JX261033_Decapterus_macrosoma _group 20

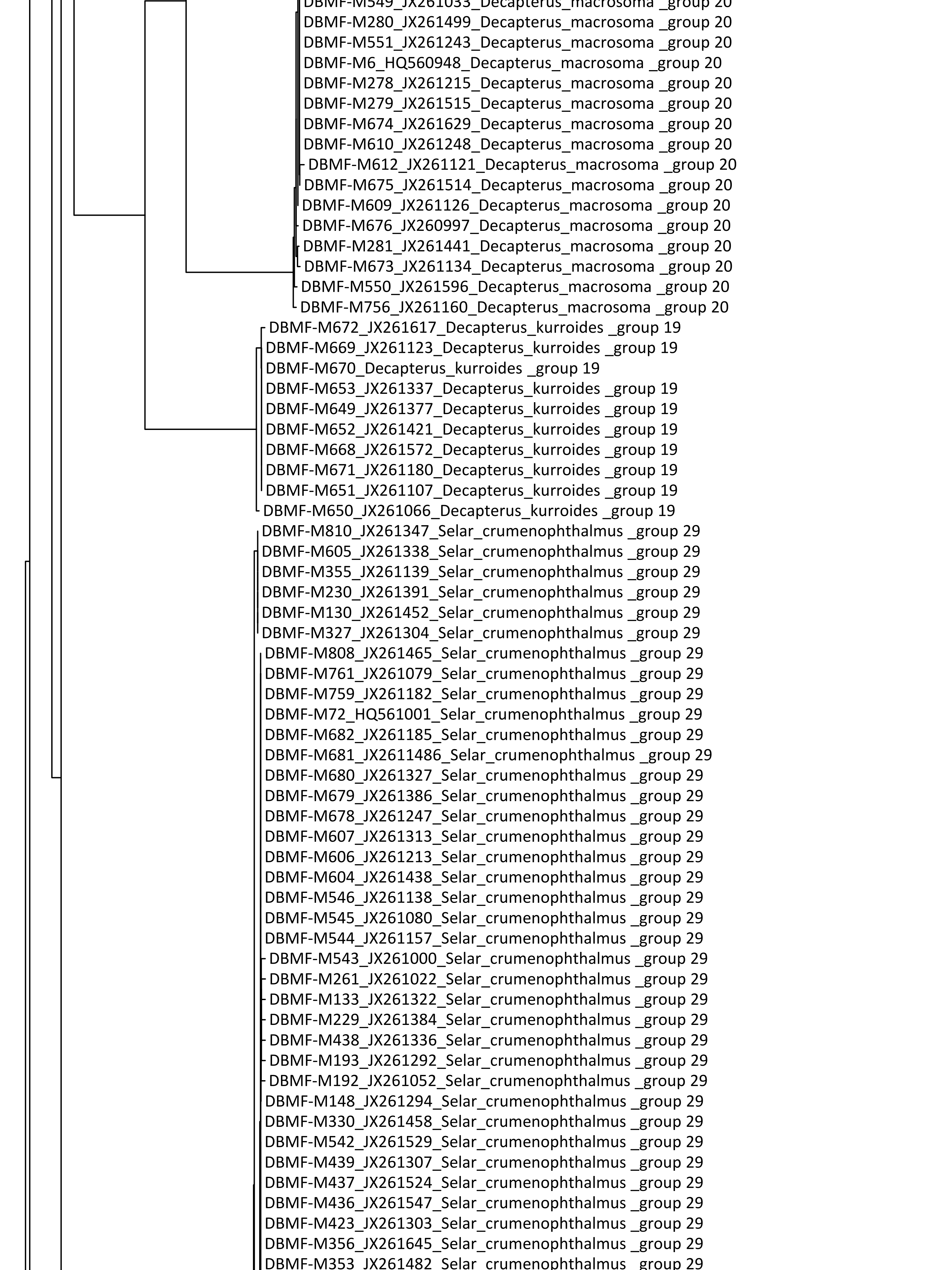
DBMF-M549_JX261033_Decapterus_macrosoma _group 20
DBMF-M280_JX261499_Decapterus_macrosoma _group 20
DBMF-M551_JX261243_Decapterus_macrosoma _group 20
DBMF-M6_HQ560948_Decapterus_macrosoma _group 20
DBMF-M278_JX261215_Decapterus_macrosoma _group 20
DBMF-M279_JX261515_Decapterus_macrosoma _group 20
DBMF-M674_JX261629_Decapterus_macrosoma _group 20
DBMF-M610_JX261248_Decapterus_macrosoma _group 20
DBMF-M612_JX261121_Decapterus_macrosoma _group 20
DBMF-M675_JX261514_Decapterus_macrosoma _group 20
DBMF-M609_JX261126_Decapterus_macrosoma _group 20
DBMF-M676_JX260997_Decapterus_macrosoma _group 20
DBMF-M281_JX261441_Decapterus_macrosoma _group 20
DBMF-M673_JX261134_Decapterus_macrosoma _group 20
DBMF-M550_JX261596_Decapterus_macrosoma _group 20
DBMF-M756_JX261160_Decapterus_macrosoma _group 20
DBMF-M672_JX261617_Decapterus_kurroides _group 19
DBMF-M669_JX261123_Decapterus_kurroides _group 19
DBMF-M670_Decapterus_kurroides _group 19
DBMF-M653_JX261337_Decapterus_kurroides _group 19
DBMF-M649_JX261377_Decapterus_kurroides _group 19
DBMF-M652_JX261421_Decapterus_kurroides _group 19
DBMF-M668_JX261572_Decapterus_kurroides _group 19
DBMF-M671_JX261180_Decapterus_kurroides _group 19
DBMF-M651_JX261107_Decapterus_kurroides _group 19
DBMF-M650_JX261066_Decapterus_kurroides _group 19
DBMF-M810_JX261347_Selar_crumenophthalmus _group 29
DBMF-M605_JX261338_Selar_crumenophthalmus _group 29
DBMF-M355_JX261139_Selar_crumenophthalmus _group 29
DBMF-M230_JX261391_Selar_crumenophthalmus _group 29
DBMF-M130_JX261452_Selar_crumenophthalmus _group 29
DBMF-M327_JX261304_Selar_crumenophthalmus _group 29
DBMF-M808_JX261465_Selar_crumenophthalmus _group 29
DBMF-M761_JX261079_Selar_crumenophthalmus _group 29
DBMF-M759_JX261182_Selar_crumenophthalmus _group 29
DBMF-M72_HQ561001_Selar_crumenophthalmus _group 29
DBMF-M682_JX261185_Selar_crumenophthalmus _group 29
DBMF-M681_JX2611486_Selar_crumenophthalmus _group 29
DBMF-M680_JX261327_Selar_crumenophthalmus _group 29
DBMF-M679_JX261386_Selar_crumenophthalmus _group 29
DBMF-M678_JX261247_Selar_crumenophthalmus _group 29
DBMF-M607_JX261313_Selar_crumenophthalmus _group 29
DBMF-M606_JX261213_Selar_crumenophthalmus _group 29
DBMF-M604_JX261438_Selar_crumenophthalmus _group 29
DBMF-M546_JX261138_Selar_crumenophthalmus _group 29
DBMF-M545_JX261080_Selar_crumenophthalmus _group 29
DBMF-M544_JX261157_Selar_crumenophthalmus _group 29
DBMF-M543_JX261000_Selar_crumenophthalmus _group 29
DBMF-M261_JX261022_Selar_crumenophthalmus _group 29
DBMF-M133_JX261322_Selar_crumenophthalmus _group 29
DBMF-M229_JX261384_Selar_crumenophthalmus _group 29
DBMF-M438_JX261336_Selar_crumenophthalmus _group 29
DBMF-M193_JX261292_Selar_crumenophthalmus _group 29
DBMF-M192_JX261052_Selar_crumenophthalmus _group 29
DBMF-M148_JX261294_Selar_crumenophthalmus _group 29
DBMF-M330_JX261458_Selar_crumenophthalmus _group 29
DBMF-M542_JX261529_Selar_crumenophthalmus _group 29
DBMF-M439_JX261307_Selar_crumenophthalmus _group 29
DBMF-M437_JX261524_Selar_crumenophthalmus _group 29
DBMF-M436_JX261547_Selar_crumenophthalmus _group 29
DBMF-M423_JX261303_Selar_crumenophthalmus _group 29
DBMF-M356_JX261645_Selar_crumenophthalmus _group 29
DBMF-M353_JX261482_Selar_crumenophthalmus _group 29

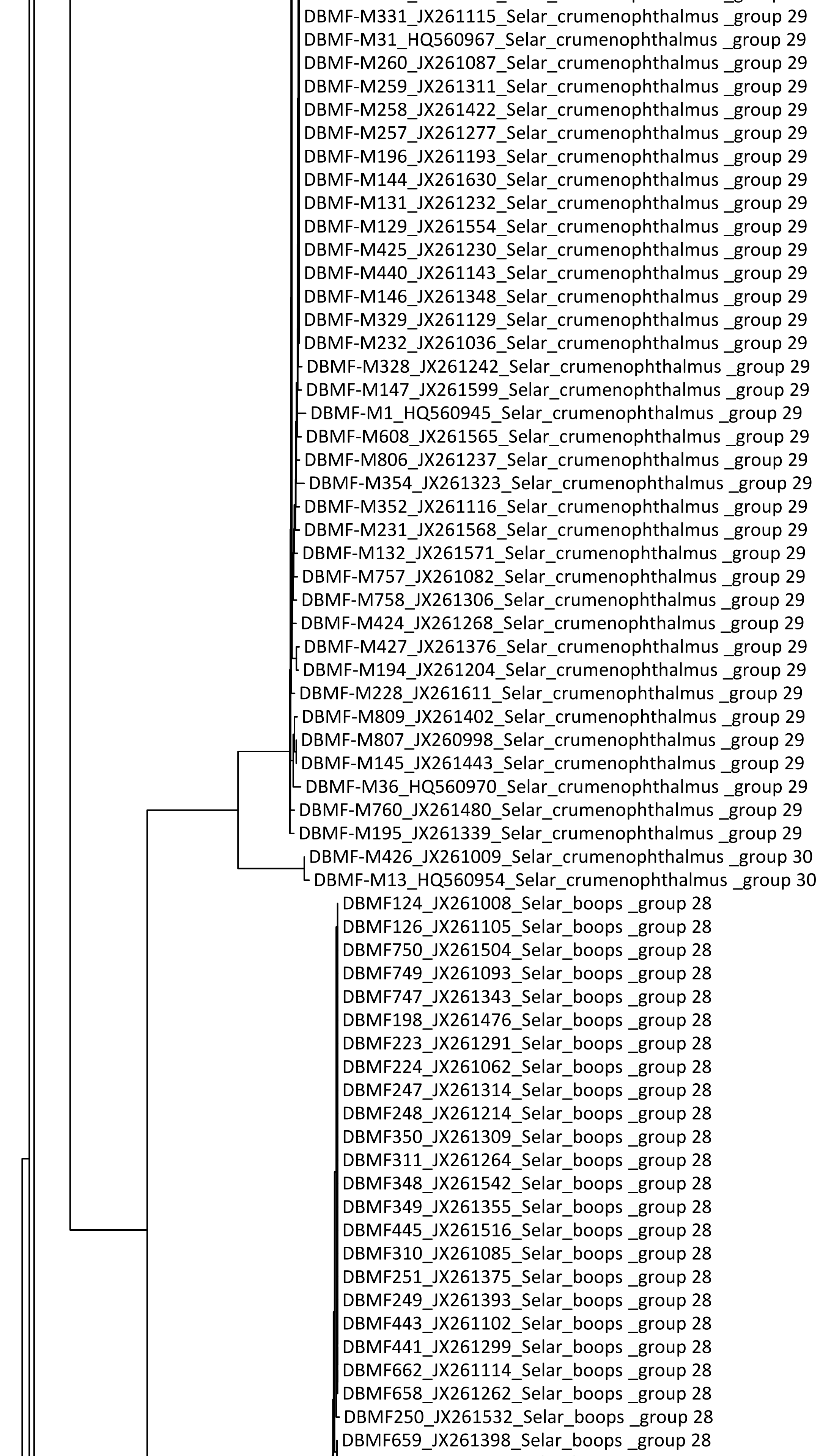
DBMF-M331_JX261115_Selar_crumenophthalmus _group 29
DBMF-M31_HQ560967_Selar_crumenophthalmus _group 29
DBMF-M260_JX261087_Selar_crumenophthalmus _group 29
DBMF-M259_JX261311_Selar_crumenophthalmus _group 29
DBMF-M258_JX261422_Selar_crumenophthalmus _group 29
DBMF-M257_JX261277_Selar_crumenophthalmus _group 29
DBMF-M196_JX261193_Selar_crumenophthalmus _group 29
DBMF-M144_JX261630_Selar_crumenophthalmus _group 29
DBMF-M131_JX261232_Selar_crumenophthalmus _group 29
DBMF-M129_JX261554_Selar_crumenophthalmus _group 29
DBMF-M425_JX261230_Selar_crumenophthalmus _group 29
DBMF-M440_JX261143_Selar_crumenophthalmus _group 29
DBMF-M146_JX261348_Selar_crumenophthalmus _group 29
DBMF-M329_JX261129_Selar_crumenophthalmus _group 29
DBMF-M232_JX261036_Selar_crumenophthalmus _group 29
DBMF-M328_JX261242_Selar_crumenophthalmus _group 29
DBMF-M147_JX261599_Selar_crumenophthalmus _group 29
DBMF-M1_HQ560945_Selar_crumenophthalmus _group 29
DBMF-M608_JX261565_Selar_crumenophthalmus _group 29
DBMF-M806_JX261237_Selar_crumenophthalmus _group 29
DBMF-M354_JX261323_Selar_crumenophthalmus _group 29
DBMF-M352_JX261116_Selar_crumenophthalmus _group 29
DBMF-M231_JX261568_Selar_crumenophthalmus _group 29
DBMF-M132_JX261571_Selar_crumenophthalmus _group 29
DBMF-M757_JX261082_Selar_crumenophthalmus _group 29
DBMF-M758_JX261306_Selar_crumenophthalmus _group 29
DBMF-M424_JX261268_Selar_crumenophthalmus _group 29
DBMF-M427_JX261376_Selar_crumenophthalmus _group 29
DBMF-M194_JX261204_Selar_crumenophthalmus _group 29
DBMF-M228_JX261611_Selar_crumenophthalmus _group 29
DBMF-M809_JX261402_Selar_crumenophthalmus _group 29
DBMF-M807_JX260998_Selar_crumenophthalmus _group 29
DBMF-M145_JX261443_Selar_crumenophthalmus _group 29
DBMF-M36_HQ560970_Selar_crumenophthalmus _group 29
DBMF-M760_JX261480_Selar_crumenophthalmus _group 29
DBMF-M195_JX261339_Selar_crumenophthalmus _group 29
DBMF-M426_JX261009_Selar_crumenophthalmus _group 30
DBMF-M13_HQ560954_Selar_crumenophthalmus _group 30
DBMF124_JX261008_Selar_boops _group 28
DBMF126_JX261105_Selar_boops _group 28
DBMF750_JX261504_Selar_boops _group 28
DBMF749_JX261093_Selar_boops _group 28
DBMF747_JX261343_Selar_boops _group 28
DBMF198_JX261476_Selar_boops _group 28
DBMF223_JX261291_Selar_boops _group 28
DBMF224_JX261062_Selar_boops _group 28
DBMF247_JX261314_Selar_boops _group 28
DBMF248_JX261214_Selar_boops _group 28
DBMF350_JX261309_Selar_boops _group 28
DBMF311_JX261264_Selar_boops _group 28
DBMF348_JX261542_Selar_boops _group 28
DBMF349_JX261355_Selar_boops _group 28
DBMF445_JX261516_Selar_boops _group 28
DBMF310_JX261085_Selar_boops _group 28
DBMF251_JX261375_Selar_boops _group 28
DBMF249_JX261393_Selar_boops _group 28
DBMF443_JX261102_Selar_boops _group 28
DBMF441_JX261299_Selar_boops _group 28
DBMF662_JX261114_Selar_boops _group 28
DBMF658_JX261262_Selar_boops _group 28
DBMF250_JX261532_Selar_boops _group 28
DBMF659_JX261398_Selar_boops _group 28

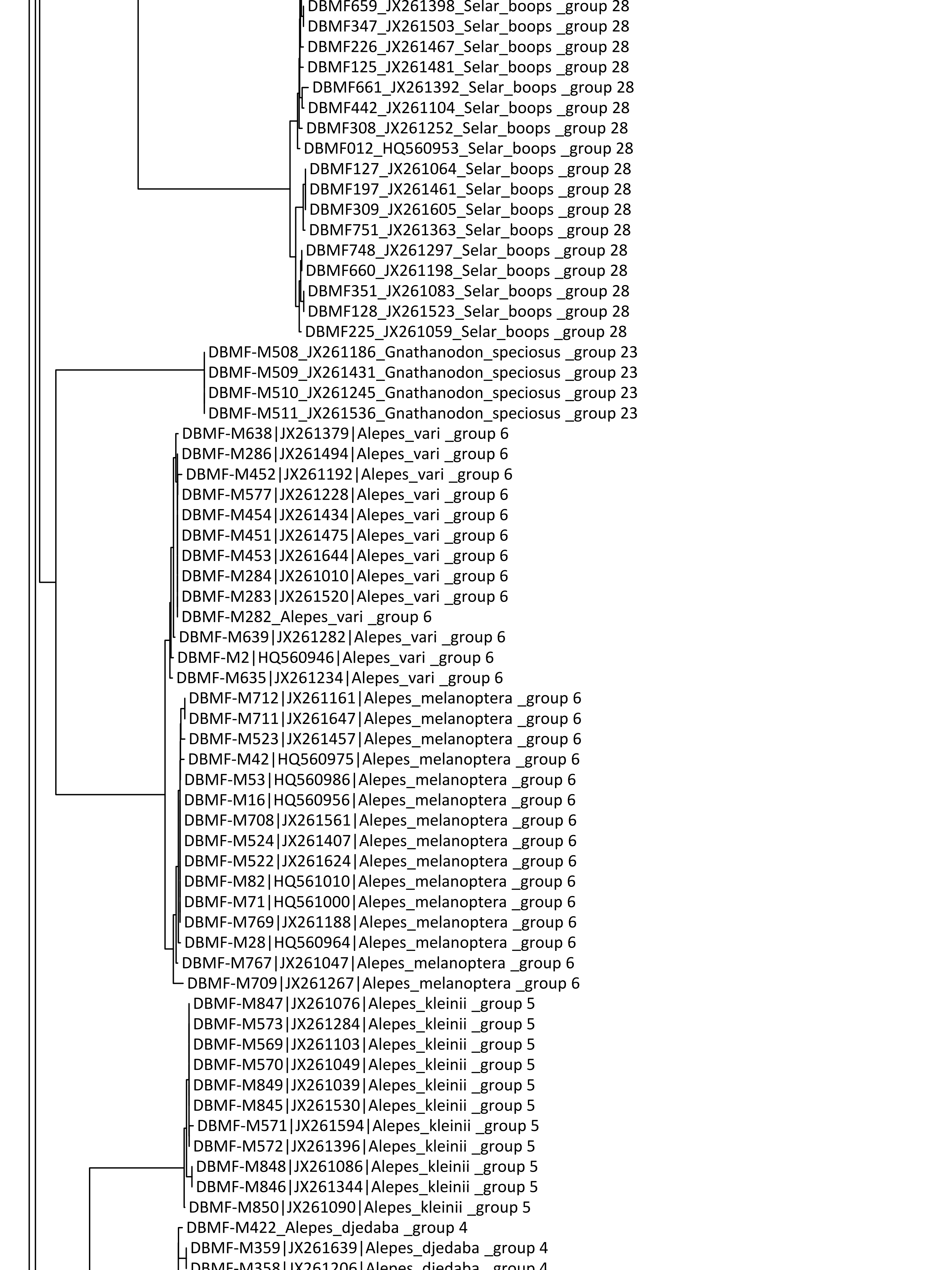
DBMF659_JX261398_Selar_boops _group 28
DBMF347_JX261503_Selar_boops _group 28
DBMF226_JX261467_Selar_boops _group 28
DBMF125_JX261481_Selar_boops _group 28
DBMF661_JX261392_Selar_boops _group 28
DBMF442_JX261104_Selar_boops _group 28
DBMF308_JX261252_Selar_boops _group 28
DBMF012_HQ560953_Selar_boops _group 28
DBMF127_JX261064_Selar_boops _group 28
DBMF197_JX261461_Selar_boops _group 28
DBMF309_JX261605_Selar_boops _group 28
DBMF751_JX261363_Selar_boops _group 28
DBMF748_JX261297_Selar_boops _group 28
DBMF660_JX261198_Selar_boops _group 28
DBMF351_JX261083_Selar_boops _group 28
DBMF128_JX261523_Selar_boops _group 28
DBMF225_JX261059_Selar_boops _group 28
DBMF-M508_JX261186_Gnathanodon_speciosus _group 23
DBMF-M509_JX261431_Gnathanodon_speciosus _group 23
DBMF-M510_JX261245_Gnathanodon_speciosus _group 23
DBMF-M511_JX261536_Gnathanodon_speciosus _group 23
DBMF-M638|JX261379|Alepes_vari _group 6
DBMF-M286|JX261494|Alepes_vari _group 6
DBMF-M452|JX261192|Alepes_vari _group 6
DBMF-M577|JX261228|Alepes_vari _group 6
DBMF-M454|JX261434|Alepes_vari _group 6
DBMF-M451|JX261475|Alepes_vari _group 6
DBMF-M453|JX261644|Alepes_vari _group 6
DBMF-M284|JX261010|Alepes_vari _group 6
DBMF-M283|JX261520|Alepes_vari _group 6
DBMF-M282_Alepes_vari _group 6
DBMF-M639|JX261282|Alepes_vari _group 6
DBMF-M2|HQ560946|Alepes_vari _group 6
DBMF-M635|JX261234|Alepes_vari _group 6
DBMF-M712|JX261161|Alepes_melanoptera _group 6
DBMF-M711|JX261647|Alepes_melanoptera _group 6
DBMF-M523|JX261457|Alepes_melanoptera _group 6
DBMF-M42|HQ560975|Alepes_melanoptera _group 6
DBMF-M53|HQ560986|Alepes_melanoptera _group 6
DBMF-M16|HQ560956|Alepes_melanoptera _group 6
DBMF-M708|JX261561|Alepes_melanoptera _group 6
DBMF-M524|JX261407|Alepes_melanoptera _group 6
DBMF-M522|JX261624|Alepes_melanoptera _group 6
DBMF-M82|HQ561010|Alepes_melanoptera _group 6
DBMF-M71|HQ561000|Alepes_melanoptera _group 6
DBMF-M769|JX261188|Alepes_melanoptera _group 6
DBMF-M28|HQ560964|Alepes_melanoptera _group 6
DBMF-M767|JX261047|Alepes_melanoptera _group 6
DBMF-M709|JX261267|Alepes_melanoptera _group 6
DBMF-M847|JX261076|Alepes_kleinii _group 5
DBMF-M573|JX261284|Alepes_kleinii _group 5
DBMF-M569|JX261103|Alepes_kleinii _group 5
DBMF-M570|JX261049|Alepes_kleinii _group 5
DBMF-M849|JX261039|Alepes_kleinii _group 5
DBMF-M845|JX261530|Alepes_kleinii _group 5
DBMF-M571|JX261594|Alepes_kleinii _group 5
DBMF-M572|JX261396|Alepes_kleinii _group 5
DBMF-M848|JX261086|Alepes_kleinii _group 5
DBMF-M846|JX261344|Alepes_kleinii _group 5
DBMF-M850|JX261090|Alepes_kleinii _group 5
DBMF-M422_Alepes_djedaba _group 4
DBMF-M359|JX261639|Alepes_djedaba _group 4
DBMF-M358|JX261206|Alepes_djedaba _group 4

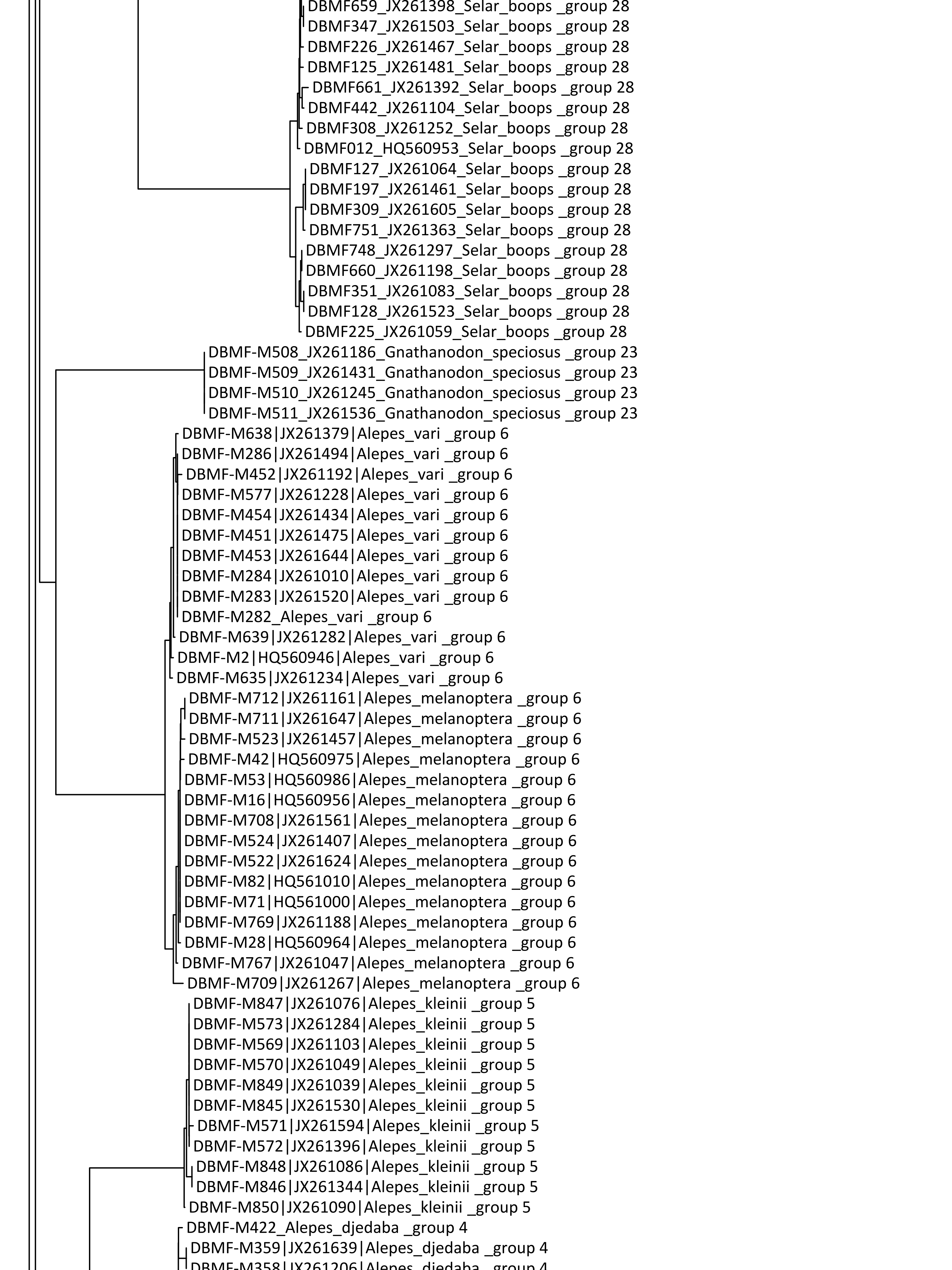
DBMF659_JX261398_Selar_boops _group 28
DBMF347_JX261503_Selar_boops _group 28
DBMF226_JX261467_Selar_boops _group 28
DBMF125_JX261481_Selar_boops _group 28
DBMF661_JX261392_Selar_boops _group 28
DBMF442_JX261104_Selar_boops _group 28
DBMF308_JX261252_Selar_boops _group 28
DBMF012_HQ560953_Selar_boops _group 28
DBMF127_JX261064_Selar_boops _group 28
DBMF197_JX261461_Selar_boops _group 28
DBMF309_JX261605_Selar_boops _group 28
DBMF751_JX261363_Selar_boops _group 28
DBMF748_JX261297_Selar_boops _group 28
DBMF660_JX261198_Selar_boops _group 28
DBMF351_JX261083_Selar_boops _group 28
DBMF128_JX261523_Selar_boops _group 28
DBMF225_JX261059_Selar_boops _group 28
DBMF-M508_JX261186_Gnathanodon_speciosus _group 23
DBMF-M509_JX261431_Gnathanodon_speciosus _group 23
DBMF-M510_JX261245_Gnathanodon_speciosus _group 23
DBMF-M511_JX261536_Gnathanodon_speciosus _group 23
DBMF-M638|JX261379|Alepes_vari _group 6
DBMF-M286|JX261494|Alepes_vari _group 6
DBMF-M452|JX261192|Alepes_vari _group 6
DBMF-M577|JX261228|Alepes_vari _group 6
DBMF-M454|JX261434|Alepes_vari _group 6
DBMF-M451|JX261475|Alepes_vari _group 6
DBMF-M453|JX261644|Alepes_vari _group 6
DBMF-M284|JX261010|Alepes_vari _group 6
DBMF-M283|JX261520|Alepes_vari _group 6
DBMF-M282_Alepes_vari _group 6
DBMF-M639|JX261282|Alepes_vari _group 6
DBMF-M2|HQ560946|Alepes_vari _group 6
DBMF-M635|JX261234|Alepes_vari _group 6
DBMF-M712|JX261161|Alepes_melanoptera _group 6
DBMF-M711|JX261647|Alepes_melanoptera _group 6
DBMF-M523|JX261457|Alepes_melanoptera _group 6
DBMF-M42|HQ560975|Alepes_melanoptera _group 6
DBMF-M53|HQ560986|Alepes_melanoptera _group 6
DBMF-M16|HQ560956|Alepes_melanoptera _group 6
DBMF-M708|JX261561|Alepes_melanoptera _group 6
DBMF-M524|JX261407|Alepes_melanoptera _group 6
DBMF-M522|JX261624|Alepes_melanoptera _group 6
DBMF-M82|HQ561010|Alepes_melanoptera _group 6
DBMF-M71|HQ561000|Alepes_melanoptera _group 6
DBMF-M769|JX261188|Alepes_melanoptera _group 6
DBMF-M28|HQ560964|Alepes_melanoptera _group 6
DBMF-M767|JX261047|Alepes_melanoptera _group 6
DBMF-M709|JX261267|Alepes_melanoptera _group 6
DBMF-M847|JX261076|Alepes_kleinii _group 5
DBMF-M573|JX261284|Alepes_kleinii _group 5
DBMF-M569|JX261103|Alepes_kleinii _group 5
DBMF-M570|JX261049|Alepes_kleinii _group 5
DBMF-M849|JX261039|Alepes_kleinii _group 5
DBMF-M845|JX261530|Alepes_kleinii _group 5
DBMF-M571|JX261594|Alepes_kleinii _group 5
DBMF-M572|JX261396|Alepes_kleinii _group 5
DBMF-M848|JX261086|Alepes_kleinii _group 5
DBMF-M846|JX261344|Alepes_kleinii _group 5
DBMF-M850|JX261090|Alepes_kleinii _group 5
DBMF-M422_Alepes_djedaba _group 4
DBMF-M359|JX261639|Alepes_djedaba _group 4
DBMF-M358|JX261206|Alepes_djedaba _group 4

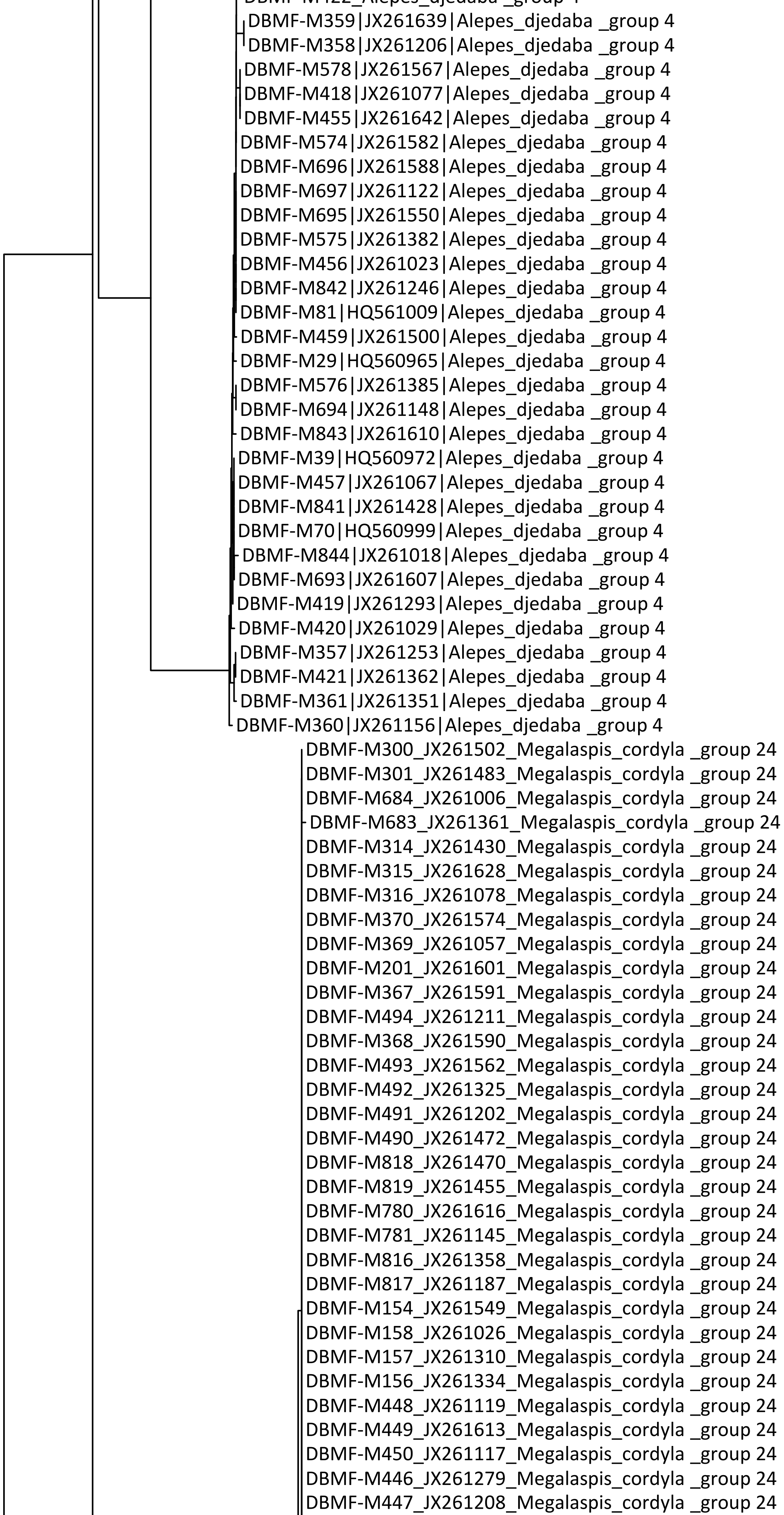

DBMF-M359|JX261639|Alepes_djedaba _group 4
DBMF-M358|JX261206|Alepes_djedaba _group 4
DBMF-M578|JX261567|Alepes_djedaba _group 4
DBMF-M418|JX261077|Alepes_djedaba _group 4
DBMF-M455|JX261642|Alepes_djedaba _group 4
DBMF-M574|JX261582|Alepes_djedaba _group 4
DBMF-M696|JX261588|Alepes_djedaba _group 4
DBMF-M697|JX261122|Alepes_djedaba _group 4
DBMF-M695|JX261550|Alepes_djedaba _group 4
DBMF-M575|JX261382|Alepes_djedaba _group 4
DBMF-M456|JX261023|Alepes_djedaba _group 4
DBMF-M842|JX261246|Alepes_djedaba _group 4
DBMF-M81|HQ561009|Alepes_djedaba _group 4
DBMF-M459|JX261500|Alepes_djedaba _group 4
DBMF-M29|HQ560965|Alepes_djedaba _group 4
DBMF-M576|JX261385|Alepes_djedaba _group 4
DBMF-M694|JX261148|Alepes_djedaba _group 4
DBMF-M843|JX261610|Alepes_djedaba _group 4
DBMF-M39|HQ560972|Alepes_djedaba _group 4
DBMF-M457|JX261067|Alepes_djedaba _group 4
DBMF-M841|JX261428|Alepes_djedaba _group 4
DBMF-M70|HQ560999|Alepes_djedaba _group 4
DBMF-M844|JX261018|Alepes_djedaba _group 4
DBMF-M693|JX261607|Alepes_djedaba _group 4
DBMF-M419|JX261293|Alepes_djedaba _group 4
DBMF-M420|JX261029|Alepes_djedaba _group 4
DBMF-M357|JX261253|Alepes_djedaba _group 4
DBMF-M421|JX261362|Alepes_djedaba _group 4
DBMF-M361|JX261351|Alepes_djedaba _group 4
DBMF-M360|JX261156|Alepes_djedaba _group 4
DBMF-M300_JX261502_Megalaspis_cordyla _group 24
DBMF-M301_JX261483_Megalaspis_cordyla _group 24
DBMF-M684_JX261006_Megalaspis_cordyla _group 24
DBMF-M683_JX261361_Megalaspis_cordyla _group 24
DBMF-M314_JX261430_Megalaspis_cordyla _group 24
DBMF-M315_JX261628_Megalaspis_cordyla _group 24
DBMF-M316_JX261078_Megalaspis_cordyla _group 24
DBMF-M370_JX261574_Megalaspis_cordyla _group 24
DBMF-M369_JX261057_Megalaspis_cordyla _group 24
DBMF-M201_JX261601_Megalaspis_cordyla _group 24
DBMF-M367_JX261591_Megalaspis_cordyla _group 24
DBMF-M494_JX261211_Megalaspis_cordyla _group 24
DBMF-M368_JX261590_Megalaspis_cordyla _group 24
DBMF-M493_JX261562_Megalaspis_cordyla _group 24
DBMF-M492_JX261325_Megalaspis_cordyla _group 24
DBMF-M491_JX261202_Megalaspis_cordyla _group 24
DBMF-M490_JX261472_Megalaspis_cordyla _group 24
DBMF-M818_JX261470_Megalaspis_cordyla _group 24
DBMF-M819_JX261455_Megalaspis_cordyla _group 24
DBMF-M780_JX261616_Megalaspis_cordyla _group 24
DBMF-M781_JX261145_Megalaspis_cordyla _group 24
DBMF-M816_JX261358_Megalaspis_cordyla _group 24
DBMF-M817_JX261187_Megalaspis_cordyla _group 24
DBMF-M154_JX261549_Megalaspis_cordyla _group 24
DBMF-M158_JX261026_Megalaspis_cordyla _group 24
DBMF-M157_JX261310_Megalaspis_cordyla _group 24
DBMF-M156_JX261334_Megalaspis_cordyla _group 24
DBMF-M448_JX261119_Megalaspis_cordyla _group 24
DBMF-M449_JX261613_Megalaspis_cordyla _group 24
DBMF-M450_JX261117_Megalaspis_cordyla _group 24
DBMF-M446_JX261279_Megalaspis_cordyla _group 24
DBMF-M447_JX261208_Megalaspis_cordyla _group 24

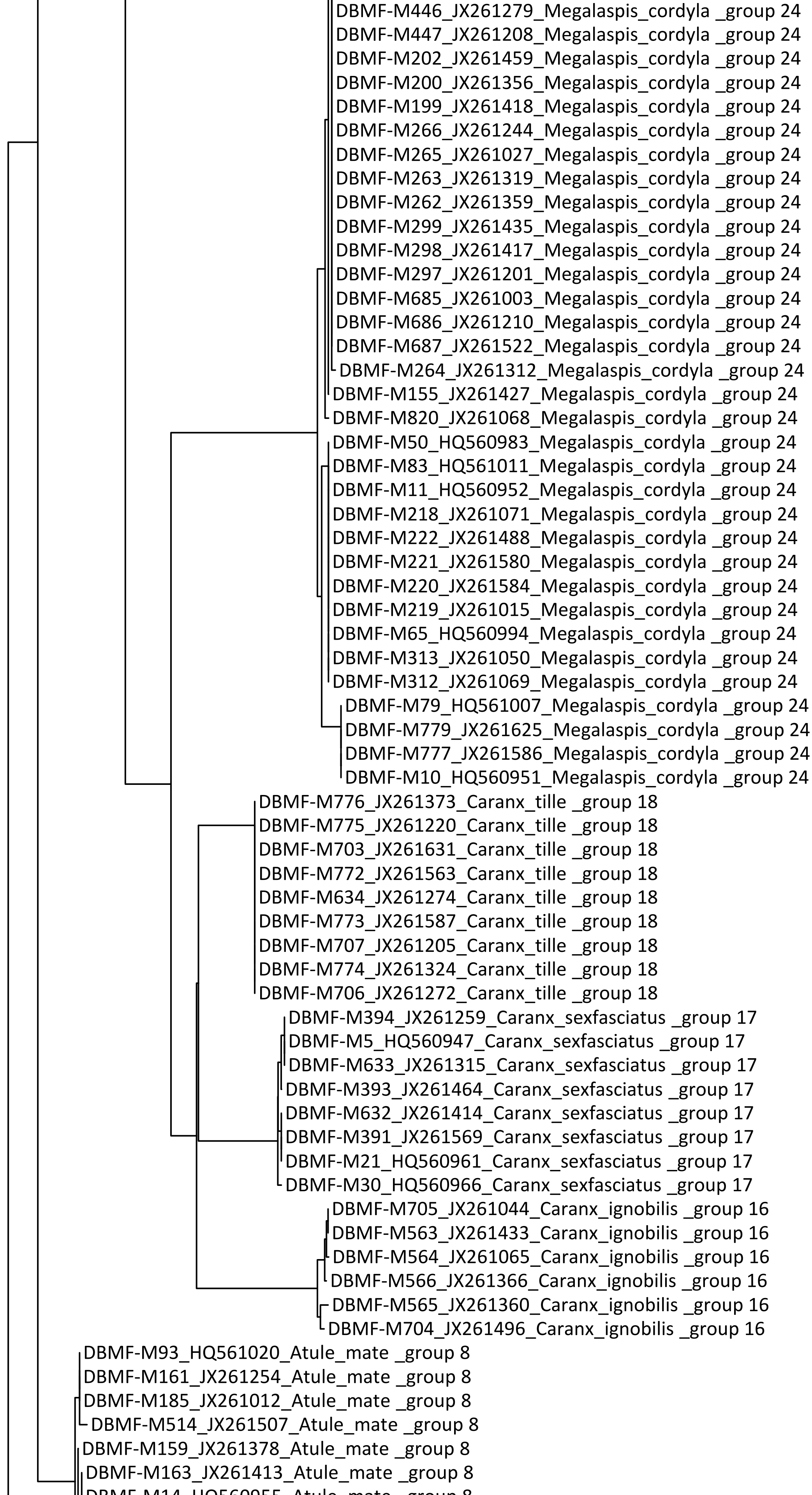
DBMF-M446_JX261279_Megalaspis_cordyla _group 24
DBMF-M447_JX261208_Megalaspis_cordyla _group 24
DBMF-M202_JX261459_Megalaspis_cordyla _group 24
DBMF-M200_JX261356_Megalaspis_cordyla _group 24
DBMF-M199_JX261418_Megalaspis_cordyla _group 24
DBMF-M266_JX261244_Megalaspis_cordyla _group 24
DBMF-M265_JX261027_Megalaspis_cordyla _group 24
DBMF-M263_JX261319_Megalaspis_cordyla _group 24
DBMF-M262_JX261359_Megalaspis_cordyla _group 24
DBMF-M299_JX261435_Megalaspis_cordyla _group 24
DBMF-M298_JX261417_Megalaspis_cordyla _group 24
DBMF-M297_JX261201_Megalaspis_cordyla _group 24
DBMF-M685_JX261003_Megalaspis_cordyla _group 24
DBMF-M686_JX261210_Megalaspis_cordyla _group 24
DBMF-M687_JX261522_Megalaspis_cordyla _group 24
DBMF-M264_JX261312_Megalaspis_cordyla _group 24
DBMF-M155_JX261427_Megalaspis_cordyla _group 24
DBMF-M820_JX261068_Megalaspis_cordyla _group 24
DBMF-M50_HQ560983_Megalaspis_cordyla _group 24
DBMF-M83_HQ561011_Megalaspis_cordyla _group 24
DBMF-M11_HQ560952_Megalaspis_cordyla _group 24
DBMF-M218_JX261071_Megalaspis_cordyla _group 24
DBMF-M222_JX261488_Megalaspis_cordyla _group 24
DBMF-M221_JX261580_Megalaspis_cordyla _group 24
DBMF-M220_JX261584_Megalaspis_cordyla _group 24
DBMF-M219_JX261015_Megalaspis_cordyla _group 24
DBMF-M65_HQ560994_Megalaspis_cordyla _group 24
DBMF-M313_JX261050_Megalaspis_cordyla _group 24
DBMF-M312_JX261069_Megalaspis_cordyla _group 24
DBMF-M79_HQ561007_Megalaspis_cordyla _group 24
DBMF-M779_JX261625_Megalaspis_cordyla _group 24
DBMF-M777_JX261586_Megalaspis_cordyla _group 24
DBMF-M10_HQ560951_Megalaspis_cordyla _group 24
DBMF-M776_JX261373_Caranx_tille _group 18
DBMF-M775_JX261220_Caranx_tille _group 18
DBMF-M703_JX261631_Caranx_tille _group 18
DBMF-M772_JX261563_Caranx_tille _group 18
DBMF-M634_JX261274_Caranx_tille _group 18
DBMF-M773_JX261587_Caranx_tille _group 18
DBMF-M707_JX261205_Caranx_tille _group 18
DBMF-M774_JX261324_Caranx_tille _group 18
DBMF-M706_JX261272_Caranx_tille _group 18
DBMF-M394_JX261259_Caranx_sexfasciatus _group 17
DBMF-M5_HQ560947_Caranx_sexfasciatus _group 17
DBMF-M633_JX261315_Caranx_sexfasciatus _group 17
DBMF-M393_JX261464_Caranx_sexfasciatus _group 17
DBMF-M632_JX261414_Caranx_sexfasciatus _group 17
DBMF-M391_JX261569_Caranx_sexfasciatus _group 17
DBMF-M21_HQ560961_Caranx_sexfasciatus _group 17
DBMF-M30_HQ560966_Caranx_sexfasciatus _group 17
DBMF-M705_JX261044_Caranx_ignobilis _group 16
DBMF-M563_JX261433_Caranx_ignobilis _group 16
DBMF-M564_JX261065_Caranx_ignobilis _group 16
DBMF-M566_JX261366_Caranx_ignobilis _group 16
DBMF-M565_JX261360_Caranx_ignobilis _group 16
DBMF-M704_JX261496_Caranx_ignobilis _group 16
DBMF-M93_HQ561020_Atule_mate _group 8
DBMF-M161_JX261254_Atule_mate _group 8
DBMF-M185_JX261012_Atule_mate _group 8
DBMF-M514_JX261507_Atule_mate _group 8
DBMF-M159_JX261378_Atule_mate _group 8
DBMF-M163_JX261413_Atule_mate _group 8

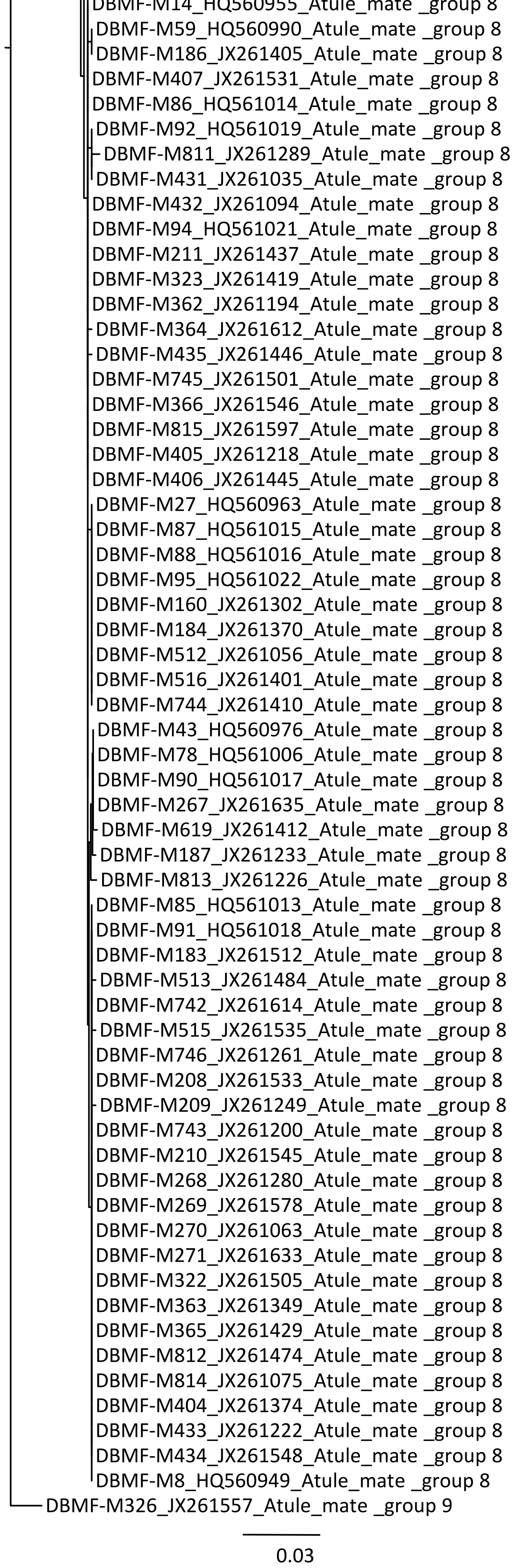
DBMF-M14_HQ560955_Atule_mate _group 8
DBMF-M59_HQ560990_Atule_mate _group 8
DBMF-M186_JX261405_Atule_mate _group 8
DBMF-M407_JX261531_Atule_mate _group 8
DBMF-M86_HQ561014_Atule_mate _group 8
DBMF-M92_HQ561019_Atule_mate _group 8
DBMF-M811_JX261289_Atule_mate _group 8
DBMF-M431_JX261035_Atule_mate _group 8
DBMF-M432_JX261094_Atule_mate _group 8
DBMF-M94_HQ561021_Atule_mate _group 8
DBMF-M211_JX261437_Atule_mate _group 8
DBMF-M323_JX261419_Atule_mate _group 8
DBMF-M362_JX261194_Atule_mate _group 8
DBMF-M364_JX261612_Atule_mate _group 8
DBMF-M435_JX261446_Atule_mate _group 8
DBMF-M745_JX261501_Atule_mate _group 8
DBMF-M366_JX261546_Atule_mate _group 8
DBMF-M815_JX261597_Atule_mate _group 8
DBMF-M405_JX261218_Atule_mate _group 8
DBMF-M406_JX261445_Atule_mate _group 8
DBMF-M27_HQ560963_Atule_mate _group 8
DBMF-M87_HQ561015_Atule_mate _group 8
DBMF-M88_HQ561016_Atule_mate _group 8
DBMF-M95_HQ561022_Atule_mate _group 8
DBMF-M160_JX261302_Atule_mate _group 8
DBMF-M184_JX261370_Atule_mate _group 8
DBMF-M512_JX261056_Atule_mate _group 8
DBMF-M516_JX261401_Atule_mate _group 8
DBMF-M744_JX261410_Atule_mate _group 8
DBMF-M43_HQ560976_Atule_mate _group 8
DBMF-M78_HQ561006_Atule_mate _group 8
DBMF-M90_HQ561017_Atule_mate _group 8
DBMF-M267_JX261635_Atule_mate _group 8
DBMF-M619_JX261412_Atule_mate _group 8
DBMF-M187_JX261233_Atule_mate _group 8
DBMF-M813_JX261226_Atule_mate _group 8
DBMF-M85_HQ561013_Atule_mate _group 8
DBMF-M91_HQ561018_Atule_mate _group 8
DBMF-M183_JX261512_Atule_mate _group 8
DBMF-M513_JX261484_Atule_mate _group 8
DBMF-M742_JX261614_Atule_mate _group 8
DBMF-M515_JX261535_Atule_mate _group 8
DBMF-M746_JX261261_Atule_mate _group 8
DBMF-M208_JX261533_Atule_mate _group 8
DBMF-M209_JX261249_Atule_mate _group 8
DBMF-M743_JX261200_Atule_mate _group 8
DBMF-M210_JX261545_Atule_mate _group 8
DBMF-M268_JX261280_Atule_mate _group 8
DBMF-M269_JX261578_Atule_mate _group 8
DBMF-M270_JX261063_Atule_mate _group 8
DBMF-M271_JX261633_Atule_mate _group 8
DBMF-M322_JX261505_Atule_mate _group 8
DBMF-M363_JX261349_Atule_mate _group 8
DBMF-M365_JX261429_Atule_mate _group 8
DBMF-M812_JX261474_Atule_mate _group 8
DBMF-M814_JX261075_Atule_mate _group 8
DBMF-M404_JX261374_Atule_mate _group 8
DBMF-M433_JX261222_Atule_mate _group 8
DBMF-M434_JX261548_Atule_mate _group 8
DBMF-M8_HQ560949_Atule_mate _group 8
DBMF-M326_JX261557_Atule_mate _group 9
0.03
