# Supplementary material for: DNA Barcoding Reveals Cryptic Diversity within Commercially Exploited Indo-Malay Carangidae (Teleosteii: Perciformes)
Source: PLoS One. 2012 Nov 29;7(11):e49623. doi: 10.1371/journal.pone.0049623 (PMC3510217; doi:10.1371/journal.pone.0049623)
Supplement: Figure S4 — Taxon ID Tree of 23 widespread Carangidae species generated by MEGA5 including conspecifics from other geographical regions. Neighbour-joining tree (Kimura 2-parameter, pairwise deletion). (PDF) [file pone.0049623.s004.pdf]

**Figure S5. Taxon ID Tree of 23 widespread Carangidae species generated by MEGA5 including conspecifics from other geographical regions.** Neighbour-joining tree (Kimura 2-parameter, pairwise deletion)

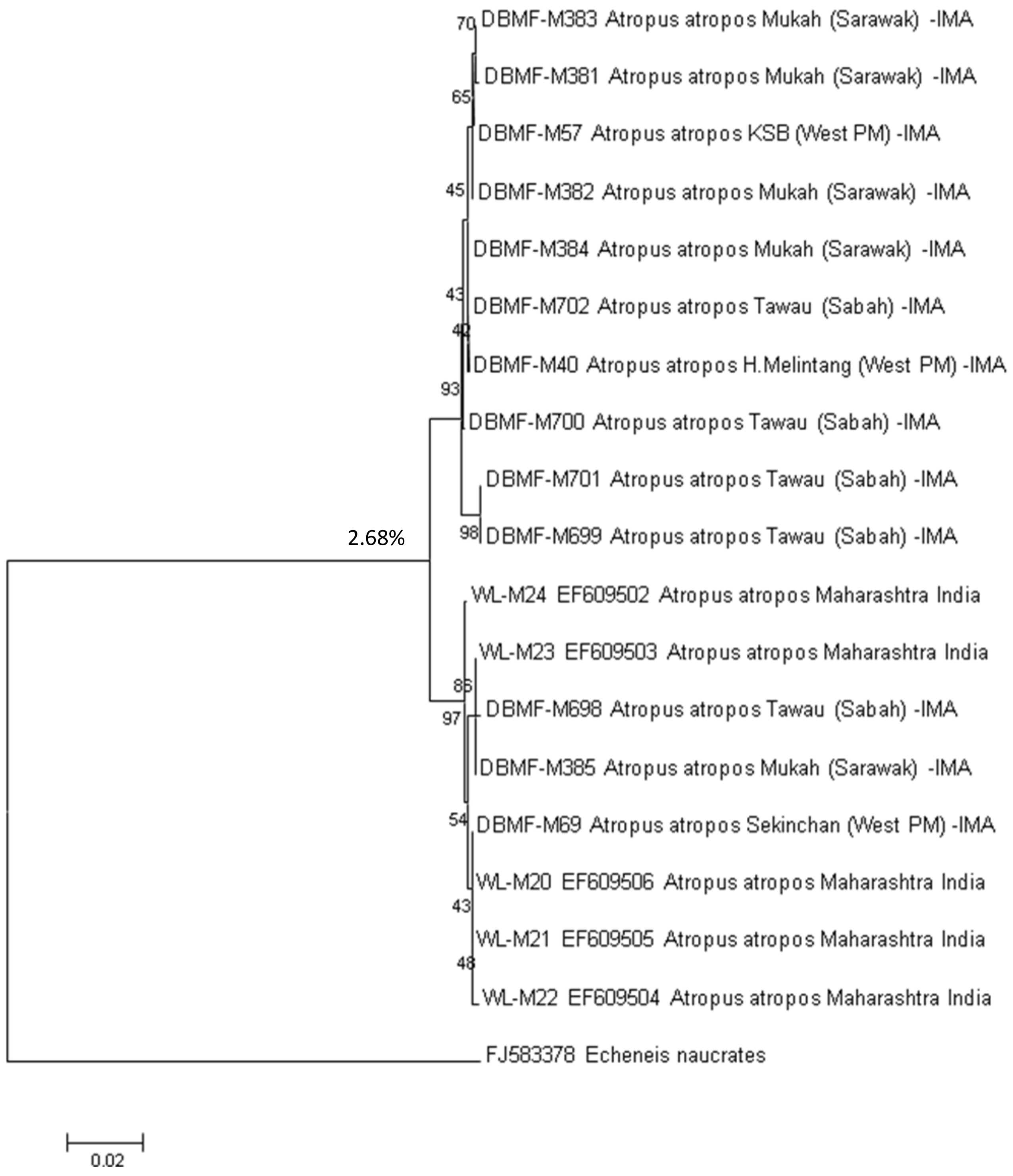

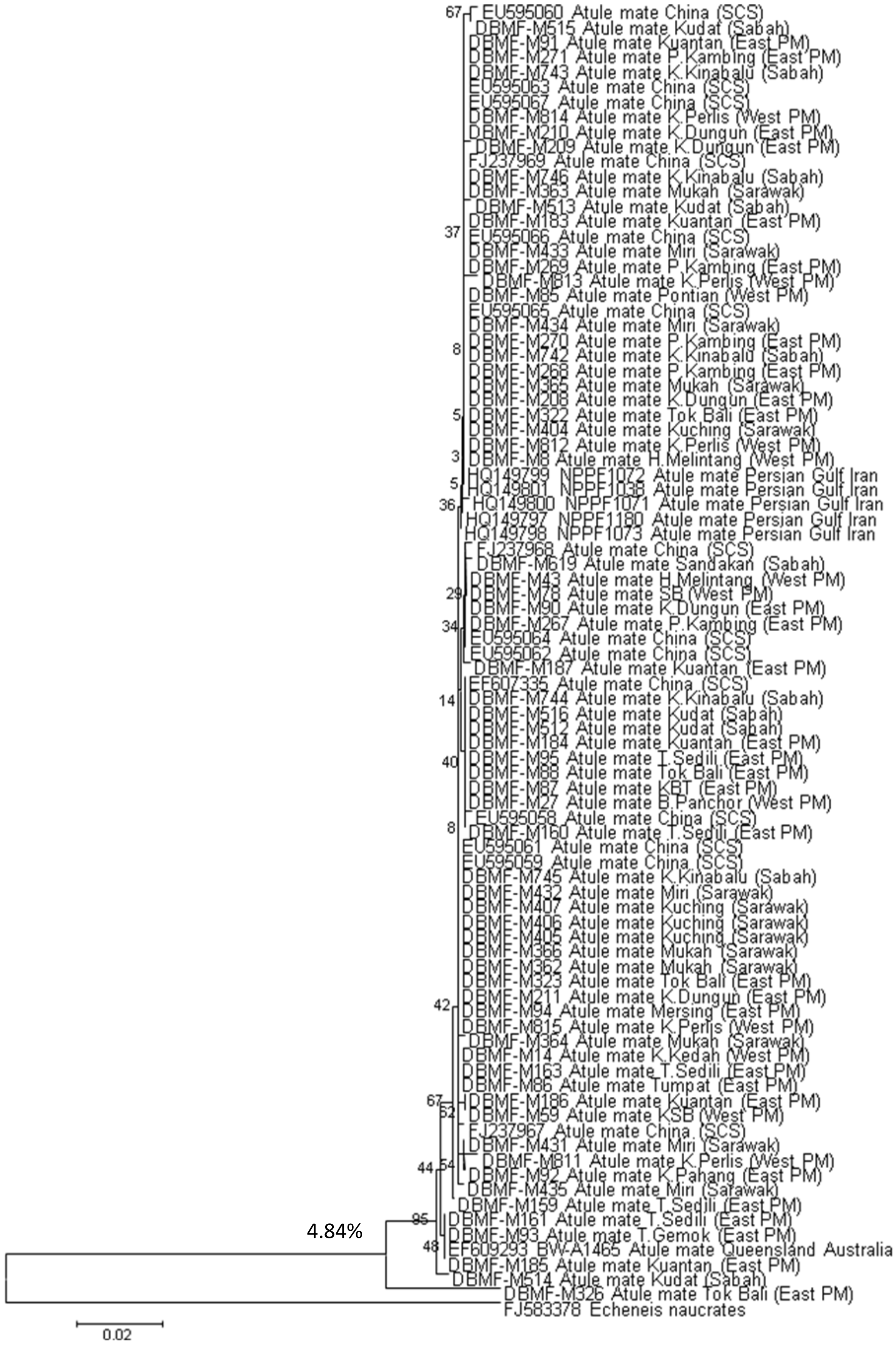
EU595060 Atule mate China (SCS)
DBMF-M515 Atule mate Kudat (Sabah)
DBMF-M91 Atule mate Kuantan (East PM)
DBMF-M271 Atule mate P.Kambing (East PM)
DBMF-M743 Atule mate K.Kinabalu (Sabah)
EU595063 Atule mate China (SCS)
EU595067 Atule mate China (SCS)
DBMF-M814 Atule mate K.Perlis (West PM)
DBMF-M210 Atule mate K.Dungun (East PM)
DBMF-M209 Atule mate K.Dungun (East PM)
FJ237969 Atule mate China (SCS)
DBMF-M746 Atule mate K.Kinabalu (Sabah)
DBMF-M363 Atule mate Mukah (Sarawak)
DBMF-M513 Atule mate Kudat (Sabah)
DBMF-M183 Atule mate Kuantan (East PM)
EU595066 Atule mate China (SCS)
DBMF-M433 Atule mate Miri (Sarawak)
DBMF-M269 Atule mate P.Kambing (East PM)
DBMF-M813 Atule mate K.Perlis (West PM)
DBMF-M85 Atule mate Pontian (West PM)
EU595065 Atule mate China (SCS)
DBMF-M434 Atule mate Miri (Sarawak)
DBMF-M270 Atule mate P.Kambing (East PM)
DBMF-M742 Atule mate K.Kinabalu (Sabah)
DBMF-M268 Atule mate P.Kambing (East PM)
DBMF-M365 Atule mate Mukah (Sarawak)
DBMF-M208 Atule mate K.Dungun (East PM)
DBMF-M322 Atule mate Tok Bali (East PM)
DBMF-M404 Atule mate Kuching (Sarawak)
DBMF-M812 Atule mate K.Perlis (West PM)
DBMF-M8 Atule mate H.Melintang (West PM)
HQ149799 NPPF1072 Atule mate Persian Gulf Iran
HQ149801 NPPF1038 Atule mate Persian Gulf Iran
HQ149800 NPPF1071 Atule mate Persian Gulf Iran
HQ149797 NPPF1180 Atule mate Persian Gulf Iran
HQ149798 NPPF1073 Atule mate Persian Gulf Iran
FJ237968 Atule mate China (SCS)
DBMF-M619 Atule mate Sandakan (Sabah)
DBMF-M43 Atule mate H.Melintang (West PM)
DBMF-M78 Atule mate SB (West PM)
DBMF-M90 Atule mate K.Dungun (East PM)
DBMF-M267 Atule mate P.Kambing (East PM)
EU595064 Atule mate China (SCS)
EU595062 Atule mate China (SCS)
DBMF-M187 Atule mate Kuantan (East PM)
EF607335 Atule mate China (SCS)
DBMF-M744 Atule mate K.Kinabalu (Sabah)
DBMF-M516 Atule mate Kudat (Sabah)
DBMF-M512 Atule mate Kudat (Sabah)
DBMF-M184 Atule mate Kuantan (East PM)
DBMF-M95 Atule mate T.Sedili (East PM)
DBMF-M88 Atule mate Tok Bali (East PM)
DBMF-M87 Atule mate KBT (East PM)
DBMF-M27 Atule mate B.Panchor (West PM)
EU595058 Atule mate China (SCS)
DBMF-M160 Atule mate T.Sedili (East PM)
EU595061 Atule mate China (SCS)
EU595059 Atule mate China (SCS)
DBMF-M745 Atule mate K.Kinabalu (Sabah)
DBMF-M432 Atule mate Miri (Sarawak)
DBMF-M407 Atule mate Kuching (Sarawak)
DBMF-M406 Atule mate Kuching (Sarawak)
DBMF-M405 Atule mate Kuching (Sarawak)
DBMF-M366 Atule mate Mukah (Sarawak)
DBMF-M362 Atule mate Mukah (Sarawak)
DBMF-M323 Atule mate Tok Bali (East PM)
DBMF-M211 Atule mate K.Dungun (East PM)
DBMF-M94 Atule mate Mersing (East PM)
DBMF-M815 Atule mate K.Perlis (West PM)
DBMF-M364 Atule mate Mukah (Sarawak)
DBMF-M14 Atule mate K.Kedah (West PM)
DBMF-M163 Atule mate T.Sedili (East PM)
DBMF-M86 Atule mate Tumpat (East PM)
DBMF-M186 Atule mate Kuantan (East PM)
DBMF-M59 Atule mate KSB (West PM)
FJ237967 Atule mate China (SCS)
DBMF-M431 Atule mate Miri (Sarawak)
DBMF-M811 Atule mate K.Perlis (West PM)
DBMF-M92 Atule mate K.Pahang (East PM)
DBMF-M435 Atule mate Miri (Sarawak)
DBMF-M159 Atule mate T.Sedili (East PM)
DBMF-M161 Atule mate T.Sedili (East PM)
DBMF-M93 Atule mate T.Gemok (East PM)
EF609293 BW-A1465 Atule mate Queensland Australia
DBMF-M185 Atule mate Kuantan (East PM)
DBMF-M514 Atule mate Kudat (Sabah)
DBMF-M326 Atule mate Tok Bali (East PM)
FJ583378 Echeneis naucrates
67
37
8
5
3
5
36
29
34
14
40
8
42
67
52
44
54
95
48
4.84%
0.02

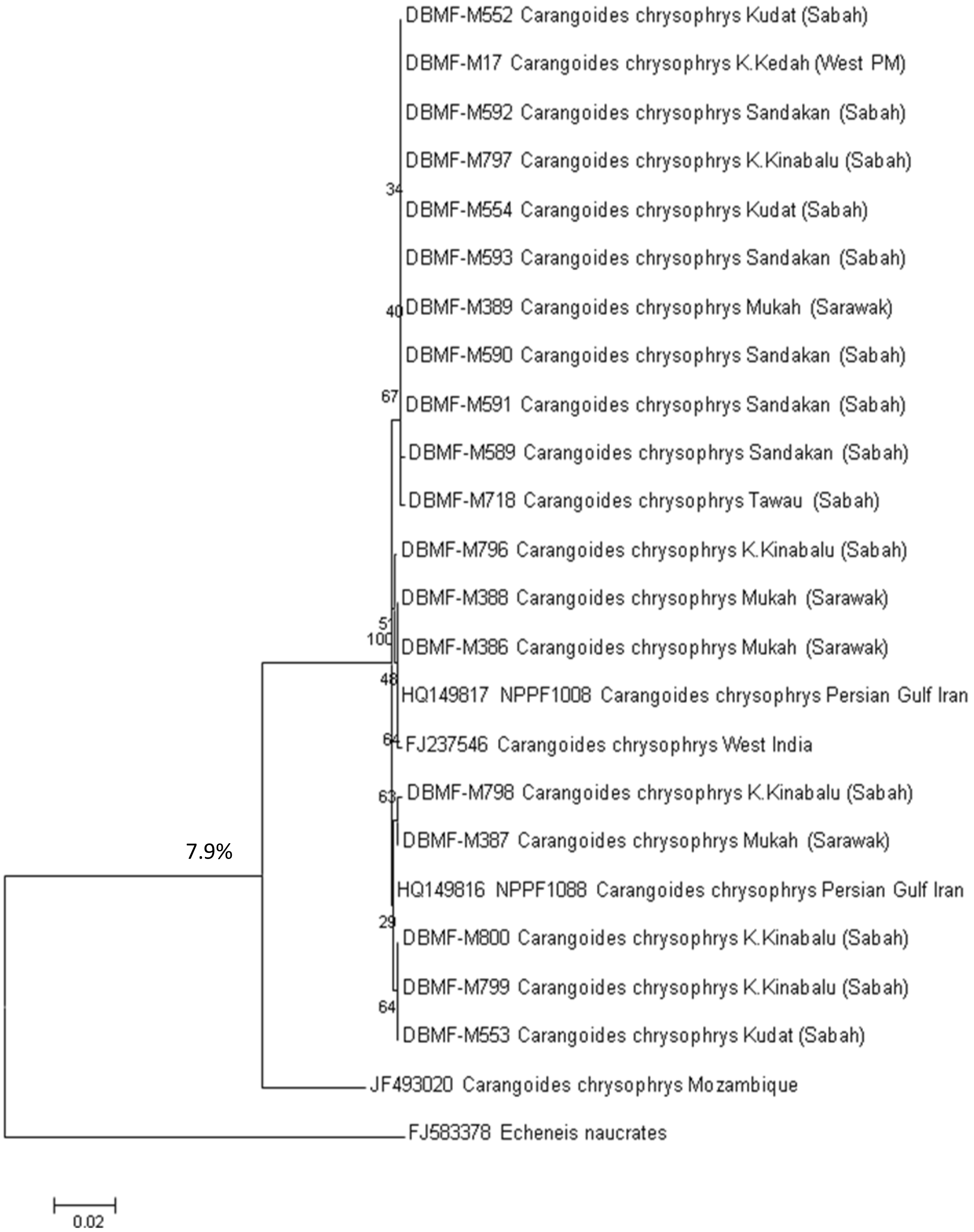

DBMF-M552 Carangoides chrysophrys Kudat (Sabah)
DBMF-M17 Carangoides chrysophrys K.Kedah (West PM)
DBMF-M592 Carangoides chrysophrys Sandakan (Sabah)
DBMF-M797 Carangoides chrysophrys K.Kinabalu (Sabah)
34
DBMF-M554 Carangoides chrysophrys Kudat (Sabah)
DBMF-M593 Carangoides chrysophrys Sandakan (Sabah)
40
DBMF-M389 Carangoides chrysophrys Mukah (Sarawak)
DBMF-M590 Carangoides chrysophrys Sandakan (Sabah)
67
DBMF-M591 Carangoides chrysophrys Sandakan (Sabah)
DBMF-M589 Carangoides chrysophrys Sandakan (Sabah)
DBMF-M718 Carangoides chrysophrys Tawau (Sabah)
DBMF-M796 Carangoides chrysophrys K.Kinabalu (Sabah)
DBMF-M388 Carangoides chrysophrys Mukah (Sarawak)
51
100
DBMF-M386 Carangoides chrysophrys Mukah (Sarawak)
48
HQ149817 NPPF1008 Carangoides chrysophrys Persian Gulf Iran
64
FJ237546 Carangoides chrysophrys West India
63
DBMF-M798 Carangoides chrysophrys K.Kinabalu (Sabah)
7.9%
DBMF-M387 Carangoides chrysophrys Mukah (Sarawak)
HQ149816 NPPF1088 Carangoides chrysophrys Persian Gulf Iran
29
DBMF-M800 Carangoides chrysophrys K.Kinabalu (Sabah)
DBMF-M799 Carangoides chrysophrys K.Kinabalu (Sabah)
64
DBMF-M553 Carangoides chrysophrys Kudat (Sabah)
JF493020 Carangoides chrysophrys Mozambique
FJ583378 Echeneis naucrates
0.02

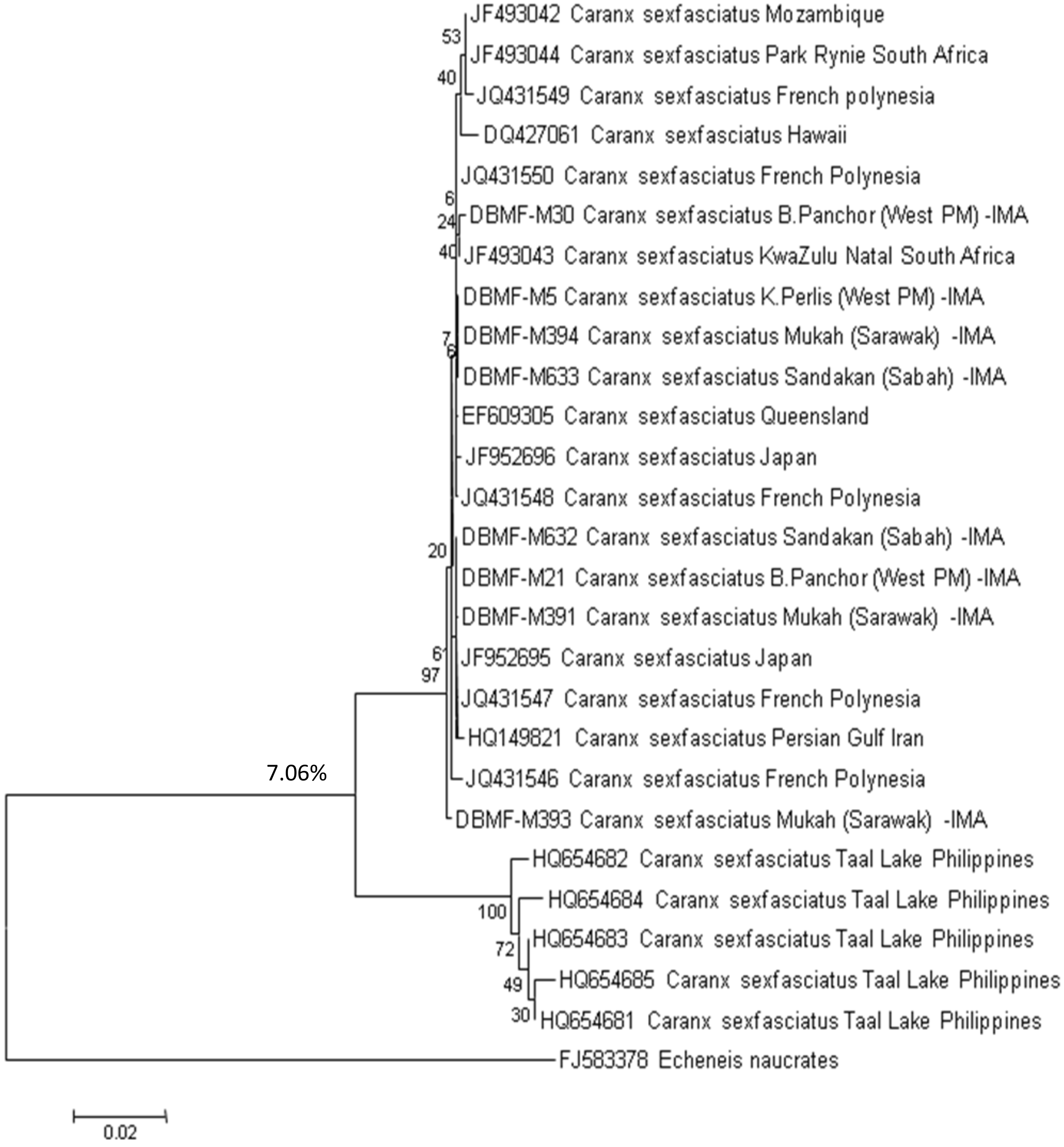
JF493042 Caranx sexfasciatus Mozambique
JF493044 Caranx sexfasciatus Park Rynie South Africa
JQ431549 Caranx sexfasciatus French polynesia
DQ427061 Caranx sexfasciatus Hawaii
JQ431550 Caranx sexfasciatus French Polynesia
DBMF-M30 Caranx sexfasciatus B.Panchor (West PM) -IMA
JF493043 Caranx sexfasciatus KwaZulu Natal South Africa
DBMF-M5 Caranx sexfasciatus K.Perlis (West PM) -IMA
DBMF-M394 Caranx sexfasciatus Mukah (Sarawak) -IMA
DBMF-M633 Caranx sexfasciatus Sandakan (Sabah) -IMA
EF609305 Caranx sexfasciatus Queensland
JF952696 Caranx sexfasciatus Japan
JQ431548 Caranx sexfasciatus French Polynesia
DBMF-M632 Caranx sexfasciatus Sandakan (Sabah) -IMA
DBMF-M21 Caranx sexfasciatus B.Panchor (West PM) -IMA
DBMF-M391 Caranx sexfasciatus Mukah (Sarawak) -IMA
JF952695 Caranx sexfasciatus Japan
JQ431547 Caranx sexfasciatus French Polynesia
HQ149821 Caranx sexfasciatus Persian Gulf Iran
JQ431546 Caranx sexfasciatus French Polynesia
DBMF-M393 Caranx sexfasciatus Mukah (Sarawak) -IMA
HQ654682 Caranx sexfasciatus Taal Lake Philippines
HQ654684 Caranx sexfasciatus Taal Lake Philippines
HQ654683 Caranx sexfasciatus Taal Lake Philippines
HQ654685 Caranx sexfasciatus Taal Lake Philippines
HQ654681 Caranx sexfasciatus Taal Lake Philippines
FJ583378 Echeneis naucrates
53
40
6
24
40
7
6
20
61
97
7.06%
100
72
49
30
0.02

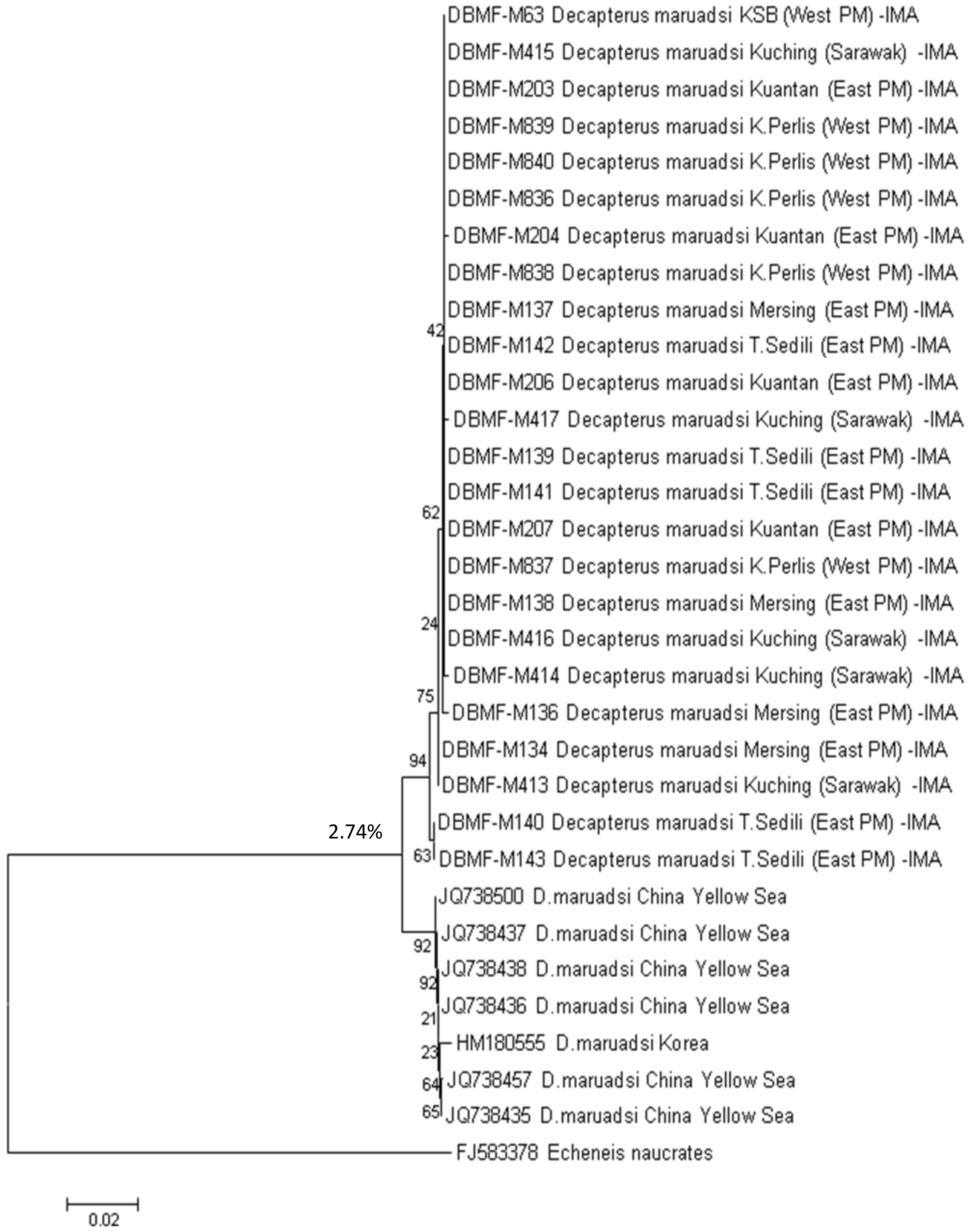

DBMF-M63 Decapterus maruadsi KSB (West PM) -IMA
DBMF-M415 Decapterus maruadsi Kuching (Sarawak) -IMA
DBMF-M203 Decapterus maruadsi Kuantan (East PM) -IMA
DBMF-M839 Decapterus maruadsi K.Perlis (West PM) -IMA
DBMF-M840 Decapterus maruadsi K.Perlis (West PM) -IMA
DBMF-M836 Decapterus maruadsi K.Perlis (West PM) -IMA
DBMF-M204 Decapterus maruadsi Kuantan (East PM) -IMA
DBMF-M838 Decapterus maruadsi K.Perlis (West PM) -IMA
DBMF-M137 Decapterus maruadsi Mersing (East PM) -IMA
42
DBMF-M142 Decapterus maruadsi T.Sedili (East PM) -IMA
DBMF-M206 Decapterus maruadsi Kuantan (East PM) -IMA
DBMF-M417 Decapterus maruadsi Kuching (Sarawak) -IMA
DBMF-M139 Decapterus maruadsi T.Sedili (East PM) -IMA
DBMF-M141 Decapterus maruadsi T.Sedili (East PM) -IMA
62
DBMF-M207 Decapterus maruadsi Kuantan (East PM) -IMA
DBMF-M837 Decapterus maruadsi K.Perlis (West PM) -IMA
DBMF-M138 Decapterus maruadsi Mersing (East PM) -IMA
24
DBMF-M416 Decapterus maruadsi Kuching (Sarawak) -IMA
DBMF-M414 Decapterus maruadsi Kuching (Sarawak) -IMA
75
DBMF-M136 Decapterus maruadsi Mersing (East PM) -IMA
DBMF-M134 Decapterus maruadsi Mersing (East PM) -IMA
94
DBMF-M413 Decapterus maruadsi Kuching (Sarawak) -IMA
2.74%
DBMF-M140 Decapterus maruadsi T.Sedili (East PM) -IMA
63
DBMF-M143 Decapterus maruadsi T.Sedili (East PM) -IMA
JQ738500 D.maruadsi China Yellow Sea
JQ738437 D.maruadsi China Yellow Sea
92
JQ738438 D.maruadsi China Yellow Sea
92
JQ738436 D.maruadsi China Yellow Sea
21
HM180555 D.maruadsi Korea
23
JQ738457 D.maruadsi China Yellow Sea
64
JQ738435 D.maruadsi China Yellow Sea
65
FJ583378 Echeneis naucrates
0.02

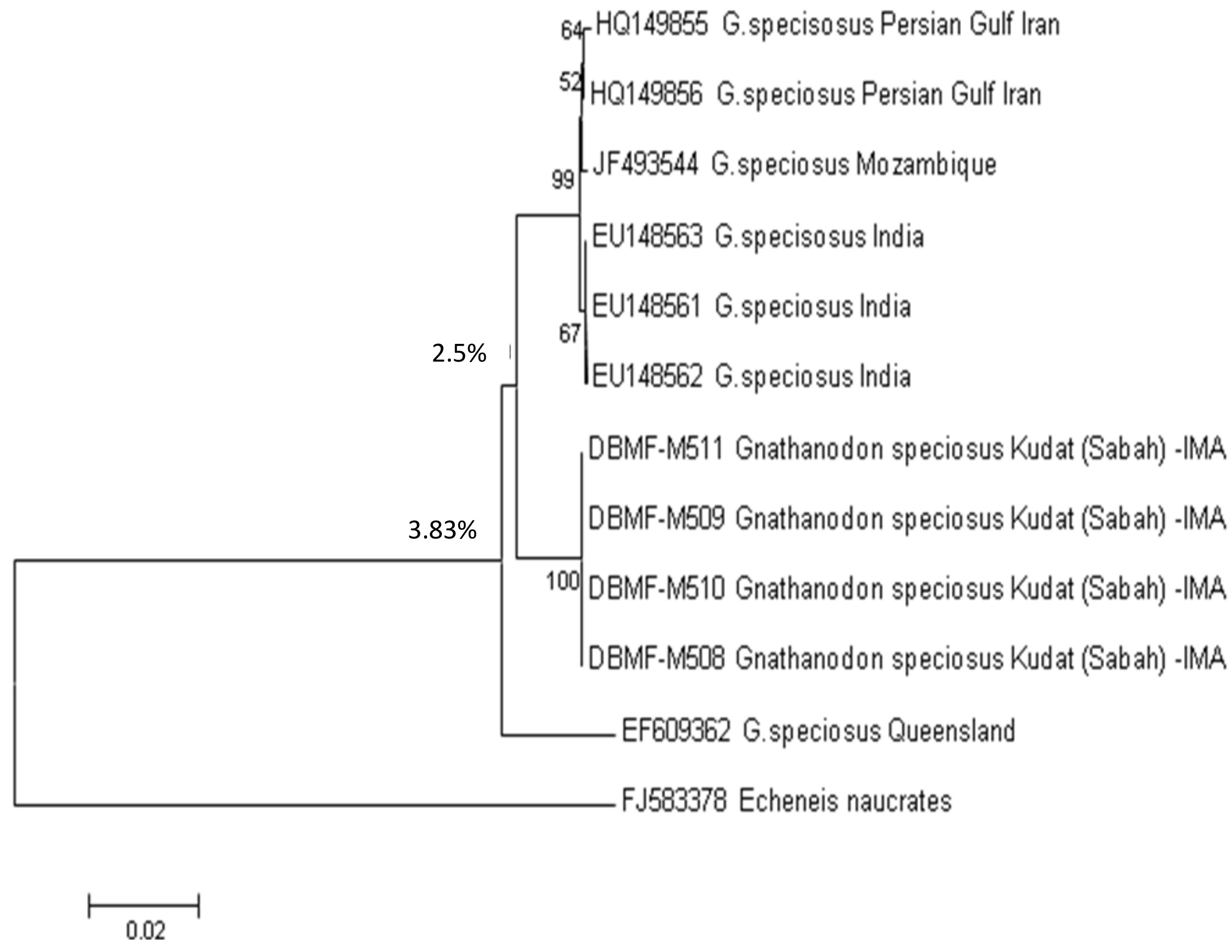
HQ149855 G.specisosus Persian Gulf Iran
HQ149856 G.speciosus Persian Gulf Iran
JF493544 G.speciosus Mozambique
EU148563 G.specisosus India
EU148561 G.speciosus India
EU148562 G.speciosus India
DBMF-M511 Gnathanodon speciosus Kudat (Sabah) -IMA
DBMF-M509 Gnathanodon speciosus Kudat (Sabah) -IMA
DBMF-M510 Gnathanodon speciosus Kudat (Sabah) -IMA
DBMF-M508 Gnathanodon speciosus Kudat (Sabah) -IMA
EF609362 G.speciosus Queensland
FJ583378 Echeneis naucrates
64
52
99
67
100
2.5%
3.83%
0.02

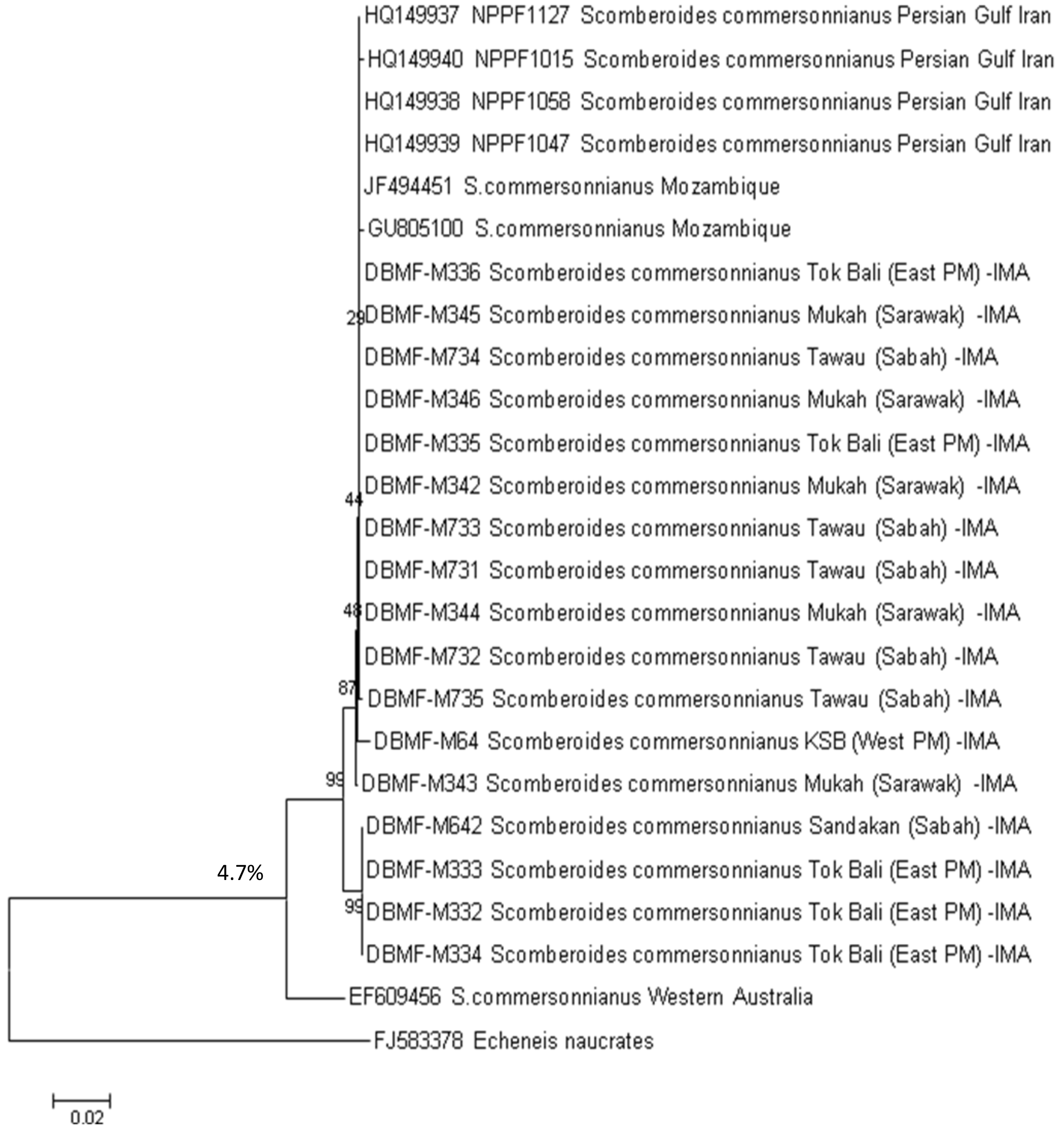

HQ149937 NPPF1127 Scomberoides commersonnianus Persian Gulf Iran
HQ149940 NPPF1015 Scomberoides commersonnianus Persian Gulf Iran
HQ149938 NPPF1058 Scomberoides commersonnianus Persian Gulf Iran
HQ149939 NPPF1047 Scomberoides commersonnianus Persian Gulf Iran
JF494451 S.commersonnianus Mozambique
GU805100 S.commersonnianus Mozambique
DBMF-M336 Scomberoides commersonnianus Tok Bali (East PM) -IMA
DBMF-M345 Scomberoides commersonnianus Mukah (Sarawak) -IMA
DBMF-M734 Scomberoides commersonnianus Tawau (Sabah) -IMA
DBMF-M346 Scomberoides commersonnianus Mukah (Sarawak) -IMA
DBMF-M335 Scomberoides commersonnianus Tok Bali (East PM) -IMA
DBMF-M342 Scomberoides commersonnianus Mukah (Sarawak) -IMA
DBMF-M733 Scomberoides commersonnianus Tawau (Sabah) -IMA
DBMF-M731 Scomberoides commersonnianus Tawau (Sabah) -IMA
DBMF-M344 Scomberoides commersonnianus Mukah (Sarawak) -IMA
DBMF-M732 Scomberoides commersonnianus Tawau (Sabah) -IMA
DBMF-M735 Scomberoides commersonnianus Tawau (Sabah) -IMA
DBMF-M64 Scomberoides commersonnianus KSB (West PM) -IMA
DBMF-M343 Scomberoides commersonnianus Mukah (Sarawak) -IMA
DBMF-M642 Scomberoides commersonnianus Sandakan (Sabah) -IMA
DBMF-M333 Scomberoides commersonnianus Tok Bali (East PM) -IMA
DBMF-M332 Scomberoides commersonnianus Tok Bali (East PM) -IMA
DBMF-M334 Scomberoides commersonnianus Tok Bali (East PM) -IMA
EF609456 S.commersonnianus Western Australia
FJ583378 Echeneis naucrates
4.7%
0.02

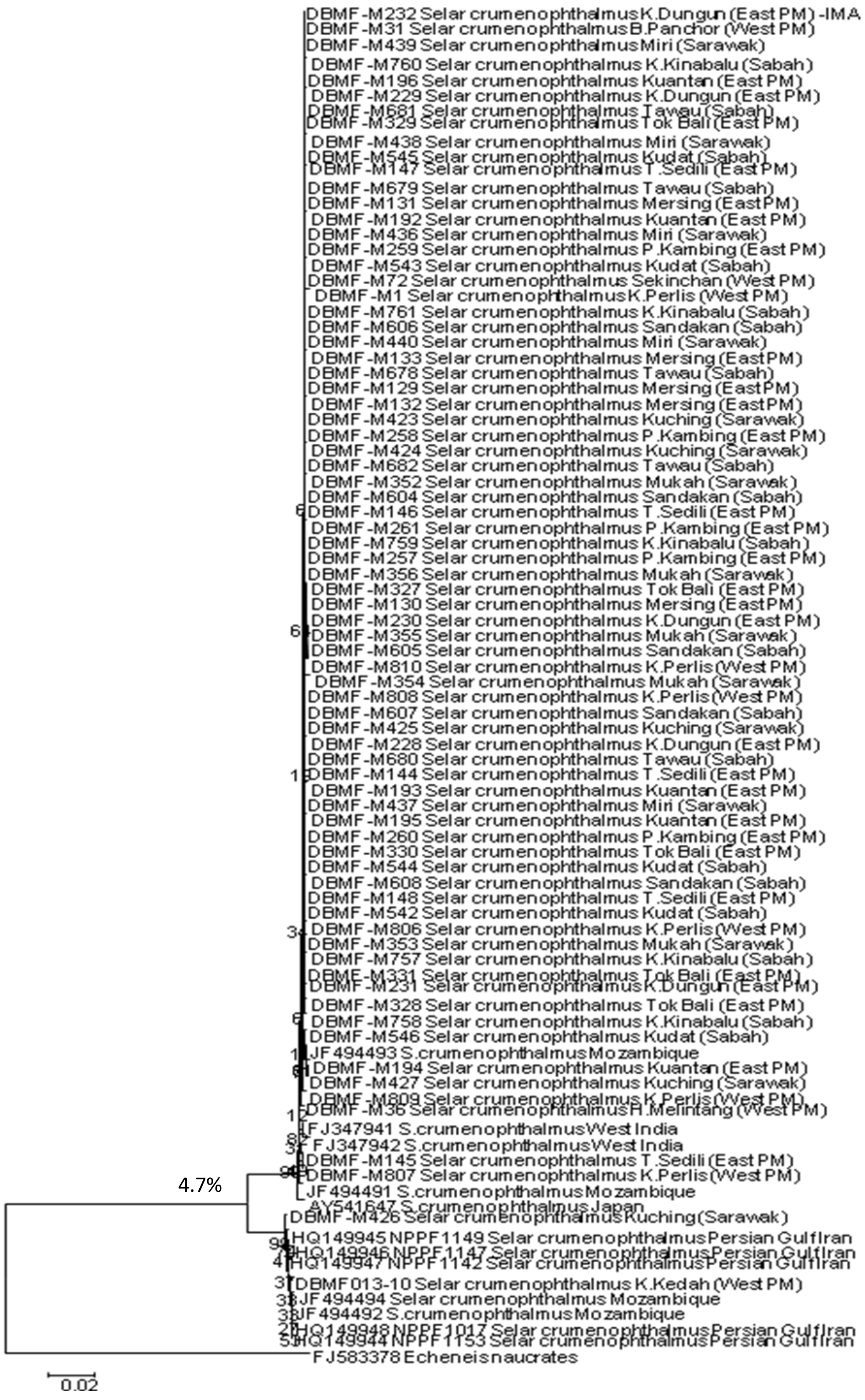
DBMF-M232 Selar crumenophthalmus K.Dungun (East PM) -IMA
DBMF-M31 Selar crumenophthalmus B.Panchor (West PM)
DBMF-M439 Selar crumenophthalmus Miri (Sarawak)
DBMF-M760 Selar crumenophthalmus K.Kinabalu (Sabah)
DBMF-M196 Selar crumenophthalmus Kuantan (East PM)
DBMF-M229 Selar crumenophthalmus K.Dungun (East PM)
DBMF-M681 Selar crumenophthalmus Tawau (Sabah)
DBMF-M329 Selar crumenophthalmus Tok Bali (East PM)
DBMF-M438 Selar crumenophthalmus Miri (Sarawak)
DBMF-M545 Selar crumenophthalmus Kudat (Sabah)
DBMF-M147 Selar crumenophthalmus T.Sedili (East PM)
DBMF-M679 Selar crumenophthalmus Tawau (Sabah)
DBMF-M131 Selar crumenophthalmus Mersing (East PM)
DBMF-M192 Selar crumenophthalmus Kuantan (East PM)
DBMF-M436 Selar crumenophthalmus Miri (Sarawak)
DBMF-M259 Selar crumenophthalmus P.Kambing (East PM)
DBMF-M543 Selar crumenophthalmus Kudat (Sabah)
DBMF-M72 Selar crumenophthalmus Sekinchan (West PM)
DBMF-M1 Selar crumenophthalmus K.Perlis (West PM)
DBMF-M761 Selar crumenophthalmus K.Kinabalu (Sabah)
DBMF-M606 Selar crumenophthalmus Sandakan (Sabah)
DBMF-M440 Selar crumenophthalmus Miri (Sarawak)
DBMF-M133 Selar crumenophthalmus Mersing (East PM)
DBMF-M678 Selar crumenophthalmus Tawau (Sabah)
DBMF-M129 Selar crumenophthalmus Mersing (East PM)
DBMF-M132 Selar crumenophthalmus Mersing (East PM)
DBMF-M423 Selar crumenophthalmus Kuching (Sarawak)
DBMF-M258 Selar crumenophthalmus P.Kambing (East PM)
DBMF-M424 Selar crumenophthalmus Kuching (Sarawak)
DBMF-M682 Selar crumenophthalmus Tawau (Sabah)
DBMF-M352 Selar crumenophthalmus Mukah (Sarawak)
DBMF-M604 Selar crumenophthalmus Sandakan (Sabah)
DBMF-M146 Selar crumenophthalmus T.Sedili (East PM)
DBMF-M261 Selar crumenophthalmus P.Kambing (East PM)
DBMF-M759 Selar crumenophthalmus K.Kinabalu (Sabah)
DBMF-M257 Selar crumenophthalmus P.Kambing (East PM)
DBMF-M356 Selar crumenophthalmus Mukah (Sarawak)
DBMF-M327 Selar crumenophthalmus Tok Bali (East PM)
DBMF-M130 Selar crumenophthalmus Mersing (East PM)
DBMF-M230 Selar crumenophthalmus K.Dungun (East PM)
DBMF-M355 Selar crumenophthalmus Mukah (Sarawak)
DBMF-M605 Selar crumenophthalmus Sandakan (Sabah)
DBMF-M810 Selar crumenophthalmus K.Perlis (West PM)
DBMF-M354 Selar crumenophthalmus Mukah (Sarawak)
DBMF-M808 Selar crumenophthalmus K.Perlis (West PM)
DBMF-M607 Selar crumenophthalmus Sandakan (Sabah)
DBMF-M425 Selar crumenophthalmus Kuching (Sarawak)
DBMF-M228 Selar crumenophthalmus K.Dungun (East PM)
DBMF-M680 Selar crumenophthalmus Tawau (Sabah)
DBMF-M144 Selar crumenophthalmus T.Sedili (East PM)
DBMF-M193 Selar crumenophthalmus Kuantan (East PM)
DBMF-M437 Selar crumenophthalmus Miri (Sarawak)
DBMF-M195 Selar crumenophthalmus Kuantan (East PM)
DBMF-M260 Selar crumenophthalmus P.Kambing (East PM)
DBMF-M330 Selar crumenophthalmus Tok Bali (East PM)
DBMF-M544 Selar crumenophthalmus Kudat (Sabah)
DBMF-M608 Selar crumenophthalmus Sandakan (Sabah)
DBMF-M148 Selar crumenophthalmus T.Sedili (East PM)
DBMF-M542 Selar crumenophthalmus Kudat (Sabah)
DBMF-M806 Selar crumenophthalmus K.Perlis (West PM)
DBMF-M353 Selar crumenophthalmus Mukah (Sarawak)
DBMF-M757 Selar crumenophthalmus K.Kinabalu (Sabah)
DBMF-M331 Selar crumenophthalmus Tok Bali (East PM)
DBMF-M231 Selar crumenophthalmus K.Dungun (East PM)
DBMF-M328 Selar crumenophthalmus Tok Bali (East PM)
DBMF-M758 Selar crumenophthalmus K.Kinabalu (Sabah)
DBMF-M546 Selar crumenophthalmus Kudat (Sabah)
JF494493 S.crumenophthalmus Mozambique
DBMF-M194 Selar crumenophthalmus Kuantan (East PM)
DBMF-M427 Selar crumenophthalmus Kuching (Sarawak)
DBMF-M809 Selar crumenophthalmus K.Perlis (West PM)
DBMF-M36 Selar crumenophthalmus H.Melintang (West PM)
FJ347941 S.crumenophthalmus West India
FJ347942 S.crumenophthalmus West India
DBMF-M145 Selar crumenophthalmus T.Sedili (East PM)
DBMF-M807 Selar crumenophthalmus K.Perlis (West PM)
JF494491 S.crumenophthalmus Mozambique
AY541647 S.crumenophthalmus Japan
DBMF-M426 Selar crumenophthalmus Kuching (Sarawak)
HQ149945 NPPF1149 Selar crumenophthalmus Persian Gulf Iran
HQ149946 NPPF1147 Selar crumenophthalmus Persian Gulf Iran
HQ149947 NPPF1142 Selar crumenophthalmus Persian Gulf Iran
DBMF013-10 Selar crumenophthalmus K.Kedah (West PM)
JF494494 Selar crumenophthalmus Mozambique
JF494492 S.crumenophthalmus Mozambique
HQ149948 NPPF1017 Selar crumenophthalmus Persian Gulf Iran
HQ149944 NPPF1153 Selar crumenophthalmus Persian Gulf Iran
FJ583378 Echeneis naucrates
4.7%
0.02

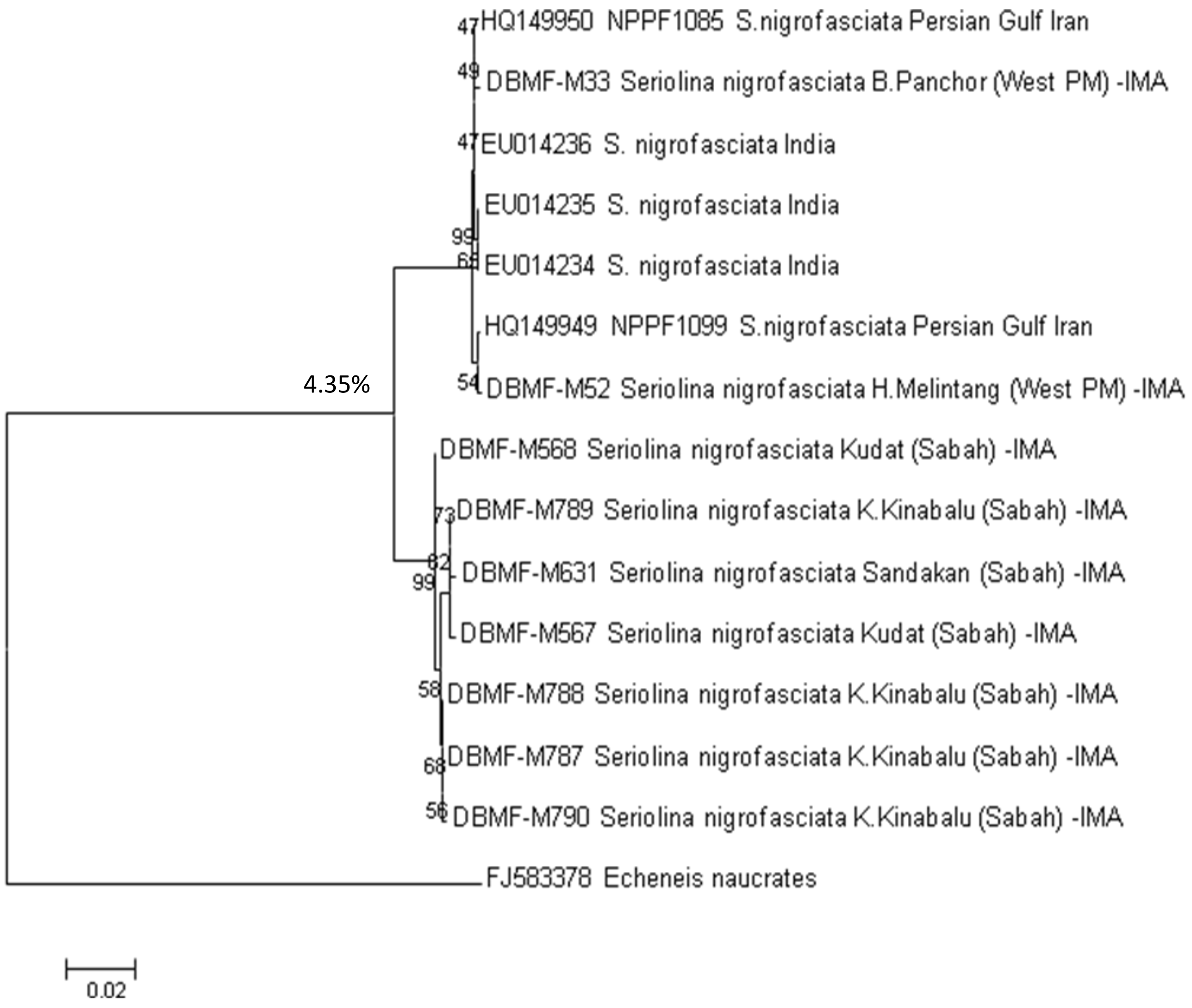
47 HQ149950 NPPF1085 S.nigrofasciata Persian Gulf Iran
49 DBMF-M33 Seriolina nigrofasciata B.Panchor (West PM) -IMA
47 EU014236 S. nigrofasciata India
EU014235 S. nigrofasciata India
99
65 EU014234 S. nigrofasciata India
HQ149949 NPPF1099 S.nigrofasciata Persian Gulf Iran
54 DBMF-M52 Seriolina nigrofasciata H.Melintang (West PM) -IMA
4.35%
DBMF-M568 Seriolina nigrofasciata Kudat (Sabah) -IMA
73 DBMF-M789 Seriolina nigrofasciata K.Kinabalu (Sabah) -IMA
82
99 DBMF-M631 Seriolina nigrofasciata Sandakan (Sabah) -IMA
DBMF-M567 Seriolina nigrofasciata Kudat (Sabah) -IMA
58 DBMF-M788 Seriolina nigrofasciata K.Kinabalu (Sabah) -IMA
68 DBMF-M787 Seriolina nigrofasciata K.Kinabalu (Sabah) -IMA
56 DBMF-M790 Seriolina nigrofasciata K.Kinabalu (Sabah) -IMA
FJ583378 Echeneis naucrates
0.02

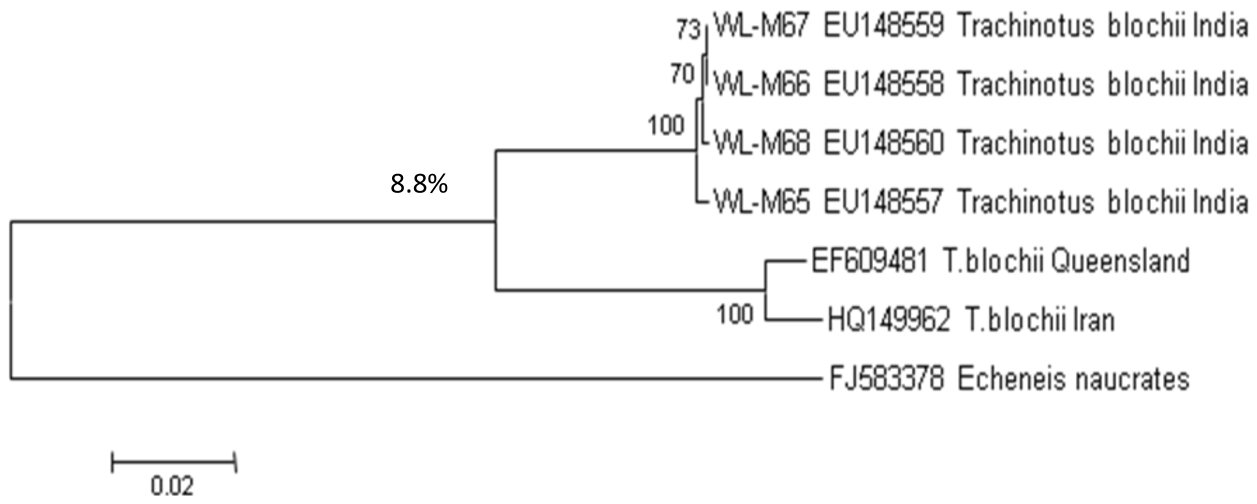
73
70
100
WL-M67 EU148559 Trachinotus blochii India
WL-M66 EU148558 Trachinotus blochii India
WL-M68 EU148560 Trachinotus blochii India
WL-M65 EU148557 Trachinotus blochii India
8.8%
EF609481 T.blochii Queensland
100
HQ149962 T.blochii Iran
FJ583378 Echeneis naucrates
0.02

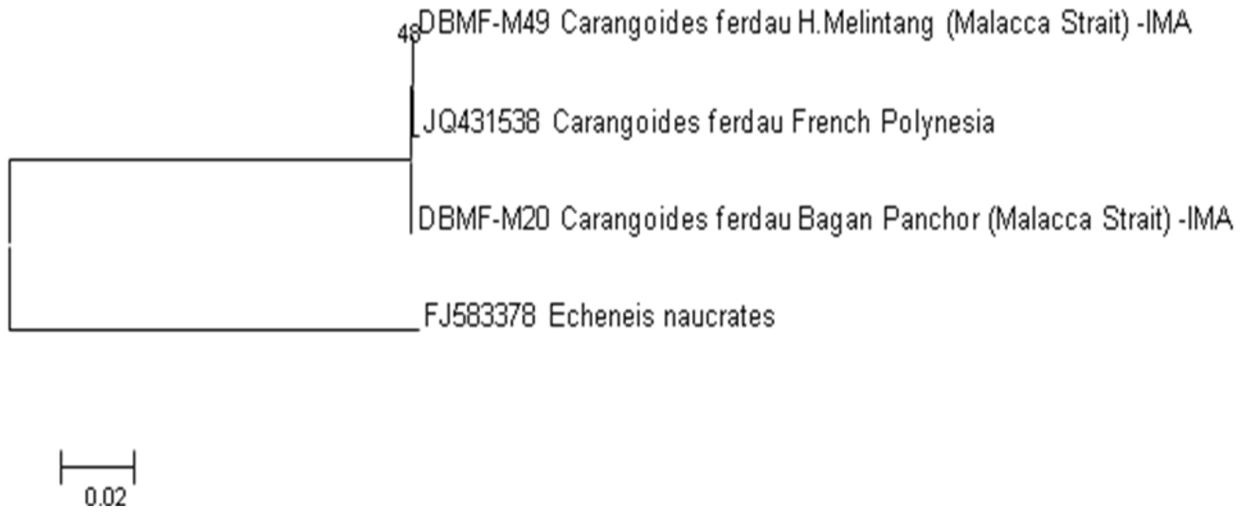
48
DBMF-M49 Carangoides ferdau H.Melintang (Malacca Strait) -IMA
JQ431538 Carangoides ferdau French Polynesia
DBMF-M20 Carangoides ferdau Bagan Panchor (Malacca Strait) -IMA
FJ583378 Echeneis naucrates
0.02

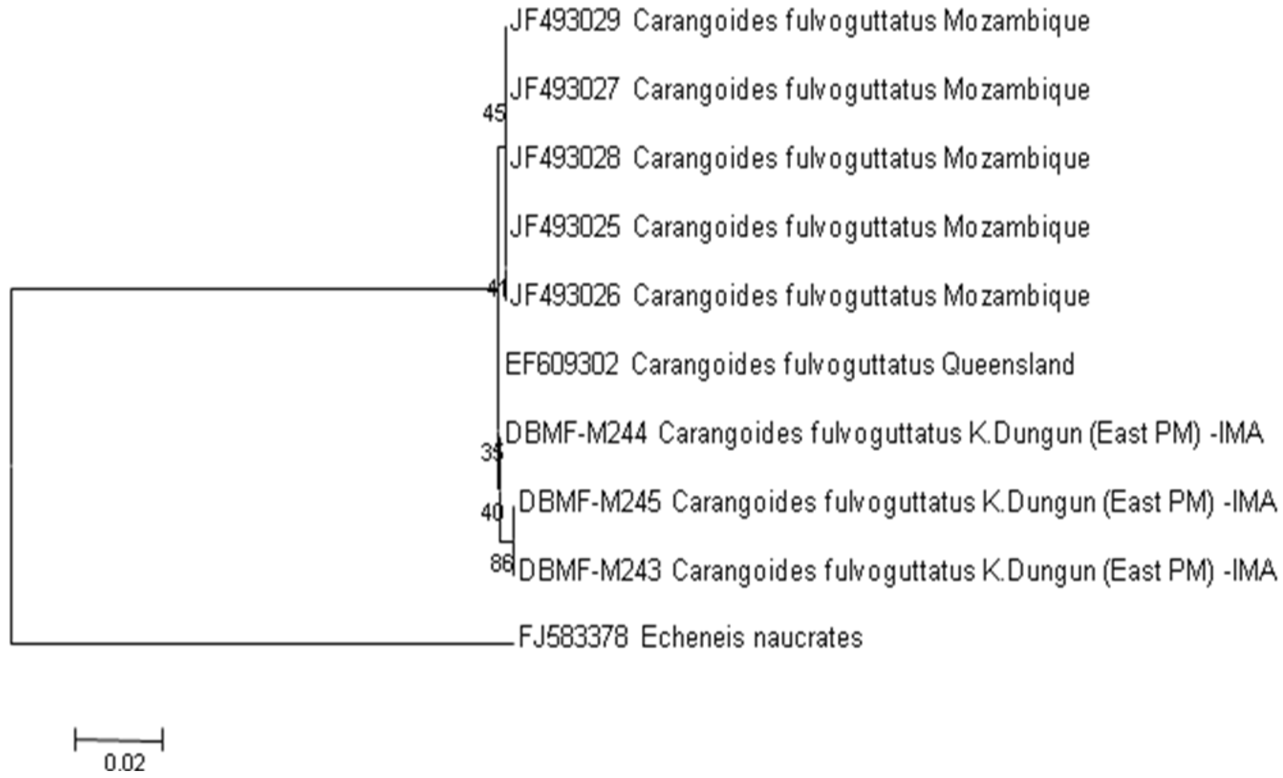
JF493029 Carangoides fulvoguttatus Mozambique
JF493027 Carangoides fulvoguttatus Mozambique
45
JF493028 Carangoides fulvoguttatus Mozambique
JF493025 Carangoides fulvoguttatus Mozambique
41
JF493026 Carangoides fulvoguttatus Mozambique
EF609302 Carangoides fulvoguttatus Queensland
DBMF-M244 Carangoides fulvoguttatus K.Dungun (East PM) -IMA
35
40
DBMF-M245 Carangoides fulvoguttatus K.Dungun (East PM) -IMA
86
DBMF-M243 Carangoides fulvoguttatus K.Dungun (East PM) -IMA
FJ583378 Echeneis naucrates
0.02

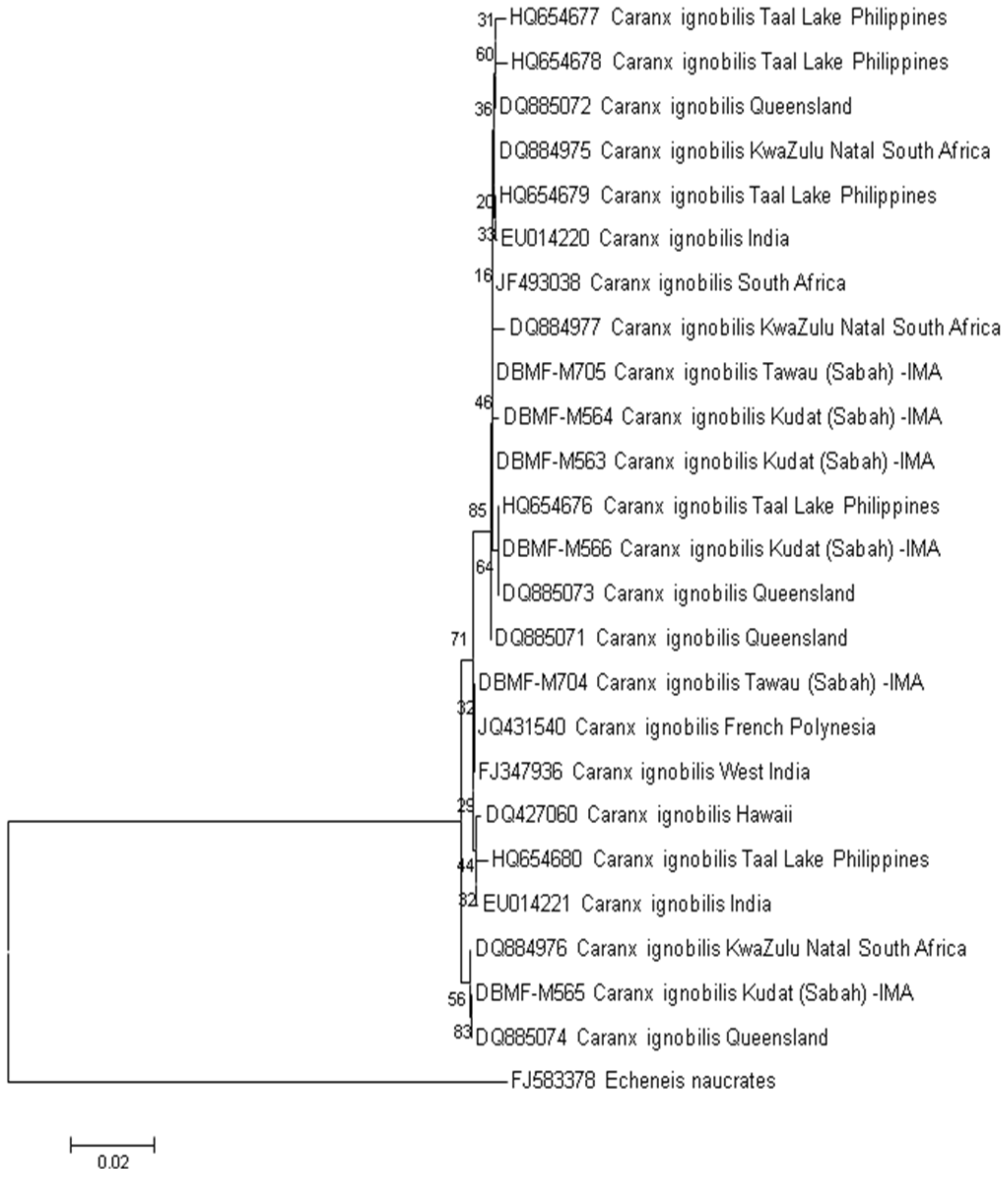

HQ654677 Caranx ignobilis Taal Lake Philippines
HQ654678 Caranx ignobilis Taal Lake Philippines
DQ885072 Caranx ignobilis Queensland
DQ884975 Caranx ignobilis KwaZulu Natal South Africa
HQ654679 Caranx ignobilis Taal Lake Philippines
EU014220 Caranx ignobilis India
JF493038 Caranx ignobilis South Africa
DQ884977 Caranx ignobilis KwaZulu Natal South Africa
DBMF-M705 Caranx ignobilis Tawau (Sabah) -IMA
DBMF-M564 Caranx ignobilis Kudat (Sabah) -IMA
DBMF-M563 Caranx ignobilis Kudat (Sabah) -IMA
HQ654676 Caranx ignobilis Taal Lake Philippines
DBMF-M566 Caranx ignobilis Kudat (Sabah) -IMA
DQ885073 Caranx ignobilis Queensland
DQ885071 Caranx ignobilis Queensland
DBMF-M704 Caranx ignobilis Tawau (Sabah) -IMA
JQ431540 Caranx ignobilis French Polynesia
FJ347936 Caranx ignobilis West India
DQ427060 Caranx ignobilis Hawaii
HQ654680 Caranx ignobilis Taal Lake Philippines
EU014221 Caranx ignobilis India
DQ884976 Caranx ignobilis KwaZulu Natal South Africa
DBMF-M565 Caranx ignobilis Kudat (Sabah) -IMA
DQ885074 Caranx ignobilis Queensland
FJ583378 Echeneis naucrates
31
60
36
20
33
16
46
85
64
71
32
29
44
32
56
83
0.02

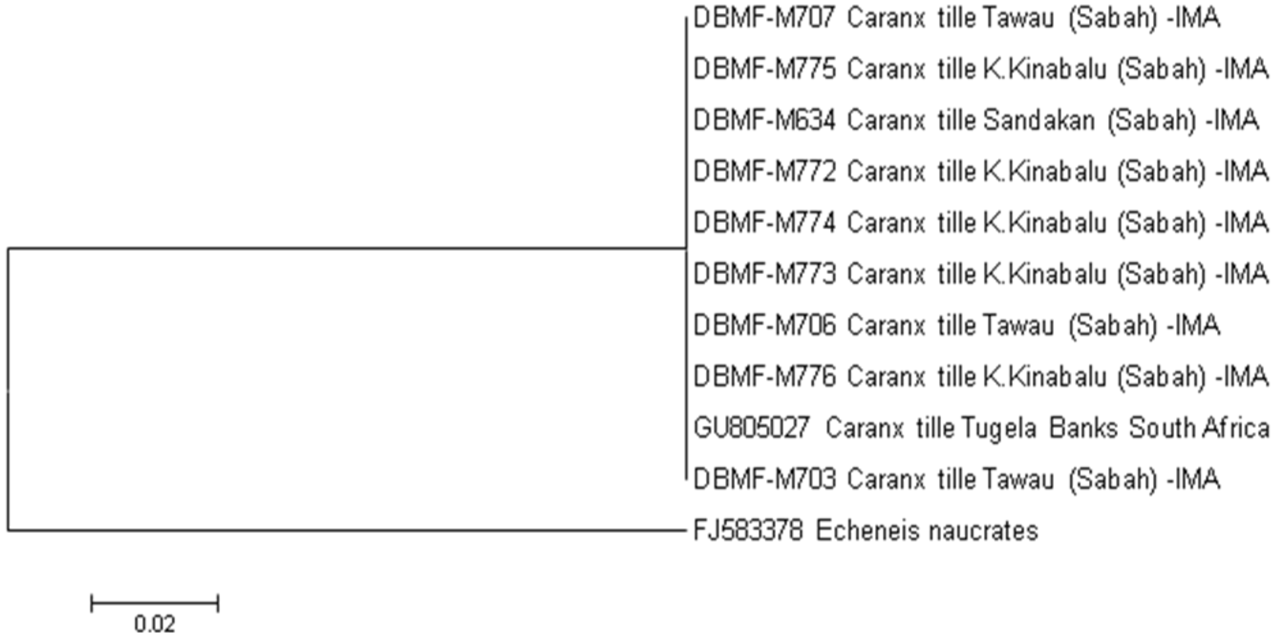
DBMF-M707 Caranx tille Tawau (Sabah) -IMA
DBMF-M775 Caranx tille K.Kinabalu (Sabah) -IMA
DBMF-M634 Caranx tille Sandakan (Sabah) -IMA
DBMF-M772 Caranx tille K.Kinabalu (Sabah) -IMA
DBMF-M774 Caranx tille K.Kinabalu (Sabah) -IMA
DBMF-M773 Caranx tille K.Kinabalu (Sabah) -IMA
DBMF-M706 Caranx tille Tawau (Sabah) -IMA
DBMF-M776 Caranx tille K.Kinabalu (Sabah) -IMA
GU805027 Caranx tille Tugela Banks South Africa
DBMF-M703 Caranx tille Tawau (Sabah) -IMA
FJ583378 Echeneis naucrates
0.02

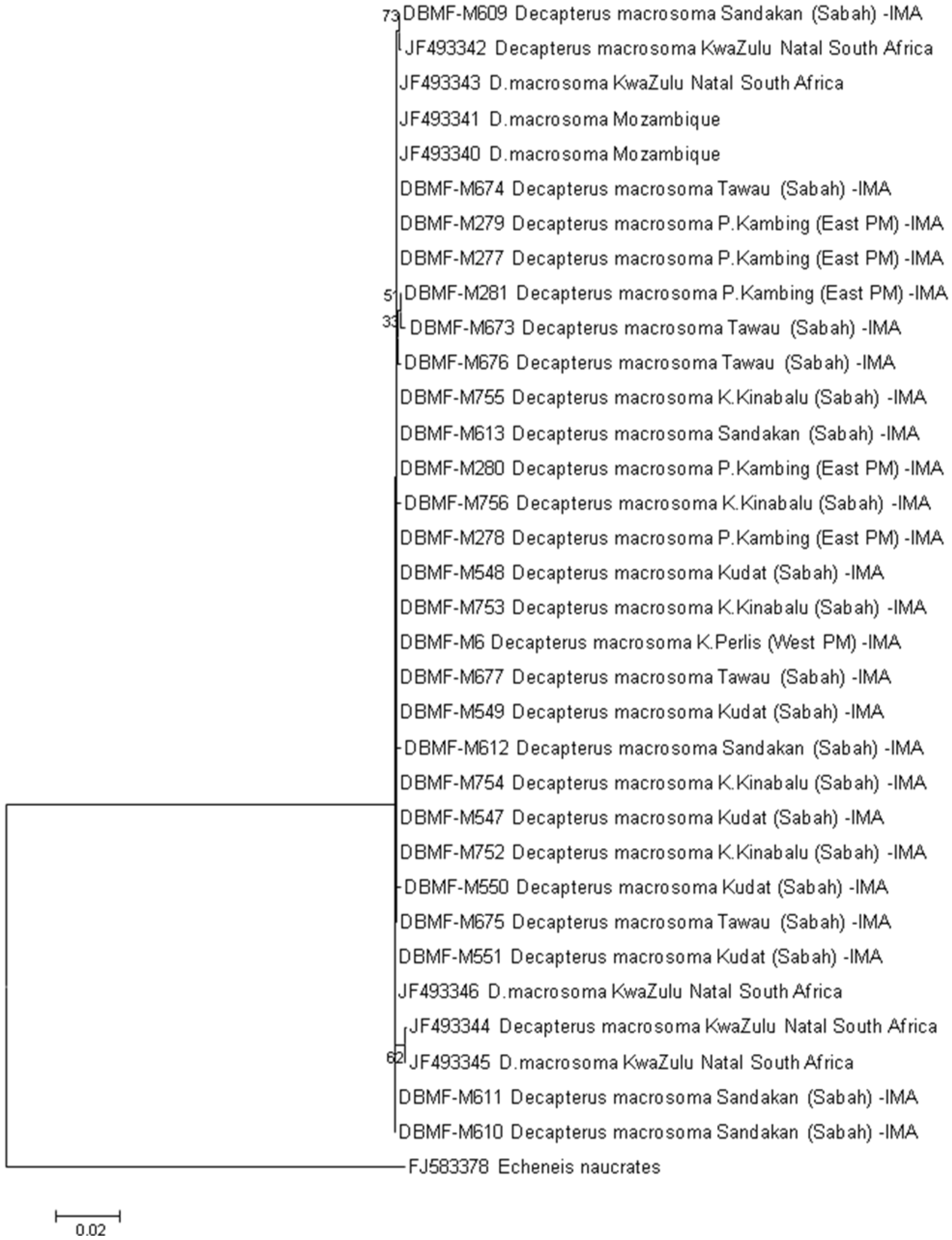

73
DBMF-M609 Decapterus macrosoma Sandakan (Sabah) -IMA
JF493342 Decapterus macrosoma KwaZulu Natal South Africa
JF493343 D.macrosoma KwaZulu Natal South Africa
JF493341 D.macrosoma Mozambique
JF493340 D.macrosoma Mozambique
DBMF-M674 Decapterus macrosoma Tawau (Sabah) -IMA
DBMF-M279 Decapterus macrosoma P.Kambing (East PM) -IMA
DBMF-M277 Decapterus macrosoma P.Kambing (East PM) -IMA
51
DBMF-M281 Decapterus macrosoma P.Kambing (East PM) -IMA
33
DBMF-M673 Decapterus macrosoma Tawau (Sabah) -IMA
DBMF-M676 Decapterus macrosoma Tawau (Sabah) -IMA
DBMF-M755 Decapterus macrosoma K.Kinabalu (Sabah) -IMA
DBMF-M613 Decapterus macrosoma Sandakan (Sabah) -IMA
DBMF-M280 Decapterus macrosoma P.Kambing (East PM) -IMA
DBMF-M756 Decapterus macrosoma K.Kinabalu (Sabah) -IMA
DBMF-M278 Decapterus macrosoma P.Kambing (East PM) -IMA
DBMF-M548 Decapterus macrosoma Kudat (Sabah) -IMA
DBMF-M753 Decapterus macrosoma K.Kinabalu (Sabah) -IMA
DBMF-M6 Decapterus macrosoma K.Perlis (West PM) -IMA
DBMF-M677 Decapterus macrosoma Tawau (Sabah) -IMA
DBMF-M549 Decapterus macrosoma Kudat (Sabah) -IMA
DBMF-M612 Decapterus macrosoma Sandakan (Sabah) -IMA
DBMF-M754 Decapterus macrosoma K.Kinabalu (Sabah) -IMA
DBMF-M547 Decapterus macrosoma Kudat (Sabah) -IMA
DBMF-M752 Decapterus macrosoma K.Kinabalu (Sabah) -IMA
DBMF-M550 Decapterus macrosoma Kudat (Sabah) -IMA
DBMF-M675 Decapterus macrosoma Tawau (Sabah) -IMA
DBMF-M551 Decapterus macrosoma Kudat (Sabah) -IMA
JF493346 D.macrosoma KwaZulu Natal South Africa
JF493344 Decapterus macrosoma KwaZulu Natal South Africa
62
JF493345 D.macrosoma KwaZulu Natal South Africa
DBMF-M611 Decapterus macrosoma Sandakan (Sabah) -IMA
DBMF-M610 Decapterus macrosoma Sandakan (Sabah) -IMA
FJ583378 Echeneis naucrates
0.02

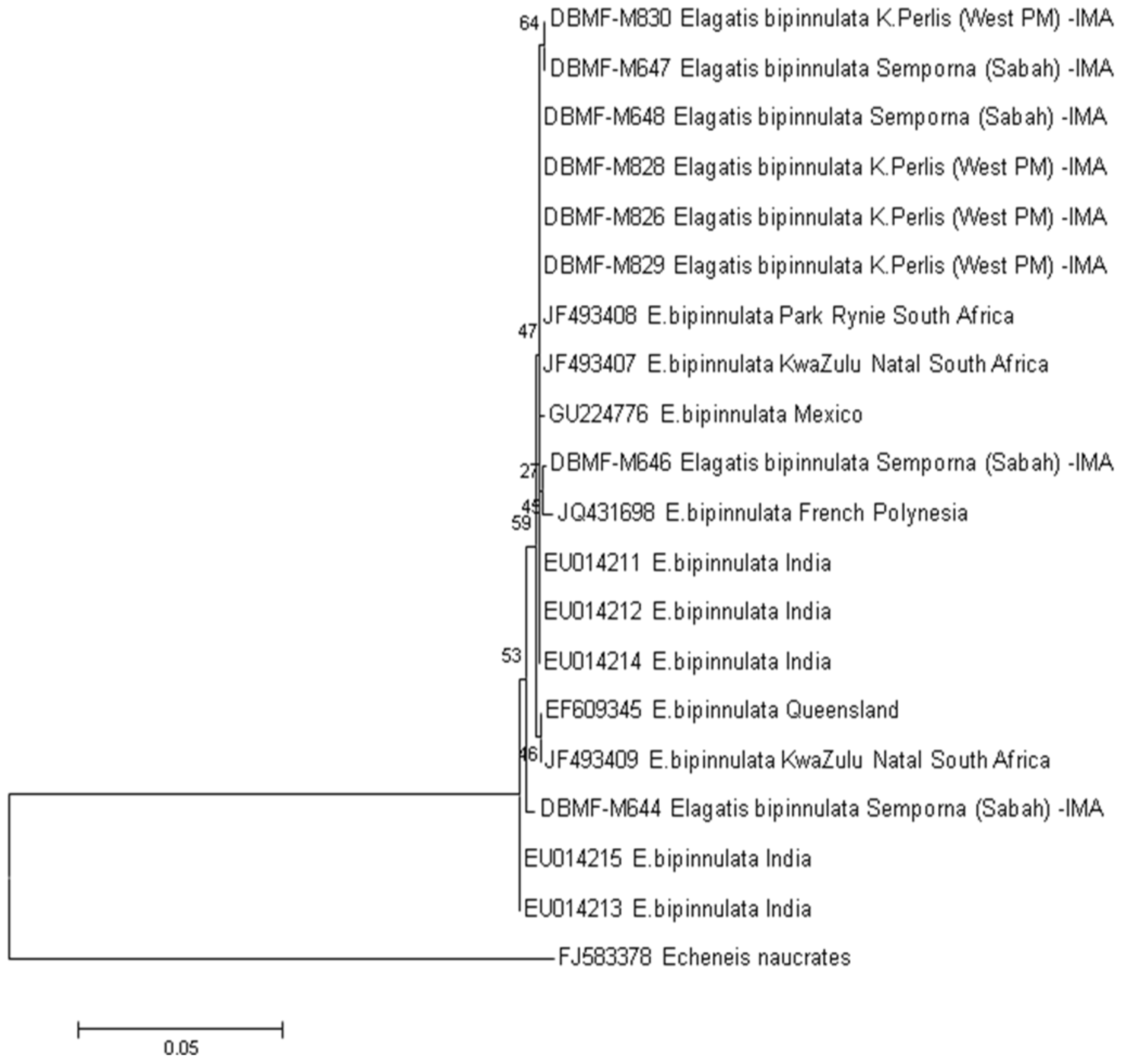
DBMF-M830 Elagatis bipinnulata K.Perlis (West PM) -IMA
DBMF-M647 Elagatis bipinnulata Semporna (Sabah) -IMA
DBMF-M648 Elagatis bipinnulata Semporna (Sabah) -IMA
DBMF-M828 Elagatis bipinnulata K.Perlis (West PM) -IMA
DBMF-M826 Elagatis bipinnulata K.Perlis (West PM) -IMA
DBMF-M829 Elagatis bipinnulata K.Perlis (West PM) -IMA
JF493408 E.bipinnulata Park Rynie South Africa
JF493407 E.bipinnulata KwaZulu Natal South Africa
GU224776 E.bipinnulata Mexico
DBMF-M646 Elagatis bipinnulata Semporna (Sabah) -IMA
JQ431698 E.bipinnulata French Polynesia
EU014211 E.bipinnulata India
EU014212 E.bipinnulata India
EU014214 E.bipinnulata India
EF609345 E.bipinnulata Queensland
JF493409 E.bipinnulata KwaZulu Natal South Africa
DBMF-M644 Elagatis bipinnulata Semporna (Sabah) -IMA
EU014215 E.bipinnulata India
EU014213 E.bipinnulata India
FJ583378 Echeneis naucrates
64
47
27
45
59
53
46
0.05

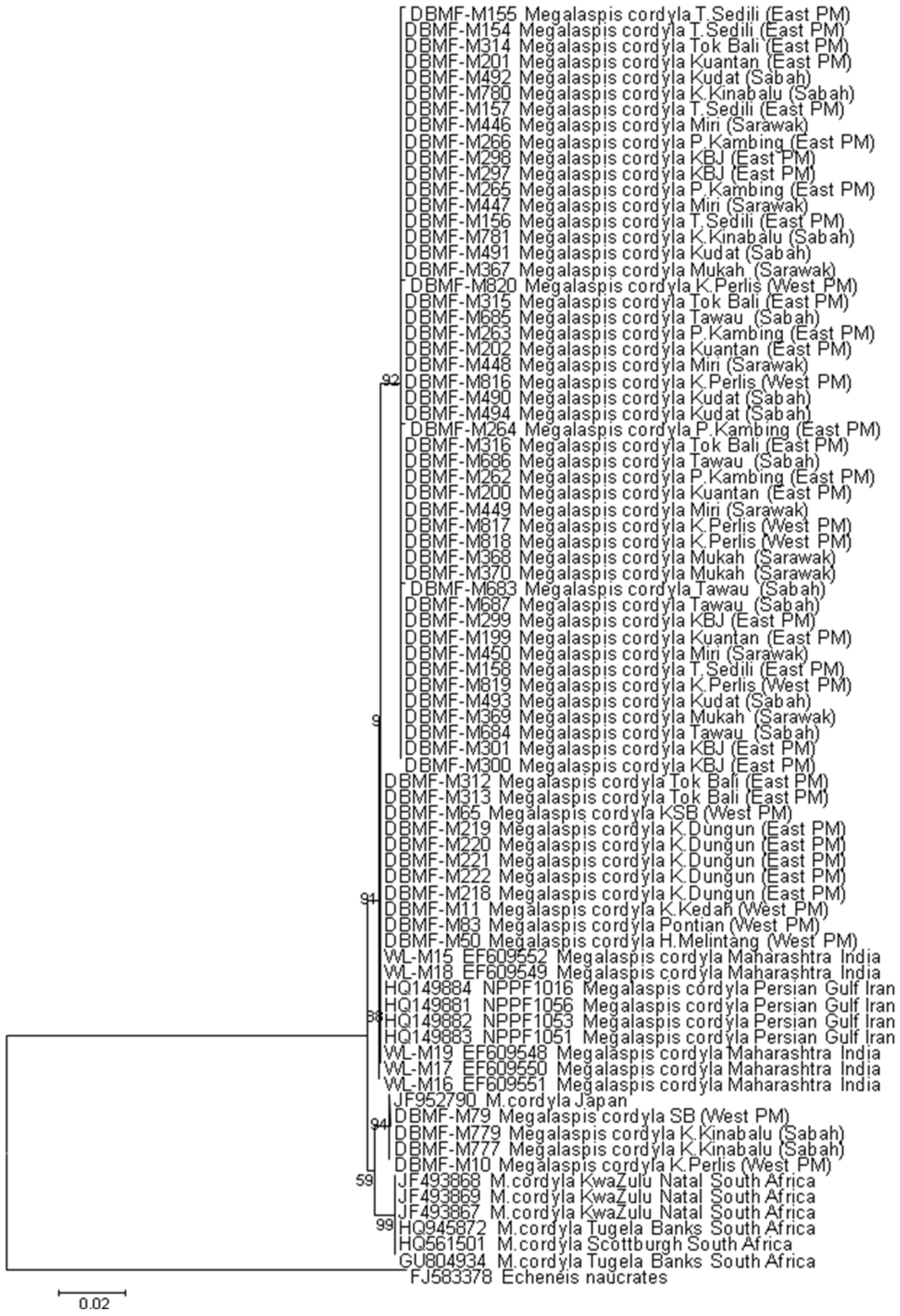
DBMF-M155 Megalaspis cordyla T.Sedili (East PM)
DBMF-M154 Megalaspis cordyla T.Sedili (East PM)
DBMF-M314 Megalaspis cordyla Tok Bali (East PM)
DBMF-M201 Megalaspis cordyla Kuantan (East PM)
DBMF-M492 Megalaspis cordyla Kudat (Sabah)
DBMF-M780 Megalaspis cordyla K.Kinabalu (Sabah)
DBMF-M157 Megalaspis cordyla T.Sedili (East PM)
DBMF-M446 Megalaspis cordyla Miri (Sarawak)
DBMF-M266 Megalaspis cordyla P.Kambing (East PM)
DBMF-M298 Megalaspis cordyla KBJ (East PM)
DBMF-M297 Megalaspis cordyla KBJ (East PM)
DBMF-M265 Megalaspis cordyla P.Kambing (East PM)
DBMF-M447 Megalaspis cordyla Miri (Sarawak)
DBMF-M156 Megalaspis cordyla T.Sedili (East PM)
DBMF-M781 Megalaspis cordyla K.Kinabalu (Sabah)
DBMF-M491 Megalaspis cordyla Kudat (Sabah)
DBMF-M367 Megalaspis cordyla Mukah (Sarawak)
DBMF-M820 Megalaspis cordyla K.Perlis (West PM)
DBMF-M315 Megalaspis cordyla Tok Bali (East PM)
DBMF-M685 Megalaspis cordyla Tawau (Sabah)
DBMF-M263 Megalaspis cordyla P.Kambing (East PM)
DBMF-M202 Megalaspis cordyla Kuantan (East PM)
DBMF-M448 Megalaspis cordyla Miri (Sarawak)
92
DBMF-M816 Megalaspis cordyla K.Perlis (West PM)
DBMF-M490 Megalaspis cordyla Kudat (Sabah)
DBMF-M494 Megalaspis cordyla Kudat (Sabah)
DBMF-M264 Megalaspis cordyla P.Kambing (East PM)
DBMF-M316 Megalaspis cordyla Tok Bali (East PM)
DBMF-M686 Megalaspis cordyla Tawau (Sabah)
DBMF-M262 Megalaspis cordyla P.Kambing (East PM)
DBMF-M200 Megalaspis cordyla Kuantan (East PM)
DBMF-M449 Megalaspis cordyla Miri (Sarawak)
DBMF-M817 Megalaspis cordyla K.Perlis (West PM)
DBMF-M818 Megalaspis cordyla K.Perlis (West PM)
DBMF-M368 Megalaspis cordyla Mukah (Sarawak)
DBMF-M370 Megalaspis cordyla Mukah (Sarawak)
DBMF-M683 Megalaspis cordyla Tawau (Sabah)
DBMF-M687 Megalaspis cordyla Tawau (Sabah)
DBMF-M299 Megalaspis cordyla KBJ (East PM)
DBMF-M199 Megalaspis cordyla Kuantan (East PM)
DBMF-M450 Megalaspis cordyla Miri (Sarawak)
DBMF-M158 Megalaspis cordyla T.Sedili (East PM)
DBMF-M819 Megalaspis cordyla K.Perlis (West PM)
DBMF-M493 Megalaspis cordyla Kudat (Sabah)
9
DBMF-M369 Megalaspis cordyla Mukah (Sarawak)
DBMF-M684 Megalaspis cordyla Tawau (Sabah)
DBMF-M301 Megalaspis cordyla KBJ (East PM)
DBMF-M300 Megalaspis cordyla KBJ (East PM)
DBMF-M312 Megalaspis cordyla Tok Bali (East PM)
DBMF-M313 Megalaspis cordyla Tok Bali (East PM)
DBMF-M65 Megalaspis cordyla KSB (West PM)
DBMF-M219 Megalaspis cordyla K.Dungun (East PM)
DBMF-M220 Megalaspis cordyla K.Dungun (East PM)
DBMF-M221 Megalaspis cordyla K.Dungun (East PM)
DBMF-M222 Megalaspis cordyla K.Dungun (East PM)
DBMF-M218 Megalaspis cordyla K.Dungun (East PM)
94
DBMF-M11 Megalaspis cordyla K.Kedah (West PM)
DBMF-M83 Megalaspis cordyla Pontian (West PM)
DBMF-M50 Megalaspis cordyla H.Melintang (West PM)
WL-M15 EF609552 Megalaspis cordyla Maharashtra India
WL-M18 EF609549 Megalaspis cordyla Maharashtra India
HQ149884 NPPF1016 Megalaspis cordyla Persian Gulf Iran
HQ149881 NPPF1056 Megalaspis cordyla Persian Gulf Iran
88
HQ149882 NPPF1053 Megalaspis cordyla Persian Gulf Iran
HQ149883 NPPF1051 Megalaspis cordyla Persian Gulf Iran
WL-M19 EF609548 Megalaspis cordyla Maharashtra India
WL-M17 EF609550 Megalaspis cordyla Maharashtra India
WL-M16 EF609551 Megalaspis cordyla Maharashtra India
JF952790 M.cordyla Japan
DBMF-M79 Megalaspis cordyla SB (West PM)
94
DBMF-M779 Megalaspis cordyla K.Kinabalu (Sabah)
DBMF-M777 Megalaspis cordyla K.Kinabalu (Sabah)
DBMF-M10 Megalaspis cordyla K.Perlis (West PM)
59
JF493868 M.cordyla KwaZulu Natal South Africa
JF493869 M.cordyla KwaZulu Natal South Africa
JF493867 M.cordyla KwaZulu Natal South Africa
99
HQ945872 M.cordyla Tugela Banks South Africa
HQ561501 M.cordyla Scottburgh South Africa
GU804934 M.cordyla Tugela Banks South Africa
FJ583378 Echeneis naucrates
0.02

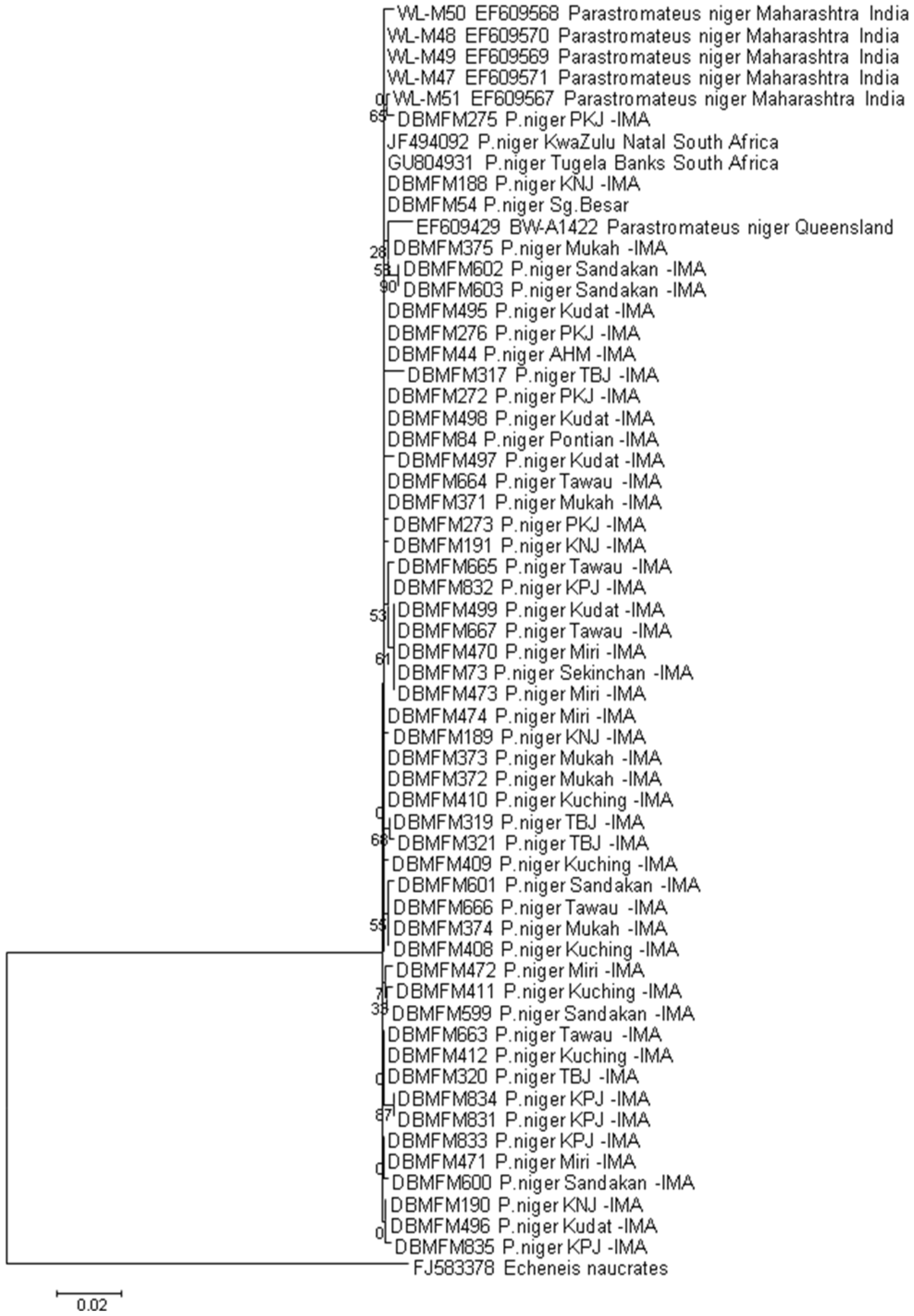

WL-M50 EF609568 Parastromateus niger Maharashtra India
WL-M48 EF609570 Parastromateus niger Maharashtra India
WL-M49 EF609569 Parastromateus niger Maharashtra India
WL-M47 EF609571 Parastromateus niger Maharashtra India
0
WL-M51 EF609567 Parastromateus niger Maharashtra India
65
DBMFM275 P.niger PKJ -IMA
JF494092 P.niger KwaZulu Natal South Africa
GU804931 P.niger Tugela Banks South Africa
DBMFM188 P.niger KNJ -IMA
DBMFM54 P.niger Sg.Besar
EF609429 BW-A1422 Parastromateus niger Queensland
28
DBMFM375 P.niger Mukah -IMA
58
DBMFM602 P.niger Sandakan -IMA
90
DBMFM603 P.niger Sandakan -IMA
DBMFM495 P.niger Kudat -IMA
DBMFM276 P.niger PKJ -IMA
DBMFM44 P.niger AHM -IMA
DBMFM317 P.niger TBJ -IMA
DBMFM272 P.niger PKJ -IMA
DBMFM498 P.niger Kudat -IMA
DBMFM84 P.niger Pontian -IMA
DBMFM497 P.niger Kudat -IMA
DBMFM664 P.niger Tawau -IMA
DBMFM371 P.niger Mukah -IMA
DBMFM273 P.niger PKJ -IMA
DBMFM191 P.niger KNJ -IMA
DBMFM665 P.niger Tawau -IMA
DBMFM832 P.niger KPJ -IMA
53
DBMFM499 P.niger Kudat -IMA
DBMFM667 P.niger Tawau -IMA
61
DBMFM470 P.niger Miri -IMA
DBMFM73 P.niger Sekinchan -IMA
DBMFM473 P.niger Miri -IMA
DBMFM474 P.niger Miri -IMA
DBMFM189 P.niger KNJ -IMA
DBMFM373 P.niger Mukah -IMA
DBMFM372 P.niger Mukah -IMA
DBMFM410 P.niger Kuching -IMA
0
DBMFM319 P.niger TBJ -IMA
68
DBMFM321 P.niger TBJ -IMA
DBMFM409 P.niger Kuching -IMA
DBMFM601 P.niger Sandakan -IMA
DBMFM666 P.niger Tawau -IMA
55
DBMFM374 P.niger Mukah -IMA
DBMFM408 P.niger Kuching -IMA
DBMFM472 P.niger Miri -IMA
7
DBMFM411 P.niger Kuching -IMA
35
DBMFM599 P.niger Sandakan -IMA
DBMFM663 P.niger Tawau -IMA
DBMFM412 P.niger Kuching -IMA
0
DBMFM320 P.niger TBJ -IMA
DBMFM834 P.niger KPJ -IMA
87
DBMFM831 P.niger KPJ -IMA
DBMFM833 P.niger KPJ -IMA
0
DBMFM471 P.niger Miri -IMA
DBMFM600 P.niger Sandakan -IMA
DBMFM190 P.niger KNJ -IMA
DBMFM496 P.niger Kudat -IMA
0
DBMFM835 P.niger KPJ -IMA
FJ583378 Echeneis naucrates
0.02

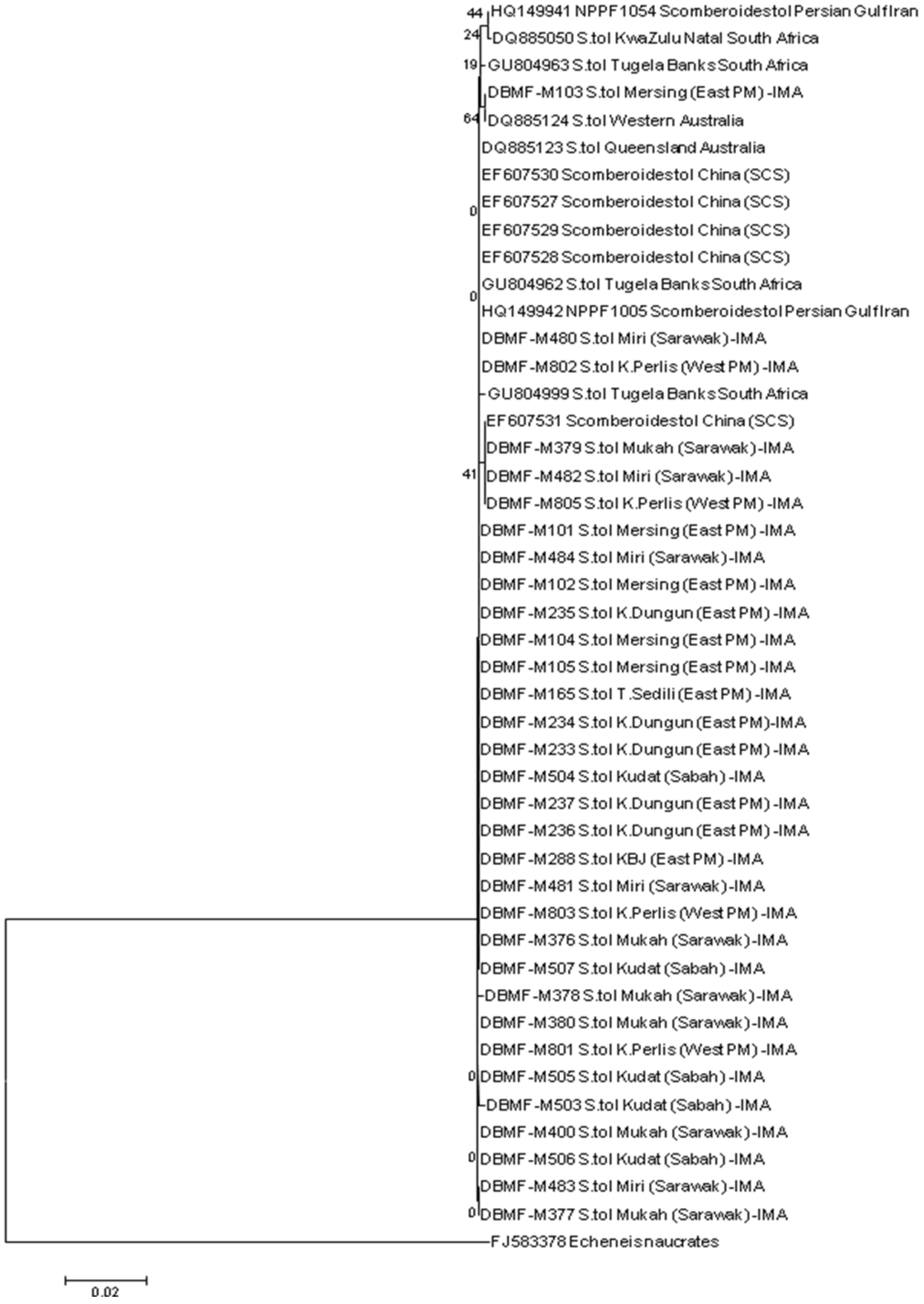
44
HQ149941 NPPF1054 Scomberoidestol Persian Gulf Iran
24
DQ885050 S.tol KwaZulu Natal South Africa
19
GU804963 S.tol Tugela Banks South Africa
DBMF-M103 S.tol Mersing (East PM) -IMA
64
DQ885124 S.tol Western Australia
DQ885123 S.tol Queensland Australia
EF607530 Scomberoidestol China (SCS)
0
EF607527 Scomberoidestol China (SCS)
EF607529 Scomberoidestol China (SCS)
EF607528 Scomberoidestol China (SCS)
0
GU804962 S.tol Tugela Banks South Africa
HQ149942 NPPF1005 Scomberoidestol Persian Gulf Iran
DBMF-M480 S.tol Miri (Sarawak) -IMA
DBMF-M802 S.tol K.Perlis (West PM) -IMA
GU804999 S.tol Tugela Banks South Africa
EF607531 Scomberoidestol China (SCS)
DBMF-M379 S.tol Mukah (Sarawak) -IMA
41
DBMF-M482 S.tol Miri (Sarawak) -IMA
DBMF-M805 S.tol K.Perlis (West PM) -IMA
DBMF-M101 S.tol Mersing (East PM) -IMA
DBMF-M484 S.tol Miri (Sarawak) -IMA
DBMF-M102 S.tol Mersing (East PM) -IMA
DBMF-M235 S.tol K.Dungun (East PM) -IMA
DBMF-M104 S.tol Mersing (East PM) -IMA
DBMF-M105 S.tol Mersing (East PM) -IMA
DBMF-M165 S.tol T.Sedili (East PM) -IMA
DBMF-M234 S.tol K.Dungun (East PM)-IMA
DBMF-M233 S.tol K.Dungun (East PM) -IMA
DBMF-M504 S.tol Kudat (Sabah) -IMA
DBMF-M237 S.tol K.Dungun (East PM) -IMA
DBMF-M236 S.tol K.Dungun (East PM) -IMA
DBMF-M288 S.tol KBJ (East PM) -IMA
DBMF-M481 S.tol Miri (Sarawak) -IMA
DBMF-M803 S.tol K.Perlis (West PM) -IMA
DBMF-M376 S.tol Mukah (Sarawak) -IMA
DBMF-M507 S.tol Kudat (Sabah) -IMA
DBMF-M378 S.tol Mukah (Sarawak) -IMA
DBMF-M380 S.tol Mukah (Sarawak) -IMA
DBMF-M801 S.tol K.Perlis (West PM) -IMA
0
DBMF-M505 S.tol Kudat (Sabah) -IMA
DBMF-M503 S.tol Kudat (Sabah) -IMA
DBMF-M400 S.tol Mukah (Sarawak) -IMA
0
DBMF-M506 S.tol Kudat (Sabah) -IMA
DBMF-M483 S.tol Miri (Sarawak) -IMA
0
DBMF-M377 S.tol Mukah (Sarawak) -IMA
FJ583378 Echeneis naucrates
0.02

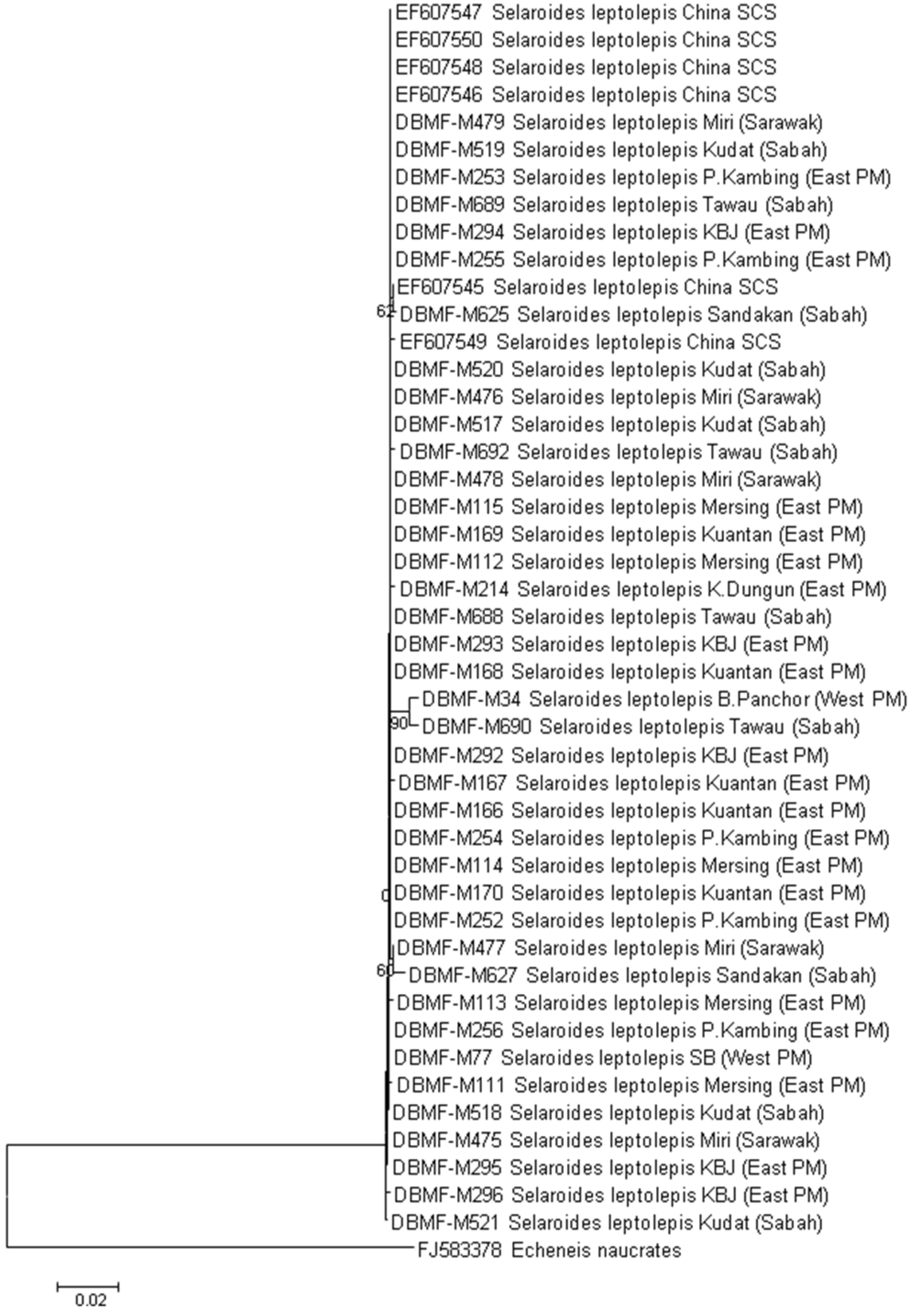
EF607547 Selaroides leptolepis China SCS
EF607550 Selaroides leptolepis China SCS
EF607548 Selaroides leptolepis China SCS
EF607546 Selaroides leptolepis China SCS
DBMF-M479 Selaroides leptolepis Miri (Sarawak)
DBMF-M519 Selaroides leptolepis Kudat (Sabah)
DBMF-M253 Selaroides leptolepis P.Kambing (East PM)
DBMF-M689 Selaroides leptolepis Tawau (Sabah)
DBMF-M294 Selaroides leptolepis KBJ (East PM)
DBMF-M255 Selaroides leptolepis P.Kambing (East PM)
EF607545 Selaroides leptolepis China SCS
62
DBMF-M625 Selaroides leptolepis Sandakan (Sabah)
EF607549 Selaroides leptolepis China SCS
DBMF-M520 Selaroides leptolepis Kudat (Sabah)
DBMF-M476 Selaroides leptolepis Miri (Sarawak)
DBMF-M517 Selaroides leptolepis Kudat (Sabah)
DBMF-M692 Selaroides leptolepis Tawau (Sabah)
DBMF-M478 Selaroides leptolepis Miri (Sarawak)
DBMF-M115 Selaroides leptolepis Mersing (East PM)
DBMF-M169 Selaroides leptolepis Kuantan (East PM)
DBMF-M112 Selaroides leptolepis Mersing (East PM)
DBMF-M214 Selaroides leptolepis K.Dungun (East PM)
DBMF-M688 Selaroides leptolepis Tawau (Sabah)
DBMF-M293 Selaroides leptolepis KBJ (East PM)
DBMF-M168 Selaroides leptolepis Kuantan (East PM)
DBMF-M34 Selaroides leptolepis B.Panchor (West PM)
90
DBMF-M690 Selaroides leptolepis Tawau (Sabah)
DBMF-M292 Selaroides leptolepis KBJ (East PM)
DBMF-M167 Selaroides leptolepis Kuantan (East PM)
DBMF-M166 Selaroides leptolepis Kuantan (East PM)
DBMF-M254 Selaroides leptolepis P.Kambing (East PM)
DBMF-M114 Selaroides leptolepis Mersing (East PM)
0
DBMF-M170 Selaroides leptolepis Kuantan (East PM)
DBMF-M252 Selaroides leptolepis P.Kambing (East PM)
DBMF-M477 Selaroides leptolepis Miri (Sarawak)
60
DBMF-M627 Selaroides leptolepis Sandakan (Sabah)
DBMF-M113 Selaroides leptolepis Mersing (East PM)
DBMF-M256 Selaroides leptolepis P.Kambing (East PM)
DBMF-M77 Selaroides leptolepis SB (West PM)
DBMF-M111 Selaroides leptolepis Mersing (East PM)
DBMF-M518 Selaroides leptolepis Kudat (Sabah)
DBMF-M475 Selaroides leptolepis Miri (Sarawak)
DBMF-M295 Selaroides leptolepis KBJ (East PM)
DBMF-M296 Selaroides leptolepis KBJ (East PM)
DBMF-M521 Selaroides leptolepis Kudat (Sabah)
FJ583378 Echeneis naucrates
0.02

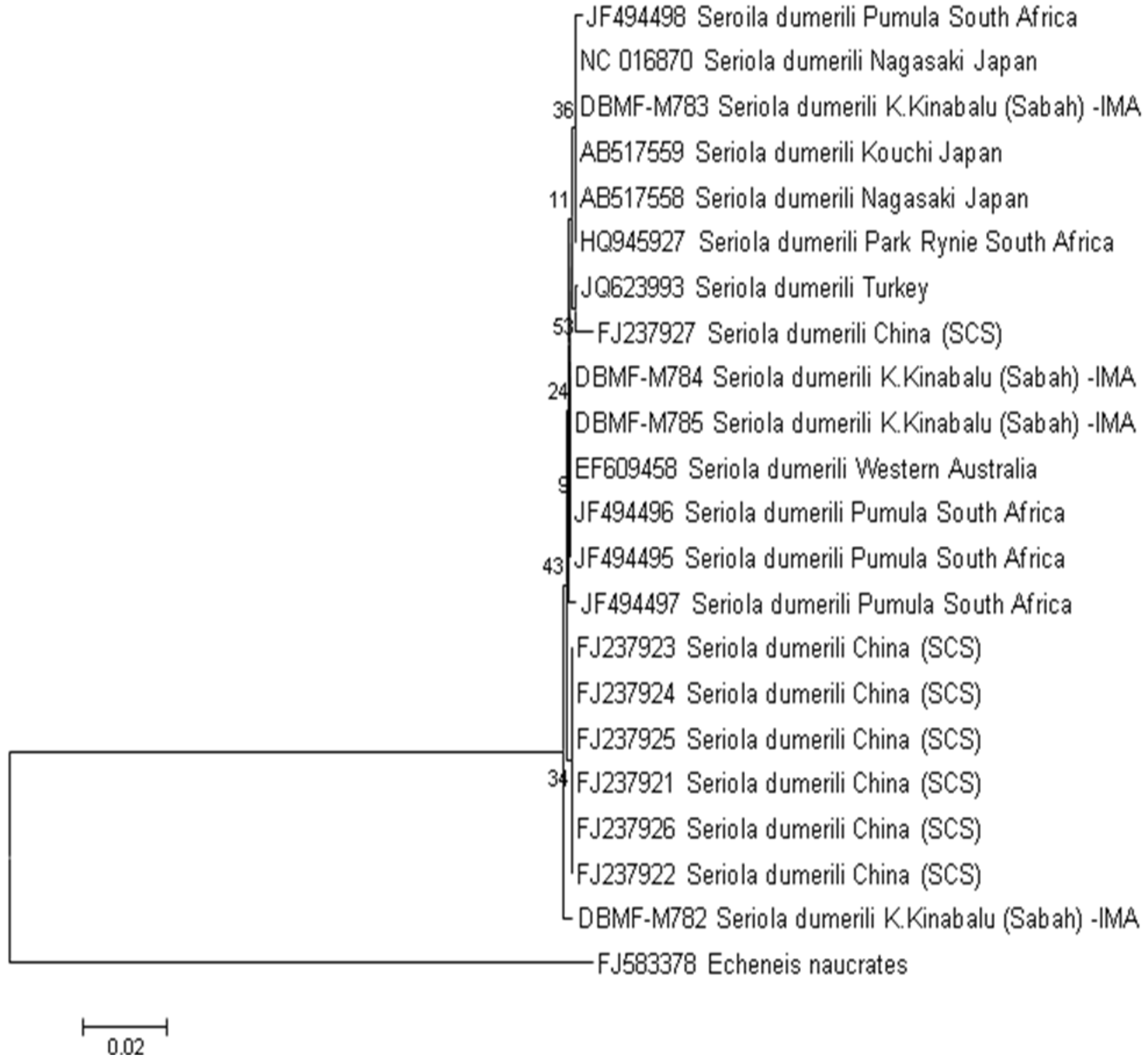
JF494498 Seroila dumerili Pumula South Africa
NC 016870 Seriola dumerili Nagasaki Japan
36
DBMF-M783 Seriola dumerili K.Kinabalu (Sabah) -IMA
AB517559 Seriola dumerili Kouchi Japan
11
AB517558 Seriola dumerili Nagasaki Japan
HQ945927 Seriola dumerili Park Rynie South Africa
JQ623993 Seriola dumerili Turkey
53
FJ237927 Seriola dumerili China (SCS)
24
DBMF-M784 Seriola dumerili K.Kinabalu (Sabah) -IMA
DBMF-M785 Seriola dumerili K.Kinabalu (Sabah) -IMA
9
EF609458 Seriola dumerili Western Australia
JF494496 Seriola dumerili Pumula South Africa
43
JF494495 Seriola dumerili Pumula South Africa
JF494497 Seriola dumerili Pumula South Africa
FJ237923 Seriola dumerili China (SCS)
FJ237924 Seriola dumerili China (SCS)
FJ237925 Seriola dumerili China (SCS)
34
FJ237921 Seriola dumerili China (SCS)
FJ237926 Seriola dumerili China (SCS)
FJ237922 Seriola dumerili China (SCS)
DBMF-M782 Seriola dumerili K.Kinabalu (Sabah) -IMA
FJ583378 Echeneis naucrates
0.02

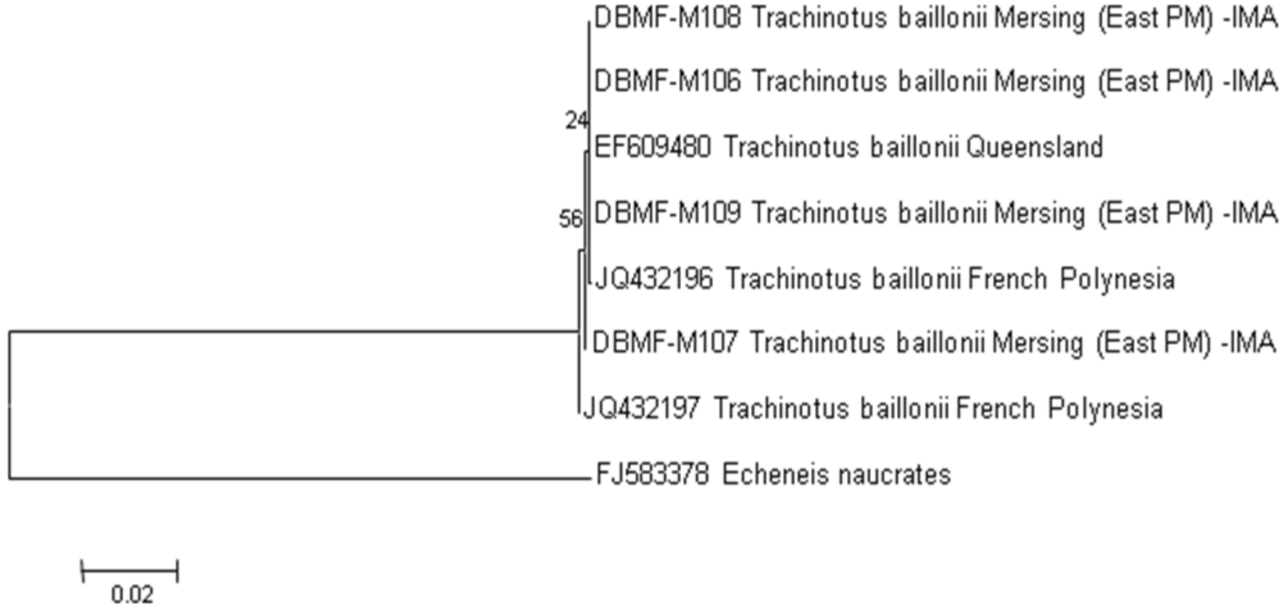
DBMF-M108 Trachinotus baillonii Mersing (East PM) -IMA
DBMF-M106 Trachinotus baillonii Mersing (East PM) -IMA
24
EF609480 Trachinotus baillonii Queensland
56
DBMF-M109 Trachinotus baillonii Mersing (East PM) -IMA
JQ432196 Trachinotus baillonii French Polynesia
DBMF-M107 Trachinotus baillonii Mersing (East PM) -IMA
JQ432197 Trachinotus baillonii French Polynesia
FJ583378 Echeneis naucrates
0.02

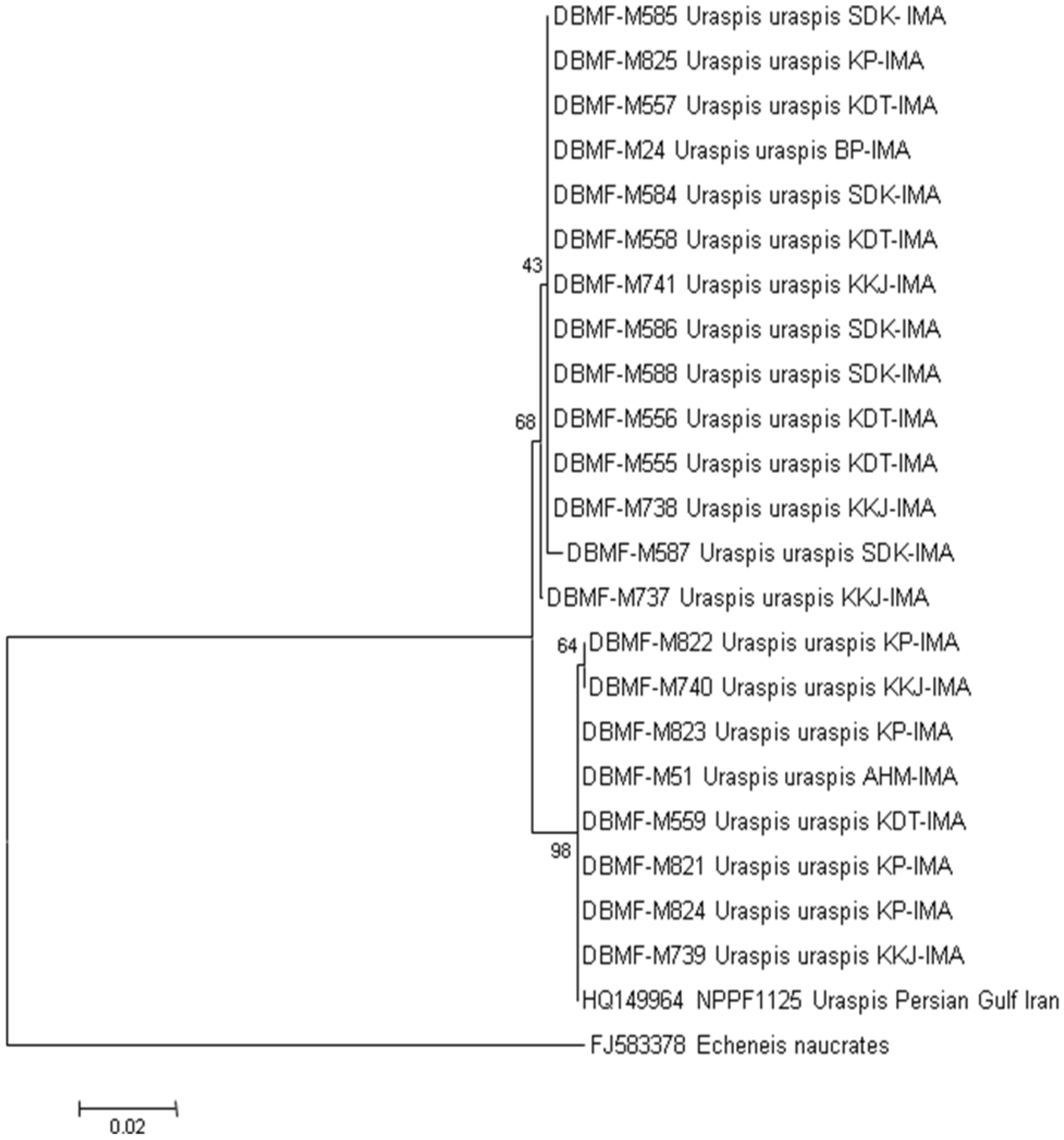

DBMF-M585 Uraspis uraspis SDK- IMA
DBMF-M825 Uraspis uraspis KP-IMA
DBMF-M557 Uraspis uraspis KDT-IMA
DBMF-M24 Uraspis uraspis BP-IMA
DBMF-M584 Uraspis uraspis SDK-IMA
DBMF-M558 Uraspis uraspis KDT-IMA
43
DBMF-M741 Uraspis uraspis KKJ-IMA
DBMF-M586 Uraspis uraspis SDK-IMA
DBMF-M588 Uraspis uraspis SDK-IMA
68
DBMF-M556 Uraspis uraspis KDT-IMA
DBMF-M555 Uraspis uraspis KDT-IMA
DBMF-M738 Uraspis uraspis KKJ-IMA
DBMF-M587 Uraspis uraspis SDK-IMA
DBMF-M737 Uraspis uraspis KKJ-IMA
64
DBMF-M822 Uraspis uraspis KP-IMA
DBMF-M740 Uraspis uraspis KKJ-IMA
DBMF-M823 Uraspis uraspis KP-IMA
DBMF-M51 Uraspis uraspis AHM-IMA
DBMF-M559 Uraspis uraspis KDT-IMA
98
DBMF-M821 Uraspis uraspis KP-IMA
DBMF-M824 Uraspis uraspis KP-IMA
DBMF-M739 Uraspis uraspis KKJ-IMA
HQ149964 NPPF1125 Uraspis Persian Gulf Iran
FJ583378 Echeneis naucrates
0.02
