# Supplementary material for: DNA Barcoding Reveals Cryptic Diversity within Commercially Exploited Indo-Malay Carangidae (Teleosteii: Perciformes)
Source: PLoS One. 2012 Nov 29;7(11):e49623. doi: 10.1371/journal.pone.0049623 (PMC3510217; doi:10.1371/journal.pone.0049623)
Supplement: Table S1 — Specimen data and GenBank accession numbers used in this study. (DOC) [file pone.0049623.s005.doc]

**Table S1**. Specimen data and GenBank accession numbers used in this study.

| **Voucher number/ Museum ID** | **Locality** | **Year of collection** | **Species** | **GenBank accession number** | **BOLD sample ID** |
| --- | --- | --- | --- | --- | --- |
| UMTF03430 | Kuantan, Peninsular Malaysia (KN) | 2010 | *Alectis ciliaris* | JX261576 | DBMF-M182 |
| AHM12-01 | Hutan Melintang, Peninsular Malaysia (AHM) | 2009 | *Alectis ciliaris* | HQ560980 | DBMF-M47 |
| UMTF03977 | Tawau, Sabah (TW) | 2010 | *Alectis ciliaris* | JX261130 | DBMF-M729 |
| UMTF03429 | Kuantan, Peninsular Malaysia (KN) | 2010 | *Alectis ciliaris* | JX261128 | DBMF-M181 |
| UMTF03976 | Tawau, Sabah (TW) | 2010 | *Alectis ciliaris* | JX261420 | DBMF-M728 |
| UMTF03736 | Miri, Sarawak (MR) | 2010 | *Alectis ciliaris* | JX261045 | DBMF-M488 |
| UMTF03978 | Tawau, Sabah (TW) | 2010 | *Alectis ciliaris* | JX261555 | DBMF-M730 |
| UMTF03866 | Sandakan, Sabah (SDK) | 2010 | *Alectis ciliaris* | JX261543 | DBMF-M618 |
| NPPF1084 | Nayband National Park Coast, Iran | 2009 | *Alectis ciliaris* | HQ149787 | NPPF1084 |
| ITHJ1 | Nagasaki, Japan | 2005 | *Alectis ciliaris* | JF952663 | [ABFJ200-07.COI-5P](http://www.boldsystems.org/connectivity/specimenlookup.php?processid=ABFJ200-07.COI-5P) |
| ITHJ2 | Nagasaki, Japan | 2005 | *Alectis ciliaris* | JF952664 | [ABFJ201-07.COI-5P](http://www.boldsystems.org/connectivity/specimenlookup.php?processid=ABFJ200-07.COI-5P) |
| BW-A1472 | Queensland, Australia | 1998 | *Alectis ciliaris* | EF609280 | FOAC473-05 |
| N/A | India | 2006 | *Alectis ciliaris* | EU514500 | N/A |
| KK09-01 | Kuala Kedah, Peninsular Malaysia (KK) | 2009 | *Alectis indicus* | HQ560959 | DBMF-M19 |
| UMTF03975 | Tawau, Sabah (TW) | 2010 | *Alectis indicus* | JX261640 | DBMF-M727 |
| UMTF03806 | Kudat, Sabah (KDT) | 2010 | *Alectis indicus* | JX261288 | DBMF-M560 |
| UMTF03733 | Miri, Sarawak (MR) | 2010 | *Alectis indicus* | JX261127 | DBMF-M485 |
| AHM10-01 | Hutan Melintang, Peninsular Malaysia (AHM) | 2009 | *Alectis indicus* | HQ560978 | DBMF-M45 |
| UMTF03734 | Miri, Sarawak (MR) | 2010 | *Alectis indicus* | JX261178 | DBMF-M486 |
| UMTF03735 | Miri, Sarawak (MR) | 2010 | *Alectis indicus* | JX261217 | DBMF-M487 |
| UMTF03974 | Tawau, Sabah (TW) | 2010 | *Alectis indicus* | JX261340 | DBMF-M726 |
| UMTF03807 | Kudat, Sabah (KDT) | 2010 | *Alectis indicus* | JX261350 | DBMF-M561 |
| SK02-01 | Sekinchan, Peninsular Malaysia (SK) | 2009 | *Alectis indicus* | HQ560997 | DBMF-M68 |
| NPPF1062 | Nayband National Park Coast, Iran | 2009 | *Alectis indicus* | HQ149788 | NPPF1062 |
| NPPF1046 | Nayband National Park Coast, Iran | 2009 | *Alectis indicus* | HQ149789 | NPPF1046 |
| NPPF1012 | Nayband National Park Coast, Iran | 2009 | *Alectis indicus* | HQ149790 | NPPF1012 |
| UMTF03607 | Mukah, Sarawak (MKS) | 2010 | *Alepes djedaba* | JX261639 | DBMF-M359 |
| PN01-01 | Pontian, Peninsular Malaysia (PN) | 2009 | *Alepes djedaba* | HQ561009 | DBMF-M81 |
| UMTF03605 | Mukah, Sarawak (MKS) | 2010 | *Alepes djedaba* | JX261253 | DBMF-M357 |
| UMTF03822 | Kudat, Sabah (KDT) | 2010 | *Alepes djedaba* | JX261582 | DBMF-M574 |
| UMTF03823 | Kudat, Sabah (KDT) | 2010 | *Alepes djedaba* | JX261382 | DBMF-M575 |
| UMTF03824 | Kudat, Sabah (KDT) | 2010 | *Alepes djedaba* | JX261385 | DBMF-M576 |
| UMTF04091 | Kuala Perlis, Peninsular Malaysia (KP) |  | *Alepes djedaba* | JX261610 | DBMF-M843 |
| UMTF04090 | Kuala Perlis, Peninsular Malaysia (KP) | 2010 | *Alepes djedaba* | JX261246 | DBMF-M842 |
| UMTF03826 | Kudat, Sabah (KDT) | 2010 | *Alepes djedaba* | JX261567 | DBMF-M578 |
| UMTF04089 | Kuala Perlis, Peninsular Malaysia (KP) | 2010 | *Alepes djedaba* | JX261428 | DBMF-M841 |
| UMTF03606 | Mukah, Sarawak (MKS) | 2010 | *Alepes djedaba* | JX261206 | DBMF-M358 |
| SK04-01 | Sekinchan, Peninsular Malaysia (SK) | 2009 | *Alepes djedaba* | HQ560999 | DBMF-M70 |
| BP10-01 | Bagan Panchor, Peninsular Malaysia (BP) | 2009 | *Alepes djedaba* | HQ560965 | DBMF-M29 |
| HM04-01 | Hutan Melintang, Peninsular Malaysia (AHM) | 2009 | *Alepes djedaba* | HQ560972 | DBMF-M39 |
| UMTF04092 | Kuala Perlis, Peninsular Malaysia (KP) | 2010 | *Alepes djedaba* | JX261018 | DBMF-M844 |
| UMTF03943 | Tawau, Sabah (TW) | 2010 | *Alepes djedaba* | JX261550 | DBMF-M695 |
| UMTF03942 | Tawau, Sabah (TW) | 2010 | *Alepes djedaba* | JX261148 | DBMF-M694 |
| UMTF03941 | Tawau, Sabah (TW) | 2010 | *Alepes djedaba* | JX261607 | DBMF-M693 |
| UMTF03703 | Miri, Sarawak (MR) | 2010 | *Alepes djedaba* | JX261642 | DBMF-M455 |
| UMTF03704 | Miri, Sarawak (MR) | 2010 | *Alepes djedaba* | JX261023 | DBMF-M456 |
| UMTF03705 | Miri, Sarawak (MR) | 2010 | *Alepes djedaba* | JX261067 | DBMF-M457 |
| UMTF03707 | Miri, Sarawak (MR) | 2010 | *Alepes djedaba* | JX261500 | DBMF-M459 |
| UMTF03669 | Kuching, Sarawak (KC) | 2010 | *Alepes djedaba* | JX261362 | DBMF-M421 |
| UMTF03609 | Mukah, Sarawak (MKS) | 2010 | *Alepes djedaba* | JX261351 | DBMF-M361 |
| UMTF03668 | Kuching, Sarawak (KC) | 2010 | *Alepes djedaba* | JX261029 | DBMF-M420 |
| UMTF03608 | Mukah, Sarawak (MKS) | 2010 | *Alepes djedaba* | JX261156 | DBMF-M360 |
| UMTF03667 | Kuching, Sarawak (KC) | 2010 | *Alepes djedaba* | JX261293 | DBMF-M419 |
| UMTF03944 | Tawau, Sabah (TW) | 2010 | *Alepes djedaba* | JX261588 | DBMF-M696 |
| UMTF03945 | Tawau, Sabah (TW) | 2010 | *Alepes djedaba* | JX261122 | DBMF-M697 |
| UMTF03666 | Kuching, Sarawak (KC) | 2010 | *Alepes djedaba* | JX261077 | DBMF-M418 |
| ADC210.3-2 | Tugela Banks, KwaZulu-Natal, South Africa | 2004 | *Alepes djedaba* | JF492804 | [TZMSB089-04.COI-5P](http://www.boldsystems.org/connectivity/specimenlookup.php?processid=TZMSB089-04.COI-5P) |
| ADC210.3-1 | Tugela Banks, KwaZulu-Natal, South Africa | 2003 | *Alepes djedaba* | JF492805 | [TZMSB088-04.COI-5P](http://www.boldsystems.org/connectivity/specimenlookup.php?processid=TZMSB088-04.COI-5P) |
| ADC210.3-1 | Tugela Banks, KwaZulu-Natal, South Africa | 2003 | *Alepes djedaba* | JF492806 | [TZMSA390-04.COI-5P](http://www.boldsystems.org/connectivity/specimenlookup.php?processid=TZMSA390-04.COI-5P) |
| WL-M36 | Maharashtra, India | 2006 | *Alepes djedaba* | EF609497 | [WLIND036-07](http://www.boldsystems.org/connectivity/specimenlookup.php?processid=WLIND036-07) |
| WL-M35 | Maharashtra, India | 2006 | *Alepes djedaba* | EF609498 | [WLIND035-07](http://www.boldsystems.org/connectivity/specimenlookup.php?processid=WLIND035-07) |
| WL-M34 | Maharashtra, India | 2006 | *Alepes djedaba* | EF609499 | [WLIND034-07](http://www.boldsystems.org/connectivity/specimenlookup.php?processid=WLIND034-07) |
| WL-M33 | Maharashtra, India | 2006 | *Alepes djedaba* | EF609500 | [WLIND033-07](http://www.boldsystems.org/connectivity/specimenlookup.php?processid=WLIND033-07) |
| WL-M32 | Maharashtra, India | 2006 | *Alepes djedaba* | EF609501 | [WLIND032-07](http://www.boldsystems.org/connectivity/specimenlookup.php?processid=WLIND032-07) |
| UMTF03819 | Kudat, Sabah (KDT) | 2010 | *Alepes kleinii* | JX261594 | DBMF-M571 |
| UMTF04093 | Kuala Perlis, Peninsular Malaysia (KP) | 2010 | *Alepes kleinii* | JX261530 | DBMF-M845 |
| UMTF04094 | Kuala Perlis, Peninsular Malaysia (KP) | 2010 | *Alepes kleinii* | JX261344 | DBMF-M846 |
| UMTF03820 | Kudat, Sabah (KDT) | 2010 | *Alepes kleinii* | JX261396 | DBMF-M572 |
| UMTF03818 | Kudat, Sabah (KDT) | 2010 | *Alepes kleinii* | JX261049 | DBMF-M570 |
| UMTF03817 | Kudat, Sabah (KDT) | 2010 | *Alepes kleinii* | JX261103 | DBMF-M569 |
| UMTF03821 | Kudat, Sabah (KDT) | 2010 | *Alepes kleinii* | JX261284 | DBMF-M573 |
| UMTF04095 | Kuala Perlis, Peninsular Malaysia (KP) | 2010 | *Alepes kleinii* | JX261076 | DBMF-M847 |
| UMTF04097 | Kuala Perlis, Peninsular Malaysia (KP) | 2010 | *Alepes kleinii* | JX261039 | DBMF-M849 |
| UMTF04098 | Kuala Perlis, Peninsular Malaysia (KP) | 2010 | *Alepes kleinii* | JX261090 | DBMF-M850 |
| UMTF04096 | Kuala Perlis, Peninsular Malaysia (KP) | 2010 | *Alepes kleinii* | JX261086 | DBMF-M848 |
| UMTF03772 | Kudat, Sabah (KDT) | 2010 | *Alepes melanoptera* | JX261407 | DBMF-M524 |
| KK06-01 | Kota Kinabalu, Sabah (KKJ) | 2009 | *Alepes melanoptera* | HQ560956 | DBMF-M16 |
| BP09-01 | Bagan Panchor, Peninsular Malaysia (BP) | 2009 | *Alepes melanoptera* | HQ560964 | DBMF-M28 |
| HM07-01 | Hutan Melintang, Peninsular Malaysia (AHM) | 2009 | *Alepes melanoptera* | HQ560975 | DBMF-M42 |
| KSB01-01 | Kuala Sungai Besar, Peninsular Malaysia (KSB) | 2009 | *Alepes melanoptera* | HQ560986 | DBMF-M53 |
| SK05-01 | Sekinchan, Peninsular Malaysia (SK) | 2009 | *Alepes melanoptera* | HQ561000 | DBMF-M71 |
| PN02-01 | Pontian, Peninsular Malaysia (PN) | 2009 | *Alepes melanoptera* | HQ561010 | DBMF-M82 |
| UMTF03770 | Kudat, Sabah (KDT) | 2010 | *Alepes melanoptera* | JX261624 | DBMF-M522 |
| UMTF03771 | Kudat, Sabah (KDT) | 2010 | *Alepes melanoptera* | JX261457 | DBMF-M523 |
| UMTF03956 | Tawau, Sabah (TW) | 2010 | *Alepes melanoptera* | JX261561 | DBMF-M708 |
| UMTF03957 | Tawau, Sabah (TW) | 2010 | *Alepes melanoptera* | JX261267 | DBMF-M709 |
| UMTF03959 | Tawau, Sabah (TW) | 2010 | *Alepes melanoptera* | JX261647 | DBMF-M711 |
| UMTF03960 | Tawau, Sabah (TW) | 2010 | *Alepes melanoptera* | JX261161 | DBMF-M712 |
| UMTF04015 | Kota Kinabalu, Sabah (KKJ) | 2010 | *Alepes melanoptera* | JX261047 | DBMF-M767 |
| UMTF04017 | Kota Kinabalu, Sabah (KKJ) | 2010 | *Alepes melanoptera* | JX261188 | DBMF-M769 |
| UMTF03887 | Sandakan, Sabah (SDK) | 2010 | *Alepes vari* | JX261282 | DBMF-M639 |
| UMTF03825 | Kudat, Sabah (KDT) | 2010 | *Alepes vari* | JX261228 | DBMF-M577 |
| UMTF03531 | Pulau Kambing, Peninsular Malaysia (PK) | 2010 | *Alepes vari* | JX261520 | DBMF-M283 |
| UMTF03532 | Pulau Kambing, Peninsular Malaysia (PK) | 2010 | *Alepes vari* | JX261010 | DBMF-M284 |
| UMTF03534 | Pulau Kambing, Peninsular Malaysia (PK) | 2010 | *Alepes vari* | JX261494 | DBMF-M286 |
| UMTF03700 | Miri, Sarawak (MR) | 2010 | *Alepes vari* | JX261192 | DBMF-M452 |
| UMTF03886 | Sandakan, Sabah (SDK) | 2010 | *Alepes vari* | JX261379 | DBMF-M638 |
| UMTF03699 | Miri, Sarawak (MR) | 2010 | *Alepes vari* | JX261475 | DBMF-M451 |
| UMTF03883 | Sandakan, Sabah (SDK) | 2010 | *Alepes vari* | JX261234 | DBMF-M635 |
| UMTF03702 | Miri, Sabah (MR) | 2010 | *Alepes vari* | JX261434 | DBMF-M454 |
| KP02-01 | Kuala Perlis, Peninsular Malaysia (KP) | 2009 | *Alepes vari* | HQ560946 | DBMF-M2 |
| UMTF03701 | Miri, Sarawak (MR) | 2010 | *Alepes vari* | JX261644 | DBMF-M453 |
| UMTF03949 | Tawau, Sabah (TW) | 2010 | *Atropus atropos* | JX261095 | DBMF-M701 |
| UMTF03948 | Tawau, Sabah (TW) | 2010 | *Atropus atropos* | JX261368 | DBMF-M700 |
| UMTF03946 | Tawau, Sabah (TW) | 2010 | *Atropus atropos* | JX261411 | DBMF-M698 |
| UMTF03632 | Mukah, Sarawak (MKS) | 2010 | *Atropus atropos* | JX261181 | DBMF-M384 |
| UMTF03631 | Mukah, Sarawak (MKS) | 2010 | *Atropus atropos* | JX261436 | DBMF-M383 |
| UMTF03630 | Mukah, Sarawak (MKS) | 2010 | *Atropus atropos* | JX261352 | DBMF-M382 |
| UMTF03629 | Mukah, Sarawak (MKS) | 2010 | *Atropus atropos* | JX261490 | DBMF-M381 |
| UMTF03947 | Tawau, Sabah (TW) | 2010 | *Atropus atropos* | JX261357 | DBMF-M699 |
| KSB05-01 | Kuala Sg. Besar, Peninsular Malaysia (KSB) | 2009 | *Atropus atropos* | HQ560989 | DBMF-M57 |
| SK03-01 | Sekinchan, Peninsular Malaysia (SK) | 2009 | *Atropus atropos* | HQ560998 | DBMF-M69 |
| UMTF03950 | Tawau, Sabah (TW) | 2010 | *Atropus atropos* | JX261172 | DBMF-M702 |
| UMTF03633 | Mukah, Sarawak (MKS) | 2010 | *Atropus atropos* | JX261287 | DBMF-M385 |
| HM05-01 | Hutan Melintang, Peninsular Malaysia (AHM) | 2009 | *Atropus atropos* | HQ560973 | DBMF-M40 |
| WL-M24 | Maharashtra, India | 2006 | *Atropus atropos* | EF609502 | [WLIND024-07](http://www.boldsystems.org/connectivity/specimenlookup.php?processid=WLIND024-07) |
| WL-M23 | Maharashtra, India | 2006 | *Atropus atropos* | EF609503 | [WLIND023-07](http://www.boldsystems.org/connectivity/specimenlookup.php?processid=WLIND023-07) |
| WL-M22 | Maharashtra, India | 2006 | *Atropus atropos* | EF609504 | [WLIND022-07](http://www.boldsystems.org/connectivity/specimenlookup.php?processid=WLIND022-07) |
| WL-M21 | Maharashtra, India | 2006 | *Atropus atropos* | EF609505 | [WLIND021-07](http://www.boldsystems.org/connectivity/specimenlookup.php?processid=WLIND021-07) |
| WL-M20 | Maharashtra, India | 2006 | *Atropus atropos* | EF609506 | [WLIND020-07](http://www.boldsystems.org/connectivity/specimenlookup.php?processid=WLIND020-07) |
| UMTF03518 | Pulau Kambing, Peninsular Malaysia (PK) | 2010 | *Atule mate* | JX261063 | DBMF-M270 |
| UMTF03610 | Mukah, Sarawak (MKS) | 2010 | *Atule mate* | JX261194 | DBMF-M362 |
| UMTF03611 | Mukah, Sarawak (MKS | 2010 | *Atule mate* | JX261349 | DBMF-M363 |
| UMTF03612 | Mukah, Sarawak (MKS) | 2010 | *Atule mate* | JX261612 | DBMF-M364 |
| UMTF03613 | Mukah, Sarawak (MKS) | 2010 | *Atule mate* | JX261429 | DBMF-M365 |
| UMTF03614 | Mukah, Sarawak (MKS) | 2010 | *Atule mate* | JX261546 | DBMF-M366 |
| UMTF03763 | Kudat, Sabah (KDT) | 2010 | *Atule mate* | JX261535 | DBMF-M515 |
| UMTF03762 | Kudat, Sabah (KDT) | 2010 | *Atule mate* | JX261507 | DBMF-M514 |
| UMTF03761 | Kudat, Sabah (KDT) | 2010 | *Atule mate* | JX261484 | DBMF-M513 |
| UMTF03760 | Kudat, Sabah (KDT) | 2010 | *Atule mate* | JX261056 | DBMF-M512 |
| HM08-01 | Hutan Melintang, Peninsular Malaysia (AHM) | 2009 | *Atule mate* | HQ560976 | DBMF-M43 |
| KSB07-01 | Kuala Sungai Besar, Peninsular Malaysia (KSB) | 2009 | *Atule mate* | HQ560990 | DBMF-M59 |
| SB02-01 | Kuala Sungai Baru, Peninsular Malaysia (SB) | 2009 | *Atule mate* | HQ561006 | DBMF-M78 |
| PN05-01 | Pontian, Peninsular Malaysia (PN) | 2009 | *Atule mate* | HQ561013 | DBMF-M85 |
| T02-01 | Tumpat, Peninsular Malaysia (T) | 2009 | *Atule mate* | HQ561014 | DBMF-M86 |
| KBT06-01 | Kuala Besar, Peninsular Malaysia (KBT) | 2009 | *Atule mate* | HQ561015 | DBMF-M87 |
| TB05-01 | Tok Bali, Peninsular Malaysia (TB) | 2009 | *Atule mate* | HQ561016 | DBMF-M88 |
| KD03-01 | Kuala Dungun , Peninsular Malaysia (KD) | 2009 | *Atule mate* | HQ561017 | DBMF-M90 |
| KN04-01 | Kuantan, Peninsular Malaysia (KN) | 2009 | *Atule mate* | HQ561018 | DBMF-M91 |
| KPG06-01 | Kuala Pahang, Peninsular Malaysia (KPG) | 2009 | *Atule mate* | HQ561019 | DBMF-M92 |
| TG06-01 | Tanjung Gemuk, Peninsular Malaysia (TG) | 2009 | *Atule mate* | HQ561020 | DBMF-M93 |
| MG06-01 | Mersing, Peninsular Malaysia (MG) | 2009 | *Atule mate* | HQ561021 | DBMF-M94 |
| TS05-01 | Tanjung Sedili, Peninsular Malaysia (TS) | 2009 | *Atule mate* | HQ561022 | DBMF-M95 |
| UMTF04063 | Kuala Perlis, Peninsular Malaysia (KP) | 2010 | *Atule mate* | JX261597 | DBMF-M815 |
| UMTF04062 | Kuala Perlis, Peninsular Malaysia (KP) | 2010 | *Atule mate* | JX261075 | DBMF-M814 |
| UMTF04061 | Kuala Perlis, Peninsular Malaysia (KP) | 2010 | *Atule mate* | JX261226 | DBMF-M813 |
| UMTF04060 | Kuala Perlis, Peninsular Malaysia (KP) | 2010 | *Atule mate* | JX261474 | DBMF-M812 |
| UMTF04059 | Kuala Perlis, Peninsular Malaysia (KP) | 2010 | *Atule mate* | JX261289 | DBMF-M811 |
| UMTF03407 | Tanjung Sedili, Peninsular Malaysia (TS) | 2010 | *Atule mate* | JX261378 | DBMF-M159 |
| UMTF03408 | Tanjung Sedili, Peninsular Malaysia (TS) | 2010 | *Atule mate* | JX261302 | DBMF-M160 |
| UMTF03409 | Tanjung Sedili, Peninsular Malaysia (TS) | 2010 | *Atule mate* | JX261254 | DBMF-M161 |
| UMTF03431 | Kuantan, Peninsular Malaysia (KN) | 2010 | *Atule mate* | JX261512 | DBMF-M183 |
| UMTF03411 | Tanjung Sedili, Peninsular Malaysia (TS) | 2010 | *Atule mate* | JX261413 | DBMF-M163 |
| UMTF03432 | Kuantan, Peninsular Malaysia (KN) | 2010 | *Atule mate* | JX261370 | DBMF-M184 |
| UMTF03433 | Kuantan, Peninsular Malaysia (KN) | 2010 | *Atule mate* | JX261012 | DBMF-M185 |
| UMTF03434 | Kuantan, Peninsular Malaysia (KN) | 2010 | *Atule mate* | JX261405 | DBMF-M186 |
| UMTF03435 | Kuantan, Peninsular Malaysia (KN) | 2010 | *Atule mate* | JX261233 | DBMF-M187 |
| KP08-01 | Kuala Perlis, Peninsular Malaysia (KP) | 2009 | *Atule mate* | HQ560949 | DBMF-M8 |
| UMTF03994 | Kota Kinabalu, Sabah (KKJ) | 2010 | *Atule mate* | JX261261 | DBMF-M746 |
| UMTF03683 | Miri, Sarawak (MR) | 2010 | *Atule mate* | JX261446 | DBMF-M435 |
| UMTF03682 | Miri, Sarawak (MR) | 2010 | *Atule mate* | JX261548 | DBMF-M434 |
| UMTF03681 | Miri, Sarawak (MR) | 2010 | *Atule mate* | JX261222 | DBMF-M433 |
| UMTF03680 | Miri, Sarawak (MR) | 2010 | *Atule mate* | JX261094 | DBMF-M432 |
| UMTF03679 | Miri, Sarawak (MR) | 2010 | *Atule mate* | JX261035 | DBMF-M431 |
| UMTF03456 | Kuala Dungun, Peninsular Malaysia (KD) | 2010 | *Atule mate* | JX261533 | DBMF-M208 |
| UMTF03457 | Kuala Dungun, Peninsular Malaysia (KD) | 2010 | *Atule mate* | JX261249 | DBMF-M209 |
| UMTF03458 | Kuala Dungun, Peninsular Malaysia (KD) | 2010 | *Atule mate* | JX261545 | DBMF-M210 |
| UMTF03459 | Kuala Dungun, Peninsular Malaysia (KD) | 2010 | *Atule mate* | JX261437 | DBMF-M211 |
| UMTF03993 | Kota Kinabalu, Sabah (KKJ) | 2010 | *Atule mate* | JX261501 | DBMF-M745 |
| UMTF03992 | Kota Kinabalu, Sabah (KKJ) | 2010 | *Atule mate* | JX261410 | DBMF-M744 |
| UMTF03991 | Kota Kinabalu, Sabah (KKJ) | 2010 | *Atule mate* | JX261200 | DBMF-M743 |
| UMTF03990 | Kota Kinabalu, Sabah (KKJ) | 2010 | *Atule mate* | JX261614 | DBMF-M742 |
| UMTF03867 | Sandakan, Sabah (SDK) | 2010 | *Atule mate* | JX261412 | DBMF-M619 |
| UMTF03764 | Kudat, Sabah (KDT) | 2010 | *Atule mate* | JX261401 | DBMF-M516 |
| UMTF03515 | Pulau Kambing, Peninsular Malaysia (PK) | 2010 | *Atule mate* | JX261635 | DBMF-M267 |
| UMTF03516 | Pulau Kambing, Peninsular Malaysia (PK) | 2010 | *Atule mate* | JX261280 | DBMF-M268 |
| UMTF03517 | Pulau Kambing, Peninsular Malaysia (PK) | 2010 | *Atule mate* | JX261578 | DBMF-M269 |
| UMTF03519 | Pulau Kambing, Peninsular Malaysia (PK) | 2010 | *Atule mate* | JX261633 | DBMF-M271 |
| KK04-01 | Kuala Kedah, Peninsular Malaysia (KK) | 2009 | *Atule mate* | HQ560955 | DBMF-M14 |
| BP08-01 | Bagan Panchor, Peninsular Malaysia (BP) | 2009 | *Atule mate* | HQ560963 | DBMF-M27 |
| UMTF03655 | Kuching, Sarawak (KC) | 2010 | *Atule mate* | JX261531 | DBMF-M407 |
| UMTF03654 | Kuching, Sarawak (KC) | 2010 | *Atule mate* | JX261445 | DBMF-M406 |
| UMTF03653 | Kuching, Sarawak (KC) | 2010 | *Atule mate* | JX261218 | DBMF-M405 |
| UMTF03652 | Kuching, Sarawak (KC) | 2010 | *Atule mate* | JX261374 | DBMF-M404 |
| UMTF03570 | Tok Bali, Peninsular Malaysia (TB) | 2010 | *Atule mate* | JX261505 | DBMF-M322 |
| UMTF03571 | Tok Bali, Peninsular Malaysia (TB) | 2010 | *Atule mate* | JX261419 | DBMF-M323 |
| UMTF03574 | Tok Bali, Peninsular Malaysia (TB) | 2010 | *Atule mate* | JX261557 | DBMF-M326 |
| MBCSC:Z711217 | China: South China Sea | 2007 | *Atule mate* | EU595060 | [FSCS551-07](http://www.boldsystems.org/connectivity/specimenlookup.php?processid=FSCS551-07) |
| MBCSC:Z711007 | China: South China Sea | 2007 | *Atule mate* | EU595063 | [FSCS337-07](http://www.boldsystems.org/connectivity/specimenlookup.php?processid=FSCS337-07) |
| MBCSC:Z711003 | China: South China Sea | 2007 | *Atule mate* | EU595067 | [FSCS333-07](http://www.boldsystems.org/connectivity/specimenlookup.php?processid=FSCS333-07) |
| MBCSC:Z711004 | China: South China Sea | 2007 | *Atule mate* | EU595066 | [FSCS334-07](http://www.boldsystems.org/connectivity/specimenlookup.php?processid=FSCS334-07) |
| MBCSC:Z711005 | China: South China Sea | 2007 | *Atule mate* | EU595065 | [FSCS335-07](http://www.boldsystems.org/connectivity/specimenlookup.php?processid=FSCS335-07) |
| MBCSC:Z711006 | China: South China Sea | 2007 | *Atule mate* | EU595064 | [FSCS336-07](http://www.boldsystems.org/connectivity/specimenlookup.php?processid=FSCS336-07) |
| MBCSC:Z711008 | China: South China Sea | 2007 | *Atule mate* | EU595062 | [FSCS338-07](http://www.boldsystems.org/connectivity/specimenlookup.php?processid=FSCS338-07) |
| MBCSC:Z711219 | China: South China Sea | 2007 | *Atule mate* | EU595058 | [FSCS553-07](http://www.boldsystems.org/connectivity/specimenlookup.php?processid=FSCS553-07) |
| MBCSC:Z711216 | China: South China Sea | 2007 | *Atule mate* | EU595061 | [FSCS550-07](http://www.boldsystems.org/connectivity/specimenlookup.php?processid=FSCS550-07) |
| MBCSC:Z711218 | China: South China Sea | 2007 | *Atule mate* | EU595059 | [FSCS552-07](http://www.boldsystems.org/connectivity/specimenlookup.php?processid=FSCS552-07) |
| GD 9083018 | China | 2006 | *Atule mate* | EF607335 | [FSCS257-06](http://www.boldsystems.org/connectivity/specimenlookup.php?processid=FSCS257-06) |
| BW-A1465 | Queensland, Australia | 1998 | *Atule mate* | EF609293 | [FOAC466-05](http://www.boldsystems.org/connectivity/specimenlookup.php?processid=FOAC466-05) |
| NPPF1180 | Nayband National Park Coast, Iran | 2009 | *Atule mate* | HQ149797 | NPPF1180 |
| NPPF1073 | Nayband National Park Coast, Iran | 2009 | *Atule mate* | HQ149798 | NPPF1073 |
| NPPF1072 | Nayband National Park Coast, Iran | 2009 | *Atule mate* | HQ149799 | NPPF1072 |
| NPPF1071 | Nayband National Park Coast, Iran | 2009 | *Atule mate* | HQ149800 | NPPF1071 |
| NPPF1038 | Nayband National Park Coast, Iran | 2009 | *Atule mate* | HQ149801 | NPPF1038 |
| MBCSC:ZC I07339 | China: South China Sea | 2007 | *Atule mate* | FJ237967 | [FSCS764-08](http://www.boldsystems.org/connectivity/specimenlookup.php?processid=FSCS764-08) |
| MBCSC:ZC I07332 | China: South China Sea | 2007 | *Atule mate* | FJ237968 | [FSCS757-08](http://www.boldsystems.org/connectivity/specimenlookup.php?processid=FSCS757-08) |
| MBCSC:ZC I07329 | China: South China Sea | 2007 | *Atule mate* | FJ237969 | [FSCS754-08](http://www.boldsystems.org/connectivity/specimenlookup.php?processid=FSCS754-08) |
| UMTF03420 | Kuantan, Peninsular Malaysia (KN) | 2010 | *Carangoides bajad* | JX261394 | DBMF-M172 |
| UMTF03585 | Tok Bali, Peninsular Malaysia (TB) | 2010 | *Carangoides bajad* | JX261219 | DBMF-M337 |
| UMTF03586 | Tok Bali, Peninsular Malaysia (TB) | 2010 | *Carangoides bajad* | JX261131 | DBMF-M338 |
| UMTF03419 | Kuantan, Peninsular Malaysia (KN) | 2010 | *Carangoides bajad* | JX261171 | DBMF-M171 |
| UMTF04011 | Kota Kinabalu, Sabah (KKJ) | 2010 | *Carangoides bajad* | JX261098 | DBMF-M763 |
| UMTF04012 | Kota Kinabalu, Sabah (KKJ) | 2010 | *Carangoides bajad* | JX261124 | DBMF-M764 |
| UMTF04013 | Kota Kinabalu, Sabah (KKJ) | 2010 | *Carangoides bajad* | JX261109 | DBMF-M765 |
| UMTF03713 | Miri, Sarawak (MR) | 2010 | *Carangoides bajad* | JX261526 | DBMF-M465 |
| UMTF03714 | Miri, Sarawak (MR) | 2010 | *Carangoides bajad* | JX261416 | DBMF-M466 |
| UMTF03715 | Miri, Sarawak (MR) | 2010 | *Carangoides bajad* | JX261021 | DBMF-M467 |
| UMTF03775 | Kudat, Sabah (KDT) | 2010 | *Carangoides bajad* | JX261641 | DBMF-M527 |
| UMTF03777 | Kudat, Sabah (KDT) | 2010 | *Carangoides bajad* | JX261263 | DBMF-M529 |
| UMTF03776 | Kudat, Sabah (KDT) | 2010 | *Carangoides bajad* | JX261510 | DBMF-M528 |
| UMTF03677 | Kuching, Sarawak (KC) | 2010 | *Carangoides bajad* | JX261273 | DBMF-M429 |
| UMTF04010 | Kota Kinabalu, Sabah (KKJ) | 2010 | *Carangoides bajad* | JX261159 | DBMF-M762 |
| UMTF03587 | Tok Bali, Peninsular Malaysia (TB) | 2010 | *Carangoides bajad* | JX261136 | DBMF-M339 |
| UMTF03588 | Tok Bali, Peninsular Malaysia (TB) | 2010 | *Carangoides bajad* | JX261424 | DBMF-M340 |
| UMTF03589 | Tok Bali, Peninsular Malaysia (TB) | 2010 | *Carangoides bajad* | JX261108 | DBMF-M341 |
| UMTF03779 | Kudat, Sabah (KDT) | 2010 | *Carangoides bajad* | JX261266 | DBMF-M531 |
| UMTF03778 | Kudat, Sabah (KDT) | 2010 | *Carangoides bajad* | JX261345 | DBMF-M530 |
| UMTF03717 | Miri, Sarawak (MR) | 2010 | *Carangoides bajad* | JX261144 | DBMF-M469 |
| UMTF03423 | Kuantan, Peninsular Malaysia (KN) | 2010 | *Carangoides bajad* | JX261460 | DBMF-M175 |
| UMTF03422 | Kuantan, Peninsular Malaysia (KN) | 2010 | *Carangoides bajad* | JX261225 | DBMF-M174 |
| UMTF03828 | Sandakan, Sabah (SDK) | 2010 | *Carangoides bajad* | JX261489 | DBMF-M580 |
| UMTF04014 | Kota Kinabalu, Sabah (KKJ) | 2010 | *Carangoides bajad* | JX261593 | DBMF-M766 |
| UMTF03676 | Kuching, Sarawak (KC) | 2010 | *Carangoides bajad* | JX261276 | DBMF-M428 |
| UMTF04047 | Kota Kinabalu, Sabah (KKJ) | 2010 | *Carangoides chrysophrys* | JX261153 | DBMF-M799 |
| UMTF03636 | Mukah, Sarawak (MKS) | 2010 | *Carangoides chrysophrys* | JX261573 | DBMF-M388 |
| KK07-01 | Kuala Kedah, Peninsular Malaysia (KK) | 2009 | *Carangoides chrysophrys* | HQ560957 | DBMF-M17 |
| UMTF04044 | Kota Kinabalu, Sabah (KKJ) | 2010 | *Carangoides chrysophrys* | JX261517 | DBMF-M796 |
| UMTF04045 | Kota Kinabalu, Sabah (KKJ) | 2010 | *Carangoides chrysophrys* | JX261622 | DBMF-M797 |
| UMTF04046 | Kota Kinabalu, Sabah (KKJ) | 2010 | *Carangoides chrysophrys* | JX261118 | DBMF-M798 |
| UMTF04048 | Kota Kinabalu, Sabah (KKJ) | 2010 | *Carangoides chrysophrys* | JX261560 | DBMF-M800 |
| UMTF03635 | Mukah, Sarawak (MKS) | 2010 | *Carangoides chrysophrys* | JX261146 | DBMF-M387 |
| UMTF03841 | Sandakan, Sabah (SDK) | 2010 | *Carangoides chrysophrys* | JX261229 | DBMF-M593 |
| UMTF03840 | Sandakan, Sabah (SDK) | 2010 | *Carangoides chrysophrys* | JX261142 | DBMF-M592 |
| UMTF03839 | Sandakan, Sabah (SDK) | 2010 | *Carangoides chrysophrys* | JX261024 | DBMF-M591 |
| UMTF03838 | Sandakan, Sabah (SDK) | 2010 | *Carangoides chrysophrys* | JX261608 | DBMF-M590 |
| UMTF03837 | Sandakan, Sabah (SDK) | 2010 | *Carangoides chrysophrys* | JX261575 | DBMF-M589 |
| UMTF03634 | Mukah, Sarawak (MKS) | 2010 | *Carangoides chrysophrys* | JX261158 | DBMF-M386 |
| UMTF03802 | Kudat, Sabah (KDT) | 2010 | *Carangoides chrysophrys* | JX261570 | DBMF-M554 |
| UMTF03637 | Mukah, Sarawak (MKS) | 2010 | *Carangoides chrysophrys* | JX261341 | DBMF-M389 |
| UMTF03966 | Tawau, Sabah (TW) | 2010 | *Carangoides chrysophrys* | JX261034 | DBMF-M718 |
| UMTF03801 | Kudat, Sabah (KDT) | 2010 | *Carangoides chrysophrys* | JX261595 | DBMF-M553 |
| UMTF03800 | Kudat, Sabah (KDT) | 2010 | *Carangoides chrysophrys* | JX261399 | DBMF-M552 |
| UMTF03972 | Tawau, Sabah (TW) | 2010 | *Carangoides dinema* | JX261552 | DBMF-M724 |
| UMTF03971 | Tawau, Sabah (TW) | 2010 | *Carangoides dinema* | JX261041 | DBMF-M723 |
| UMTF03903 | Semporna, Sabah (SMP) | 2010 | *Carangoides dinema* | JX261235 | DBMF-M655 |
| UMTF03902 | Semporna, Sabah (SMP) | 2010 | *Carangoides dinema* | JX261508 | DBMF-M654 |
| UMTF03904 | Semporna, Sabah (SMP) | 2010 | *Carangoides dinema* | JX261007 | DBMF-M656 |
| UMTF03889 | Sandakan, Sabah (SDK) | 2010 | *Carangoides dinema* | JX261328 | DBMF-M641 |
| AHM14-01 | Hutan Melintang, Peninsular Malaysia (AHM) | 2009 | *Carangoides ferdau* | HQ560982 | DBMF-M49 |
| BP01-01 | Bagan Panchor, Peninsular Malaysia (BP) | 2009 | *Carangoides ferdau* | HQ560960 | DBMF-M20 |
| MBIO1835.4 | French Polynesia: Society Islands, Moorea | 2006 | *Carangoides ferdau* | JQ431538 | [MBFB018-07.COI-5P](http://www.boldsystems.org/connectivity/specimenlookup.php?processid=MBFB018-07.COI-5P) |
| UMTF03493 | Kuala Dungun, Peninsular Malaysia (KD) | 2010 | *Carangoides fulvoguttatus* | JX261364 | DBMF-M245 |
| UMTF03492 | Kuala Dungun, Peninsular Malaysia (KD) | 2010 | *Carangoides fulvoguttatus* | JX261174 | DBMF-M244 |
| UMTF03491 | Kuala Dungun, Peninsular Malaysia (KD) | 2010 | *Carangoides fulvoguttatus* | JX261084 | DBMF-M243 |
| BW-A1474 | Queensland, Australia | 1998 | *Carangoides fulvoguttatus* | EF609302 | [FOAC475-05](http://www.boldsystems.org/connectivity/specimenlookup.php?processid=FOAC475-05) |
| ADC08 Smith 210.11 #6 | Mozambique: Pomene | 2008 | *Carangoides fulvoguttatus* | JF493025 | [DSFSE764-08.COI-5P](http://www.boldsystems.org/connectivity/specimenlookup.php?processid=DSFSE764-08.COI-5P) |
| ADC08 Smith 210.11 #5 | Mozambique: Pomene | 2008 | *Carangoides fulvoguttatus* | JF493026 | [DSFSE765-08.COI-5P](http://www.boldsystems.org/connectivity/specimenlookup.php?processid=DSFSE765-08.COI-5P) |
| ADC08 Smith 210.11 #2 | Mozambique: Pomene | 2008 | *Carangoides fulvoguttatus* | JF493027 | [DSFSE783-08.COI-5P](http://www.boldsystems.org/connectivity/specimenlookup.php?processid=DSFSE783-08.COI-5P) |
| ADC08 Smith 210.11 #3 | Mozambique: Pomene | 2008 | *Carangoides fulvoguttatus* | JF493028 | [DSFSE782-08.COI-5P](http://www.boldsystems.org/connectivity/specimenlookup.php?processid=DSFSE782-08.COI-5P) |
| ADC08 Smith 210.11 #4 | Mozambique: Pomene | 2008 | *Carangoides fulvoguttatus* | JF493029 | [DSFSE781-08.COI-5P](http://www.boldsystems.org/connectivity/specimenlookup.php?processid=DSFSE781-08.COI-5P) |
| BP03-01 | Bagan Panchor, Peninsular Malaysia (BP) | 2009 | *Carangoides gymnostethus* | HQ560962 | DBMF-M22 |
| UMTF03369 | Mersing, Peninsular Malaysia (MG) | 2010 | *Carangoides hedlandensis* | JX261275 | DBMF-M121 |
| UMTF03370 | Mersing, Peninsular Malaysia (MG) | 2010 | *Carangoides hedlandensis* | JX261305 | DBMF-M122 |
| UMTF03371 | Mersing, Peninsular Malaysia (MG) | 2010 | *Carangoides hedlandensis* | JX261372 | DBMF-M123 |
| UMTF03845 | Sandakan, Sabah (SDK) | 2010 | *Carangoides malabaricus* | JX261632 | DBMF-M597 |
| UMTF04041 | Kota Kinabalu, Sabah (KKJ) | 2010 | *Carangoides malabaricus* | JX261329 | DBMF-M793 |
| UMTF03788 | Kudat, Sabah (KDT) | 2010 | *Carangoides malabaricus* | JX261061 | DBMF-M540 |
| UMTF03786 | Kudat, Sabah (KDT) | 2010 | *Carangoides malabaricus* | JX261092 | DBMF-M538 |
| UMTF03969 | Tawau, Sabah (TW) | 2010 | *Carangoides malabaricus* | JX261190 | DBMF-M721 |
| UMTF03486 | Kuala Dungun, Peninsular Malaysia (KD) | 2010 | *Carangoides malabaricus* | JX261643 | DBMF-M238 |
| UMTF03487 | Kuala Dungun, Peninsular Malaysia (KD) | 2010 | *Carangoides malabaricus* | JX261011 | DBMF-M239 |
| UMTF03488 | Kuala Dungun, Peninsular Malaysia (KD) | 2010 | *Carangoides malabaricus* | JX261637 | DBMF-M240 |
| UMTF03489 | Kuala Dungun, Peninsular Malaysia (KD) | 2010 | *Carangoides malabaricus* | JX261618 | DBMF-M241 |
| UMTF03490 | Kuala Dungun, Peninsular Malaysia (KD) | 2010 | *Carangoides malabaricus* | JX261111 | DBMF-M242 |
| UMTF04043 | Kota Kinabalu, Sabah (KKJ) | 2010 | *Carangoides malabaricus* | JX261046 | DBMF-M795 |
| UMTF03842 | Sandakan, Sabah (SDK) | 2010 | *Carangoides malabaricus* | JX261369 | DBMF-M594 |
| UMTF03785 | Kudat, Sabah (KDT) | 2010 | *Carangoides malabaricus* | JX261346 | DBMF-M537 |
| AHM13-01 | Hutan Melintang, Peninsular Malaysia (AHM) | 2009 | *Carangoides malabaricus* | HQ560981 | DBMF-M48 |
| UMTF03550 | Kuala Besut, Peninsular Malaysia (KB) | 2010 | *Carangoides malabaricus* | JX261409 | DBMF-M302 |
| UMTF03553 | Kuala Besut, Peninsular Malaysia (KB) | 2010 | *Carangoides malabaricus* | JX261471 | DBMF-M305 |
| UMTF03554 | Kuala Besut, Peninsular Malaysia (KB) | 2010 | *Carangoides malabaricus* | JX261581 | DBMF-M306 |
| UMTF03647 | Mukah, Sarawak (MKS) | 2010 | *Carangoides malabaricus* | JX261290 | DBMF-M399 |
| UMTF03646 | Mukah, Sarawak (MKS) | 2010 | *Carangoides malabaricus* | JX261551 | DBMF-M398 |
| UMTF03708 | Miri, Sarawak (MR) | 2010 | *Carangoides malabaricus* | JX261406 | DBMF-M460 |
| UMTF03710 | Miri, Sarawak (MR) | 2010 | *Carangoides malabaricus* | JX260999 | DBMF-M462 |
| UMTF03711 | Miri, Sarawak (MR) | 2010 | *Carangoides malabaricus* | JX261135 | DBMF-M463 |
| UMTF03712 | Miri, Sarawak (MR) | 2010 | *Carangoides malabaricus* | JX261600 | DBMF-M464 |
| UMTF03424 | Kuantan, Peninsular Malaysia (KN) | 2010 | *Carangoides malabaricus* | JX261270 | DBMF-M176 |
| UMTF03425 | Kuantan, Peninsular Malaysia (KN) | 2010 | *Carangoides malabaricus* | JX261149 | DBMF-M177 |
| UMTF03426 | Kuantan, Peninsular Malaysia (KN) | 2010 | *Carangoides malabaricus* | JX261155 | DBMF-M178 |
| UMTF03427 | Kuantan, Peninsular Malaysia (KN) | 2010 | *Carangoides malabaricus* | JX261113 | DBMF-M179 |
| UMTF03428 | Kuantan, Peninsular Malaysia (KN) | 2010 | *Carangoides malabaricus* | JX261173 | DBMF-M180 |
| UMTF03843 | Sandakan, Sabah (SDK) | 2010 | *Carangoides malabaricus* | JX261133 | DBMF-M595 |
| UMTF04040 | Kota Kinabalu, Sabah (KKJ) | 2010 | *Carangoides malabaricus* | JX261469 | DBMF-M792 |
| UMTF03844 | Sandakan, Sabah (SDK) | 2010 | *Carangoides malabaricus* | JX261473 | DBMF-M596 |
| UMTF03846 | Sandakan, Sabah (SDK) | 2010 | *Carangoides malabaricus* | JX261466 | DBMF-M598 |
| UMTF 04039 | Kota Kinabalu, Sabah (KKJ) | 2010 | *Carangoides malabaricus* | [JX261004](http://www.ncbi.nlm.nih.gov/nuccore/JX261004) | DBMF-M791 |
| UMTF03953 | Tawau, Sabah (TW) | 2010 | *Caranx ignobilis* | JX261044 | DBMF-M705 |
| UMTF03812 | Kudat, Sabah (KDT) | 2010 | *Caranx ignobilis* | JX261366 | DBMF-M566 |
| UMTF03810 | Kudat, Sabah (KDT) | 2010 | *Caranx ignobilis* | JX261065 | DBMF-M564 |
| UMTF03811 | Kudat, Sabah (KDT) | 2010 | *Caranx ignobilis* | JX261360 | DBMF-M565 |
| UMTF03809 | Kudat, Sabah (KDT) | 2010 | *Caranx ignobilis* | JX261433 | DBMF-M563 |
| UMTF03952 | Tawau, Sabah (TW) | 2010 | *Caranx ignobilis* | JX261496 | DBMF-M704 |
| Cign5 | Philippines: Batangas, Calabarzon, Taal Lake, Butong | 2010 | *Caranx ignobilis* | HQ654676 | [BTL066-10.COI-5P](http://www.boldsystems.org/connectivity/specimenlookup.php?processid=BTL066-10.COI-5P) |
| Cign4 | Philippines: Batangas, Calabarzon, Taal Lake, Butong | 2010 | *Caranx ignobilis* | HQ654677 | [BTL065-10.COI-5P](http://www.boldsystems.org/connectivity/specimenlookup.php?processid=BTL065-10.COI-5P) |
| Cign3 | Philippines: Batangas, Calabarzon, Taal Lake, Butong | 2010 | *Caranx ignobilis* | HQ654678 | [BTL064-10.COI-5P](http://www.boldsystems.org/connectivity/specimenlookup.php?processid=BTL064-10.COI-5P) |
| Cign2 | Philippines: Batangas, Calabarzon, Taal Lake, Butong | 2010 | *Caranx ignobilis* | HQ654679 | [BTL063-10.COI-5P](http://www.boldsystems.org/connectivity/specimenlookup.php?processid=BTL063-10.COI-5P) |
| Cign1 | Philippines: Batangas, Calabarzon, Taal Lake, Butong | 2010 | *Caranx ignobilis* | HQ654680 | [BTL062-10.COI-5P](http://www.boldsystems.org/connectivity/specimenlookup.php?processid=BTL062-10.COI-5P) |
| ADC 210.17-1 | South Africa: Kwazulu Natal | 2004 | *Caranx ignobilis* | DQ884975 | [TZMSB094-04](http://www.boldsystems.org/connectivity/specimenlookup.php?processid=TZMSB094-04) |
| ADC 210.17-3 | South Africa: Kwazulu Natal | 2004 | *Caranx ignobilis* | DQ884976 | [TZMSC059-05](http://www.boldsystems.org/connectivity/specimenlookup.php?processid=TZMSC059-05) |
| ADC 210.17-4 | South Africa: Kwazulu Natal | 2004 | *Caranx ignobilis* | DQ884977 | [TZMSC060-05](http://www.boldsystems.org/connectivity/specimenlookup.php?processid=TZMSC060-05) |
| BIOUG<CAN>:BW-A1440 | Australia: Queensland | 1998 | *Caranx ignobilis* | DQ885071 | [FOAC441-05](http://www.boldsystems.org/connectivity/specimenlookup.php?processid=FOAC441-05) |
| BIOUG<CAN>:BW-A1436 | Australia: Queensland | 1998 | *Caranx ignobilis* | DQ885072 | [FOAC437-05](http://www.boldsystems.org/connectivity/specimenlookup.php?processid=FOAC437-05) |
| BIOUG<CAN>:BW-A1437 | Australia: Queensland | 1998 | *Caranx ignobilis* | DQ885073 | [FOAC438-05](http://www.boldsystems.org/connectivity/specimenlookup.php?processid=FOAC438-05) |
| BIOUG<CAN>:BW-A1438 | Australia: Queensland | 1998 | *Caranx ignobilis* | DQ885074 | [FOAC439-05](http://www.boldsystems.org/connectivity/specimenlookup.php?processid=FOAC439-05) |
| BPBM 39590; PCMB B415 | USA: Hawaii, Oahu, off Kahuku | 2004 | *Caranx ignobilis* | DQ427060 | N/A |
| MBIO1858.4 | French Polynesia: Society Islands, Moorea, Cook bays | 2006 | *Caranx ignobilis* | JQ431540 | [MBFB037-07.COI-5P](http://www.boldsystems.org/connectivity/specimenlookup.php?processid=MBFB037-07.COI-5P) |
| Smith 210.17 #6_05 | South Africa: Cape Videl | 2006 | *Caranx ignobilis* | JF493038 | [DSFSE302-07.COI-5P](http://www.boldsystems.org/connectivity/specimenlookup.php?processid=DSFSE302-07.COI-5P) |
| NBFGR:11803D | India | N/A | *Caranx ignobilis* | FJ347936 | N/A |
| WL-M53 | India | N/A | *Caranx ignobilis* | EU014220 | N/A |
| WL-M54 | India | N/A | *Caranx ignobilis* | EU014221 | N/A |
| BP02-01 | Bagan Panchor, Peninsular Malaysia (BP) | 2009 | *Caranx sexfasciatus* | HQ560961 | DBMF-M21 |
| BP11-01 | Bagan Panchor, Peninsular Malaysia (BP) | 2009 | *Caranx sexfasciatus* | HQ560966 | DBMF-M30 |
| KP05-01 | Kuala Perlis, Peninsular Malaysia (KP) | 2009 | *Caranx sexfasciatus* | HQ560947 | DBMF-M5 |
| UMTF03881 | Sandakan, Sabah (SDK) | 2010 | *Caranx sexfasciatus* | JX261315 | DBMF-M633 |
| UMTF03639 | Mukah, Sarawak (MKS) | 2010 | *Caranx sexfasciatus* | JX261569 | DBMF-M391 |
| UMTF03880 | Sandakan, Sabah (SDK) | 2010 | *Caranx sexfasciatus* | JX261414 | DBMF-M632 |
| UMTF03641 | Mukah, Sarawak (MKS) | 2010 | *Caranx sexfasciatus* | JX261464 | DBMF-M393 |
| UMTF03642 | Mukah, Sarawak (MKS) | 2010 | *Caranx sexfasciatus* | JX261259 | DBMF-M394 |
| ADC09_210.22#4 | Mozambique: Pomene | 2008 | *Caranx sexfasciatus* | JF493042 | [DSFSF026-09.COI-5P](http://www.boldsystems.org/connectivity/specimenlookup.php?processid=DSFSF026-09.COI-5P) |
| ADC 210.22-2 | South Africa: KwaZulu-Natal, Mpenjati Estuary | 2004 | *Caranx sexfasciatus* | JF493043 | [TZMSB099-04.COI-5P](http://www.boldsystems.org/connectivity/specimenlookup.php?processid=TZMSB099-04.COI-5P) |
| Smith 210.22 #3_05 | South Africa: Park Rynie | 2007 | *Caranx sexfasciatus* | JF493044 | [DSFSE215-07.COI-5P](http://www.boldsystems.org/connectivity/specimenlookup.php?processid=DSFSE215-07.COI-5P) |
| MBIO836.4 | French Polynesia: Society Islands, Moorea, Haapiti | 2006 | *Caranx sexfasciatus* | JQ431548 | [MBFA507-07.COI-5P](http://www.boldsystems.org/connectivity/specimenlookup.php?processid=MBFA507-07.COI-5P) |
| MBIO835.4 | French Polynesia: Society Islands, Moorea, Haapiti | 2006 | *Caranx sexfasciatus* | JQ431549 | [MBFA506-07.COI-5P](http://www.boldsystems.org/connectivity/specimenlookup.php?processid=MBFA506-07.COI-5P) |
| MBIO1860.4 | French Polynesia: Society Islands, Moorea, Cook bays | 2006 | *Caranx sexfasciatus* | JQ431550 | [MBFB039-07.COI-5P](http://www.boldsystems.org/connectivity/specimenlookup.php?processid=MBFB039-07.COI-5P) |
| BPBM 39581; PCMB B416 | USA: Hawaii | 2004 | *Caranx sexfasciatus* | DQ427061 | N/A |
| BIOUG<CAN>:BW-A1446 | Australia: Queensland | 1995 | *Caranx sexfasciatus* | EF609305 | [FOAC447-05](http://www.boldsystems.org/connectivity/specimenlookup.php?processid=FOAC447-05) |
| GGAJ2 | Japan: Nagasaki, Nagasaki, Teguma | 2005 | *Caranx sexfasciatus* | JF952696 | [ABFJ208-07.COI-5P](http://www.boldsystems.org/connectivity/specimenlookup.php?processid=ABFJ208-07.COI-5P) |
| GGAJ1 | Japan: Nagasaki, Nagasaki, Teguma | 2005 | *Caranx sexfasciatus* | JF952695 | [ABFJ207-07.COI-5P](http://www.boldsystems.org/connectivity/specimenlookup.php?processid=ABFJ207-07.COI-5P) |
| MBIO1861.4 | French Polynesia: Society Islands, Moorea, Cook bays | 2006 | *Caranx sexfasciatus* | JQ431547 | [MBFB040-07.COI-5P](http://www.boldsystems.org/connectivity/specimenlookup.php?processid=MBFB040-07.COI-5P) |
| NPPF1158 | Nayband National Park Coast, Iran | 2009 | *Caranx sexfasciatus* | HQ149821 | NPPF1158 |
| MBIO1191.4 | French Polynesia: Society Islands, Moorea, SE Opunohu Bay | 2006 | *Caranx sexfasciatus* | JQ431546 | [MBFA701-07.COI-5P](http://www.boldsystems.org/connectivity/specimenlookup.php?processid=MBFA701-07.COI-5P) |
| Csex5 | Philippines: Batangas, Calabarzon, Taal Lake, Talisay | 2010 | *Caranx sexfasciatus* | HQ654682 | [BTL075-10.COI-5P](http://www.boldsystems.org/connectivity/specimenlookup.php?processid=BTL075-10.COI-5P) |
| Csex3 | Philippines: Batangas, Calabarzon, Taal Lake, Butong | 2010 | *Caranx sexfasciatus* | HQ654684 | [BTL073-10.COI-5P](http://www.boldsystems.org/connectivity/specimenlookup.php?processid=BTL073-10.COI-5P) |
| Csex4 | Philippines: Batangas, Calabarzon, Taal Lake, Butong | 2010 | *Caranx sexfasciatus* | HQ654683 | [BTL074-10.COI-5P](http://www.boldsystems.org/connectivity/specimenlookup.php?processid=BTL074-10.COI-5P) |
| Csex2 | Philippines: Batangas, Calabarzon, Taal Lake, Butong | 2010 | *Caranx sexfasciatus* | HQ654685 | [BTL072-10.COI-5P](http://www.boldsystems.org/connectivity/specimenlookup.php?processid=BTL072-10.COI-5P) |
| Csex8 | Philippines: Batangas, Calabarzon, Taal Lake, Talisay |  | *Carans sexfasciatus* | HQ654681 | [BTL076-10.COI-5P](http://www.boldsystems.org/connectivity/specimenlookup.php?processid=BTL076-10.COI-5P) |
| UMTF03951 | Tawau, Sabah (TW) | 2010 | *Caranx tille* | JX261631 | DBMF-M703 |
| UMTF04024 | Kota Kinabalu, Sabah (KKJ) | 2010 | *Caranx tille* | JX261373 | DBMF-M776 |
| UMTF04023 | Kota Kinabalu, Sabah (KKJ) | 2010 | *Caranx tille* | JX261220 | DBMF-M775 |
| UMTF04022 | Kota Kinabalu, Sabah (KKJ) | 2010 | *Caranx tille* | JX261324 | DBMF-M774 |
| UMTF04021 | Kota Kinabalu, Sabah (KKJ) | 2010 | *Caranx tille* | JX261587 | DBMF-M773 |
| UMTF04020 | Kota Kinabalu, Sabah (KKJ) | 2010 | *Caranx tille* | JX261563 | DBMF-M772 |
| UMTF03955 | Tawau, Sabah (TW) | 2010 | *Caranx tille* | JX261205 | DBMF-M707 |
| UMTF03954 | Tawau, Sabah (TW) | 2010 | *Caranx tille* | JX261272 | DBMF-M706 |
| UMTF03882 | Sandakan, Sabah (SDK) | 2010 | *Caranx tille* | JX261274 | DBMF-M634 |
| ADC09_210.23#1 | South Africa: Tugela Banks | 2009 | *Caranx tille* | GU805027 | [DSFSF433-09.COI-5P](http://www.boldsystems.org/connectivity/specimenlookup.php?processid=DSFSF433-09.COI-5P) |
| UMTF03900 | Semporna, Sabah (SMP) | 2010 | *Decapterus kurroides* | JX261421 | DBMF-M652 |
| UMTF03901 | Semporna, Sabah (SMP) | 2010 | *Decapterus kurroides* | JX261337 | DBMF-M653 |
| UMTF03898 | Semporna, Sabah (SMP) | 2010 | *Decapterus kurroides* | JX261066 | DBMF-M650 |
| UMTF03897 | Semporna, Sabah (SMP) | 2010 | *Decapterus kurroides* | JX261377 | DBMF-M649 |
| UMTF03920 | Tawau, Sabah (TW) | 2010 | *Decapterus kurroides* | JX261617 | DBMF-M672 |
| UMTF03919 | Tawau, Sabah (TW) | 2010 | *Decapterus kurroides* | JX261180 | DBMF-M671 |
| UMTF03916 | Tawau, Sabah (TW) | 2010 | *Decapterus kurroides* | JX261572 | DBMF-M668 |
| UMTF03917 | Tawau, Sabah (TW) | 2010 | *Decapterus kurroides* | JX261123 | DBMF-M669 |
| UMTF03899 | Semporna, Sabah (SMP) | 2010 | *Decapterus kurroides* | JX261107 | DBMF-M651 |
| UMTF04003 | Kota Kinabalu, Sabah (KKJ) | 2010 | *Decapterus macrosoma* | JX261016 | DBMF-M755 |
| UMTF04004 | Kota Kinabalu, Sabah (KKJ) | 2010 | *Decapterus macrosoma* | JX261160 | DBMF-M756 |
| UMTF03529 | Pulau Kambing, Peninsular Malaysia (PK) | 2010 | *Decapterus macrosoma* | JX261441 | DBMF-M281 |
| UMTF03528 | Pulau Kambing, Peninsular Malaysia (PK) | 2010 | *Decapterus macrosoma* | JX261499 | DBMF-M280 |
| UMTF03527 | Pulau Kambing, Peninsular Malaysia (PK) | 2010 | *Decapterus macrosoma* | JX261515 | DBMF-M279 |
| UMTF03526 | Pulau Kambing, Peninsular Malaysia (PK) | 2010 | *Decapterus macrosoma* | JX261215 | DBMF-M278 |
| UMTF03525 | Pulau Kambing, Peninsular Malaysia (PK) | 2010 | *Decapterus macrosoma* | JX261389 | DBMF-M277 |
| UMTF03921 | Tawau, Sabah (TW) | 2010 | *Decapterus macrosoma* | JX261134 | DBMF-M673 |
| UMTF03922 | Tawau, Sabah (TW) | 2010 | *Decapterus macrosoma* | JX261629 | DBMF-M674 |
| UMTF03796 | Kudat, Sabah (KDT) | 2010 | *Decapterus macrosoma* | JX261203 | DBMF-M548 |
| UMTF03797 | Kudat, Sabah (KDT) | 2010 | *Decapterus macrosoma* | JX261033 | DBMF-M549 |
| UMTF03798 | Kudat, Sabah (KDT) | 2010 | *Decapterus macrosoma* | JX261596 | DBMF-M550 |
| UMTF03923 | Tawau, Sabah (TW) | 2010 | *Decapterus macrosoma* | JX261514 | DBMF-M675 |
| KP06-01 | Kuala Perlis, Peninsular Malaysia (KP) | 2009 | *Decapterus macrosoma* | HQ560948 | DBMF-M6 |
| UMTF03799 | Kudat, Sabah (KDT) | 2010 | *Decapterus macrosoma* | JX261243 | DBMF-M551 |
| UMTF03924 | Tawau, Sabah (TW) | 2010 | *Decapterus macrosoma* | JX260997 | DBMF-M676 |
| UMTF03925 | Tawau, Sabah (TW) | 2010 | *Decapterus macrosoma* | JX261534 | DBMF-M677 |
| UMTF04000 | Kota Kinabalu, Sabah (KKJ) | 2010 | *Decapterus macrosoma* | JX261216 | DBMF-M752 |
| UMTF03861 | Sandakan, Sabah (SDK) | 2010 | *Decapterus macrosoma* | JX261442 | DBMF-M613 |
| UMTF03860 | Sandakan, Sabah (SDK) | 2010 | *Decapterus macrosoma* | JX261121 | DBMF-M612 |
| UMTF03859 | Sandakan, Sabah (SDK) | 2010 | *Decapterus macrosoma* | JX261269 | DBMF-M611 |
| UMTF03858 | Sandakan, Sabah (SDK) | 2010 | *Decapterus macrosoma* | JX261248 | DBMF-M610 |
| UMTF03857 | Sandakan, Sabah (SDK) | 2010 | *Decapterus macrosoma* | JX261126 | DBMF-M609 |
| UMTF04001 | Kota Kinabalu, Sabah (KKJ) | 2010 | *Decapterus macrosoma* | JX261449 | DBMF-M753 |
| UMTF04002 | Kota Kinabalu, Sabah (KKJ) | 2010 | *Decapterus macrosoma* | JX261170 | DBMF-M754 |
| UMTF03795 | Kudat, Sabah (KDT) | 2010 | *Decapterus macrosoma* | JX261519 | DBMF-M547 |
| ADC 210.27-3 | South Africa: KwaZulu-Natal, Durban | 2004 | *Decapterus macrosoma* | JF493342 | [TZMSB169-04.COI-5P](http://www.boldsystems.org/connectivity/specimenlookup.php?processid=TZMSB169-04.COI-5P) |
| ADC 210.27-2 | South Africa: KwaZulu-Natal, Durban | 2004 | *Decapterus macrosoma* | JF493343 | [TZMSB168-04.COI-5P](http://www.boldsystems.org/connectivity/specimenlookup.php?processid=TZMSB168-04.COI-5P) |
| ADC09_210.27#8 | Mozambique: Pomene | 2008 | *Decapterus macrosoma* | JF493341 | [DSFSF018-09.COI-5P](http://www.boldsystems.org/connectivity/specimenlookup.php?processid=DSFSF018-09.COI-5P) |
| ADC09_210.27#6 | Mozambique: Pomene | 2008 | *Decapterus macrosoma* | JF493340 | [DSFSF114-09.COI-5P](http://www.boldsystems.org/connectivity/specimenlookup.php?processid=DSFSF114-09.COI-5P) |
| Smith 210.27-5 | South Africa: KwaZulu-Natal, Park Rynie | 2005 | *Decapterus macrosoma* | JF493346 | [TZMSC462-05.COI-5P](http://www.boldsystems.org/connectivity/specimenlookup.php?processid=TZMSC462-05.COI-5P) |
| ADC210.27-1 | South Africa: KwaZulu-Natal, Park Rynie | 2003 | *Decapterus macrosoma* | JF493344 | [TZMSA181-04.COI-5P](http://www.boldsystems.org/connectivity/specimenlookup.php?processid=TZMSA181-04.COI-5P) |
| Smith 210.27-4 | South Africa: KwaZulu-Natal, Park Rynie | 2005 | *Decapterus macrosoma* | JF493345 | [TZMSC461-05.COI-5P](http://www.boldsystems.org/connectivity/specimenlookup.php?processid=TZMSC461-05.COI-5P) |
| UMTF03386 | Mersing, Peninsular Malaysia (MG) | 2010 | *Decapterus maruadsi* | JX261074 | DBMF-M138 |
| UMTF03385 | Mersing, Peninsular Malaysia (MG) | 2010 | *Decapterus maruadsi* | JX261320 | DBMF-M137 |
| UMTF03384 | Mersing, Peninsular Malaysia (MG) | 2010 | *Decapterus maruadsi* | JX261589 | DBMF-M136 |
| UMTF03382 | Mersing, Peninsular Malaysia (MG) | 2010 | *Decapterus maruadsi* | JX261479 | DBMF-M134 |
| UMTF04087 | Kuala Perlis, Peninsular Malaysia (KP) | 2010 | *Decapterus maruadsi* | JX261196 | DBMF-M839 |
| UMTF03661 | Kuching, Sarawak (KC) | 2010 | *Decapterus maruadsi* | JX261400 | DBMF-M413 |
| UMTF03662 | Kuching, Sarawak (KC) | 2010 | *Decapterus maruadsi* | JX261183 | DBMF-M414 |
| UMTF03663 | Kuching, Sarawak (KC) | 2010 | *Decapterus maruadsi* | JX261283 | DBMF-M415 |
| UMTF03664 | Kuching, Sarawak (KC) | 2010 | *Decapterus maruadsi* | JX261177 | DBMF-M416 |
| UMTF03665 | Kuching, Sarawak (KC) | 2010 | *Decapterus maruadsi* | JX261197 | DBMF-M417 |
| UMTF03389 | Tanjung Sedili, Peninsular Malaysia (TS) | 2010 | *Decapterus maruadsi* | JX261013 | DBMF-M141 |
| UMTF03390 | Tanjung Sedili, Peninsular Malaysia (TS) | 2010 | *Decapterus maruadsi* | JX261141 | DBMF-M142 |
| UMTF04088 | Kuala Perlis, Peninsular Malaysia (KP) | 2010 | *Decapterus maruadsi* | JX261278 | DBMF-M840 |
| UMTF03452 | Kuantan, Peninsular Malaysia (KN) | 2010 | *Decapterus maruadsi* | JX261048 | DBMF-M204 |
| UMTF03391 | Tanjung Sedili, Peninsular Malaysia (TS) | 2010 | *Decapterus maruadsi* | JX261150 | DBMF-M143 |
| UMTF03451 | Kuantan, Peninsular Malaysia (KN) | 2010 | *Decapterus maruadsi* | JX261053 | DBMF-M203 |
| UMTF03455 | Kuantan, Peninsular Malaysia (KN) | 2010 | *Decapterus maruadsi* | JX261444 | DBMF-M207 |
| UMTF03454 | Kuantan, Peninsular Malaysia (KN) | 2010 | *Decapterus maruadsi* | JX261553 | DBMF-M206 |
| UMTF04084 | Kuala Perlis, Peninsular Malaysia (KP) | 2010 | *Decapterus maruadsi* | JX261425 | DBMF-M836 |
| KSB11-01 | Kuala Sg. Besar, Peninsular Malaysia (KSB) | 2009 | *Decapterus maruadsi* | HQ560993 | DBMF-M63 |
| UMTF04085 | Kuala Perlis, Peninsular Malaysia (KP) | 2010 | *Decapterus maruadsi* | JX261260 | DBMF-M837 |
| UMTF04086 | Kuala Perlis, Peninsular Malaysia (KP) | 2010 | *Decapterus maruadsi* | JX261140 | DBMF-M838 |
| UMTF03388 | Tanjung Sedili, Peninsular Malaysia (TS) | 2010 | *Decapterus maruadsi* | JX261169 | DBMF-M140 |
| UMTF03387 | Tanjung Sedili, Peninsular Malaysia (TS) | 2010 | *Decapterus maruadsi* | JX261397 | DBMF-M139 |
| UMTF04078 | Kuala Perlis, Peninsular Malaysia (KP) | 2010 | *Elagatis bipinnulata* | JX261166 | DBMF-M830 |
| UMTF03892 | Semporna, Sabah (SMP) | 2010 | *Elagatis bipinnulata* | JX261525 | DBMF-M644 |
| UMTF03895 | Semporna, Sabah (SMP) | 2009 | *Elagatis bipinnulata* | JX261619 | DBMF-M647 |
| UMTF04074 | Kuala Perlis, Peninsular Malaysia (KP) | 2010 | *Elagatis bipinnulata* | JX261544 | DBMF-M826 |
| UMTF03896 | Semporna, Sabah (SMP) | 2010 | *Elagatis bipinnulata* | JX261002 | DBMF-M648 |
| UMTF04076 | Kuala Perlis, Peninsular Malaysia (KP) | 2010 | *Elagatis bipinnulata* | JX261521 | DBMF-M828 |
| UMTF04077 | Kuala Perlis, Peninsular Malaysia (KP) | 2010 | *Elagatis bipinnulata* | JX261189 | DBMF-M829 |
| UMTF03894 | Semporna, Sabah (SMP) | 2010 | *Elagatis bipinnulata* | JX261506 | DBMF-M646 |
| ADC210.31-1 | South Africa: KwaZulu-Natal, Park Rynie | 2004 | *Elagatis bipinnulata* | JF493407 | [TZMSC069-05.COI-5P](http://www.boldsystems.org/connectivity/specimenlookup.php?processid=TZMSC069-05.COI-5P) |
| Smith 210.31 #3_05 | South Africa: Park Rynie | 2007 | *Elagatis bipinnulata* | JF493408 | [DSFSE202-07.COI-5P](http://www.boldsystems.org/connectivity/specimenlookup.php?processid=DSFSE202-07.COI-5P) |
| Smith 210.31-2 | South Africa: KwaZulu-Natal, Park Rynie | 2005 | *Elagatis bipinnulata* | JF493409 | [TZMSC469-05.COI-5P](http://www.boldsystems.org/connectivity/specimenlookup.php?processid=TZMSC469-05.COI-5P) |
| MBIO1297.4 | French Polynesia: Society Islands, Moorea | 2006 | *Elagatis bipinnulata* | JQ431698 | [MBFA781-07.COI-5P](http://www.boldsystems.org/connectivity/specimenlookup.php?processid=MBFA781-07.COI-5P) |
| WL-M82 | India | N/A | *Elagatis bipinnulata* | EU014211 | N/A |
| WL-M83 | India | N/A | *Elagatis bipinnulata* | EU014212 | N/A |
| WL-M84 | India | N/A | *Elagatis bipinnulata* | EU014213 | N/A |
| WL-M85 | India | N/A | *Elagatis bipinnulata* | EU014214 | N/A |
| WL-M86 | India | N/A | *Elagatis bipinnulata* | EU014215 | N/A |
| MFL882 | Mexico: Quintana Roo, Xcalak | 2005 | *Elagatis bipinnulata* | GU224776 | [MFLII562-07.COI-5P](http://www.boldsystems.org/connectivity/specimenlookup.php?processid=MFLII562-07.COI-5P) |
| BIOUG<CAN>:BW-A1417 | Australia: Queensland | 2000 | *Elagatis bipinnulata* | EF609345 | [FOAC418-05](http://www.boldsystems.org/connectivity/specimenlookup.php?processid=FOAC418-05) |
| UMTF03758 | Kudat, Sabah (KDT) | 2010 | *Gnathanodon speciosus* | JX261245 | DBMF-M510 |
| UMTF03759 | Kudat, Sabah (KDT) | 2010 | *Gnathanodon speciosus* | JX261536 | DBMF-M511 |
| UMTF03756 | Kudat, Sabah (KDT) | 2010 | *Gnathanodon speciosus* | JX261186 | DBMF-M508 |
| UMTF03757 | Kudat, Sabah (KDT) | 2010 | *Gnathanodon speciosus* | JX261431 | DBMF-M509 |
| NPPF1121 | Iran: Bushehr, Nayband National Park Coast | 2009 | *Gnathanodon speciosus* | HQ149855 | NPPF1121 |
| NPPF1032 | Iran: Bushehr, Nayband National Park Coast | 2009 | *Gnathanodon speciosus* | HQ149856 | NPPF1032 |
| ADC08 Smith 210.32 #1 | Mozambique: Pomene | 2008 | *Gnathanodon speciosus* | JF493544 | [DSFSE776-08.COI-5P](http://www.boldsystems.org/connectivity/specimenlookup.php?processid=DSFSE776-08.COI-5P) |
| WL-M73 | India | N/A | *Gnathanodon speciosus* | EU148563 | N/A |
| WL-M71 | India | N/A | *Gnathanodon speciosus* | EU148562 | N/A |
| WL-M70 | India | N/A | *Gnathanodon speciosus* | EU148561 | N/A |
| BIOUG<CAN>:BW-A1412 | Australia: Queensland | 1995 | *Gnathanodon speciosus* | EF609362 | [FOAC413-05](http://www.boldsystems.org/connectivity/specimenlookup.php?processid=FOAC413-05) |
| UMTF03615 | Mukah, Sarawak (MKS) | 2010 | *Megalaspis cordyla* | JX261591 | DBMF-M367 |
| UMTF03617 | Mukah, Sarawak (MKS) | 2010 | *Megalaspis cordyla* | JX261057 | DBMF-M369 |
| UMTF03618 | Mukah, Sarawak (MKS) | 2010 | *Megalaspis cordyla* | JX261574 | DBMF-M370 |
| UMTF03564 | Tok Bali, Peninsular Malaysia (TB) | 2010 | *Megalaspis cordyla* | JX261078 | DBMF-M316 |
| UMTF03563 | Tok Bali, Peninsular Malaysia (TB) | 2010 | *Megalaspis cordyla* | JX261628 | DBMF-M315 |
| UMTF03562 | Tok Bali, Peninsular Malaysia (TB) | 2010 | *Megalaspis cordyla* | JX261430 | DBMF-M314 |
| UMTF03561 | Tok Bali, Peninsular Malaysia (TB) | 2010 | *Megalaspis cordyla* | JX261050 | DBMF-M313 |
| UMTF03560 | Tok Bali, Peninsular Malaysia (TB) | 2010 | *Megalaspis cordyla* | JX261069 | DBMF-M312 |
| UMTF03931 | Tawau, Sabah (TW) | 2010 | *Megalaspis cordyla* | JX261361 | DBMF-M683 |
| UMTF03932 | Tawau, Sabah (TW) | 2010 | *Megalaspis cordyla* | JX261006 | DBMF-M684 |
| UMTF03549 | Kuala Besut, Peninsular Malaysia (KB) | 2010 | *Megalaspis cordyla* | JX261483 | DBMF-M301 |
| UMTF03548 | Kuala Besut, Peninsular Malaysia (KB) | 2010 | *Megalaspis cordyla* | JX261502 | DBMF-M300 |
| UMTF03547 | Kuala Besut, Peninsular Malaysia (KB) | 2010 | *Megalaspis cordyla* | JX261435 | DBMF-M299 |
| UMTF03546 | Kuala Besut, Peninsular Malaysia (KB) | 2010 | *Megalaspis cordyla* | JX261417 | DBMF-M298 |
| UMTF03545 | Kuala Besut, Peninsular Malaysia (KB) | 2010 | *Megalaspis cordyla* | JX261201 | DBMF-M297 |
| UMTF03933 | Tawau, Sabah (TW) | 2010 | *Megalaspis cordyla* | JX261003 | DBMF-M685 |
| UMTF03934 | Tawau, Sabah (TW) | 2010 | *Megalaspis cordyla* | JX261210 | DBMF-M686 |
| UMTF03935 | Tawau, Sabah (TW) | 2010 | *Megalaspis cordyla* | JX261522 | DBMF-M687 |
| UMTF03514 | Pulau Kambing, Peninsular Malaysia (PK) | 2010 | *Megalaspis cordyla* | JX261244 | DBMF-M266 |
| UMTF03513 | Pulau Kambing, Peninsular Malaysia (PK) | 2010 | *Megalaspis cordyla* | JX261027 | DBMF-M265 |
| UMTF03512 | Pulau Kambing, Peninsular Malaysia (PK) | 2010 | *Megalaspis cordyla* | JX261312 | DBMF-M264 |
| UMTF03511 | Pulau Kambing, Peninsular Malaysia (PK) | 2010 | *Megalaspis cordyla* | JX261319 | DBMF-M263 |
| UMTF03510 | Pulau Kambing, Peninsular Malaysia (PK) | 2010 | *Megalaspis cordyla* | JX261359 | DBMF-M262 |
| UMTF03470 | Kuala Dungun, Peninsular Malaysia (KD) | 2010 | *Megalaspis cordyla* | JX261488 | DBMF-M222 |
| UMTF03469 | Kuala Dungun, Peninsular Malaysia (KD) | 2010 | *Megalaspis cordyla* | JX261580 | DBMF-M221 |
| UMTF03468 | Kuala Dungun, Peninsular Malaysia (KD) | 2010 | *Megalaspis cordyla* | JX261584 | DBMF-M220 |
| UMTF03467 | Kuala Dungun, Peninsular Malaysia (KD) | 2010 | *Megalaspis cordyla* | JX261015 | DBMF-M219 |
| UMTF03466 | Kuala Dungun, Peninsular Malaysia (KD) | 2010 | *Megalaspis cordyla* | JX261071 | DBMF-M218 |
| UMTF03450 | Kuantan, Peninsular Malaysia (KN) | 2010 | *Megalaspis cordyla* | JX261459 | DBMF-M202 |
| UMTF03449 | Kuantan, Peninsular Malaysia (KN) | 2010 | *Megalaspis cordyla* | JX261601 | DBMF-M201 |
| UMTF03448 | Kuantan, Peninsular Malaysia (KN) | 2010 | *Megalaspis cordyla* | JX261356 | DBMF-M200 |
| UMTF03447 | Kuantan, Peninsular Malaysia (KN) | 2010 | *Megalaspis cordyla* | JX261418 | DBMF-M199 |
| UMTF03694 | Miri, Sarawak (MR) | 2010 | *Megalaspis cordyla* | JX261279 | DBMF-M446 |
| UMTF03695 | Miri, Sarawak (MR) | 2010 | *Megalaspis cordyla* | JX261208 | DBMF-M447 |
| UMTF03696 | Miri, Sarawak (MR) | 2010 | *Megalaspis cordyla* | JX261119 | DBMF-M448 |
| UMTF03697 | Miri, Sarawak (MR) | 2010 | *Megalaspis cordyla* | JX261613 | DBMF-M449 |
| UMTF03698 | Miri, Sarawak (MR) | 2010 | *Megalaspis cordyla* | JX261117 | DBMF-M450 |
| KK01-01 | Kuala Kedah, Peninsular Malaysia (KK) | 2009 | *Megalaspis cordyla* | HQ560952 | DBMF-M11 |
| KP10-01 | Kuala Perlis, Peninsular Malaysia (KP) | 2009 | *Megalaspis cordyla* | HQ560951 | DBMF-M10 |
| UMTF03406 | Tanjung Sedili, Peninsular Malaysia (TS) | 2010 | *Megalaspis cordyla* | JX261026 | DBMF-M158 |
| UMTF03405 | Tanjung Sedili, Peninsular Malaysia (TS) | 2010 | *Megalaspis cordyla* | JX261310 | DBMF-M157 |
| UMTF03404 | Tanjung Sedili, Peninsular Malaysia (TS) | 2010 | *Megalaspis cordyla* | JX261334 | DBMF-M156 |
| UMTF03403 | Tanjung Sedili, Peninsular Malaysia (TS) | 2010 | *Megalaspis cordyla* | JX261427 | DBMF-M155 |
| UMTF03402 | Tanjung Sedili, Peninsular Malaysia (TS) | 2010 | *Megalaspis cordyla* | JX261549 | DBMF-M154 |
| UMTF04025 | Kota Kinabalu, Sabah (KKJ) | 2010 | *Megalaspis cordyla* | JX261586 | DBMF-M777 |
| UMTF04027 | Kota Kinabalu, Sabah (KKJ) | 2010 | *Megalaspis cordyla* | JX261625 | DBMF-M779 |
| UMTF04028 | Kota Kinabalu, Sabah (KKJ) | 2010 | *Megalaspis cordyla* | JX261616 | DBMF-M780 |
| UMTF04029 | Kota Kinabalu, Sabah (KKJ) | 2010 | *Megalaspis cordyla* | JX261145 | DBMF-M781 |
| UMTF04064 | Kuala Perlis, Peninsular Malaysia (KP) | 2010 | *Megalaspis cordyla* | JX261358 | DBMF-M816 |
| UMTF04065 | Kuala Perlis, Peninsular Malaysia (KP) | 2010 | *Megalaspis cordyla* | JX261187 | DBMF-M817 |
| UMTF04066 | Kuala Perlis, Peninsular Malaysia (KP) | 2010 | *Megalaspis cordyla* | JX261470 | DBMF-M818 |
| UMTF04067 | Kuala Perlis, Peninsular Malaysia (KP) | 2010 | *Megalaspis cordyla* | JX261455 | DBMF-M819 |
| UMTF04068 | Kuala Perlis, Peninsular Malaysia (KP) | 2010 | *Megalaspis cordyla* | JX261068 | DBMF-M820 |
| PN03-01 | Pontian, Peninsular Malaysia (PN) | 2009 | *Megalaspis cordyla* | HQ561011 | DBMF-M83 |
| SB03-01 | Kuala Sg. Baru, Peninsular Malaysia (SB) | 2009 | *Megalaspis cordyla* | HQ561007 | DBMF-M79 |
| UMTF03738 | Kudat, Sabah (KDT) | 2010 | *Megalaspis cordyla* | JX261472 | DBMF-M490 |
| UMTF03739 | Kudat, Sabah (KDT) | 2010 | *Megalaspis cordyla* | JX261202 | DBMF-M491 |
| UMTF03740 | Kudat, Sabah (KDT) | 2010 | *Megalaspis cordyla* | JX261325 | DBMF-M492 |
| UMTF03741 | Kudat, Sabah (KDT) | 2010 | *Megalaspis cordyla* | JX261562 | DBMF-M493 |
| UMTF03742 | Kudat, Sabah (KDT) | 2010 | *Megalaspis cordyla* | JX261211 | DBMF-M494 |
| KSB13-01 | Kuala Sg. Besar, Peninsular Malaysia (KSB) | 2009 | *Megalaspis cordyla* | HQ560994 | DBMF-M65 |
| AHM15-01 | Hutan Melintang, Peninsular Malaysia (AHM) | 2009 | *Megalaspis cordyla* | HQ560983 | DBMF-M50 |
| UMTF03616 | Mukah, Sarawak (MKS) | 2010 | *Megalaspis cordyla* | JX261590 | DBMF-M368 |
| WL-M19 | India: Maharashtra | 2006 | *Megalaspis cordyla* | EF609548 | [WLIND019-07](http://www.boldsystems.org/connectivity/specimenlookup.php?processid=WLIND019-07) |
| WL-M18 | India: Maharashtra | 2006 | *Megalaspis cordyla* | EF609549 | [WLIND018-07](http://www.boldsystems.org/connectivity/specimenlookup.php?processid=WLIND018-07) |
| WL-M17 | India: Maharashtra | 2006 | *Megalaspis cordyla* | EF609550 | [WLIND017-07](http://www.boldsystems.org/connectivity/specimenlookup.php?processid=WLIND017-07) |
| WL-M16 | India: Maharashtra | 2006 | *Megalaspis cordyla* | EF609551 | [WLIND016-07](http://www.boldsystems.org/connectivity/specimenlookup.php?processid=WLIND016-07) |
| WL-M15 | India: Maharashtra | 2006 | *Megalaspis cordyla* | EF609552 | [WLIND015-07](http://www.boldsystems.org/connectivity/specimenlookup.php?processid=WLIND015-07) |
| NPPF1056 | Iran: Bushehr, Nayband National Park Coast | 2009 | *Megalaspis cordyla* | HQ149881 | NPPF1056 |
| NPPF1053 | Iran: Bushehr, Nayband National Park Coast | 2009 | *Megalaspis cordyla* | HQ149882 | NPPF1053 |
| NPPF1051 | Iran: Bushehr, Nayband National Park Coast | 2009 | *Megalaspis cordyla* | HQ149883 | NPPF1051 |
| NPPF1016 | Iran: Bushehr, Nayband National Park Coast | 2009 | *Megalaspis cordyla* | HQ149884 | NPPF1016 |
| ON8 | Japan: Yokohama, Yokosuka, Arasaki | 2006 | *Megalaspis cordyla* | JF952790 | [ABFJ246-07.COI-5P](http://www.boldsystems.org/connectivity/specimenlookup.php?processid=ABFJ246-07.COI-5P) |
| Smith 210.34 #3 | South Africa: KwaZulu-Natal | 2005 | *Megalaspis cordyla* | JF493867 | [TZMSC616-06.COI-5P](http://www.boldsystems.org/connectivity/specimenlookup.php?processid=TZMSC616-06.COI-5P) |
| Smith 210.34 #2 | South Africa: KwaZulu-Natal | 2005 | *Megalaspis cordyla* | JF493868 | [TZMSC615-06.COI-5P](http://www.boldsystems.org/connectivity/specimenlookup.php?processid=TZMSC615-06.COI-5P) |
| Smith 210.34 #1 | South Africa: KwaZulu-Natal | 2005 | *Megalaspis cordyla* | JF493869 | [TZMSC614-06.COI-5P](http://www.boldsystems.org/connectivity/specimenlookup.php?processid=TZMSC614-06.COI-5P) |
| ADC10_210.34 #4 | South Africa: Tugela Banks | 2009 | *Megalaspis cordyla* | HQ945872 | [DSFSG233-10.COI-5P](http://www.boldsystems.org/connectivity/specimenlookup.php?processid=DSFSG233-10.COI-5P) |
| ADC10_210.17 #7 | South Africa: Scottburgh | 2010 | *Megalaspis cordyla* | HQ561501 | [DSFSG166-10.COI-5P](http://www.boldsystems.org/connectivity/specimenlookup.php?processid=DSFSG166-10.COI-5P) |
| ADC09_210.34#5 | South Africa: Tugela Banks | 2009 | *Megalaspis cordyla* | GU804934 | [DSFSF706-09.COI-5P](http://www.boldsystems.org/connectivity/specimenlookup.php?processid=DSFSF706-09.COI-5P) |
| UMTF03568 | Tok Bali, Peninsular Malaysia (TB) | 2010 | *Parastromateus niger* | JX261342 | DBMF-M320 |
| HM09-01 | Hutan Melintang, Peninsular Malaysia (AHM) | 2009 | *Parastromateus niger* | HQ560977 | DBMF-M44 |
| KSB02-01 | Kuala Sg. Besar, Peninsular Malaysia (KSB) | 2009 | *Parastromateus niger* | HQ560987 | DBMF-M54 |
| SK07-01 | Sekinchan, Peninsular Malaysia (SK) | 2009 | *Parastromateus niger* | HQ561002 | DBMF-M73 |
| PN04-01 | Pontian, Peninsular Malaysia (PN) | 2009 | *Parastromateus niger* | HQ561012 | DBMF-M84 |
| UMTF03436 | Kuantan, Peninsular Malaysia (KN) | 2010 | *Parastromateus niger* | JX261395 | DBMF-M188 |
| UMTF03437 | Kuantan, Peninsular Malaysia (KN) | 2010 | *Parastromateus niger* | JX261073 | DBMF-M189 |
| UMTF03438 | Kuantan, Peninsular Malaysia (KN) | 2010 | *Parastromateus niger* | JX261184 | DBMF-M190 |
| UMTF03439 | Kuantan, Peninsular Malaysia (KN) | 2010 | *Parastromateus niger* | JX261318 | DBMF-M191 |
| UMTF03520 | Pulau Kambing, Peninsular Malaysia (PK) | 2010 | *Parastromateus niger* | JX261592 | DBMF-M272 |
| UMTF03521 | Pulau Kambing, Peninsular Malaysia (PK) | 2010 | *Parastromateus niger* | JX261125 | DBMF-M273 |
| UMTF03523 | Pulau Kambing, Peninsular Malaysia (PK) | 2010 | *Parastromateus niger* | JX261403 | DBMF-M275 |
| UMTF03524 | Pulau Kambing, Peninsular Malaysia (PK) | 2010 | *Parastromateus niger* | JX261527 | DBMF-M276 |
| UMTF03565 | Tok Bali, Peninsular Malaysia (TB) | 2010 | *Parastromateus niger* | JX261380 | DBMF-M317 |
| UMTF03567 | Tok Bali, Peninsular Malaysia (TB) | 2010 | *Parastromateus niger* | JX261492 | DBMF-M319 |
| UMTF03569 | Tok Bali, Peninsular Malaysia (TB) | 2010 | *Parastromateus niger* | JX261579 | DBMF-M321 |
| UMTF03619 | Mukah, Sarawak (MKS) | 2010 | *Parastromateus niger* | JX261448 | DBMF-M371 |
| UMTF03620 | Mukah, Sarawak (MKS) | 2010 | *Parastromateus niger* | JX261239 | DBMF-M372 |
| UMTF03621 | Mukah, Sarawak (MKS) | 2010 | *Parastromateus niger* | JX261365 | DBMF-M373 |
| UMTF03622 | Mukah, Sarawak (MKS) | 2010 | *Parastromateus niger* | JX261331 | DBMF-M374 |
| UMTF03623 | Mukah, Sarawak (MKS) | 2010 | *Parastromateus niger* | JX261332 | DBMF-M375 |
| UMTF03656 | Kuching, Sarawak (KC) | 2010 | *Parastromateus niger* | JX261537 | DBMF-M408 |
| UMTF03657 | Kuching, Sarawak (KC) | 2010 | *Parastromateus niger* | JX261509 | DBMF-M409 |
| UMTF03658 | Kuching, Sarawak (KC) | 2010 | *Parastromateus niger* | JX261513 | DBMF-M410 |
| UMTF03659 | Kuching, Sarawak (KC) | 2010 | *Parastromateus niger* | JX261101 | DBMF-M411 |
| UMTF03660 | Kuching, Sarawak (KC) | 2010 | *Parastromateus niger* | JX261621 | DBMF-M412 |
| UMTF03718 | Miri, Sarawak (MR) | 2010 | *Parastromateus niger* | JX261030 | DBMF-M470 |
| UMTF03719 | Miri, Sarawak (MR) | 2010 | *Parastromateus niger* | JX261301 | DBMF-M471 |
| UMTF03720 | Miri, Sarawak (MR) | 2010 | *Parastromateus niger* | JX261627 | DBMF-M472 |
| UMTF03721 | Miri, Sarawak (MR) | 2010 | *Parastromateus niger* | JX261043 | DBMF-M473 |
| UMTF03722 | Miri, Sarawak (MR) | 2010 | *Parastromateus niger* | JX261195 | DBMF-M474 |
| UMTF03743 | Kudat, Sabah (KDT) | 2010 | *Parastromateus niger* | JX261353 | DBMF-M495 |
| UMTF03744 | Kudat, Sabah (KDT) | 2010 | *Parastromateus niger* | JX261540 | DBMF-M496 |
| UMTF03745 | Kudat, Sabah (KDT) | 2010 | *Parastromateus niger* | JX261120 | DBMF-M497 |
| UMTF03746 | Kudat, Sabah (KDT) | 2010 | *Parastromateus niger* | JX261271 | DBMF-M498 |
| UMTF03747 | Kudat, Sabah (KDT) | 2010 | *Parastromateus niger* | JX261051 | DBMF-M499 |
| UMTF03847 | Sandakan, Sabah (SDK) | 2010 | *Parastromateus niger* | JX261511 | DBMF-M599 |
| UMTF03848 | Sandakan, Sabah (SDK) | 2010 | *Parastromateus niger* | JX261326 | DBMF-M600 |
| UMTF03849 | Sandakan, Sabah (SDK) | 2010 | *Parastromateus niger* | JX261415 | DBMF-M601 |
| UMTF03850 | Sandakan, Sabah (SDK) | 2010 | *Parastromateus niger* | JX261191 | DBMF-M602 |
| UMTF03851 | Sandakan, Sabah (SDK) | 2010 | *Parastromateus niger* | JX261055 | DBMF-M603 |
| UMTF03911 | Tawau, Sabah (TW) | 2010 | *Parastromateus niger* | JX261602 | DBMF-M663 |
| UMTF03912 | Tawau, Sabah (TW) | 2010 | *Parastromateus niger* | JX261462 | DBMF-M664 |
| UMTF03913 | Tawau, Sabah (TW) | 2010 | *Parastromateus niger* | JX261493 | DBMF-M665 |
| UMTF03914 | Tawau, Sabah (TW) | 2010 | *Parastromateus niger* | JX261224 | DBMF-M666 |
| UMTF03915 | Tawau, Sabah (TW) | 2010 | *Parastromateus niger* | JX261615 | DBMF-M667 |
| UMTF04079 | Kuala Perlis, Peninsular Malaysia (KP) | 2010 | *Parastromateus niger* | JX261558 | DBMF-M831 |
| UMTF04080 | Kuala Perlis, Peninsular Malaysia (KP) | 2010 | *Parastromateus niger* | JX261463 | DBMF-M832 |
| UMTF04081 | Kuala Perlis, Peninsular Malaysia (KP) | 2010 | *Parastromateus niger* | JX261468 | DBMF-M833 |
| UMTF04082 | Kuala Perlis, Peninsular Malaysia (KP) | 2010 | *Parastromateus niger* | JX261478 | DBMF-M834 |
| UMTF04083 | Kuala Perlis, Peninsular Malaysia (KP) | 2010 | *Parastromateus niger* | JX261147 | DBMF-M835 |
| WL-M51 | India: Maharashtra | 2006 | *Parastromateus niger* | EF609567 | [WLIND051-07](http://www.boldsystems.org/connectivity/specimenlookup.php?processid=WLIND051-07) |
| WL-M50 | India: Maharashtra | 2006 | *Parastromateus niger* | EF609568 | [WLIND050-07](http://www.boldsystems.org/connectivity/specimenlookup.php?processid=WLIND050-07) |
| WL-M49 | India: Maharashtra | 2006 | *Parastromateus niger* | EF609569 | [WLIND049-07](http://www.boldsystems.org/connectivity/specimenlookup.php?processid=WLIND049-07) |
| WL-M48 | India: Maharashtra | 2006 | *Parastromateus niger* | EF609570 | [WLIND048-07](http://www.boldsystems.org/connectivity/specimenlookup.php?processid=WLIND048-07) |
| WL-M47 | India: Maharashtra | 2006 | *Parastromateus niger* | EF609571 | [WLIND047-07](http://www.boldsystems.org/connectivity/specimenlookup.php?processid=WLIND047-07) |
| Smith 210.36 #1 | South Africa: KwaZulu-Natal | 2005 | *Parastromateus niger* | JF494092 | [TZMSC617-06.COI-5P](http://www.boldsystems.org/connectivity/specimenlookup.php?processid=TZMSC617-06.COI-5P) |
| ADC09_210.36#2 | South Africa: Tugela Banks | 2009 | *Parastromateus niger* | GU804931 | [DSFSF710-09.COI-5P](http://www.boldsystems.org/connectivity/specimenlookup.php?processid=DSFSF710-09.COI-5P) |
| BW-A1422 | Australia: Queensland | 1997 | *Parastromateus niger* | EF609429 | [FOAC423-05](http://www.boldsystems.org/connectivity/specimenlookup.php?processid=FOAC423-05) |
| UMTF03983 | Tawau, Sabah (TW) | 2010 | *Scomberoides commersonnianus* | JX261298 | DBMF-M735 |
| UMTF03593 | Mukah, Sarawak (MKS) | 2010 | *Scomberoides commersonnianus* | JX261179 | DBMF-M345 |
| UMTF03592 | Mukah, Sarawak (MKS) | 2010 | *Scomberoides commersonnianus* | JX261037 | DBMF-M344 |
| UMTF03928 | Tawau, Sabah (TW) | 2010 | *Scomberoides commersonnianus* | JX261381 | DBMF-M734 |
| UMTF03981 | Tawau, Sabah (TW) | 2010 | *Scomberoides commersonnianus* | JX261255 | DBMF-M733 |
| UMTF03980 | Tawau, Sabah (TW) | 2010 | *Scomberoides commersonnianus* | JX261634 | DBMF-M732 |
| UMTF03979 | Tawau, Sabah (TW) | 2010 | *Scomberoides commersonnianus* | JX261603 | DBMF-M731 |
| UMTF03890 | Sandakan, Sabah (SDK) | 2010 | *Scomberoides commersonnianus* | JX261451 | DBMF-M642 |
| KSB12-01 | Kuala Sg. Besar, Peninsular Malaysia (KSB) | 2009 | *Scomberoides commersonnianus* | JX261439 | DBMF-M64 |
| UMTF03580 | Tok Bali, Peninsular Malaysia (TB) | 2010 | *Scomberoides commersonnianus* | JX261423 | DBMF-M332 |
| UMTF03581 | Tok Bali, Peninsular Malaysia (TB) | 2010 | *Scomberoides commersonnianus* | JX261017 | DBMF-M333 |
| UMTF03591 | Mukah, Sarawak (MKS) | 2010 | *Scomberoides commersonnianus* | JX261020 | DBMF-M343 |
| UMTF03594 | Mukah, Sarawak (MKS) | 2010 | *Scomberoides commersonnianus* | JX261487 | DBMF-M346 |
| UMTF03582 | Tok Bali, Peninsular Malaysia (TB) | 2010 | *Scomberoides commersonnianus* | JX261209 | DBMF-M334 |
| UMTF03583 | Tok Bali, Peninsular Malaysia (TB) | 2010 | *Scomberoides commersonnianus* | JX261258 | DBMF-M335 |
| UMTF03584 | Tok Bali, Peninsular Malaysia (TB) | 2010 | *Scomberoides commersonnianus* | JX261564 | DBMF-M336 |
| UMTF03590 | Mukah, Sarawak (MKS) | 2010 | *Scomberoides commersonnianus* | JX261031 | DBMF-M342 |
| NPPF1127 | Iran: Bushehr, Nayband National Park Coast | 2009 | *Scomberoides commersonnianus* | HQ149937 | NPPF1127 |
| NPPF1058 | Iran: Bushehr, Nayband National Park Coast | 2009 | *Scomberoides commersonnianus* | HQ149938 | NPPF1058 |
| NPPF1047 | Iran: Bushehr, Nayband National Park Coast | 2009 | *Scomberoides commersonnianus* | HQ149939 | NPPF1047 |
| NPPF1015 | Iran: Bushehr, Nayband National Park Coast | 2009 | *Scomberoides commersonnianus* | HQ149940 | NPPF1015 |
| ADC09_210.38#1 | Mozambique: Pomene | 2008 | *Scomberoides commersonnianus* | JF494451 | [DSFSF584-09.COI-5P](http://www.boldsystems.org/connectivity/specimenlookup.php?processid=DSFSF584-09.COI-5P) |
| ADC09_210.38#2 | Mozambique: Pomene | 2008 | *Scomberoides commersonnianus* | GU805100 | [DSFSF518-09.COI-5P](http://www.boldsystems.org/connectivity/specimenlookup.php?processid=DSFSF518-09.COI-5P) |
| BIOUG<CAN>:BW-A1487 | Australia: Western Australia | 1995 | *Scomberoides commersonnianus* | EF609456 | [FOAC488-05](http://www.boldsystems.org/connectivity/specimenlookup.php?processid=FOAC488-05) |
| UMTF03539 | Kuala Besut, Peninsular Malaysia (KB) | 2010 | *Scomberoides tala* | JX261223 | DBMF-M291 |
| UMTF03748 | Kudat, Sabah (KDT) | 2010 | *Scomberoides tala* | JX261518 | DBMF-M500 |
| UMTF03984 | Tawau, Sabah (TW) | 2010 | *Scomberoides tala* | JX261091 | DBMF-M736 |
| UMTF03344 | Mersing, Peninsular Malaysia (MG) | 2010 | *Scomberoides tala* | JX261606 | DBMF-M96 |
| UMTF03345 | Mersing, Peninsular Malaysia (MG) | 2010 | *Scomberoides tala* | JX261456 | DBMF-M97 |
| UMTF03535 | Kuala Besut, Peninsular Malaysia (KB) | 2010 | *Scomberoides tala* | JX261447 | DBMF-M287 |
| UMTF03346 | Mersing, Peninsular Malaysia (MG) | 2010 | *Scomberoides tala* | JX261316 | DBMF-M98 |
| UMTF03347 | Mersing, Peninsular Malaysia (MG) | 2010 | *Scomberoides tala* | JX261096 | DBMF-M99 |
| UMTF03348 | Mersing, Peninsular Malaysia (MG) | 2010 | *Scomberoides tala* | JX261626 | DBMF-M100 |
| UMTF03537 | Kuala Besut, Peninsular Malaysia (KB) | 2010 | *Scomberoides tala* | JX261100 | DBMF-M289 |
| UMTF03538 | Kuala Besut, Peninsular Malaysia (KB) | 2010 | *Scomberoides tala* | JX261566 | DBMF-M290 |
| UMTF03627 | Mukah, Sarawak (MKS) | 2010 | *Scomberoides tol* | JX261638 | DBMF-M379 |
| UMTF03626 | Mukah, Sarawak (MKS) | 2010 | *Scomberoides tol* | JX261598 | DBMF-M378 |
| UMTF03625 | Mukah, Sarawak (MKS) | 2010 | *Scomberoides tol* | JX261060 | DBMF-M377 |
| UMTF03628 | Mukah, Sarawak (MKS) | 2010 | *Scomberoides tol* | JX261556 | DBMF-M380 |
| UMTF03751 | Kudat, Sabah (KDT) | 2010 | *Scomberoides tol* | JX261295 | DBMF-M503 |
| UMTF03732 | Miri, Sarawak (MR) | 2010 | *Scomberoides tol* | JX261453 | DBMF-M484 |
| UMTF03731 | Miri, Sarawak (MR) | 2010 | *Scomberoides tol* | JX261112 | DBMF-M483 |
| UMTF03624 | Mukah, Sarawak (MKS) | 2010 | *Scomberoides tol* | JX261371 | DBMF-M376 |
| UMTF03729 | Miri, Sarawak (MR) | 2010 | *Scomberoides tol* | JX261477 | DBMF-M481 |
| UMTF03728 | Miri, Sarawak (MR) | 2010 | *Scomberoides tol* | JX261354 | DBMF-M480 |
| UMTF03349 | Mersing, Peninsular Malaysia (MG) | 2010 | *Scomberoides tol* | JX261236 | DBMF-M101 |
| UMTF03350 | Mersing, Peninsular Malaysia (MG) | 2010 | *Scomberoides tol* | JX261001 | DBMF-M102 |
| UMTF03351 | Mersing, Peninsular Malaysia (MG) | 2010 | *Scomberoides tol* | JX261296 | DBMF-M103 |
| UMTF03352 | Mersing, Peninsular Malaysia (MG) | 2010 | *Scomberoides tol* | JX261154 | DBMF-M104 |
| UMTF03353 | Mersing, Peninsular Malaysia (MG) | 2010 | *Scomberoides tol* | JX261321 | DBMF-M105 |
| UMTF03755 | Kudat, Sabah (KDT) | 2010 | *Scomberoides tol* | JX261387 | DBMF-M507 |
| UMTF03754 | Kudat, Sabah (KDT) | 2010 | *Scomberoides tol* | JX261609 | DBMF-M506 |
| UMTF04053 | Kuala Perlis, Peninsular Malaysia (KP) | 2010 | *Scomberoides tol* | JX261164 | DBMF-M805 |
| UMTF04051 | Kuala Perlis, Peninsular Malaysia (KP) | 2010 | *Scomberoides tol* | JX261604 | DBMF-M803 |
| UMTF04050 | Kuala Perlis, Peninsular Malaysia (KP) | 2010 | *Scomberoides tol* | JX261058 | DBMF-M802 |
| UMTF04049 | Kuala Perlis, Peninsular Malaysia (KP) | 2010 | *Scomberoides tol* | JX261231 | DBMF-M801 |
| UMTF03413 | Tanjung Sedili, Peninsular Malaysia (TS) | 2010 | *Scomberoides tol* | JX261238 | DBMF-M165 |
| UMTF03753 | Kudat, Sabah (KDT) | 2010 | *Scomberoides tol* | JX261199 | DBMF-M505 |
| UMTF03752 | Kudat, Sabah (KDT) | 2010 | *Scomberoides tol* | JX261250 | DBMF-M504 |
| UMTF03481 | Kuala Dungun, Peninsular Malaysia (KD) | 2010 | *Scomberoides tol* | JX261070 | DBMF-M233 |
| UMTF03482 | Kuala Dungun, Peninsular Malaysia (KD) | 2010 | *Scomberoides tol* | JX261559 | DBMF-M234 |
| UMTF03483 | Kuala Dungun, Peninsular Malaysia (KD) | 2010 | *Scomberoides tol* | JX261539 | DBMF-M235 |
| UMTF03484 | Kuala Dungun, Peninsular Malaysia (KD) | 2010 | *Scomberoides tol* | JX261227 | DBMF-M236 |
| UMTF03485 | Kuala Dungun, Peninsular Malaysia (KD) | 2010 | *Scomberoides tol* | JX261005 | DBMF-M237 |
| UMTF03648 | Mukah, Sarawak (MKS) | 2010 | *Scomberoides tol* | JX261450 | DBMF-M400 |
| UMTF03536 | Kuala Besut, Peninsular Malaysia (KB) | 2010 | *Scomberoides tol* | JX261285 | DBMF-M288 |
| UMTF 03730 | Miri, Sarawak (MR) | 2010 | *Scomberoides tol* | [JX261165](http://www.ncbi.nlm.nih.gov/nuccore/JX261165) | DBMF-M482 |
| NPPF1054 | Iran: Bushehr, Nayband National Park Coast | 2009 | *Scomberoides tol* | HQ149941 | NPPF1054 |
| NPPF1005 | Iran: Bushehr, Nayband National Park Coast | 2009 | *Scomberoides tol* | HQ149942 | NPPF1005 |
| GD 9086042 | China | 2006 | *Scomberoides tol* | EF607527 | [FSCS067-06](http://www.boldsystems.org/connectivity/specimenlookup.php?processid=FSCS067-06) |
| GD 9086041 | China | 2006 | *Scomberoides tol* | EF607528 | [FSCS066-06](http://www.boldsystems.org/connectivity/specimenlookup.php?processid=FSCS066-06) |
| GD 9086040 | China | 2006 | *Scomberoides tol* | EF607529 | [FSCS065-06](http://www.boldsystems.org/connectivity/specimenlookup.php?processid=FSCS065-06) |
| GD 9086039 | China | 2006 | *Scomberoides tol* | EF607530 | [FSCS064-06](http://www.boldsystems.org/connectivity/specimenlookup.php?processid=FSCS064-06) |
| GD 9086038 | China | 2006 | *Scomberoides tol* | EF607531 | [FSCS063-06](http://www.boldsystems.org/connectivity/specimenlookup.php?processid=FSCS063-06) |
| ADC 210.40-2 | South Africa: Kwazulu Natal | 2004 | *Scomberoides tol* | DQ885050 | [TZMSB200-04](http://www.boldsystems.org/connectivity/specimenlookup.php?processid=TZMSB200-04) |
| ADC09_210.40#7 | South Africa: Tugela Banks | 2009 | *Scomberoides tol* | GU804963 | [DSFSF667-09.COI-5P](http://www.boldsystems.org/connectivity/specimenlookup.php?processid=DSFSF667-09.COI-5P) |
| BIOUG<CAN>:BW-A1484 | Australia: Western Australia | 1995 | *Scomberoides tol* | DQ885124 | [FOAC485-05](http://www.boldsystems.org/connectivity/specimenlookup.php?processid=FOAC485-05) |
| BIOUG<CAN>:BW-A1485 | Australia: Queensland | 1996 | *Scomberoides tol* | DQ885123 | [FOAC486-05](http://www.boldsystems.org/connectivity/specimenlookup.php?processid=FOAC486-05) |
| ADC09_210.40 #8 | South Africa: Tugela Banks | 2009 | *Scomberoides tol* | GU804962 | [DSFSF669-09.COI-5P](http://www.boldsystems.org/connectivity/specimenlookup.php?processid=DSFSF669-09.COI-5P) |
| ADC09_210.40#6 | South Africa: Tugela Banks | 2009 | *Scomberoides tol* | GU804999 | [DSFSF464-09.COI-5P](http://www.boldsystems.org/connectivity/specimenlookup.php?processid=DSFSF464-09.COI-5P) |
| KK02-01 | Kuala Kedah, Peninsular Malaysia (KK) | 2009 | *Selar boops* | HQ560953 | DBMF-M12 |
| UMTF03376 | Mersing, Peninsular Malaysia (MG) | 2010 | *Selar boops* | JX261523 | DBMF-M128 |
| UMTF03693 | Miri, Sarawak (MR) | 2010 | *Selar boops* | JX261516 | DBMF-M445 |
| UMTF03691 | Miri, Sarawak (MR) | 2010 | *Selar boops* | JX261102 | DBMF-M443 |
| UMTF03690 | Miri, Sarawak (MR) | 2010 | *Selar boops* | JX261104 | DBMF-M442 |
| UMTF03689 | Miri, Sarawak (MR) | 2010 | *Selar boops* | JX261299 | DBMF-M441 |
| UMTF03599 | Mukah, Sarawak (MKS) | 2010 | *Selar boops* | JX261083 | DBMF-M351 |
| UMTF03598 | Mukah, Sarawak (MKS) | 2010 | *Selar boops* | JX261309 | DBMF-M350 |
| UMTF03597 | Mukah, Sarawak (MKS) | 2010 | *Selar boops* | JX261355 | DBMF-M349 |
| UMTF03596 | Mukah, Sarawak (MKS) | 2010 | *Selar boops* | JX261542 | DBMF-M348 |
| UMTF03595 | Mukah, Sarawak (MKS) | 2010 | *Selar boops* | JX261503 | DBMF-M347 |
| UMTF03906 | Semporna, Sabah (SMP) | 2010 | *Selar boops* | JX261262 | DBMF-M658 |
| UMTF03907 | Semporna, Sabah (SMP) | 2010 | *Selar boops* | JX261398 | DBMF-M659 |
| UMTF03908 | Semporna, Sabah (SMP) | 2010 | *Selar boops* | JX261198 | DBMF-M660 |
| UMTF03909 | Semporna, Sabah (SMP) | 2010 | *Selar boops* | JX261392 | DBMF-M661 |
| UMTF03910 | Semporna, Sabah (SMP) | 2010 | *Selar boops* | JX261114 | DBMF-M662 |
| UMTF03559 | Kuala Besut, Peninsular Malaysia (KB) | 2010 | *Selar boops* | JX261264 | DBMF-M311 |
| UMTF03558 | Kuala Besut, Peninsular Malaysia (KB) | 2010 | *Selar boops* | JX261085 | DBMF-M310 |
| UMTF03557 | Kuala Besut, Peninsular Malaysia (KB) | 2010 | *Selar boops* | JX261605 | DBMF-M309 |
| UMTF03556 | Kuala Besut, Peninsular Malaysia (KB) | 2010 | *Selar boops* | JX261252 | DBMF-M308 |
| UMTF03499 | Pulau Kambing, Peninsular Malaysia (PK) | 2010 | *Selar boops* | JX261375 | DBMF-M251 |
| UMTF03498 | Pulau Kambing, Peninsular Malaysia (PK) | 2010 | *Selar boops* | JX261532 | DBMF-M250 |
| UMTF03497 | Pulau Kambing, Peninsular Malaysia (PK) | 2010 | *Selar boops* | JX261393 | DBMF-M249 |
| UMTF03496 | Pulau Kambing, Peninsular Malaysia (PK) | 2010 | *Selar boops* | JX261214 | DBMF-M248 |
| UMTF03495 | Pulau Kambing, Peninsular Malaysia (PK) | 2010 | *Selar boops* | JX261314 | DBMF-M247 |
| UMTF03474 | Kuala Dungun, Peninsular Malaysia (KD) | 2010 | *Selar boops* | JX261467 | DBMF-M226 |
| UMTF03473 | Kuala Dungun, Peninsular Malaysia (KD) | 2010 | *Selar boops* | JX261059 | DBMF-M225 |
| UMTF03472 | Kuala Dungun, Peninsular Malaysia (KD) | 2010 | *Selar boops* | JX261062 | DBMF-M224 |
| UMTF03471 | Kuala Dungun, Peninsular Malaysia (KD) | 2010 | *Selar boops* | JX261291 | DBMF-M223 |
| UMTF03446 | Kuantan, Peninsular Malaysia (KN) | 2010 | *Selar boops* | JX261476 | DBMF-M198 |
| UMTF03995 | Kota Kinabalu, Sabah (KKJ) | 2010 | *Selar boops* | JX261343 | DBMF-M747 |
| UMTF03996 | Kota Kinabalu, Sabah (KKJ) | 2010 | *Selar boops* | JX261297 | DBMF-M748 |
| UMTF03997 | Kota Kinabalu, Sabah (KKJ) | 2010 | *Selar boops* | JX261093 | DBMF-M749 |
| UMTF03998 | Kota Kinabalu, Sabah (KKJ) | 2010 | *Selar boops* | JX261504 | DBMF-M750 |
| UMTF03999 | Kota Kinabalu, Sabah (KKJ) | 2010 | *Selar boops* | JX261363 | DBMF-M751 |
| UMTF03445 | Kuantan, Peninsular Malaysia (KN) | 2010 | *Selar boops* | JX261461 | DBMF-M197 |
| UMTF03375 | Mersing, Peninsular Malaysia (MG) | 2010 | *Selar boops* | JX261064 | DBMF-M127 |
| UMTF03374 | Mersing, Peninsular Malaysia (MG) | 2010 | *Selar boops* | JX261105 | DBMF-M126 |
| UMTF03373 | Mersing, Peninsular Malaysia (MG) | 2010 | *Selar boops* | JX261481 | DBMF-M125 |
| UMTF03372 | Mersing, Peninsular Malaysia (MG) | 2010 | *Selar boops* | JX261008 | DBMF-M124 |
| UMTF03930 | Tawau, Sabah (TW) | 2010 | *Selar crumenophthalmus* | JX261185 | DBMF-M682 |
| UMTF03602 | Mukah, Sarawak (MKS) | 2010 | *Selar crumenophthalmus* | JX261323 | DBMF-M354 |
| UMTF03601 | Mukah, Sarawak (MKS) | 2010 | *Selar crumenophthalmus* | JX261482 | DBMF-M353 |
| KK03-01 | Kuala Kedah, Peninsular Malaysia (KK) | 2010 | *Selar crumenophthalmus* | HQ560954 | DBMF-M13 |
| UMTF03929 | Tawau, Sabah (TW) | 2010 | *Selar crumenophthalmus* | JX261486 | DBMF-M681 |
| UMTF03928 | Tawau, Sabah (TW) | 2010 | *Selar crumenophthalmus* | JX261327 | DBMF-M680 |
| UMTF03927 | Tawau, Sabah (TW) | 2010 | *Selar crumenophthalmus* | JX261386 | DBMF-M679 |
| UMTF03685 | Miri, Sarawak (MR) | 2010 | *Selar crumenophthalmus* | JX261524 | DBMF-M437 |
| UMTF03686 | Miri, Sarawak (MR) | 2010 | *Selar crumenophthalmus* | JX261336 | DBMF-M438 |
| UMTF03687 | Miri, Sarawak (MR) | 2010 | *Selar crumenophthalmus* | JX261307 | DBMF-M439 |
| UMTF03688 | Miri, Sarawak (MR) | 2010 | *Selar crumenophthalmus* | JX261143 | DBMF-M440 |
| UMTF03856 | Sandakan, Sabah (SDK) | 2010 | *Selar crumenophthalmus* | JX261565 | DBMF-M608 |
| UMTF03855 | Sandakan, Sabah (SDK) | 2010 | *Selar crumenophthalmus* | JX261313 | DBMF-M607 |
| UMTF03926 | Tawau, Sabah (TW) | 2010 | *Selar crumenophthalmus* | JX261247 | DBMF-M678 |
| KP01-01 | Kuala Perlis, Peninsular Malaysia (KP) | 2009 | *Selar crumenophthalmus* | HQ560945 | DBMF-M1 |
| UMTF03575 | Tok Bali, Peninsular Malaysia (TB) | 2010 | *Selar crumenophthalmus* | JX261304 | DBMF-M327 |
| UMTF03576 | Tok Bali, Peninsular Malaysia (TB) | 2010 | *Selar crumenophthalmus* | JX261242 | DBMF-M328 |
| UMTF03577 | Tok Bali, Peninsular Malaysia (TB) | 2010 | *Selar crumenophthalmus* | JX261129 | DBMF-M329 |
| UMTF03578 | Tok Bali, Peninsular Malaysia (TB) | 2010 | *Selar crumenophthalmus* | JX261458 | DBMF-M330 |
| UMTF03579 | Tok Bali, Peninsular Malaysia (TB) | 2010 | *Selar crumenophthalmus* | JX261115 | DBMF-M331 |
| UMTF03794 | Kudat, Sabah (KDT) | 2010 | *Selar crumenophthalmus* | JX261138 | DBMF-M546 |
| UMTF03793 | Kudat, Sabah (KDT) | 2010 | *Selar crumenophthalmus* | JX261080 | DBMF-M545 |
| UMTF03792 | Kudat, Sabah (KDT) | 2010 | *Selar crumenophthalmus* | JX261157 | DBMF-M544 |
| UMTF03791 | Kudat, Sabah (KDT) | 2010 | *Selar crumenophthalmus* | JX261000 | DBMF-M543 |
| UMTF03790 | Kudat, Sabah (KDT) | 2010 | *Selar crumenophthalmus* | JX261529 | DBMF-M542 |
| UMTF03444 | Kuantan, Peninsular Malaysia (KN) | 2010 | *Selar crumenophthalmus* | JX261193 | DBMF-M196 |
| UMTF03443 | Kuantan, Peninsular Malaysia (KN) | 2010 | *Selar crumenophthalmus* | JX261339 | DBMF-M195 |
| UMTF03442 | Kuantan, Peninsular Malaysia (KN) | 2010 | *Selar crumenophthalmus* | JX261204 | DBMF-M194 |
| UMTF03441 | Kuantan, Peninsular Malaysia (KN) | 2010 | *Selar crumenophthalmus* | JX261292 | DBMF-M193 |
| UMTF04005 | Kota Kinabalu, Sabah (KKJ) | 2010 | *Selar crumenophthalmus* | JX261082 | DBMF-M757 |
| UMTF04006 | Kota Kinabalu, Sabah (KKJ) | 2010 | *Selar crumenophthalmus* | JX261306 | DBMF-M758 |
| UMTF04007 | Kota Kinabalu, Sabah (KKJ) | 2010 | *Selar crumenophthalmus* | JX261182 | DBMF-M759 |
| UMTF04008 | Kota Kinabalu, Sabah (KKJ) | 2010 | *Selar crumenophthalmus* | JX261480 | DBMF-M760 |
| UMTF04009 | Kota Kinabalu, Sabah (KKJ) | 2010 | *Selar crumenophthalmus* | JX261079 | DBMF-M761 |
| UMTF03440 | Kuantan, Peninsular Malaysia (KN) | 2010 | *Selar crumenophthalmus* | JX261052 | DBMF-M192 |
| UMTF03854 | Sandakan, Sabah (SDK) | 2010 | *Selar crumenophthalmus* | JX261213 | DBMF-M606 |
| UMTF03853 | Sandakan, Sabah (SDK) | 2010 | *Selar crumenophthalmus* | JX261338 | DBMF-M605 |
| UMTF03396 | Tanjung Sedili, Peninsular Malaysia (TS) | 2010 | *Selar crumenophthalmus* | JX261294 | DBMF-M148 |
| UMTF03395 | Tanjung Sedili, Peninsular Malaysia (TS) | 2010 | *Selar crumenophthalmus* | JX261599 | DBMF-M147 |
| UMTF03394 | Tanjung Sedili, Peninsular Malaysia (TS) | 2010 | *Selar crumenophthalmus* | JX261348 | DBMF-M146 |
| UMTF03393 | Tanjung Sedili, Peninsular Malaysia (TS) | 2010 | *Selar crumenophthalmus* | JX261443 | DBMF-M145 |
| UMTF03392 | Tanjung Sedili, Peninsular Malaysia (TS) | 2010 | *Selar crumenophthalmus* | JX261630 | DBMF-M144 |
| UMTF03381 | Mersing, Peninsular Malaysia (MG) | 2010 | *Selar crumenophthalmus* | JX261322 | DBMF-M133 |
| UMTF03380 | Mersing, Peninsular Malaysia (MG) | 2010 | *Selar crumenophthalmus* | JX261571 | DBMF-M132 |
| UMTF03379 | Mersing, Peninsular Malaysia (MG) | 2010 | *Selar crumenophthalmus* | JX261232 | DBMF-M131 |
| UMTF03378 | Mersing, Peninsular Malaysia (MG) | 2010 | *Selar crumenophthalmus* | JX261452 | DBMF-M130 |
| UMTF03377 | Mersing, Peninsular Malaysia (MG) | 2010 | *Selar crumenophthalmus* | JX261554 | DBMF-M129 |
| UMTF03852 | Sandakan, Sabah (SDK) | 2010 | *Selar crumenophthalmus* | JX261438 | DBMF-M604 |
| HM01-01 | Hutan Melintang, Peninsular Malaysia (AHM) | 2009 | *Selar crumenophthalmus* | HQ560970 | DBMF-M36 |
| UMTF03604 | Mukah, Sarawak (MKS) | 2010 | *Selar crumenophthalmus* | JX261645 | DBMF-M356 |
| UMTF04054 | Kuala Perlis, Peninsular Malaysia (KP) | 2010 | *Selar crumenophthalmus* | JX261237 | DBMF-M806 |
| UMTF04055 | Kuala Perlis, Peninsular Malaysia (KP) | 2010 | *Selar crumenophthalmus* | JX260998 | DBMF-M807 |
| UMTF04056 | Kuala Perlis, Peninsular Malaysia (KP) | 2010 | *Selar crumenophthalmus* | JX261465 | DBMF-M808 |
| UMTF04057 | Kuala Perlis, Peninsular Malaysia (KP) | 2010 | *Selar crumenophthalmus* | JX261402 | DBMF-M809 |
| UMTF04058 | Kuala Perlis, Peninsular Malaysia (KP) | 2010 | *Selar crumenophthalmus* | JX261347 | DBMF-M810 |
| UMTF03600 | Mukah, Sarawak (MKS) | 2010 | *Selar crumenophthalmus* | JX261116 | DBMF-M352 |
| UMTF03671 | Kuching, Sarawak (KC) | 2010 | *Selar crumenophthalmus* | JX261303 | DBMF-M423 |
| UMTF03509 | Pulau Kambing, Peninsular Malaysia (PK) | 2010 | *Selar crumenophthalmus* | JX261022 | DBMF-M261 |
| UMTF03508 | Pulau Kambing, Peninsular Malaysia (PK) | 2010 | *Selar crumenophthalmus* | JX261087 | DBMF-M260 |
| UMTF03507 | Pulau Kambing, Peninsular Malaysia (PK) | 2010 | *Selar crumenophthalmus* | JX261311 | DBMF-M259 |
| UMTF03506 | Pulau Kambing, Peninsular Malaysia (PK) | 2010 | *Selar crumenophthalmus* | JX261422 | DBMF-M258 |
| UMTF03505 | Pulau Kambing, Peninsular Malaysia (PK) | 2010 | *Selar crumenophthalmus* | JX261277 | DBMF-M257 |
| SK06-01 | Sekinchan, Peninsular Malaysia (SK) | 2009 | *Selar crumenophthalmus* | HQ561001 | DBMF-M72 |
| BP12-01 | Bagan Panchor, Peninsular Malaysia (BP) | 2009 | *Selar crumenophthalmus* | HQ560967 | DBMF-M31 |
| UMTF03603 | Mukah, Sarawak (MKS) | 2010 | *Selar crumenophthalmus* | JX261139 | DBMF-M355 |
| UMTF03672 | Kuching, Sarawak (KC) | 2010 | *Selar crumenophthalmus* | JX261268 | DBMF-M424 |
| UMTF03673 | Kuching, Sarawak (KC) | 2010 | *Selar crumenophthalmus* | JX261230 | DBMF-M425 |
| UMTF03674 | Kuching, Sarawak (KC) | 2010 | *Selar crumenophthalmus* | JX261009 | DBMF-M426 |
| UMTF03675 | Kuching, Sarawak (KC) | 2010 | *Selar crumenophthalmus* | JX261376 | DBMF-M427 |
| UMTF03684 | Miri, Sarawak (MR) | 2010 | *Selar crumenophthalmus* | JX261547 | DBMF-M436 |
| UMTF03480 | Kuala Dungun, Peninsular Malaysia (KD) | 2010 | *Selar crumenophthalmus* | JX261036 | DBMF-M232 |
| UMTF03479 | Kuala Dungun, Peninsular Malaysia (KD) | 2010 | *Selar crumenophthalmus* | JX261568 | DBMF-M231 |
| UMTF03478 | Kuala Dungun, Peninsular Malaysia (KD) | 2010 | *Selar crumenophthalmus* | JX261391 | DBMF-M230 |
| UMTF03477 | Kuala Dungun, Peninsular Malaysia (KD) | 2010 | *Selar crumenophthalmus* | JX261384 | DBMF-M229 |
| UMTF03476 | Kuala Dungun, Peninsular Malaysia (KD) | 2010 | *Selar crumenophthalmus* | JX261611 | DBMF-M228 |
| ADC08 Smith 210.41 #5 | Mozambique: Pomene | 2008 | *Selar crumenophthalmus* | JF494491 | [DSFSE546-08.COI-5P](http://www.boldsystems.org/connectivity/specimenlookup.php?processid=DSFSE546-08.COI-5P) |
| ADC08 Smith 210.41 #4 | Mozambique: Pomene | 2008 | *Selar crumenophthalmus* | JF494492 | [DSFSE551-08.COI-5P](http://www.boldsystems.org/connectivity/specimenlookup.php?processid=DSFSE551-08.COI-5P) |
| ADC08 Smith 210.41 #1 | Mozambique: Pomene | 2008 | *Selar crumenophthalmus* | JF494493 | [DSFSE554-08.COI-5P](http://www.boldsystems.org/connectivity/specimenlookup.php?processid=DSFSE554-08.COI-5P) |
| ADC08 Smith 210.41 #2 | Mozambique: Pomene | 2008 | *Selar crumenophthalmus* | JF494494 | [DSFSE563-08.COI-5P](http://www.boldsystems.org/connectivity/specimenlookup.php?processid=DSFSE563-08.COI-5P) |
| NBFGR:SC187 | India | N/A | *Selar crumenophthalmus* | FJ347941 | N/A |
| NBFGR:SC188 | India | N/A | *Selar crumenophthalmus* | FJ347942 | N/A |
| N/A | Japan | N/A | *Selar crumenophthalmus* | AY541647 | N/A |
| NPPF1153 | Iran: Bushehr, Nayband National Park Coast | 2009 | *Selar crumenophthalmus* | HQ149944 | NPPF1153 |
| NPPF1149 | Iran: Bushehr, Nayband National Park Coast | 2009 | *Selar crumenophthalmus* | HQ149945 | NPPF1149 |
| NPPF1147 | Iran: Bushehr, Nayband National Park Coast | 2009 | *Selar crumenophthalmus* | HQ149946 | NPPF1147 |
| NPPF1142 | Iran: Bushehr, Nayband National Park Coast | 2009 | *Selar crumenophthalmus* | HQ149947 | NPPF1142 |
| NPPF1017 | Iran: Bushehr, Nayband National Park Coast | 2009 | *Selar crumenophthalmus* | HQ149948 | NPPF1017 |
| UMTF03768 | Kudat, Sabah (KDT) | 2010 | *Selaroides leptolepis* | JX261054 | DBMF-M520 |
| UMTF03724 | Miri, Sarawak (MR) | 2010 | *Selaroides leptolepis* | JX261333 | DBMF-M476 |
| UMTF03725 | Miri, Sarawak (MR) | 2010 | *Selaroides leptolepis* | JX261281 | DBMF-M477 |
| UMTF03726 | Miri, Sarawak (MR) | 2010 | *Selaroides leptolepis* | JX261498 | DBMF-M478 |
| UMTF03727 | Miri, Sarawak (MR) | 2010 | *Selaroides leptolepis* | JX261152 | DBMF-M479 |
| UMTF03363 | Mersing, Peninsular Malaysia (MG) | 2010 | *Selaroides leptolepis* | JX261646 | DBMF-M115 |
| UMTF03541 | Kuala Besut, Peninsular Malaysia (KB) | 2010 | *Selaroides leptolepis* | JX261330 | DBMF-M293 |
| UMTF03540 | Kuala Besut, Peninsular Malaysia (KB) | 2010 | *Selaroides leptolepis* | JX261388 | DBMF-M292 |
| UMTF03875 | Sandakan, Sabah (SDK) | 2010 | *Selaroides leptolepis* | JX261176 | DBMF-M627 |
| UMTF03873 | Sandakan, Sabah (SDK) | 2010 | *Selaroides leptolepis* | JX261491 | DBMF-M625 |
| UMTF03362 | Mersing, Peninsular Malaysia (MG) | 2010 | *Selaroides leptolepis* | JX261221 | DBMF-M114 |
| UMTF03361 | Mersing, Peninsular Malaysia (MG) | 2010 | *Selaroides leptolepis* | JX261585 | DBMF-M113 |
| UMTF03462 | Kuala Dungun, Peninsular Malaysia (KD) | 2010 | *Selaroides leptolepis* | JX261583 | DBMF-M214 |
| UMTF03504 | Pulau Kambing, Peninsular Malaysia (PK) | 2010 | *Selaroides leptolepis* | JX261110 | DBMF-M256 |
| UMTF03503 | Pulau Kambing, Peninsular Malaysia (PK) | 2010 | *Selaroides leptolepis* | JX261089 | DBMF-M255 |
| UMTF03502 | Pulau Kambing, Peninsular Malaysia (PK) | 2010 | *Selaroides leptolepis* | JX261167 | DBMF-M254 |
| UMTF03501 | Pulau Kambing, Peninsular Malaysia (PK) | 2010 | *Selaroides leptolepis* | JX261432 | DBMF-M253 |
| UMTF03500 | Pulau Kambing, Peninsular Malaysia (PK) | 2010 | *Selaroides leptolepis* | JX261623 | DBMF-M252 |
| SB01-01 | Kuala Sg. Baru, Peninsular Malaysia (SB) | 2009 | *Selaroides leptolepis* | HQ561005 | DBMF-M77 |
| UMTF03360 | Mersing, Peninsular Malaysia (MG) | 2010 | *Selaroides leptolepis* | JX261286 | DBMF-M112 |
| UMTF03359 | Mersing, Peninsular Malaysia (MG) | 2010 | *Selaroides leptolepis* | JX261137 | DBMF-M111 |
| UMTF03765 | Kudat, Sabah (KDT) | 2010 | *Selaroides leptolepis* | JX261308 | DBMF-M517 |
| BP15-01 | Bagan Panchor, Peninsular Malaysia (BP) | 2009 | *Selaroides leptolepis* | HQ560969 | DBMF-M34 |
| UMTF03940 | Tawau, Sabah (TW) | 2010 | *Selaroides leptolepis* | JX261440 | DBMF-M692 |
| UMTF03766 | Kudat, Sabah (KDT) | 2010 | *Selaroides leptolepis* | JX261649 | DBMF-M518 |
| UMTF03769 | Kudat, Sabah (KDT) | 2010 | *Selaroides leptolepis* | JX261099 | DBMF-M521 |
| UMTF03415 | Kuantan, Peninsular Malaysia (KN) | 2010 | *Selaroides leptolepis* | JX261038 | DBMF-M167 |
| UMTF03414 | Kuantan, Peninsular Malaysia (KN) | 2010 | *Selaroides leptolepis* | JX261032 | DBMF-M166 |
| UMTF03937 | Tawau, Sabah (TW) | 2010 | *Selaroides leptolepis* | JX261454 | DBMF-M689 |
| UMTF03938 | Tawau, Sabah (TW) | 2010 | *Selaroides leptolepis* | JX261390 | DBMF-M690 |
| UMTF03723 | Miri, Sarawak (MR) | 2010 | *Selaroides leptolepis* | JX261265 | DBMF-M475 |
| UMTF03544 | Kuala Besut, Peninsular Malaysia (KB) | 2010 | *Selaroides leptolepis* | JX261014 | DBMF-M296 |
| UMTF03543 | Kuala Besut, Peninsular Malaysia (KB) | 2010 | *Selaroides leptolepis* | JX261241 | DBMF-M295 |
| UMTF03542 | Kuala Besut, Peninsular Malaysia (KB) | 2010 | *Selaroides leptolepis* | JX261019 | DBMF-M294 |
| UMTF03936 | Tawau, Sabah (TW) | 2010 | *Selaroides leptolepis* | JX261367 | DBMF-M688 |
| UMTF03418 | Kuantan, Peninsular Malaysia (KN) | 2010 | *Selaroides leptolepis* | JX261163 | DBMF-M170 |
| UMTF03417 | Kuantan, Peninsular Malaysia (KN) | 2010 | *Selaroides leptolepis* | JX261528 | DBMF-M169 |
| UMTF03416 | Kuantan, Peninsular Malaysia (KN) | 2010 | *Selaroides leptolepis* | JX261620 | DBMF-M168 |
| GD 9086061 | China | 2006 | *Selaroides leptolepis* | EF607545 | [FSCS086-06](http://www.boldsystems.org/connectivity/specimenlookup.php?processid=FSCS086-06) |
| GD 9086060 | China | 2006 | *Selaroides leptolepis* | EF607546 | [FSCS085-06](http://www.boldsystems.org/connectivity/specimenlookup.php?processid=FSCS085-06) |
| GD 9086059 | China | 2006 | *Selaroides leptolepis* | EF607547 | [FSCS084-06](http://www.boldsystems.org/connectivity/specimenlookup.php?processid=FSCS084-06) |
| GD 9086058 | China | 2006 | *Selaroides leptolepis* | EF607548 | [FSCS083-06](http://www.boldsystems.org/connectivity/specimenlookup.php?processid=FSCS083-06) |
| GD 9081027 | China | 2006 | *Selaroides leptolepis* | EF607549 | [FSCS179-06](http://www.boldsystems.org/connectivity/specimenlookup.php?processid=FSCS179-06) |
| GD 9086062 | China | 2006 | *Selaroides leptolepis* | EF607550 | [FSCS087-06](http://www.boldsystems.org/connectivity/specimenlookup.php?processid=FSCS087-06) |
| UMTF04030 | Kota Kinabalu, Sabah (KKJ) | 2010 | *Seriola dumerili* | JX261106 | DBMF-M782 |
| UMTF04031 | Kota Kinabalu, Sabah (KKJ) | 2010 | *Seriola dumerili* | JX261426 | DBMF-M783 |
| UMTF04032 | Kota Kinabalu, Sabah (KKJ) | 2010 | *Seriola dumerili* | JX261404 | DBMF-M784 |
| UMTF04033 | Kota Kinabalu, Sabah (KKJ) | 2010 | *Seriola dumerili* | JX261257 | DBMF-M785 |
| ADC09_210.43#1 | South Africa: Pumula | 2009 | *Seriola dumerili* | JF494498 | [DSFSF133-09.COI-5P](http://www.boldsystems.org/connectivity/specimenlookup.php?processid=DSFSF133-09.COI-5P) |
| N/A | Japan: Nagasaki | 2006 | *Seriola dumerili* | NC016870 | N/A |
| N/A | Japan: Kouchi | 2006 | *Seriola dumerili* | AB517559 | N/A |
| N/A | Japan: Nagasaki | 2006 | *Seriola dumerili* | AB517558 | N/A |
| ADC10_210.43 #6 | South Africa:Park Rynie | 2010 | *Seriola dumerili* | HQ945927 | [DSFSG343-10.COI-5P](http://www.boldsystems.org/connectivity/specimenlookup.php?processid=DSFSG343-10.COI-5P) |
| N/A | Turkey | N/A | *Seriola dumerili* | JQ623993 | N/A |
| MBCSC:HN SY08340 | China: South China Sea | 2008 | *Seriola dumerili* | FJ237927 | [CFCS024-08](http://www.boldsystems.org/connectivity/specimenlookup.php?processid=CFCS024-08) |
| BIOUG<CAN>:BW-A1492 | Australia: Western Australia | 1995 | *Seriola dumerili* | EF609458 | [FOAC493-05](http://www.boldsystems.org/connectivity/specimenlookup.php?processid=FOAC493-05) |
| ADC09_210.43#4 | South Africa: Pumula | 2009 | *Seriola dumerili* | JF494496 | [DSFSF136-09.COI-5P](http://www.boldsystems.org/connectivity/specimenlookup.php?processid=DSFSF136-09.COI-5P) |
| ADC09_210.43#3 | South Africa: Pumula | 2009 | *Seriola dumerili* | JF494495 | BOLD:[DSFSF135-09.COI-5P](http://www.boldsystems.org/connectivity/specimenlookup.php?processid=DSFSF135-09.COI-5P) |
| ADC09_210.43#2 | South Africa: Pumula | 2009 | *Seriola dumerili* | JF494497 | [DSFSF134-09.COI-5P](http://www.boldsystems.org/connectivity/specimenlookup.php?processid=DSFSF134-09.COI-5P) |
| MBCSC:HN SY08576 | China: South China Sea | 2008 | *Seriola dumerili* | FJ237923 | [CFCS260-08](http://www.boldsystems.org/connectivity/specimenlookup.php?processid=CFCS260-08) |
| MBCSC:HN SY08575 | China: South China Sea | 2008 | *Seriola dumerili* | FJ237924 | [CFCS259-08](http://www.boldsystems.org/connectivity/specimenlookup.php?processid=CFCS259-08) |
| MBCSC:HN SY08574 | China: South China Sea | 2008 | *Seriola dumerili* | FJ237925 | [CFCS258-08](http://www.boldsystems.org/connectivity/specimenlookup.php?processid=CFCS258-08) |
| MBCSC:HN SY08578 | China: South China Sea | 2008 | *Seriola dumerili* | FJ237921 | [CFCS262-08](http://www.boldsystems.org/connectivity/specimenlookup.php?processid=CFCS262-08) |
| MBCSC:HN SY08358 | China: South China Sea | 2008 | *Seriola dumerili* | FJ237926 | [CFCS042-08](http://www.boldsystems.org/connectivity/specimenlookup.php?processid=CFCS042-08) |
| MBCSC:HN SY08577 | China: South China Sea | 2008 | *Seriola dumerili* | FJ237922 | [CFCS261-08](http://www.boldsystems.org/connectivity/specimenlookup.php?processid=CFCS261-08) |
| UMTF04035 | Kota Kinabalu, Sabah (KKJ) | 2010 | *Seriolina nigrofasciata* | JX261025 | DBMF-M787 |
| UMTF04036 | Kota Kinabalu, Sabah (KKJ) | 2010 | *Seriolina nigrofasciata* | JX260996 | DBMF-M788 |
| UMTF04037 | Kota Kinabalu, Sabah (KKJ) | 2010 | *Seriolina nigrofasciata* | JX261240 | DBMF-M789 |
| UMTF04038 | Kota Kinabalu, Sabah (KKJ) | 2010 | *Seriolina nigrofasciata* | JX261538 | DBMF-M790 |
| UMTF03813 | Kudat, Sabah (KDT) | 2010 | *Seriolina nigrofasciata* | JX261648 | DBMF-M567 |
| UMTF03814 | Kudat, Sabah (KDT) | 2010 | *Seriolina nigrofasciata* | JX261335 | DBMF-M568 |
| AHM17-01 | Hutan Melintang, Peninsular Malaysia (AHM) | 2009 | *Seriolina nigrofasciata* | HQ560985 | DBMF-M52 |
| UMTF03879 | Sandakan, Sabah (SDK) | 2010 | *Seriolina nigrofasciata* | JX261162 | DBMF-M631 |
| BP14-01 | Bagan Panchor, Peninsular Malaysia (BP) | 2009 | *Seriolina nigrofasciata* | HQ560968 | DBMF-M33 |
| NPPF1099 | Iran: Bushehr, Nayband National Park Coast | 2009 | *Seriolina nigrofasciata* | HQ149949 | NPPF1099 |
| NPPF1085 | Iran: Bushehr, Nayband National Park Coast | 2009 | *Seriolina nigrofasciata* | HQ149950 | NPPF1085 |
| WL-M74 | India | N/A | *Seriolina nigrofasciata* | EU014234 | N/A |
| WL-M75 | India | N/A | *Seriolina nigrofasciata* | EU014235 | N/A |
| WL-M76 | India | N/A | *Seriolina nigrofasciata* | EU014236 | N/A |
| UMTF03357 | Mersing, Peninsular Malaysia (MG) | 2010 | *Trachinotus baillonii* | JX261097 | DBMF-M109 |
| UMTF03356 | Mersing, Peninsular Malaysia (MG) | 2010 | *Trachinotus baillonii* | JX261081 | DBMF-M108 |
| UMTF03355 | Mersing, Peninsular Malaysia (MG) | 2010 | *Trachinotus baillonii* | JX261175 | DBMF-M107 |
| UMTF03354 | Mersing, Peninsular Malaysia (MG) | 2010 | *Trachinotus baillonii* | JX261383 | DBMF-M106 |
| BIOUG<CAN>:BW-A1406 | Australia: Queensland | 1999 | *Trachinotus baillonii* | EF609480 | [FOAC407-05](http://www.boldsystems.org/connectivity/specimenlookup.php?processid=FOAC407-05) |
| MBIO1276.4 | French Polynesia: Society Islands, Moorea | 2006 | *Trachinotus baillonii* | JQ432196 | [MBFA763-07.COI-5P](http://www.boldsystems.org/connectivity/specimenlookup.php?processid=MBFA763-07.COI-5P) |
| MBIO1437.4 | French Polynesia: Society Islands, Moorea | 2006 | *Trachinotus baillonii* | JQ432197 | [MBFA842-07.COI-5P](http://www.boldsystems.org/connectivity/specimenlookup.php?processid=MBFA842-07.COI-5P) |
| UMTF04071 | Kuala Perlis, Peninsular Malaysia (KP) | 2010 | *Uraspis uraspis* | JX261636 | DBMF-M823 |
| UMTF03832 | Sandakan, Sabah (SDK) | 2010 | *Uraspis uraspis* | JX261300 | DBMF-M584 |
| BP05-01 | Bagan Panchor, Peninsular Malaysia (BP) | 2009 | *Uraspis uraspis* | JX261251 | DBMF-M24 |
| AHM16-01 | Hutan Melintang, Peninsular Malaysia (AHM) | 2009 | *Uraspis uraspis* | HQ560984 | DBMF-M51 |
| UMTF04070 | Kuala Perlis, Peninsular Malaysia (KP) | 2010 | *Uraspis uraspis* | JX261028 | DBMF-M822 |
| UMTF03833 | Sandakan, Sabah (SDK) | 2010 | *Uraspis uraspis* | JX261040 | DBMF-M585 |
| UMTF04069 | Kuala Perlis, Peninsular Malaysia (KP) | 2010 | *Uraspis uraspis* | JX261408 | DBMF-M821 |
| UMTF03803 | Kudat, Sabah (KDT) | 2010 | *Uraspis uraspis* | JX261207 | DBMF-M555 |
| UMTF03816 | Kudat, Sabah (KDT) | 2010 | *Uraspis uraspis* | JX261072 | DBMF-M559 |
| UMTF03989 | Kota Kinabalu, Sabah (KKJ) | 2010 | *Uraspis uraspis* | JX261088 | DBMF-M741 |
| UMTF03988 | Kota Kinabalu, Sabah (KKJ) | 2010 | *Uraspis uraspis* | JX261042 | DBMF-M740 |
| UMTF03987 | Kota Kinabalu, Sabah (KKJ) | 2010 | *Uraspis uraspis* | JX261151 | DBMF-M739 |
| UMTF03986 | Kota Kinabalu, Sabah (KKJ) | 2010 | *Uraspis uraspis* | JX261256 | DBMF-M738 |
| UMTF04072 | Kuala Perlis, Peninsular Malaysia (KP) | 2010 | *Uraspis uraspis* | JX261168 | DBMF-M824 |
| UMTF04073 | Kuala Perlis, Peninsular Malaysia (KP) | 2010 | *Uraspis uraspis* | JX261577 | DBMF-M825 |
| UMTF03834 | Sandakan, Sabah (SDK) | 2010 | *Uraspis uraspis* | JX261212 | DBMF-M586 |
| UMTF03985 | Kota Kinabalu, Sabah (KKJ) | 2010 | *Uraspis uraspis* | JX261495 | DBMF-M737 |
| UMTF03815 | Kudat, Sabah (KDT) | 2010 | *Uraspis uraspis* | JX261541 | DBMF-M558 |
| UMTF03805 | Kudat, Sabah (KDT) | 2010 | *Uraspis uraspis* | JX261497 | DBMF-M557 |
| UMTF03835 | Sandakan, Sabah (SDK) | 2010 | *Uraspis uraspis* | JX261317 | DBMF-M587 |
| UMTF03836 | Sandakan, Sabah (SDK) | 2010 | *Uraspis uraspis* | JX261132 | DBMF-M588 |
| UMTF03804 | Kudat, Sabah (KDT) | 2010 | *Uraspis uraspis* | JX261485 | DBMF-M556 |
| NPPF1125 | Iran: Bushehr, Nayband National Park Coast | 2009 | *Uraspis uraspis* | HQ149964 | NPPF1125 |
