# Supplementary material for: DNA Barcoding Reveals Cryptic Diversity within Commercially Exploited Indo-Malay Carangidae (Teleosteii: Perciformes)
Source: PLoS One. 2012 Nov 29;7(11):e49623. doi: 10.1371/journal.pone.0049623 (PMC3510217; doi:10.1371/journal.pone.0049623)
Supplement: Table S2 — K2P distances of Indo-Malay Carangidae. (DOC) [file pone.0049623.s006.doc]

**Table S2**. A Kimura 2-parameter distances of each Indo-Malay Carangidae species.

| **Species** | **Common name** | **n** | **min** | **mean** | **max** | **SE** |
| --- | --- | --- | --- | --- | --- | --- |
| *Alectis ciliaris* | African pompano | 8 | 0 | 0.16 | 0.63 | 0.04 |
| *Alectis indicus* | Indian threadfish | 10 | 0 | 0.17 | 0.62 | 0.02 |
| *Alepes djedaba* | Shrimp scad | 31 | 0 | 0.25 | 0.62 | 0.01 |
| *Alepes kleinii* | Razorbelly scad | 11 | 0 | 0.16 | 0.46 | 0.02 |
| *Alepes melanoptera* | Blackfin scad | 15 | 0 | 0.40 | 1.65 | 0.03 |
| *Alepes vari* | Herring scad | 13 | 0 | 0.16 | 0.64 | 0.02 |
| *Atropus atropus* | Cleftbelly trevally | 13 | 0 | 1.13 | 2.68 | 0.11 |
| *Atule mate* | Yellowtail scad | 67 | 0 | 0.34 | 4.82 | 0.02 |
| *Carangoides bajad* | Orangespotted trevally | 26 | 0 | 0.39 | 1.93 | 0.02 |
| *Carangoides chrysophrys* | Longnose trevally | 19 | 0 | 0.33 | 0.81 | 0.02 |
| *Carangoides dinema* | Shadow trevally | 6 | 0 | 0.03 | 0.16 | 0.02 |
| *Carangoides ferdau* | Blue trevally | 2 |  |  |  |  |
| *Carangoides fulvoguttatus* | Yellowspotted trevally | 3 | 0 | 0.21 | 0.31 | 0.09 |
| *Carangoides gymnostethus* | Bludger | 1 |  |  |  |  |
| *Carangoides hedlandensis* | Bumpnose trevally | 3 | 0.16 | 0.31 | 0.47 | 0.07 |
| *Carangoides malabaricus* | Malabar trevally | 33 | 0 | 0.54 | 2.05 | 0.16 |
| *Caranx ignobilis* | Tille trevally | 6 | 0 | 0.506 | 1.09 | 0.09 |
| *Caranx sexfasciatus* | Redtail scad | 8 | 0 | 0.16 | 0.31 | 0.02 |
| *Caranx tille* | Shortfin scad | 9 | 0 | 0.07 | 0.31 | 0.02 |
| *Decapterus kurroides* | Round scad/ Japanese scad | 10 | 0 | 0.09 | 0.47 | 0.02 |
| *Decapterus macrosoma* | Rainbow runner | 26 | 0 | 0.08 | 0.48 | 0.01 |
| *Decapterus maruadsi* | Golden trevally | 24 | 0 | 0.15 | 0.66 | 0.01 |
| *Elagatis bipinnulata* | Torpedo scad | 8 | 0 | 0.22 | 0.63 | 0.04 |
| *Gnathanodon speciosus* | Black pomfret | 4 | 0 | 0 | 0 | 0 |
| *Megalaspis cordyla* | Talang queenfish | 63 | 0 | 0.53 | 2.06 | 0.01 |
| *Parastromateus niger* | Barred queenfish | 51 | 0 | 0.3 | 1.09 | 0.01 |
| *Scomberoides commersonnianus* | Needlescaled queenfish | 17 | 0 | 0.56 | 1.78 | 0.05 |
| *Scomberoides tala* | Oxeye scad | 11 | 0 | 0.08 | 0.34 | 0.01 |
| *Scomberoides tol* | Bigeye scad | 32 | 0 | 0.09 | 0.46 | 0.01 |
| *Selar boops* | Yellowstripe scad | 40 | 0 | 0.37 | 1.27 | 0.01 |
| *Selar crumenophthalmus* | Greater amberjack | 75 | 0 | 0.39 | 4.66 | 0.02 |
| *Selaroides leptolepis* | Blackbanded trevally | 39 | 0 | 0.18 | 1.62 | 0.01 |
| *Seriola dumerili* | Small spotted dart | 4 | 0 | 0.31 | 0.47 | 0.06 |
| *Seriolina nigrofasciata* | Snubnose pompano | 9 | 0 | 1.79 | 4.317 | 0.30 |
| *Trachinotus baillonii* | Whitemouth jack | 4 | 0 | 0 | 0 | 0 |
| *Uraspis uraspis* | Whitemouth jack | 22 | 0 | 0.67 | 1.72 | 0.04 |

*no sequence yet

n= 723
